# Visual imagination and cognitive mapping of a virtual building

**DOI:** 10.1017/S0373463321000588

**Published:** 2022-01

**Authors:** Kate Jeffery, Wanying Guo, Danny Ball, Julia Rodriguez-Sanchez

**Affiliations:** 1 Experimental Psychology, University College London, London, UK; 2 Institute of Cognitive Neuroscience, University College London, London, UK; 3 Division of Biosciences, University College London, London, UK

**Keywords:** imagined navigation, visual navigation, visual, verbal, symmetry, spatial abilities, cognitive map formation

## Abstract

We investigated the contribution of visual imagination to the cognitive mapping of a building when initial exploration was simulated either visually by using a passive video walk-through, or mentally by using verbal guidance. Building layout had repeating elements with either rotational or mirror symmetry. Cognitive mapping of the virtual building, determined using questionnaires and map drawings, was present following verbal guidance but inferior to that following video guidance. Mapping was not affected by the building's structural symmetry. However, notably, it correlated with small-scale mental rotation scores for both video and verbal guidance conditions. There was no difference between males and females. A common factor that may have influenced cognitive mapping was the availability of visual information about the relationships of the building elements, either directly perceived (during the video walk-through) or imagined (during the verbal walk-through and/or during recall). Differences in visual imagination, particularly mental rotation, may thus account for some of the individual variance in cognitive mapping of complex built environments, which is relevant to how designers provide navigation-relevant information.

## 1 Introduction

Real-world navigation requires construction of a mental, or cognitive, map of the environment (Tolman, 1948; O'Keefe and Nadel, 1978). This type of environmental knowledge is called 'configurational' because it entails placing, in a mental map, environment sub-spaces in the correct relationship (configuration) to each other, which enables flexible navigational processes such as short-cutting and detouring. Understanding how people make cognitive maps is important for designing wayfinding aids for real-world navigation, as well as for elucidating the foundations of large-scale spatial cognition.

Cognitive maps are made during real navigation when people move physically through a space, but they are also made when people are given information that lets them mentally simulate their journey. This can occur, for example, when people ask for directions and are given verbal instructions. The aim of the present study was to assess cognitive mapping of a building, using simulated navigation scenarios to allow control of the relevant parameters. The first parameter of interest was how well people can form a cognitive map of the environment layout when given visual information, in the form of a video tour, versus verbal information in the form of either written or spoken instructions. The main difference between these information sources is that with the verbal instructions, movements - translation and rotation - are not perceived directly but have to be entirely imagined, along with the change in relative locations of salient environmental features. It is an open question how well people can mentally rotate their sense of direction, with the associated updating of relative positions of environmental features that this requires. Vision on the other hand allows people to process movements, especially changes in direction, more easily, since both the rotation/translation and the new viewpoint generated by the movement are directly perceived rather than mentally constructed. Our guiding hypothesis was that cognitive mapping would be hindered if people had to imagine turning corners instead of visually perceiving the turns.

**Figure 1:**
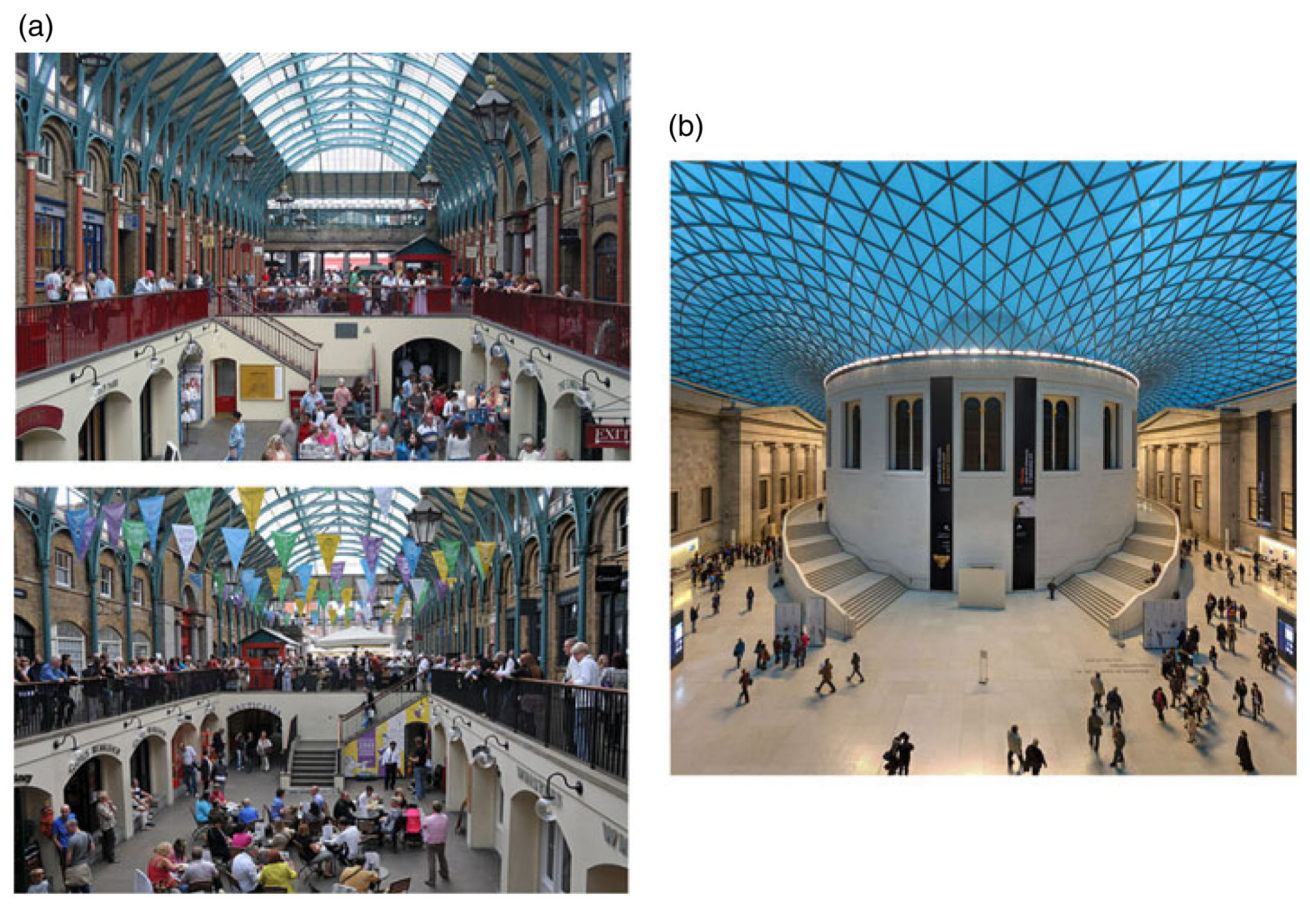
(a) Two similar views of Covent Garden Market, one facing north (upper) and one facing south (lower). There is abundant visual information to help maintain a sense of orientation except for the north/south confusion – if that coarse directional ambiguity can be resolved then fine-grained orientation using vision is enabled. (b) Mirror symmetry in a built space. Although the left side of the space is a reflection of the right, the specific view is unique to this particular facing direction and so can unambiguously inform the sense of direction.

The second factor of interest was the effect of environmental rotational symmetry, which may confound the sense of direction, on mental map quality. A problem that people often face is how to orient themselves when there are ambiguities in the layout of the environment they are in: for example, repeating sub-spaces that require knowledge of position and heading for their disambiguation. An example is given in Figure 1(a) in which the visual scene is similar whether the viewer is looking north or south towards a central walkway. To use vision to help navigate an ambiguous environment, one needs to have a stable sense of direction to know which view is which, after which vision can in turn help stabilise the direction sense.

Theoretical considerations suggest that mirror symmetry (Figure 1(b)) should be easier for heading determination because - unlike with rotational symmetry - no two directions yield the exact same visual scene, and so vision uniquely indicates facing direction. We thus compared mental mapping of two buildings with layout symmetry: in one building this was rotational and in the other it was reflectional (mirror). We also considered whether there might be an interaction between the presentation modality (visual vs verbal) and symmetry type: it might be predicted that mirror symmetry would be disruptive in the visual but not verbal tour, because of the visual ambiguities present.

Third, we were interested in sex differences that might affect mental mapping. This is relevant because previous research has suggested that males and females may navigate differently, with females more likely to rely on landmarks and males more likely to use a more global mapping strategy (Galea and Kimura, 1993; Moffat et al., 1998). There is also a literature suggesting a contribution of small-scale mental rotation ability to navigation (Blajenkova et al., 2005; Driscoll et al., 2005; Hegarty et al., 2006). We therefore included the Vandenberg and Kuse Mental Rotation Task (MRT) in our assessment of spatial characteristics, as well as a widely used self-report test of navigation ability, the Santa Barbara Sense of Direction Scale (SBSODS; Hegarty et al., 2002).

In order to provide visual information about movement we chose a video tour, presented on a desktop computer. Participants watched a video walk-through of a journey through a building (see supplemental materials on https://figshare.com/s/beba971f4e12d0c62cb1) and were then queried as to their understanding of the building layout via a questionnaire and a map-drawing exercise. For the matching non-visual condition we provided verbal information about the tour, either visual (people read the instructions) to control for sensory modality *per se,* or via an audio tour, as might be given, for example, to visually impaired people. Each participant received one video and one of the verbal tours in randomised order.

## 2 Materials and methods

### 2.1 Participants

Eighty participants (51 female, 29 male) were recruited through online platforms such as the University College London (UCL) Psychology Subject Pool (SONA) or Facebook. Pilot studies suggested that people engaged with the task better in person than online, so participants were asked to come to the laboratory at UCL, to run through the task under the guidance of an experimenter. All procedures contributing to this work complied with the ethical standards of the relevant national and institutional committees on human experimentation and with the Helsinki Declaration of 1975, as revised in 2008. All participants gave consent and the study was granted ethical approval by the UCL Department of Psychology Ethics Committee; no. EP/2018/03.

### 2.2 Design and apparatus

The experiment was designed using the online experiment design platform Gorilla Experiment Builder (www.gorilla.sc), and conducted using desktop computers to deliver the materials. A mixed design was employed, with a within-subjects comparison of differences in map learning between the video tour and the verbal (spoken and written) tours, and between the rotationally and mirror symmetric buildings, and a between-subjects design to compare the effects of spoken versus written verbal guidance. Thus, every participant received two building tours, counterbalanced in order, the first with the rotationally symmetric and the second with the mirror symmetric building. Each tour itself comprised two run-throughs, after each of which a short questionnaire (five questions) was administered. Participants 1–40 received half their tours by video and the other half by spoken audio; participants 41–80 received half by video and half by written text (also counterbalanced).

### 2.3 Environments

**Figure 2:**
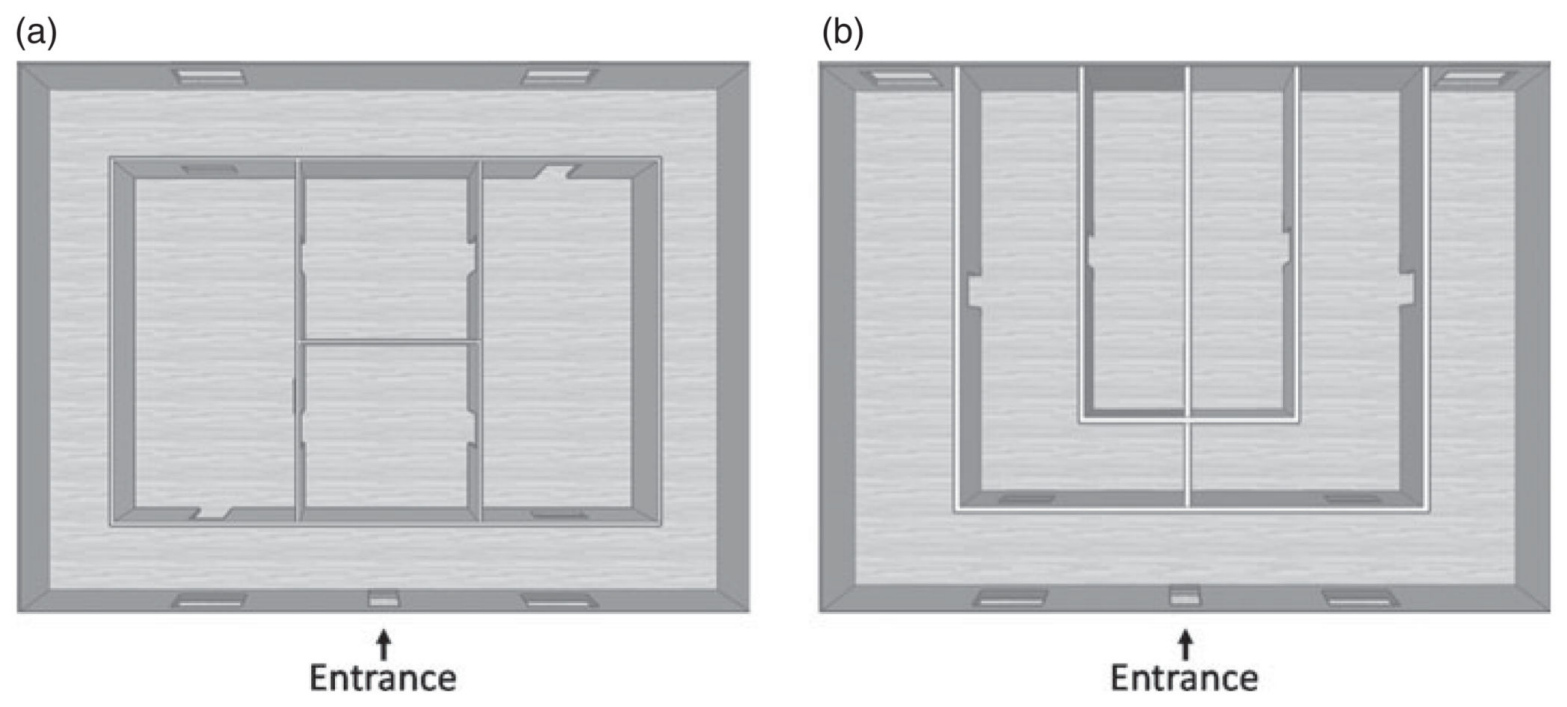
Plan (overhead) views of the two buildings. (a) The rotationally symmetric building, which was entered from the door at the bottom. It comprised a perimeter corridor and four internal rooms. Note that if wall colour is ignored, the views inside the two square rooms or the two rectangular rooms are the same and so only the sense of direction/position can be used to determine which one is which. (b) The mirror-symmetric building which also had an outside corridor (albeit not continuous) and four internal rooms. The mirror symmetry means that every room provides a unique visual guide to direction.

The stimuli presented were two virtual buildings (Figure 2), designed using SketchUp software. The building with rotational symmetry (Rot.) was designed along similar principles to those used in a recent study of rodent head direction cells (Jacob et al., 2017) and was intended to be visually ambiguous with regard to direction. To help break the symmetries and make the task slightly easier (pilot subjects had difficulty with it), the walls of each room were also painted a different colour, by analogy with how the compartments in the rodent experiment were odour-scented. The building with mirror symmetry (Mirr.) was designed to be similar in terms of complexity, with the same number of landmarks (13 doors and windows), the same layout of the environment (rectangular) and the same starting point (left corner outside the front of the building, facing down the street towards the front door).

### 2.4 Task procedure

**Figure 3:**
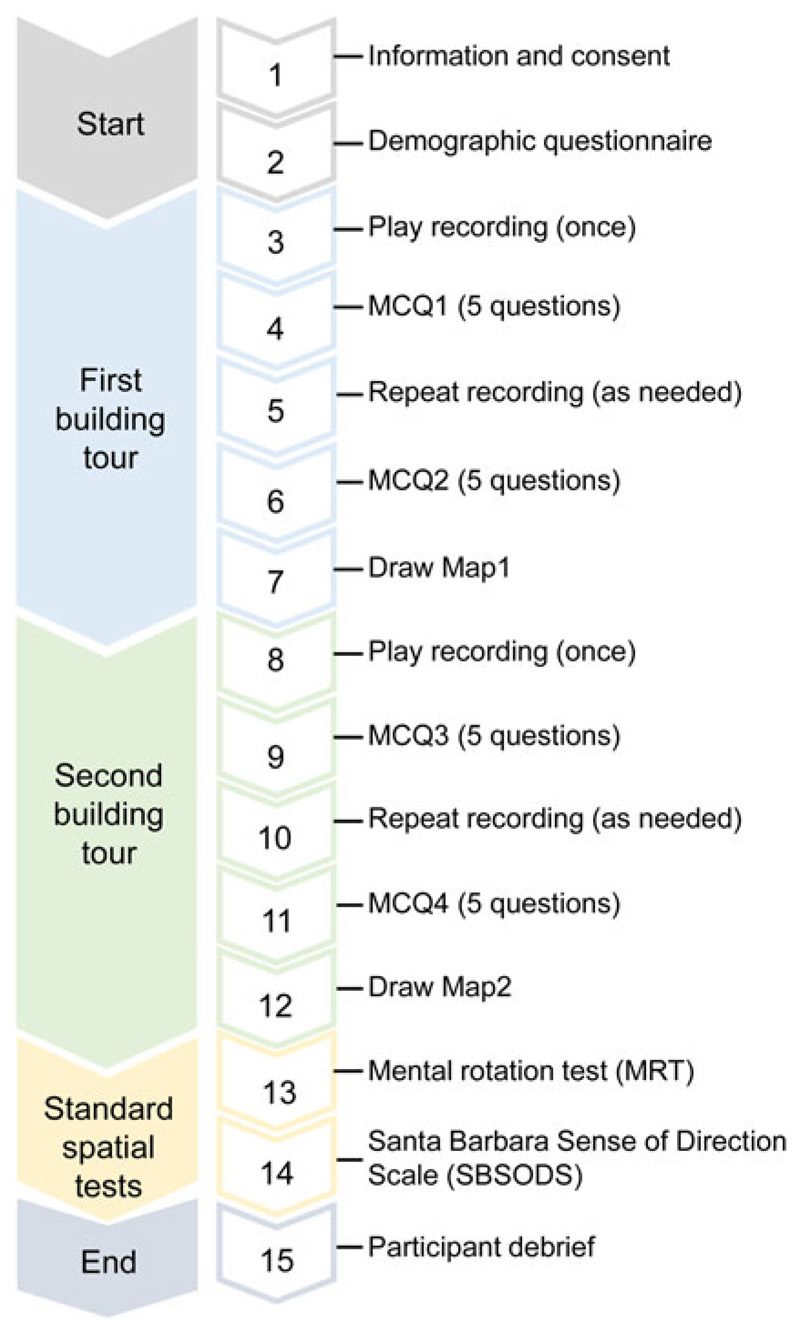
Study protocol.

The procedure for each participant is shown in Figure 3. Participants were first given instructions and asked to sign a consent form and fill in basic demographic information about their age, sex, handedness and first language. They were then presented with the first of the two tours, which varied according to the conditions to which they were assigned (See Table 1). All three types of presentation (video, spoken guidance or written text) were shown or described from a first-person point of view. For the videos, the three recordings were presented as mp4 files. Both the Rot. video and Mirr. video lasted 4·30 min. For the spoken presentations, the Rot. presentations were 6·22 min and the Mirr. presentations were 5·05 min. Each tour consisted of two run-throughs. Participants were first asked to play the recording from the start to the end without stopping, after which they were asked five check multiple choice questions (MCQs) about their understanding of the building layout, for example, 'How many doors are there in the yellow room?'. They then had the opportunity to replay the recording until they felt they understood the layout - this was to control for differences in learning rate and make sure everyone had as much time as they needed. Once they were as confident as they felt they could be in their understanding they answered a second set of five MCQs. Finally, they were asked to draw a map of the building as seen from above (survey view) using a pen/pencil on paper. The entire procedure was then repeated using the other building layout and one of the other two presentation formats.

At the end of the experiment we administered two standard tests of spatial processing, one relating to navigation and one to mental rotation of objects. For navigation we used the Santa Barbara Sense of Direction Scale (SBSODS) questionnaire (Hegarty et al., 2002) which has a total of 15 Likert-type items: participants were required to rate the extent to which they agreed with each statement on a scale of 1 to 7. For mental rotation of objects we used the Vandenberg mental rotation test (MRT; Vandenberg and Kuse, 1978) which is a widely used measurement of small-scale spatial abilities. During this task, participants viewed a three-dimensional target figure and four choice figures, and were required to determine, as quickly and as accurately as possible, which two out of the four choice figures were rotations of the test figure. The task consisted of a set of 10 items, and participants were given a time limit of 3 min for each set. (Participants also completed a second set of 20 but due to a timing error these results were discounted).

At the end of the experiment the maps were scanned by the experimenter and uploaded for later analysis.

#### 2.4.1 Analysis

The quality of mental mapping was assessed both by the maps drawn at the end of each tour and by the MCQs. To score the maps, we rotated them so that the entrance to the building was always at the bottom, and scaled them for convenience to an approximate constant size and aspect ratio (since we were not assessing scale). The maps were then manually classified as either error-free or with minimal errors (e.g., a missing door or window) or structurally incorrect (scrambled spatial structure). To quantify this classification we assigned a score of 5 for a perfect map, 4 for one with minor errors and 1 for a scrambled map. For chi-square analyses we combined the correct and mostly-correct maps to make two categories, 'accurate' and 'inaccurate'. To analyse the MCQs we added the scores for the two sets from each tour and expressed the results as the number correct.

The SBSODS questionnaire was scored by taking the reverse score of the positively phrased items and then summing the scores of all the items, before dividing the total by the number of items (*n* = 15) to compute the overall score (Hegarty et al., 2002). The MRT had a maximum score of 20. Pairs of answers that were both correct were assigned a score of 2, 1 correct and 1 incorrect answer were awarded a score of 0, and a single correct answer was given a score of 1.

## 3 Results

Materials pertaining to this study, including the video tours, audio tours, reading script, questionnaires, maps and tabulated raw data, are available for upload on https://doi.org/10.6084/m9.figshare.13633892. Eighty people participated overall: 51 female and 29 male. Most were native English speakers (*n* = 67), the rest were distributed between Chinese (*n* = 9), Italian (*n* = 2) and Romanian (*n* = 1). All were fluent in English.

### 3.1 Cognitive mapping assessment

**Figure 4:**
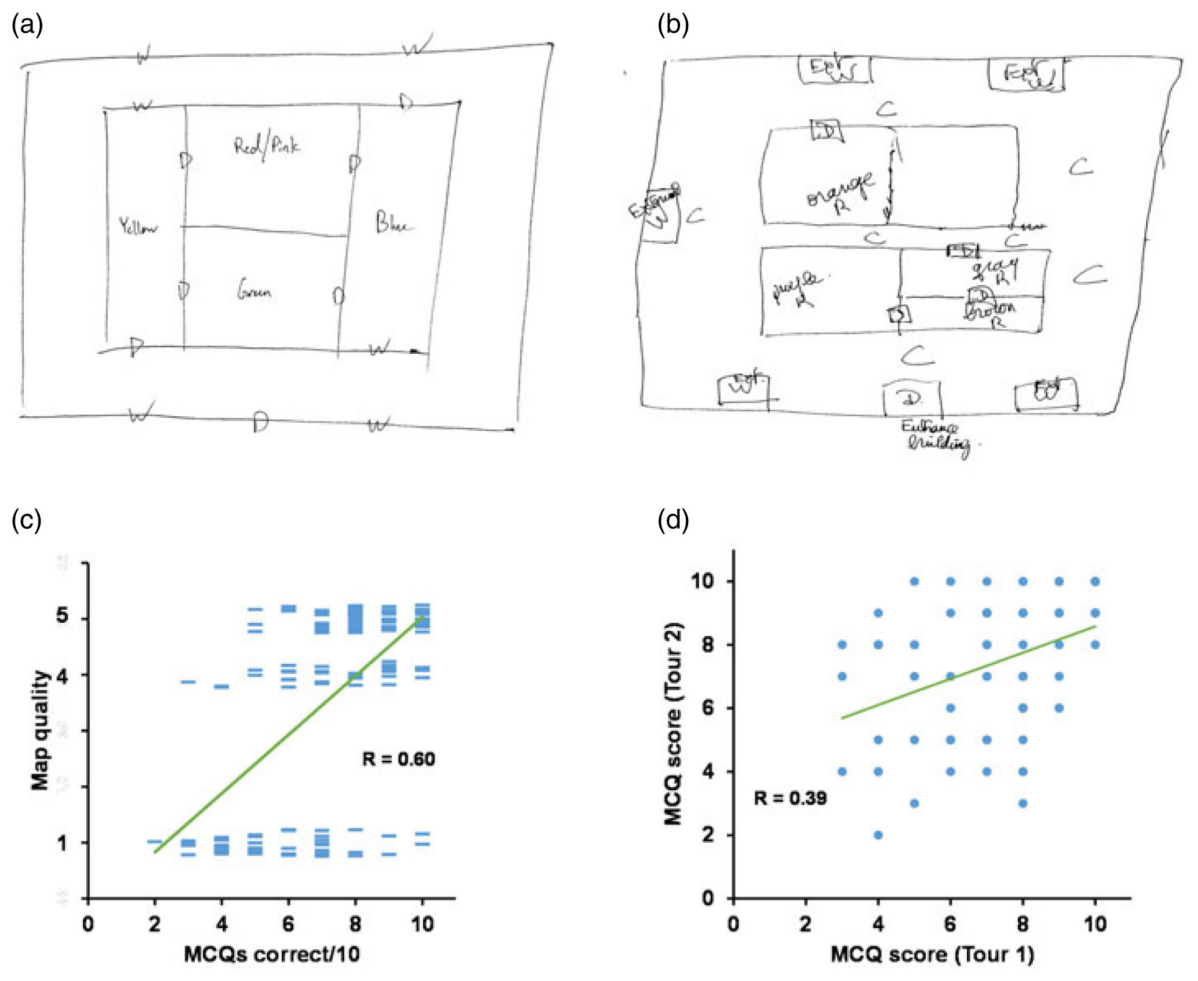
Examples of maps (a) of the rotationally symmetric building that gained a ‘perfect’ score and (b) of the mirror-symmetric building that gained a ‘scrambled’ score. (c) Correlation between MCQ performance and map quality (1 = scrambled; 4 = minor errors; 5 = perfect; each participant contributed two tours). (d) Correlation between MCQ scores for the first and second tours.

Two participants declined to supply a map after the second tour and so only 78 maps (instead of 80) enter into these analyses. We observed that the maps drawn by participants appeared to be distributed in a binary fashion between perfect or near-perfect, or structurally disordered/scrambled (Figure 4(a) and 4(b)). Maps were thus first given a categorical assessment of perfect, minimal errors or scrambled, which for correlation purposes was converted to a numeric score in which we assigned 5 points for a perfect map, 4 for one with minor errors (missing doors etc.) and 1 for a scrambled map. We compared the map scores with the overall MCQ score to check that they correlated, as would be expected if they both assess mental mapping; there was indeed a high correlation (*R* = 0·65, *p <* 0·00001; Figure 4(c)). We then divided the maps into accurate (no or minimal errors) and inaccurate (many errors) to compare performance between the sexes. Females and males did not differ either in map quality (Female accurate maps = 69%; Male = 74%; X^2^(1, *N* = 158) = 0·34, *p* = 0·56) or in MCQ performance (Females = 7·32 ± 0·24, males = 7·19 ± 0·32;t(78) = 0·34, *p* = 0·74). Henceforth we combined the sex data (except in interaction analyses) and used both map quality and MCQ measures to assess mental mapping.

### 3.2 Practice effects occurred within but not between tours

We next looked to see if there were practice effects, bearing in mind that the order of presentation was confounded by symmetry type (the mirror condition was always second). MCQ scores were found to be slightly better after the second run-through of each tour than the first (scores averaged across the two maps: first run-through mean ± s.e.m = 3·44 ± 0·10; second run-through 3·84 ± 0·12; paired t(79) = 3·70, *p* = 0·0004; Cohen's *d* = 0·41). When the first building tour and second were compared, no difference was found, either in the map quality (accurate maps Tour 1 = 56/80, Tour 2 = 56/78; *X* ^2^(1, *N* = 158) = 0-06, *p* = 0·94) or in the MCQ scores (mean ± s.e.m. Tour 1 = 7·15 ± 0·22, Tour 2 = 7·40 ± 0·23; paired t(79) = 0·99, *p* = 0·32). However, people who scored well in the MCQs in one were more likely to score well in the other (R = 0·39, *p* = 0·004).

### 3.3 Cognitive mapping performance was higher for video than verbal guidance

**Figure 5:**
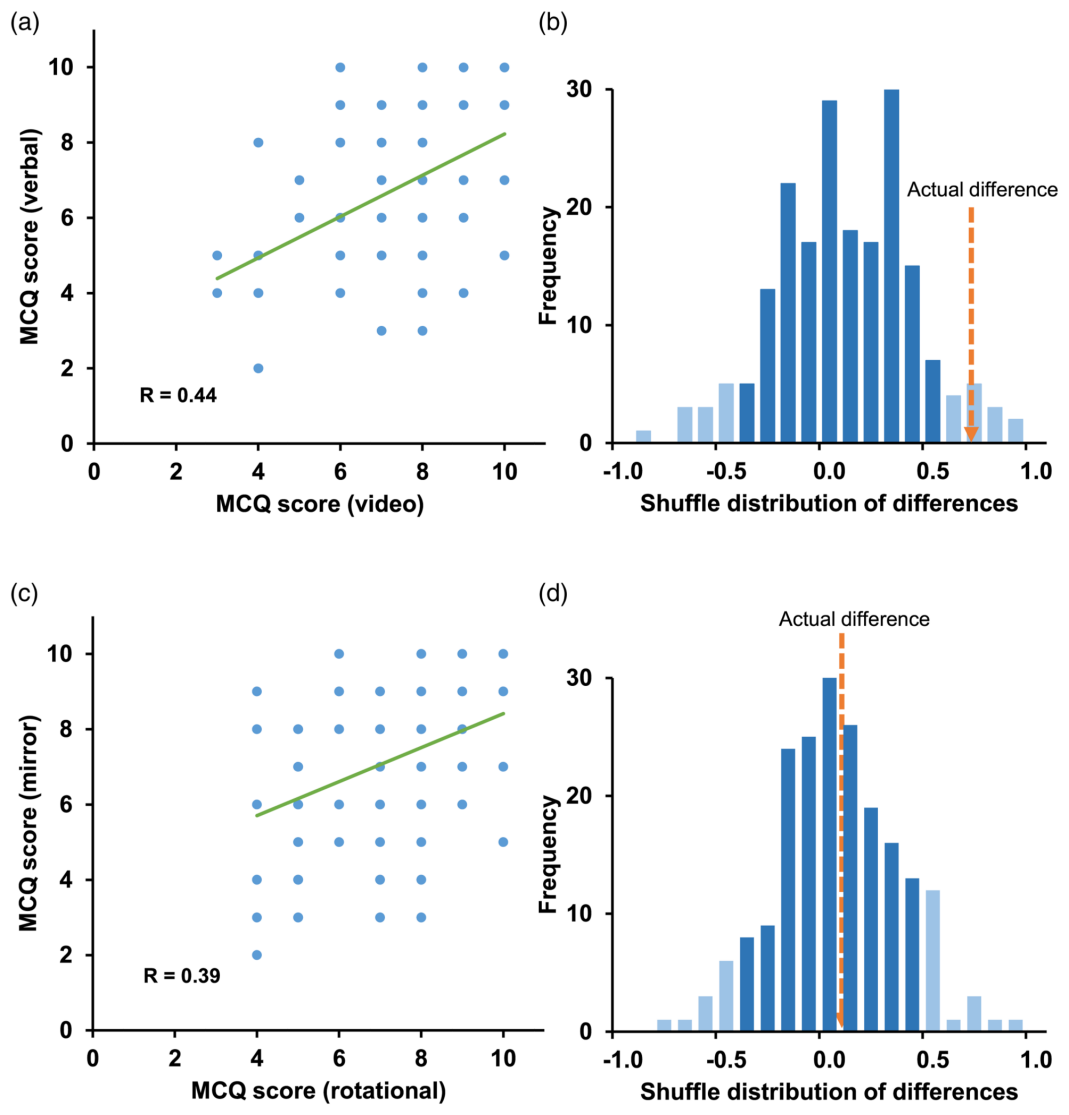
Comparison of cognitive mapping scores following video versus verbal guidance. (a) Each data point shows the MCQ score plotted following verbal guidance against that following video guidance. (b) Visualisation of the video versus verbal mapping score differences. If mapping is equal the values should lie around zero, as shown by the null distribution (actual data randomly shuffled and resampled 200 times; 5% and 95% confidence intervals in light shading). The actual difference, shown by the dotted arrow, lay well outside the 95% confidence intervals. (c) and (d) The same comparison for rotational versus mirror symmetry, showing no effect of building type on mental mapping, with the real difference data lying close to zero and well within the confidence intervals.

We then compared performance for tours that were presented with the video or verbally. Map quality was higher for video than verbal presentation (accurate maps video tour = 67/80, verbal tour = 45/78; *X* ^2^(1, *N* = 158) = 12·99, *φ* = 0·28, *p* < 0.05) and MCQ answers scored higher in the video condition (Figure 5(a); mean ± s.e.m. video tour = 7·65 ± 0·20, verbal tour = 6·90 ± 0·25; paired t(79) = 3·14, *p* = 0·002; Cohen's *d* = 0·36).

The difference between video and verbal scores is illustrated in Figure 5(b) by plotting the actual data against a control set. To generate the control set the combined set of MCQ scores was randomly shuffled, divided in two and re-differenced, this repeated 200 times, to generate a null distribution of differences that the real data were compared against. The figure shows that actual difference between the video and verbal MCQ scores lay outside the confidence intervals of the null distribution. Despite these differences, there was a significant positive correlation between performance in the two settings (*R* = 0·44, *p* = 0·00004). Within the verbal map scores, spoken versus written presentations were compared but there was no difference (accurate maps from the spoken tour = 22/39, written tour = 23/39; *X* ^2^(1, *N* = 78) = 0·05, *p* = 0·82). No interaction was found in sensitivity to presentation type as a function of sex. A chi-square test of accurate versus inaccurate maps was non-significant (*X* ^2^(1, *N* = 112) = 0·04, *p* = 0·84) and a two-way analysis of variance (ANOVA) comparing MCQ scores for the two conditions found no interaction (F(1,156) = 0·09, *p* = 0·77).

### 3.4 Mental mapping performance was not affected by building symmetry

We next compared map accuracy and MCQ performance for tours of the rotationally versus mirror symmetric buildings. Map quality did not differ between building types (Figure 5(c); accurate maps rotational = 58/80, mirror = 54/78; *X* ^2^(1, *N* = 158) = 0·20, *p* = 0·65). Likewise, MCQ scores did not differ (mean ± s.e.m. rotational = 7·34 ± 0·21, mirror = 7·21 ± 0·24; paired t(79) = 0·49, *p* = 0·62; Figure 5(d)). Thus, contrary to our predictions, the presence of rotational symmetry was not detrimental to mapping performance relative to mirror symmetry. As before, there was a positive correlation between the two conditions (*R* = 0·39; *p* = 0·0004). We also looked at sensitivity to building symmetry as a function of sex. A chi-square test of accurate versus inaccurate maps was non-significant (*X* ^2^(1, *N* = 112) = 0·77, *p* = 0·38) and a two-way ANOVA comparing MCQ scores for the two conditions found no interaction (F(1,156) = 0·07, *p* = 0·79).

### 3.5 Interaction effects

We looked at whether there might be interaction between presentation type and building symmetry but found no effect. A chi-square test of accurate maps (perfect plus minor errors) as a function of presentation type versus building symmetry was non-significant (*X* ^2^(1, *N* = 112) = 0·007, *p* = 0·93) and a two-way ANOVA comparing MCQ scores for the two conditions found no interaction (F(1,156) = 0·03, *p* = 0·90).

### 3.6 Correlation of mapping scores with standard spatial processing metrics

**Figure 6:**
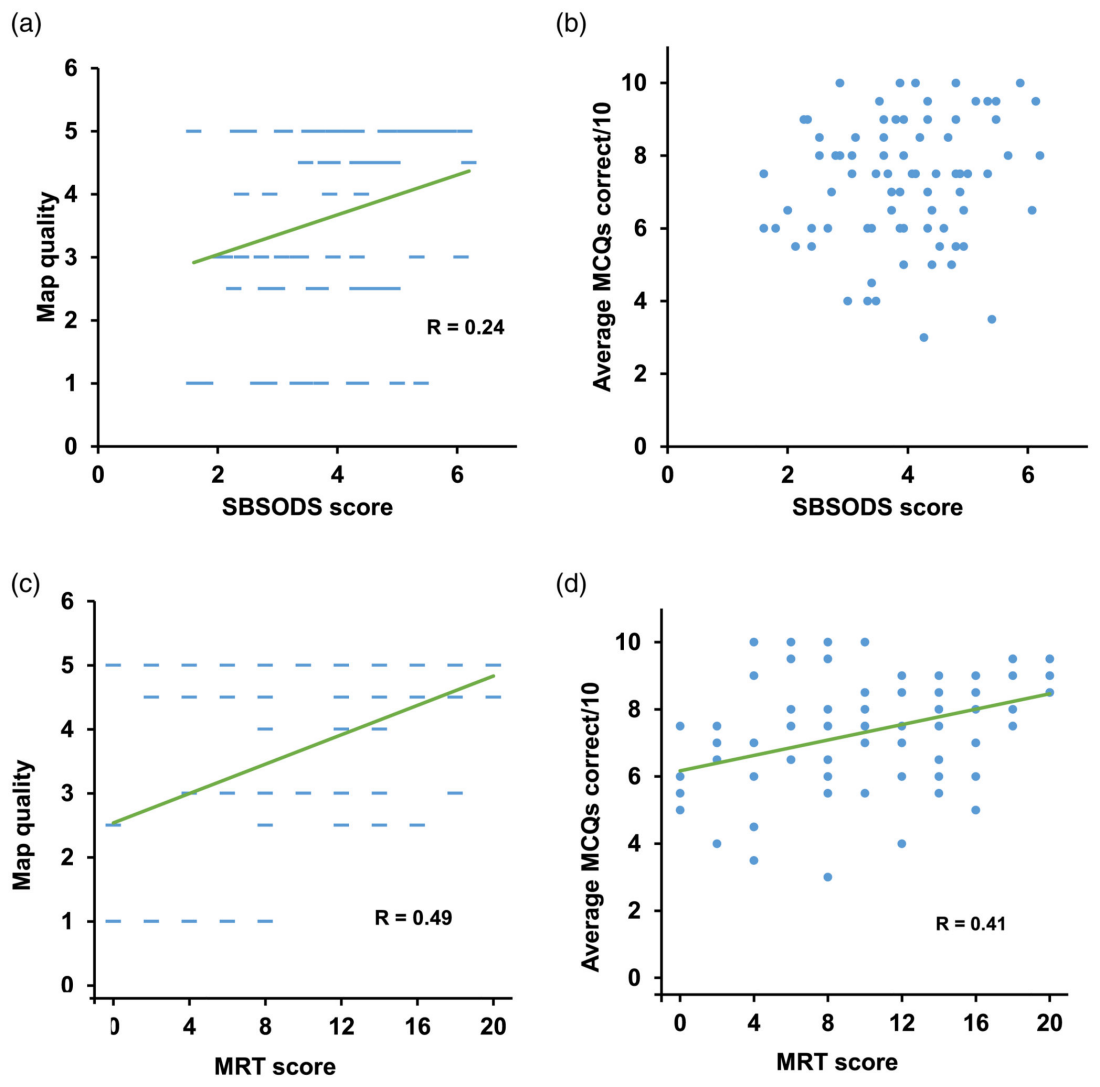
Comparison between (a) score on the Santa Barbara Sense of Direction Scale (SBSODS) and map quality (average of both maps) showing correlation (scores, as before, are 1 = scrambled; 4 = almost perfect, 5 = perfect). (b) SBSODS and MCQ score, showing no correlation. (c) and (d) The same measures for Mental Rotation Test (MRT).

We then looked at how map quality and MCQ score related to performance on a self-report measure of navigation, the Santa Barbara Sense of Direction Scale (SBSODS), and a measure of local egocentric spatial processing, the Mental Rotation Test (MRT; see Methods). There was a very weak correlation of SBSODS score with map quality (*R* = 0·24,*p* = 0·03; Figure 6(a)) and no correlation with the MCQ scores (*R* = 0·15, *p* = 0·18; Figure 6(b)). However there was a correlation of the small-scale object rotation task, the MRT, with both map quality (*R* = 0·49, *p <* 0·00001;Figure 6(c)), and MCQ performance (*R* = 0·41, *p* = 0·0002; Figure 6(d)). We investigated whether this might be due to a greater reliance on imagination and mental rotation when the tour was presented verbally, and so we recalculated the correlations with the video and verbal maps independently, using the same numerical map score as earlier (perfect = 5, minor errors = 4, scrambled = 1). We found a positive correlation of MRT with maps following both types of presentation (video *R* = 0·50, *p* = <0.00001; verbal *R* = 0·37, *p* = 0·0008); there was no difference between these (*p* = 0·16). We looked at whether mapping scores correlated with sex. There was no difference in MRT score (females = 9·29 ± 0·81, males = 10·34 ± 1·24;t(78) = 0·74, *p* = 0·46). There was, however, a significant difference in the SBSODS, with females rating their navigation ability lower (females = 3·61 ± 0·15, males = 4·45 ± 0·17; t(78) = 3·44, *p* = 0·0009; Cohen’s *d* = 0·75).

## 4 Discussion

This study set out to investigate the role of imagination processes in facilitating cognitive mapping. We simulated navigation using two presentation methods, one requiring mental simulation of a tour around a building (the verbal guidance) and the other presenting the journey by video. We also used two building layouts: one having structural rotational symmetry, which adds visual directional ambiguity and thus theoretically loads the internal sense of direction more heavily, and the other having mirror symmetry, in which every room presents a unique visual panorama and is potentially less demanding of memory. We used both map-drawing and questionnaires to assess how well people had formed a cognitive map of the layout: scores from these two methods correlated despite their very different formats (one requiring mental rotation of the imagined viewpoint to an overhead view, and the other maintaining a ground-level perspective) supporting their validity as assessments of mapping. The two main positive findings are that cognitive mapping (1) occurred following both visual and imagined navigation but was better following the video tour, and (2) was correlated with performance in a small-scale mental rotation task and slightly correlated with a self-report navigation measure (which also showed a large sex difference). The negative findings are that performance did not differ (1) between buildings with rotational versus mirror symmetry or (2) between males and females. We discuss these observations in light of the factors known or postulated to support cognitive mapping, in particular the role of visuospatial processing.

### 4.1 Video versus verbal guidance

When people walk physically through a space they receive vestibular, optic flow, proprioceptive and motor cues as to the speed and direction of movement, whereas in verbally guided navigation they have to imagine moving with no sensory inputs. The question addressed here is to what extent they are still able to form an accurate cognitive map of the imaginary environment layout. Our finding, in agreement with earlier studies of verbal guidance (Denis and Zimmer, 1992), was that topographically accurate maps can be formed following such verbal descriptions, indicating that sensory motion cues are not obligatory for cognitive mapping. However, such cues clearly contribute, since maps were better following video guidance. This leads to two questions: (1) how does verbal guidance enable cognitive mapping at all, and (2) what additional information is provided by the video tour?

Verbal inputs could enable cognitive mapping in two ways. One is by updating the head direction system, which is the neural circuit that supports the internal 'sense of direction', used by the hippocampal place cells to orient their activity (Harland et al., 2017) and thus establish a cognitive map (O'Keefe and Dostrovsky, 1971). During active navigation, self-motion cues arising from actively commanded physical rotations update the head direction signal (Shinder and Taube, 2014). This could in principle be achieved without vision; the question is whether it could be achieved verbally. We do not yet know whether the human head direction system is updated during verbally guided navigation although there is some evidence that it does operate in imagined navigation (Horner et al., 2016), as well as in desktop virtual navigation, despite the absence of movement (Shine et al., 2016; Kim et al., 2017). It has been reported that imagining of a verbally instructed space activates primary visual cortex (Lambert et al., 2004) which could potentially provide a surrogate visual input to the head direction system, likely via retrosplenial cortex. An alternative, however, is that verbal input bypasses the head direction system altogether and mediates direct updating of an internal mental model of the space that orients and positions spatial features relative to each other, without reference to global orientation. Thus, a person can perhaps mentally construct an imagined space while their head direction system purely encodes their orientation in the real world. Future experiments can attempt to disentangle these possibilities by means of interference tasks, in which a mental model of a space is manipulated independently of tracked orientation in the real world.

Despite the verbal contribution to mapping, nevertheless a higher number of maps following verbal guidance were structurally incorrect ('scrambled') and people made more errors when quizzed about the layout in the MCQs. This indicates that additional information is provided by the video tour, which enabled better cognitive mapping. Given the two putative processes discussed above – updating of the head direction signal or of an orientation-independent relational map – it is plausible that updating of either type of representation is easier when the movements are directly perceived (via optic flow and changing visual scene). Thus, people with vision available could have processed spatial relationships more easily than those lacking it.

A final possible reason why mapping was poorer in the verbally guided condition is that information connecting spaces is presented sequentially in time, as opposed to in the video condition in which the connectivity can be apprehended in a single glance. The sequentiality of verbal processing requires that information that links spaces is held in working memory (De Beni et al., 2004), which imposes additional cognitive load. Arguing against this is the fact that spoken guidance was not worse than written, even though when reading, people can pace the rate of information input and go back over things if necessary. However, reading is still, ultimately, sequential rather than parallel. It may be that all three factors – internal directional rotation, visual scene rotation and memory – play a part in cognitive map construction, and that the verbal condition taxed all these aspects of cognitive processing. Resolving this will require more understanding of the degree to which the head direction system is engaged and updated during virtual and imagined navigation. This has implications for how navigation instructions are conveyed to real-world navigators.

### 4.2 Correlation with mental rotation

The second positive finding of this study is that mental mapping of the buildings correlated with performance on the Vandenberg and Kuse Mental Rotation Task (Vandenberg and Kuse, 1978), which is a widely used test of small-scale three-dimensional visualisation which involves deciding whether abstract shapes are rotated versions of each other or not. In principle this faculty should not engage the navigation system as it involves local egocentric processing, more typically associated with parietal cortex (Clark et al., 2018), whereas navigation involves the allocentric hippocampal system. However, there is an extensive prior body of work showing correlations between MRT performance and various navigation tasks including a virtual analogue of the Morris water maze task (Driscoll et al., 2005) which is thought to be a relatively pure cognitive mapping task. Blajenkova et al. (2005) found that people who explored a two-floor building and were subsequently able to draw a three-dimensional map of the layout had higher mental rotation scores than people whose maps were only two or even one dimensional, suggesting a role for small-scale visuospatial ability in large-scale cognitive mapping. These people also tended to report using more directional cues. Similarly, Hegarty et al. (2006) found, in a study of 221 participants, that mental rotation correlated more with ability to learn spatial layouts from visual presentation (virtual environment or video walk-through) than from direct experience. Thus, the correlation in our study may be a function of the fact that none of the modes of spatial learning involved directly moving through a space. This difference could relate to the presence versus absence of vestibular cues to changes in direction, or (to a lesser extent) translation. Future experiments comparing active navigation with passive will be needed to investigate this more fully.

Interestingly, we did not see the typical sex difference in either mental rotation or cognitive mapping that has been widely reported previously (Galea and Kimura, 1993; Moffat et al., 1998). This observation is frequent but still unexplained – it does not universally occur, and may have explanations beyond mental rotation capability *per se,* such as spatial anxiety (Alvarez-Vargas et al., 2020), differences in strategy use (Hegarty, 2018), nature of stimulus presentation (Fisher et al., 2018) and practice effects (Meneghetti et al., 2017) as well as spatial experience generally. Our sample was small, however, and males comprised less than half (29/80). At present, it seems that the question of intrinsic sex differences in spatial processing is still open.

### 4.3 Lack of difference between mirror and rotational symmetry

Despite our initial hypothesis that rotational symmetry would be detrimental to mental mapping, we saw no difference in map quality for the two buildings, which was surprising. It could be that because the mirror condition was always presented after the rotational one, there was negative interference from the experience of the first building, but this seems unlikely – indeed it seems more likely that there would have been an additional positive practice effect. Nevertheless, the results should be treated with caution at this stage. Another explanation is that there was a ceiling effect due to the help provided by the coloured walls, which broke the symmetry for both environments. These were introduced because pilot studies suggested the mapping task was difficult with plain walls, but they may have had the effect of making the disambiguation too easy by allowing vision alone to inform the head direction system about ongoing heading direction, provided this system is sensitive to colour as well as shape. Such sensitivity is suggested by animal research showing that another contextual cue, odour, can be used to disambiguate direction (Jacob et al., 2017). A future repeat of this experiment without the coloured walls, as well as with a no-symmetry condition for comparison, will help answer this. Another alternative is that the mirror symmetric building was also slightly more complex due to the L-shaped rooms it also possessed – this seems unlikely as these features should, if anything, have helped with mapping.

An alternative possibility is that the hypothesis is just wrong, and that having visually ambiguous structures in a building is not detrimental to orientation. This may be because in this setting the sense of direction updates constitutively, based on self-motion cues alone, and does not need any visual reinforcement. In other words, perhaps people tend always to know where they are due to having continuously processed their movements, and so they know which of two identical rooms they are in without needing affirmation from visual cues. Additionally, returning to the possibility discussed earlier that imagined navigation may not require the head direction system at all, it could be that people are able to link together, mentally, the sequences of rooms and their spatial relations in such a way that a coherent map forms even though some of the elements of it are visually repeating.

### 4.4 Weak correlation with mapping and a large sex difference for the SBSODS

The SBSODS is commonly used as a means to assess navigation ability, and correlates with other measures of spatial performance (Burte et al., 2018), but because it is a self-report measure, it is vulnerable to confidence effects. In the present study we found a large sex difference in SBSODS scores despite the absence of a difference in mental mapping or mental rotation. This might be because men were more confident in their spatial abilities than women of the same competence. Scores on this test correlated only weakly with map quality and not at all with MCQ performance, despite previous claims that the SBSODS accurately tests real-world navigation ability (Hegarty et al., 2002). However, since our tasks were passive and did not require physical movement through space, the missing correlation could be due to how well people can process vestibular cues to movement. Repeating the present study in a real-world environment could answer this question.

## 5 Conclusions

Our study confirms that accurate mental maps can be created following verbal guidance. However, the pattern of results is consistent with the notion that mental mapping was optimised by the quality of visual information available to people as they either learned the environment or mentally modelled it for the later retrieval and map generation. On the learning side, support comes from the fact that the participants' maps were better following a video tour than a verbal tour. On the mental model side, maps were better in those with better mental rotation ability, even for the verbal tours. Our results thus add to the growing literature suggesting a role for visuospatial processing in human cognitive mapping, during both learning and retrieval.

## Supplementary Material

The supplementary material for this paper can be found at https://doi.org/10.1017/S0373463321000588.

1

2

3

4

5

## Figures and Tables

**Table 1:** Study conditions.

Condition	*n*	Tour	Presentation	Building symmetry
1	20	1st	Video	Rotational
		2nd	Spoken	Mirror
2	20	1st	Spoken	Rotational
		2nd	Video	Mirror
3	20	1st	Video	Rotational
		2nd	Written	Mirror
4	20	1st	Written	Rotational
		2nd	Video	Mirror
