# Supplementary material for: Visual imagination and cognitive mapping of a virtual building
Source: J Navig. Author manuscript; Available in PMC 2022 Apr 12. (PMC7612610; doi:10.1017/S0373463321000588)

01 Map 1 Mirror Spoken

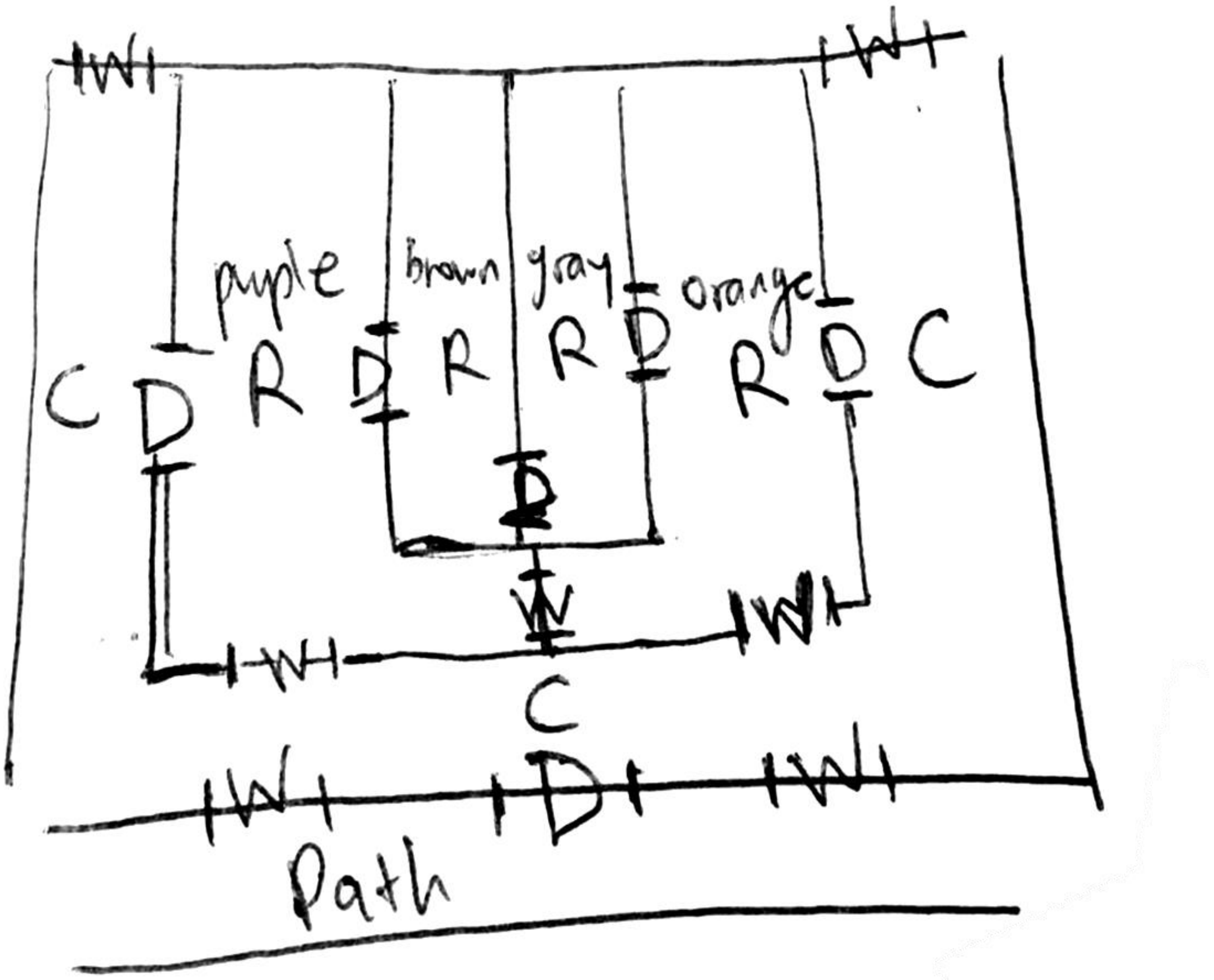

01 Map 2 Rotational Video

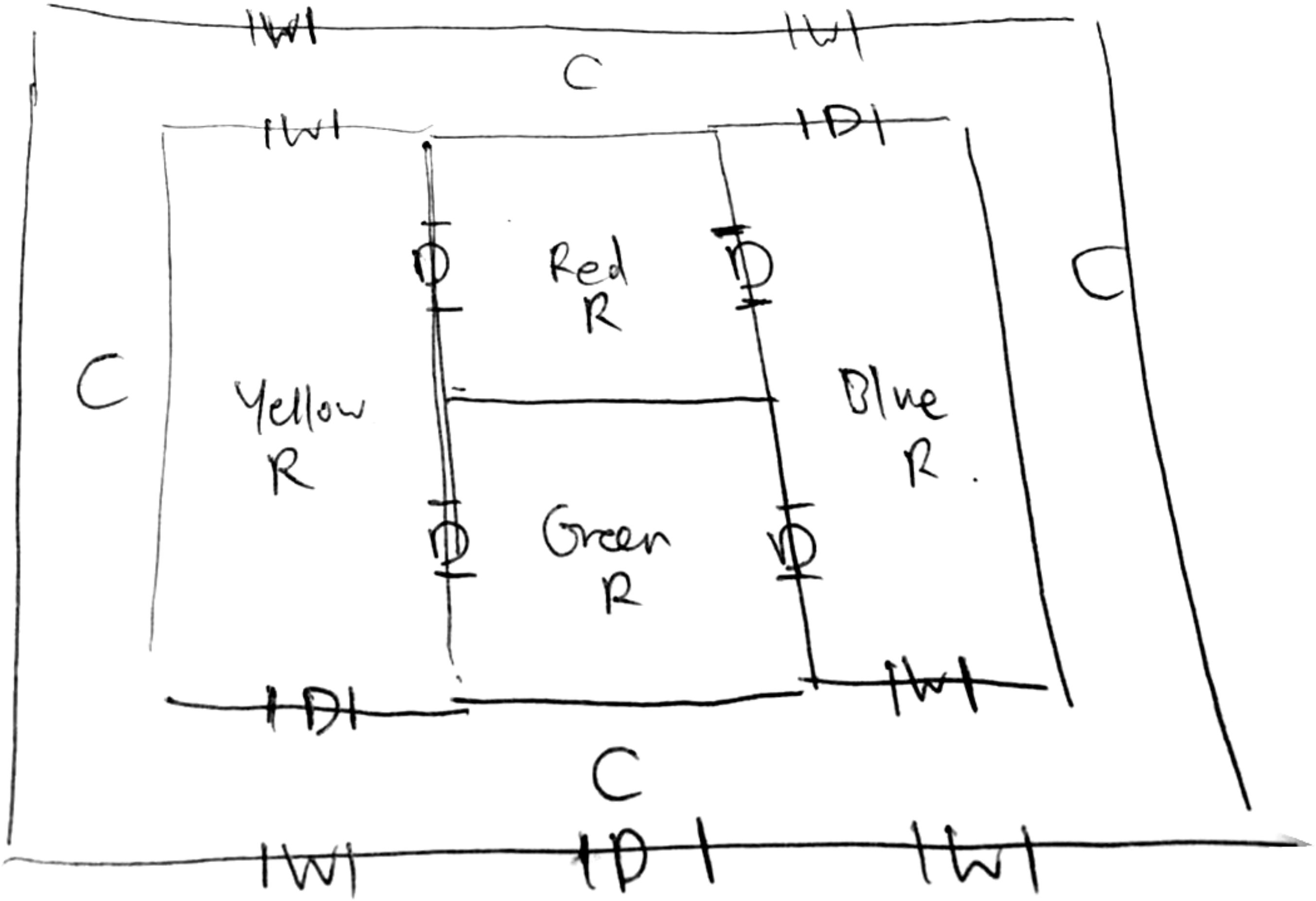

02 Map 1 Mirror Spoken

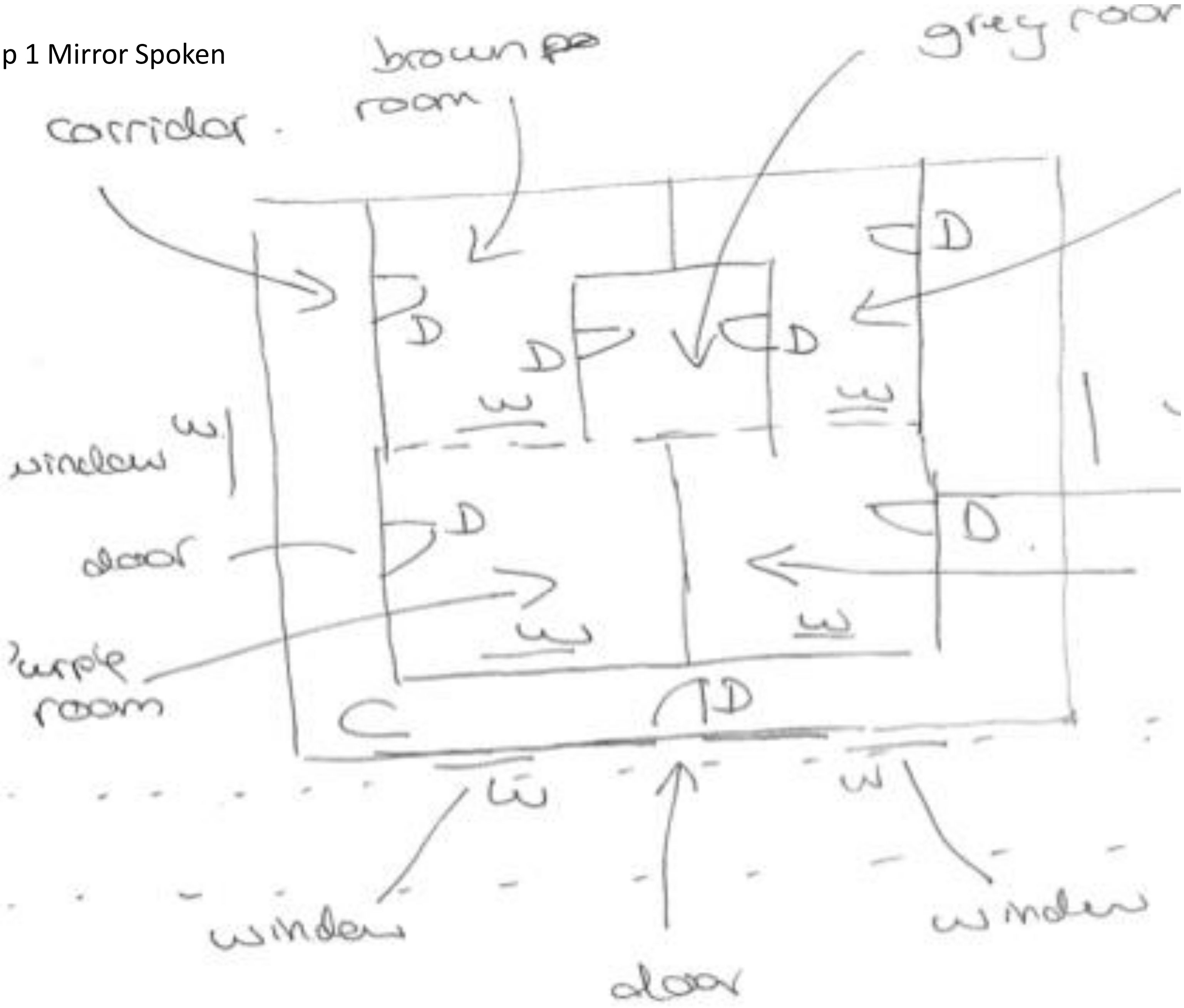

02 Map 2 Rotational Video

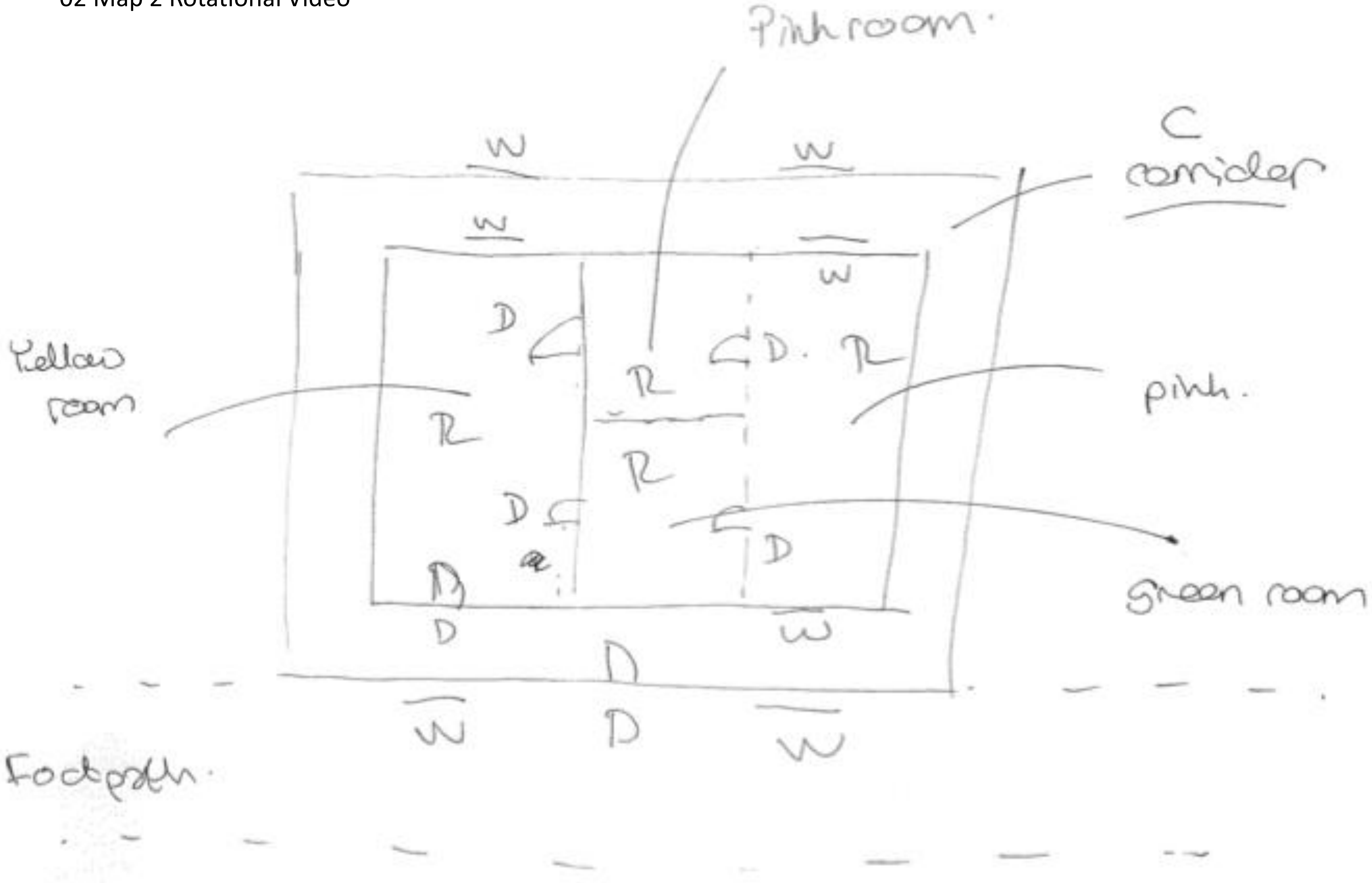

03 Map 1 Rotational Video

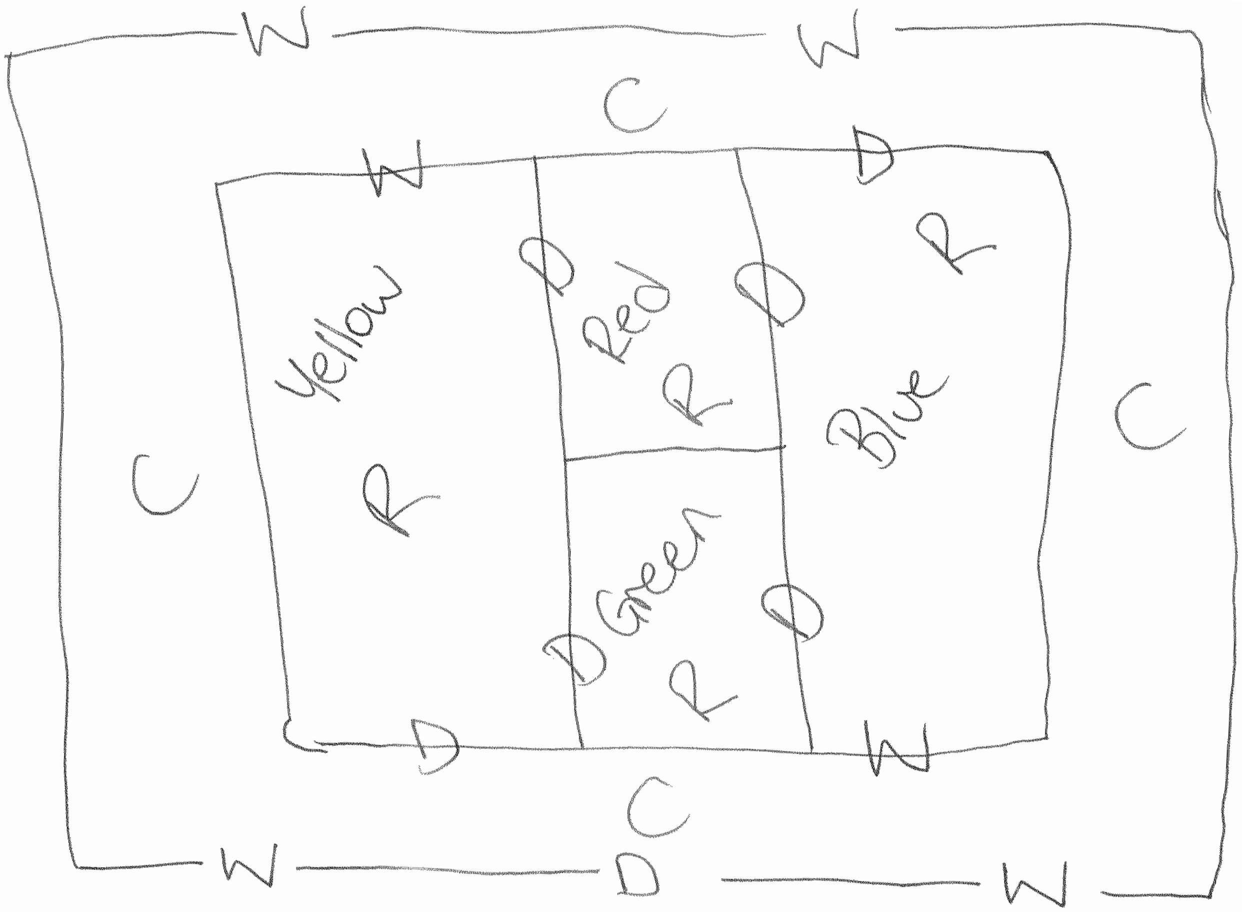

03 Map 2 Mirror Spoken

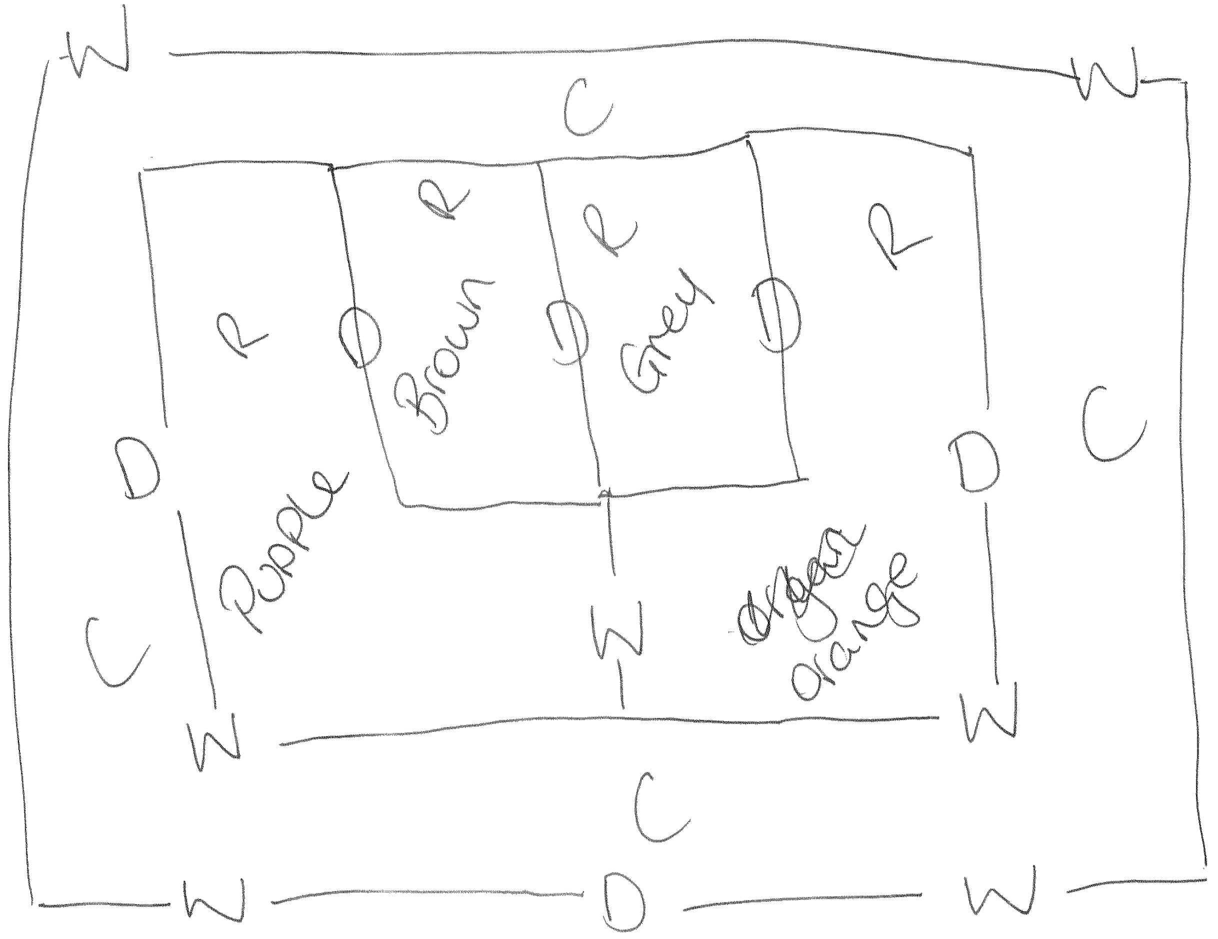

04 Map 1 Mirror Spoken

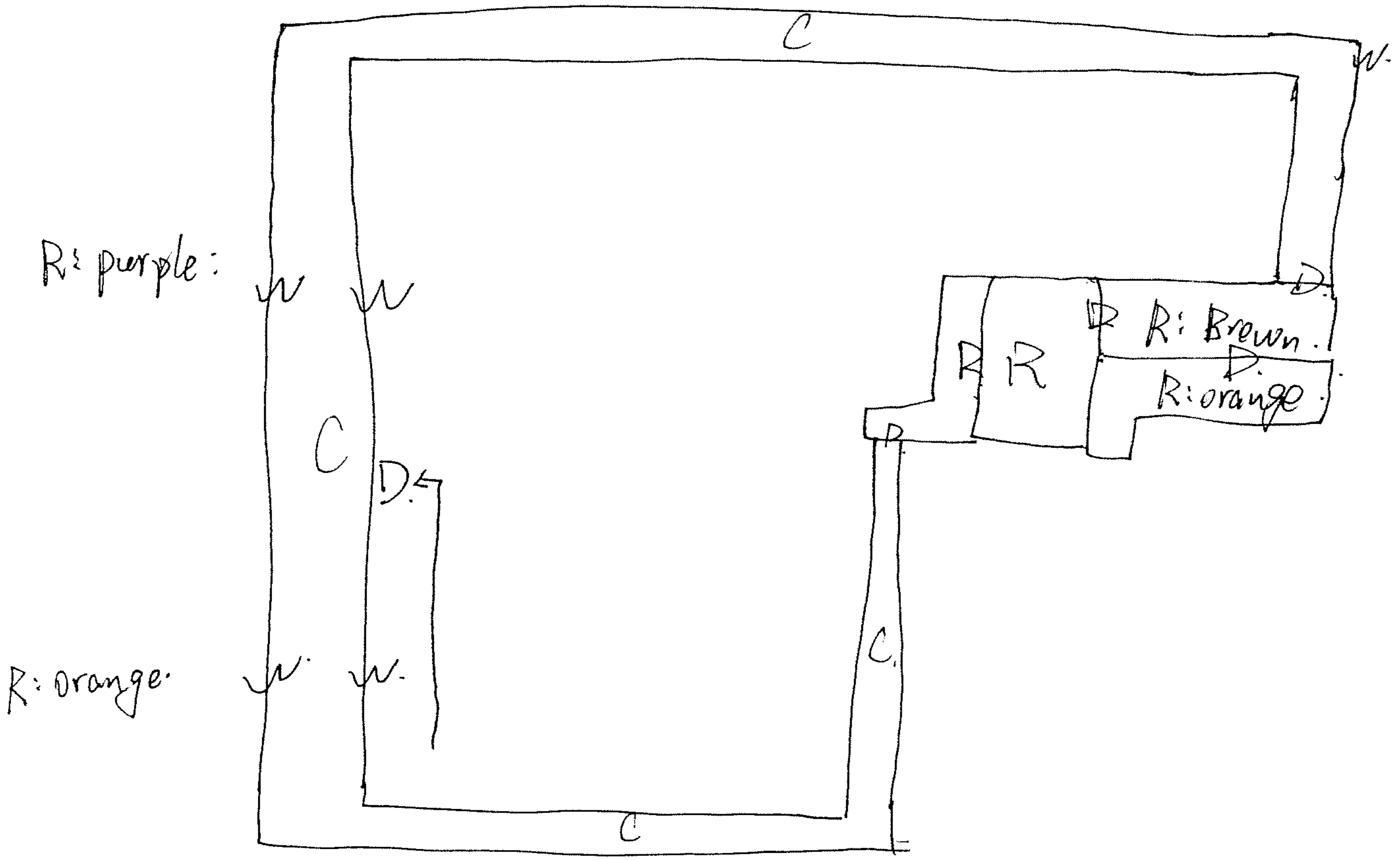

04 Map 2 Rotational Video

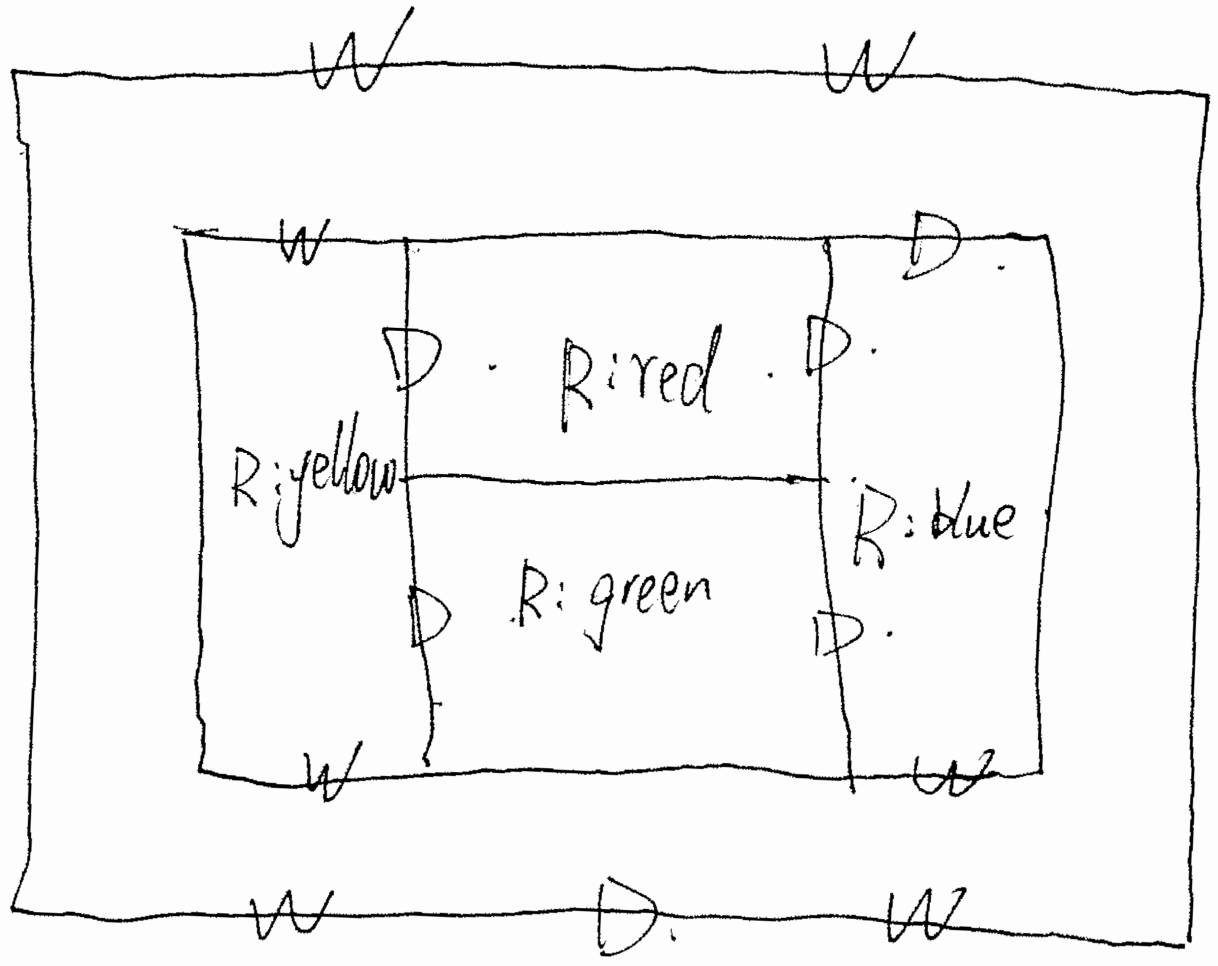

05 Map 1 Rotational Video

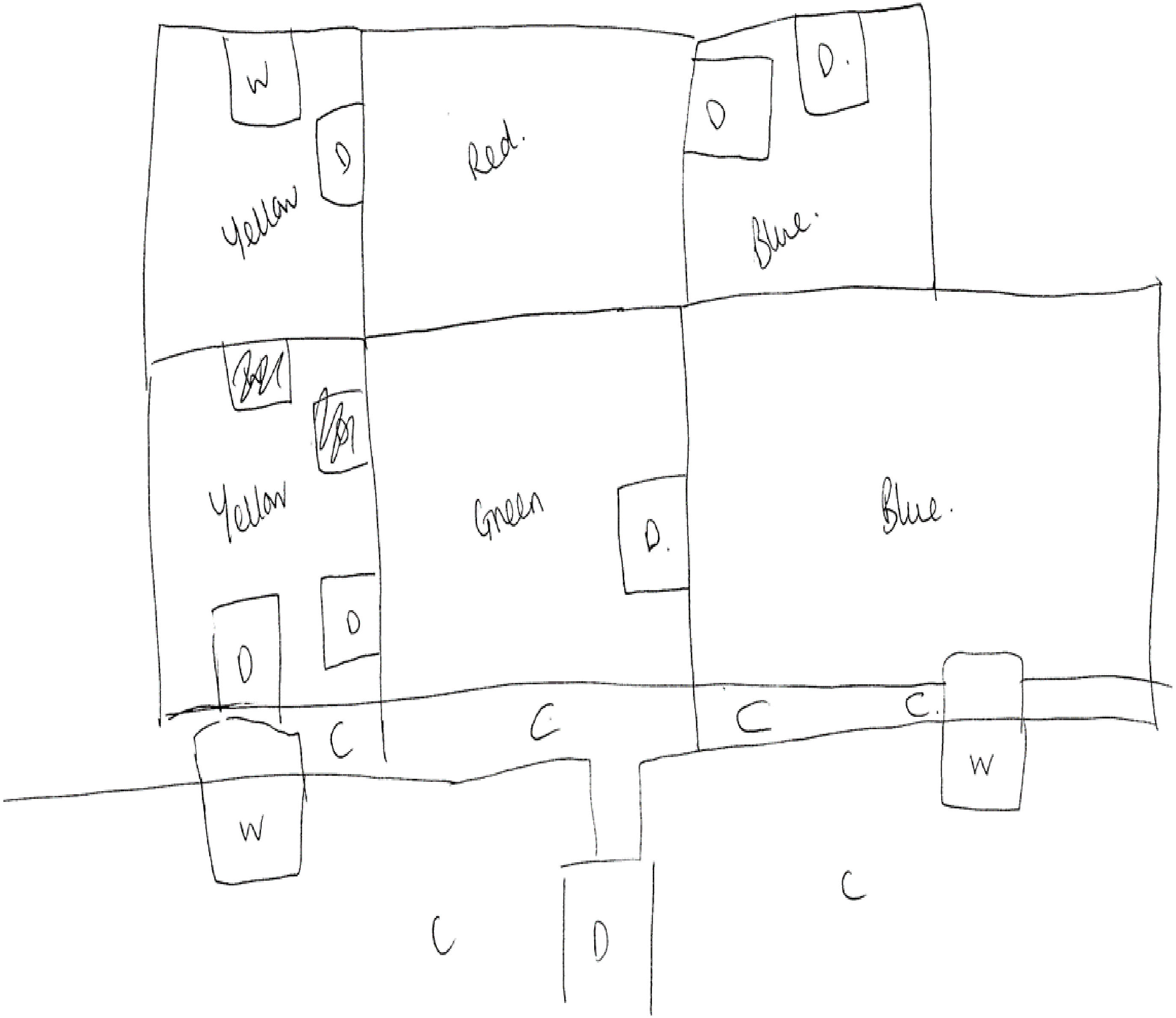

05 Map 2 Mirror Spoken

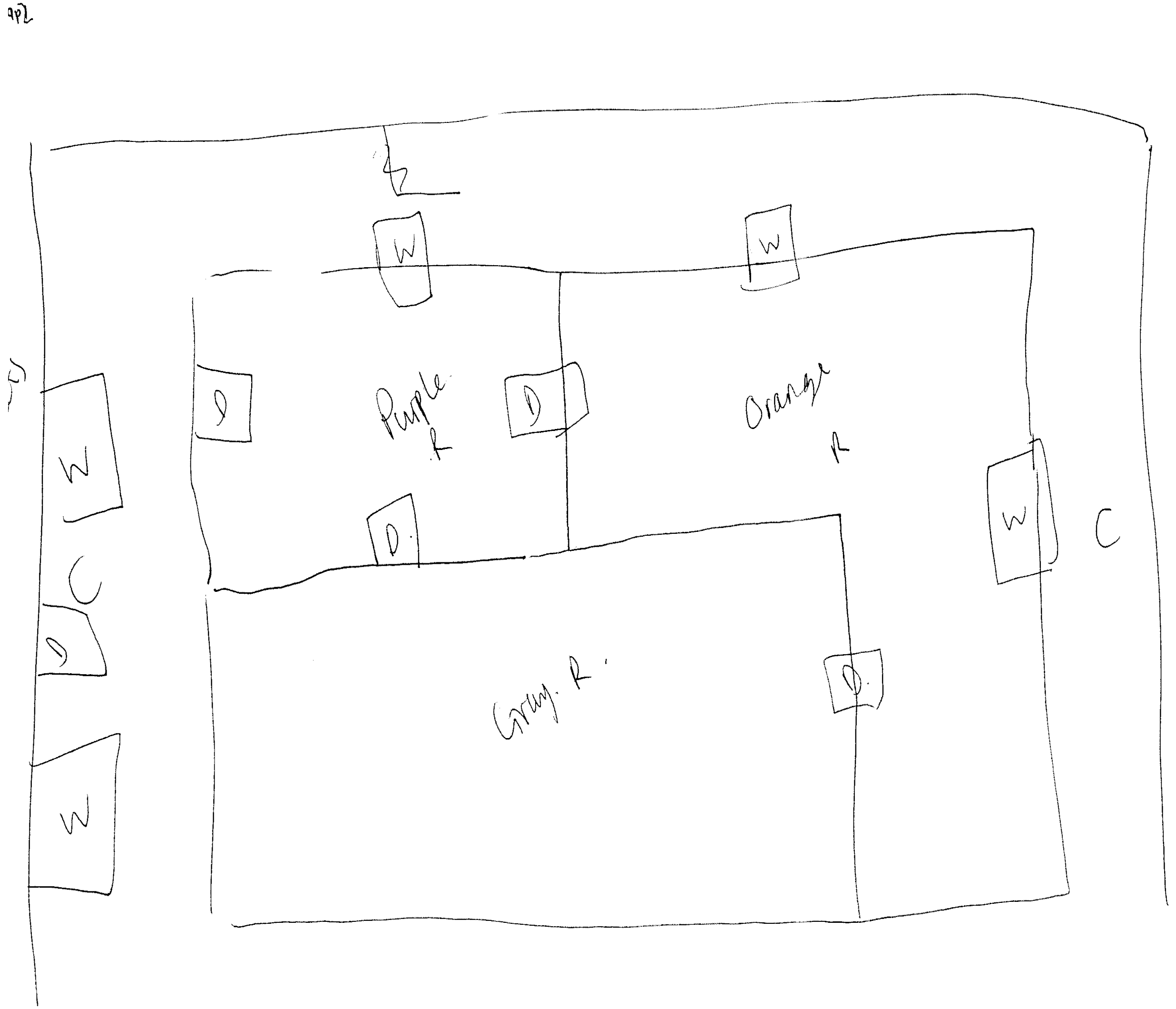

06 Map 1 Mirror Spoken

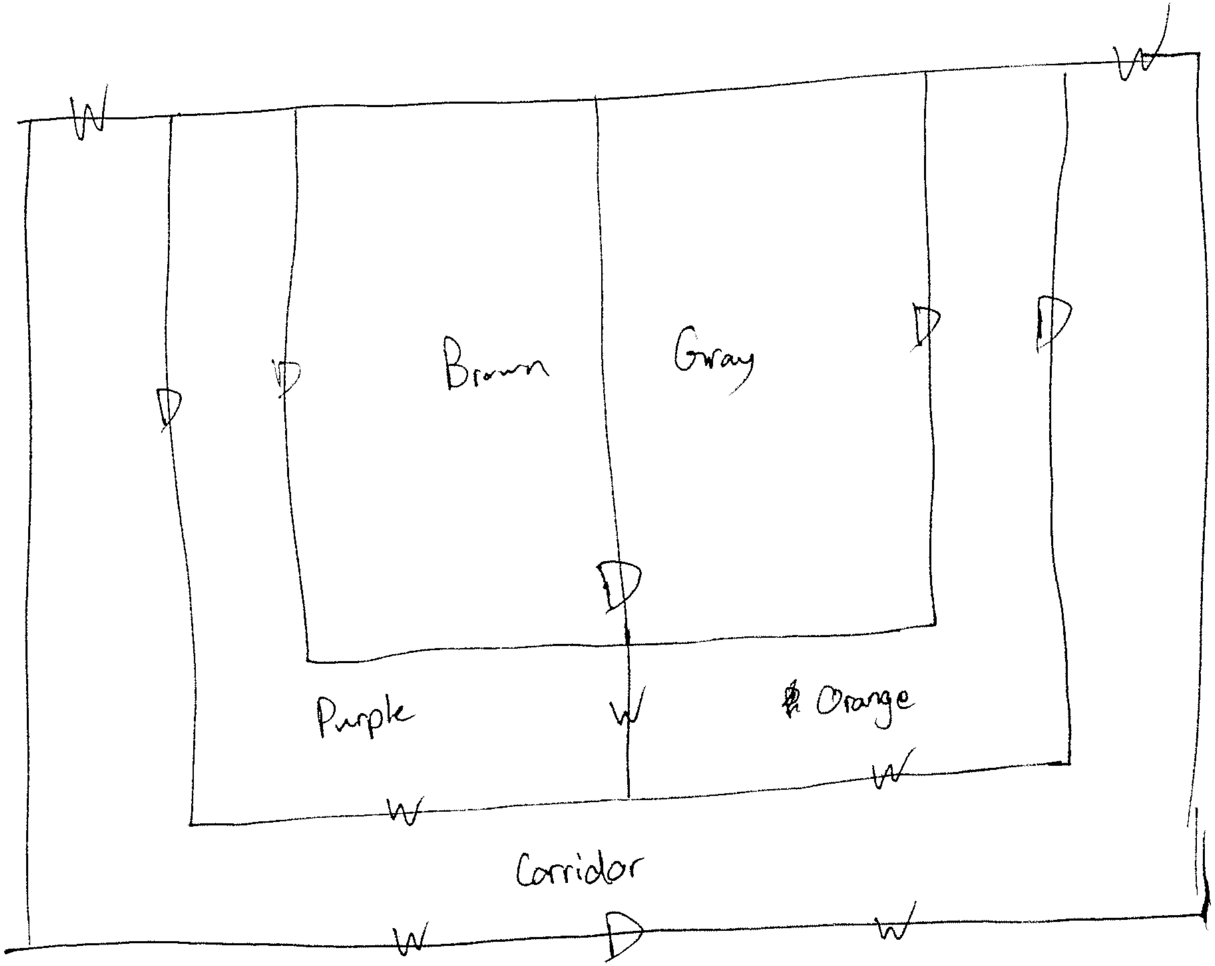

06 Map 2 Rotational Video

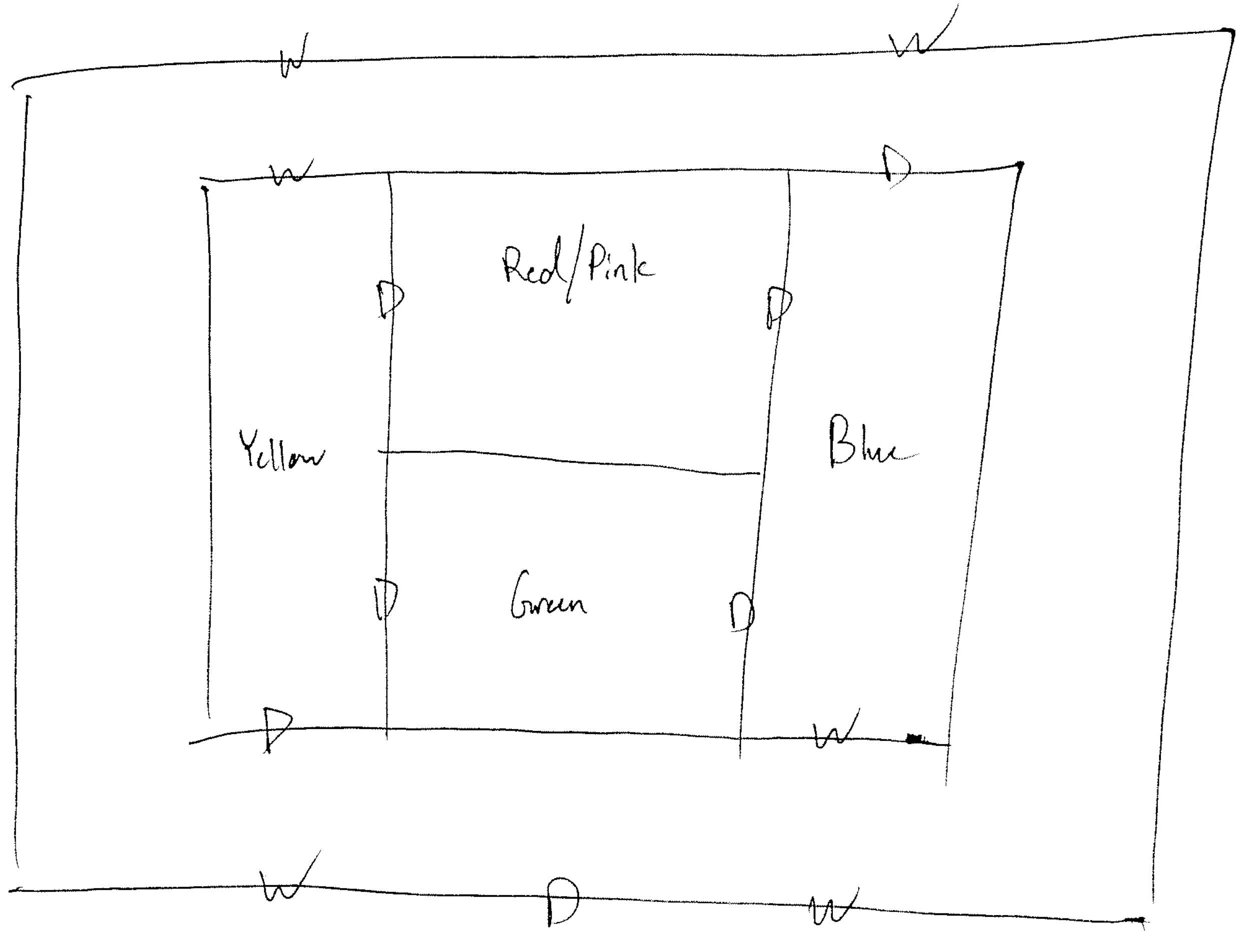

07 Map 1 Mirror Spoken

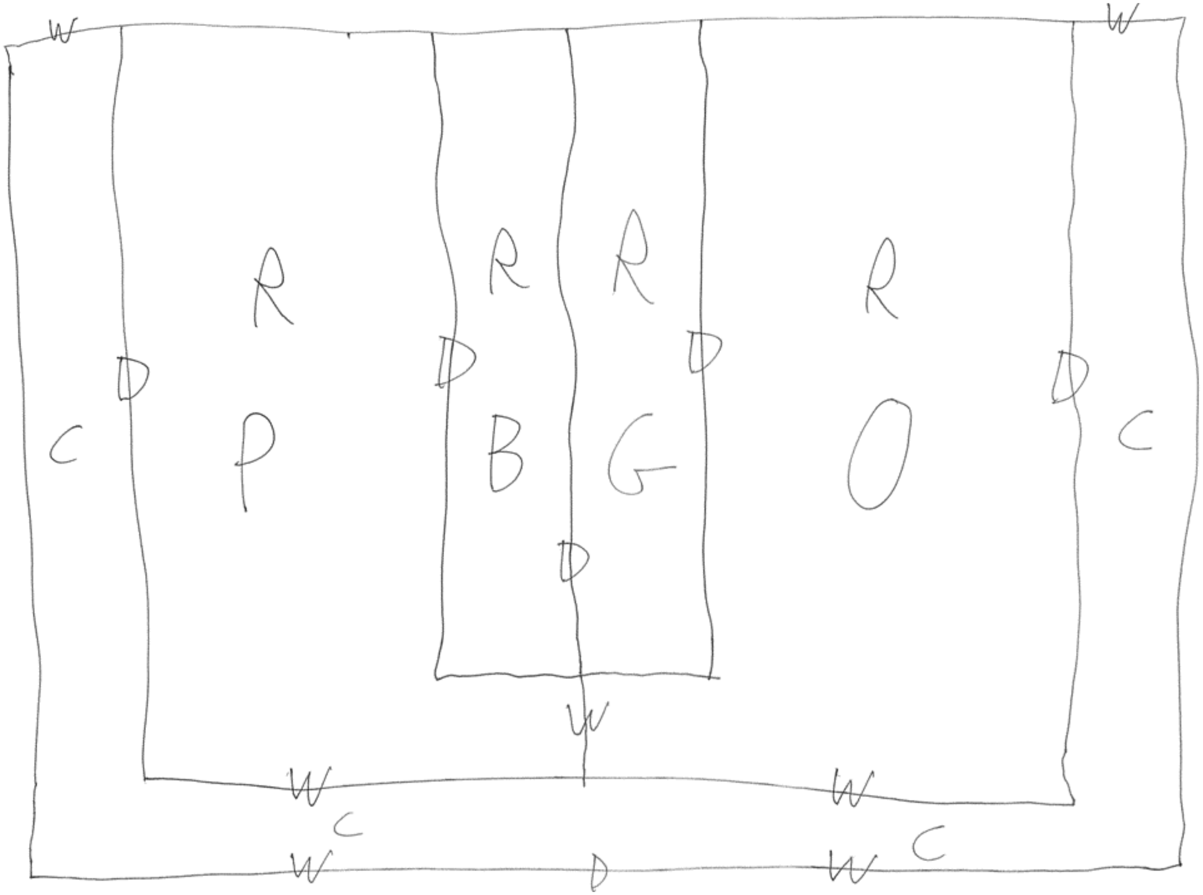

07 Map 2 Rotational Video

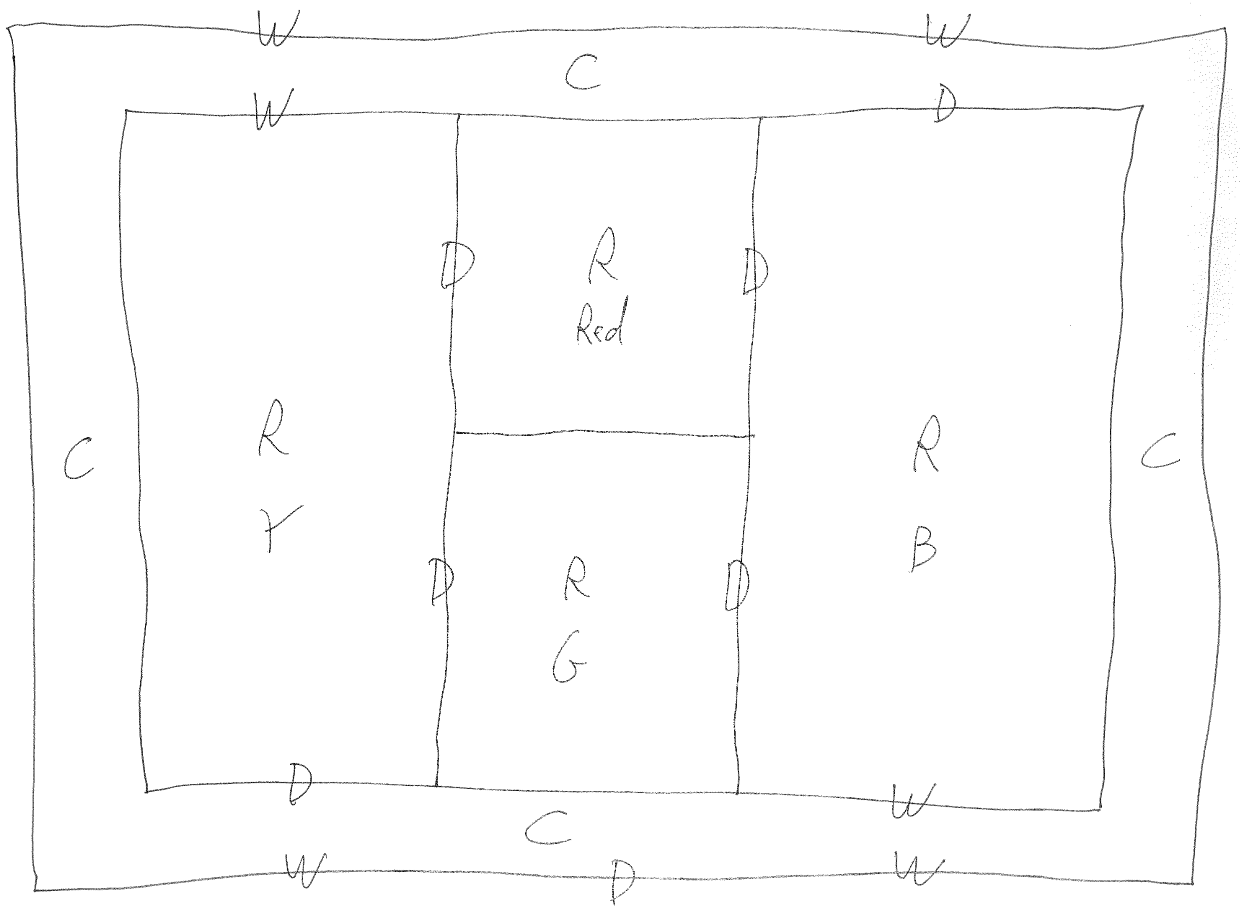

08 Map 1 Rotational Video

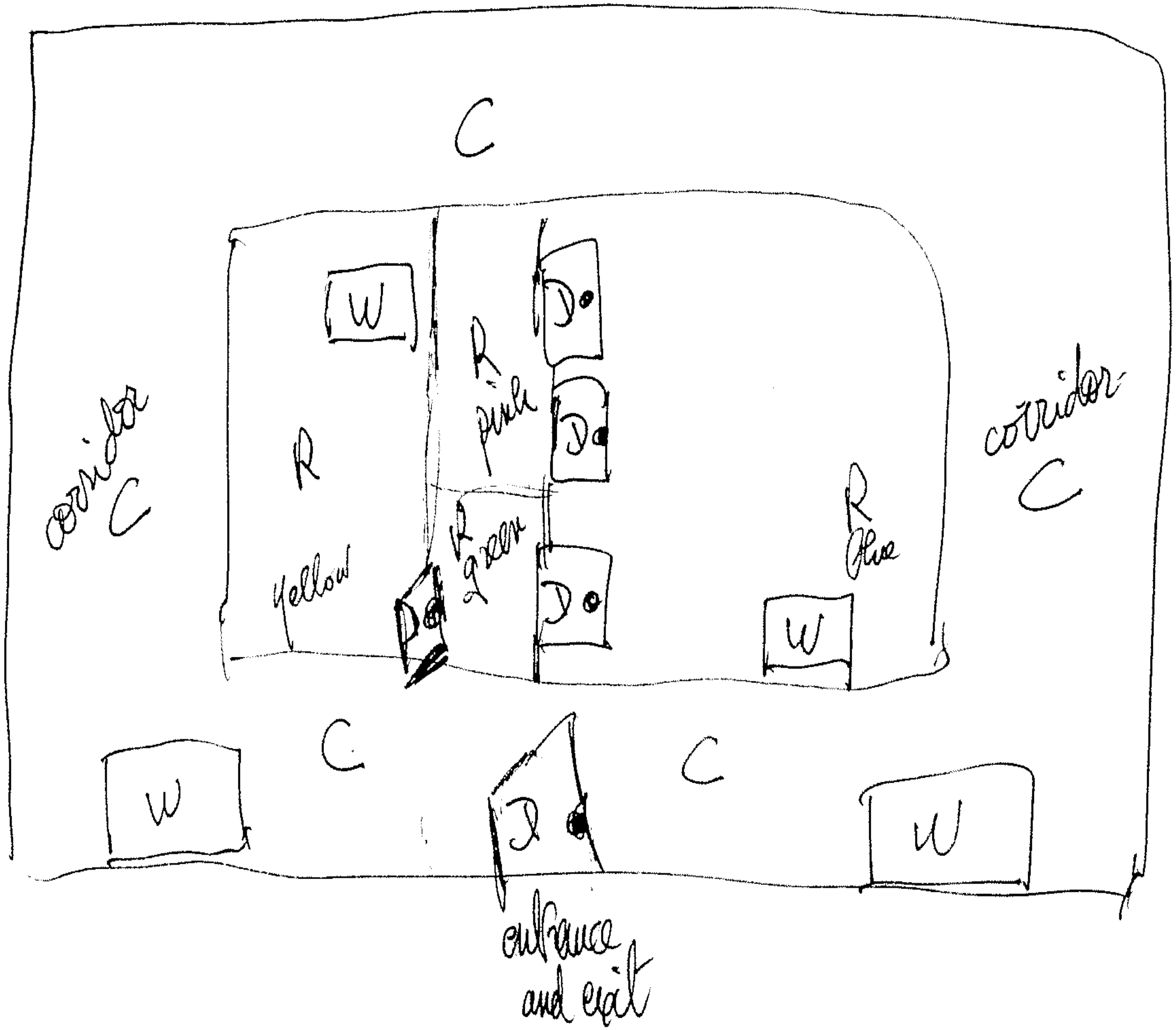

08 Map 2 Mirror Spoken

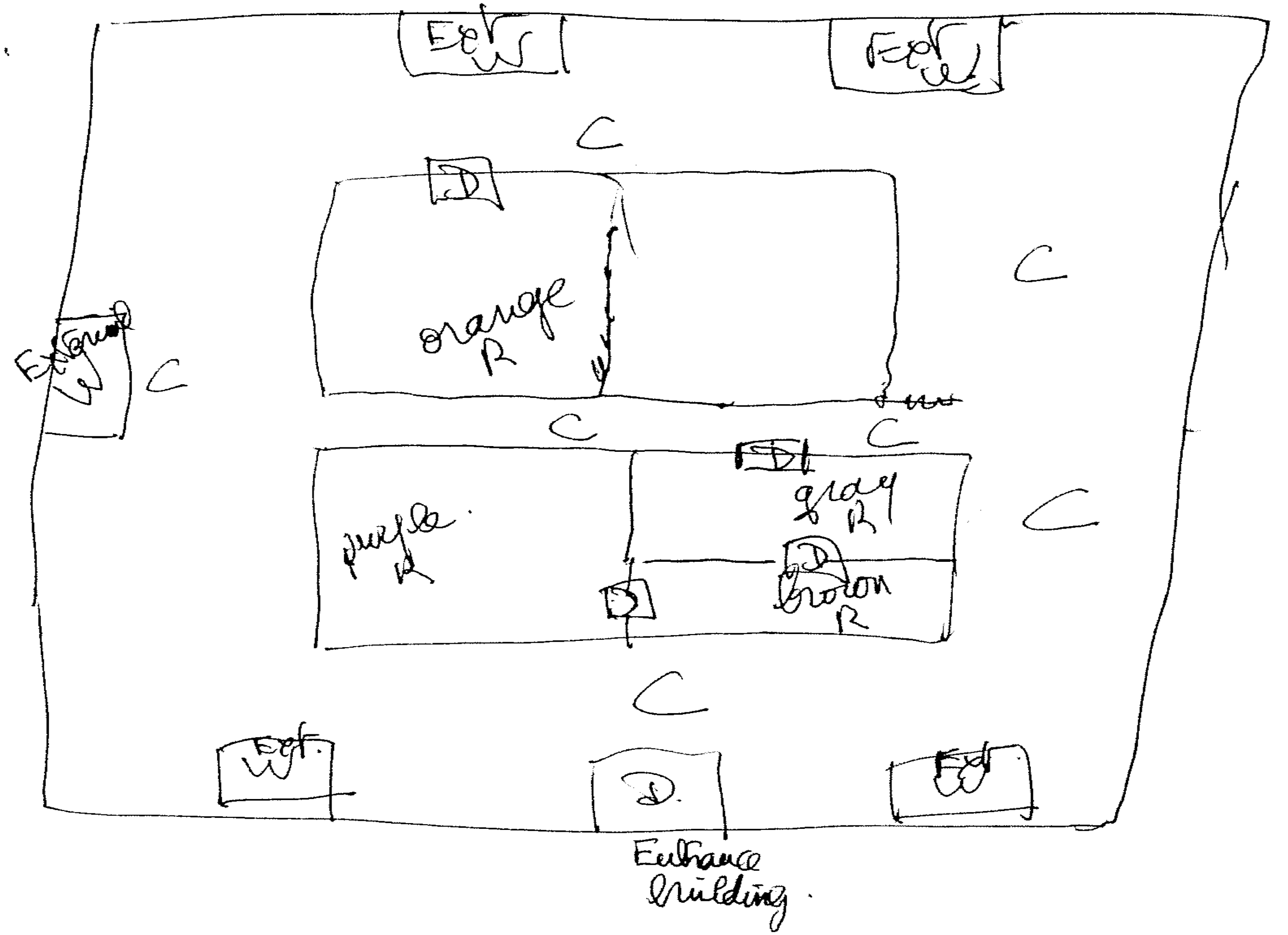

09 Map 1 Mirror Video

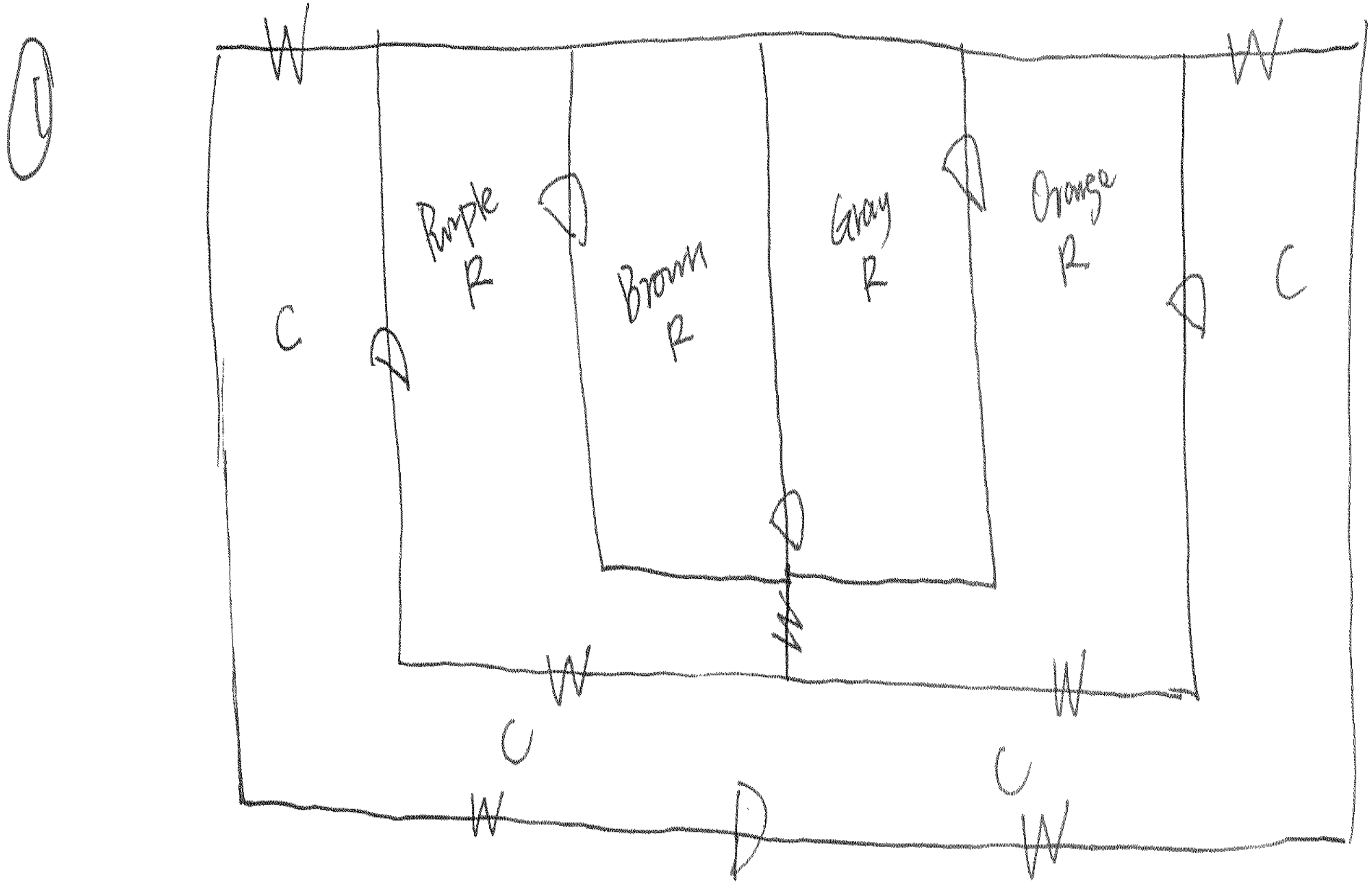

09 Map 2 Rotational Spoken

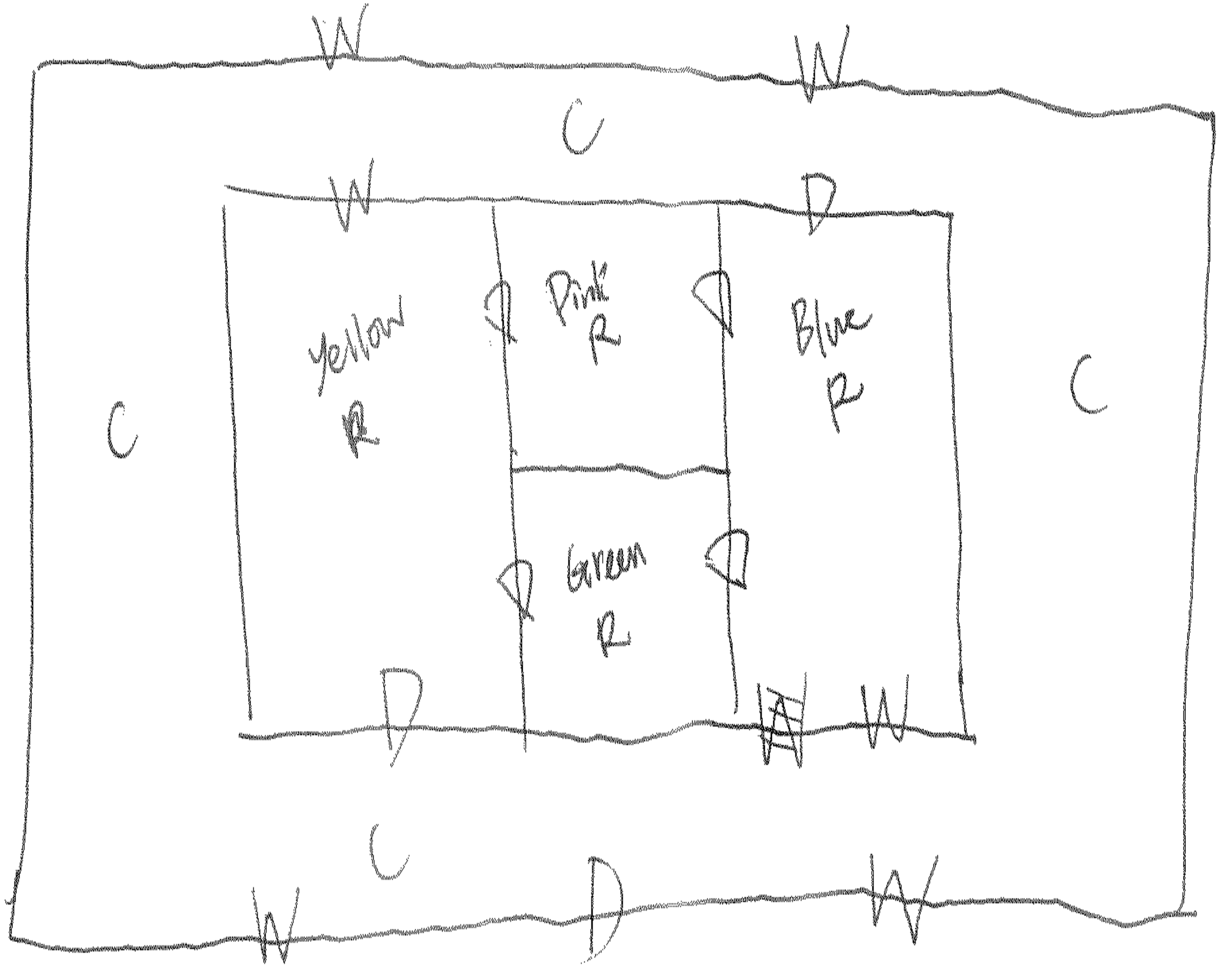

10 Map 1 Rotational Video

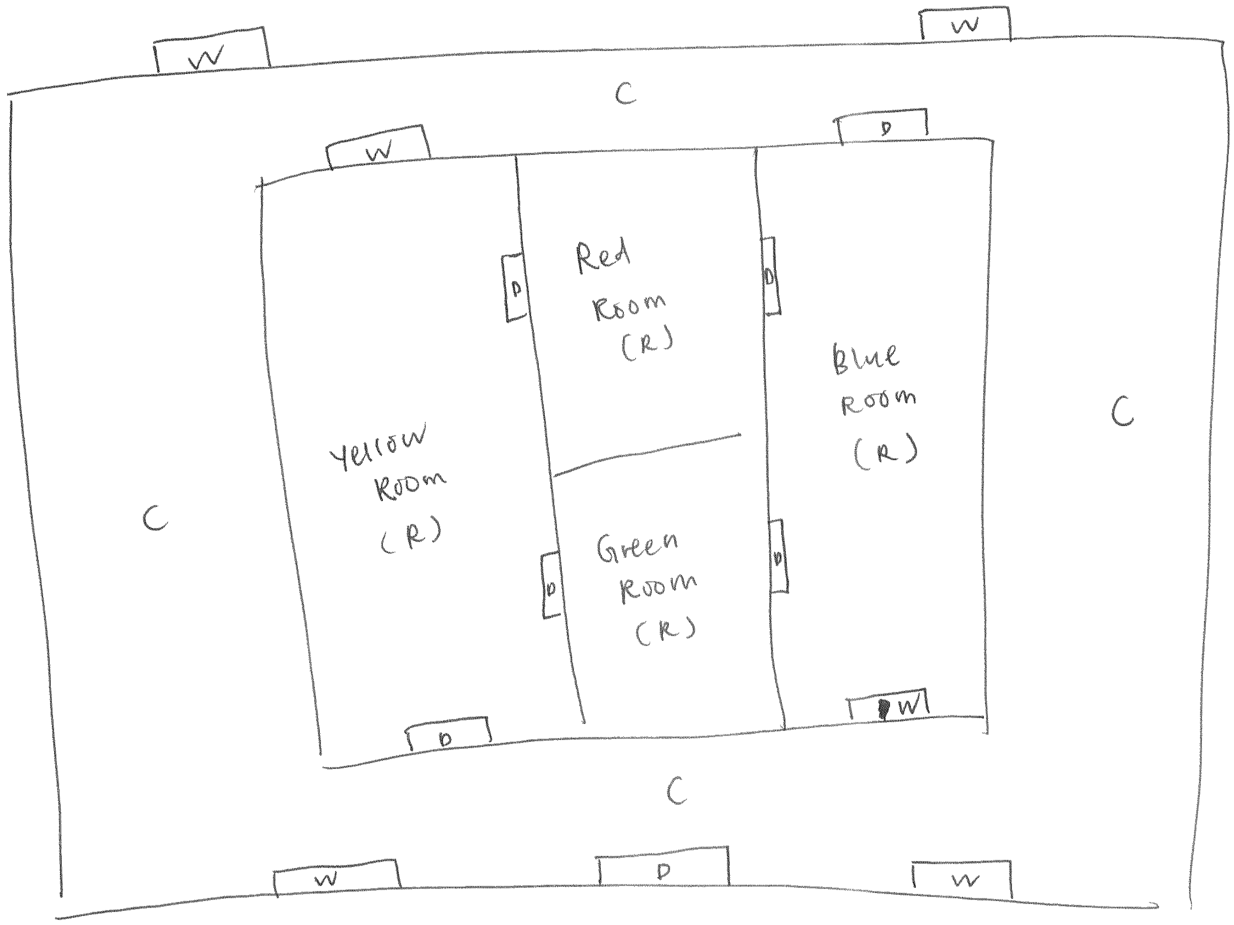

10 Map 2 Mirror Spoken

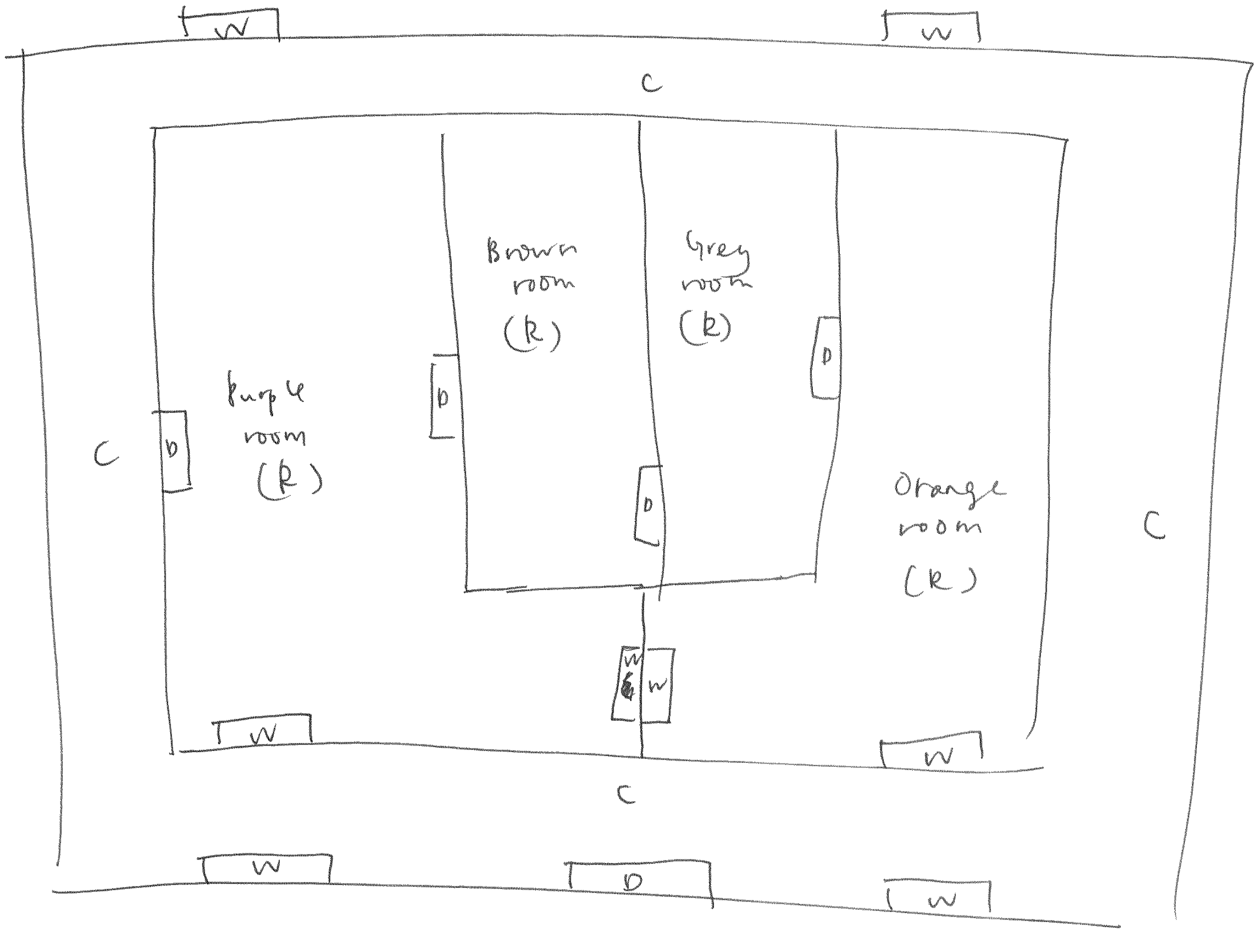

11 Map 1 Mirror Spoken

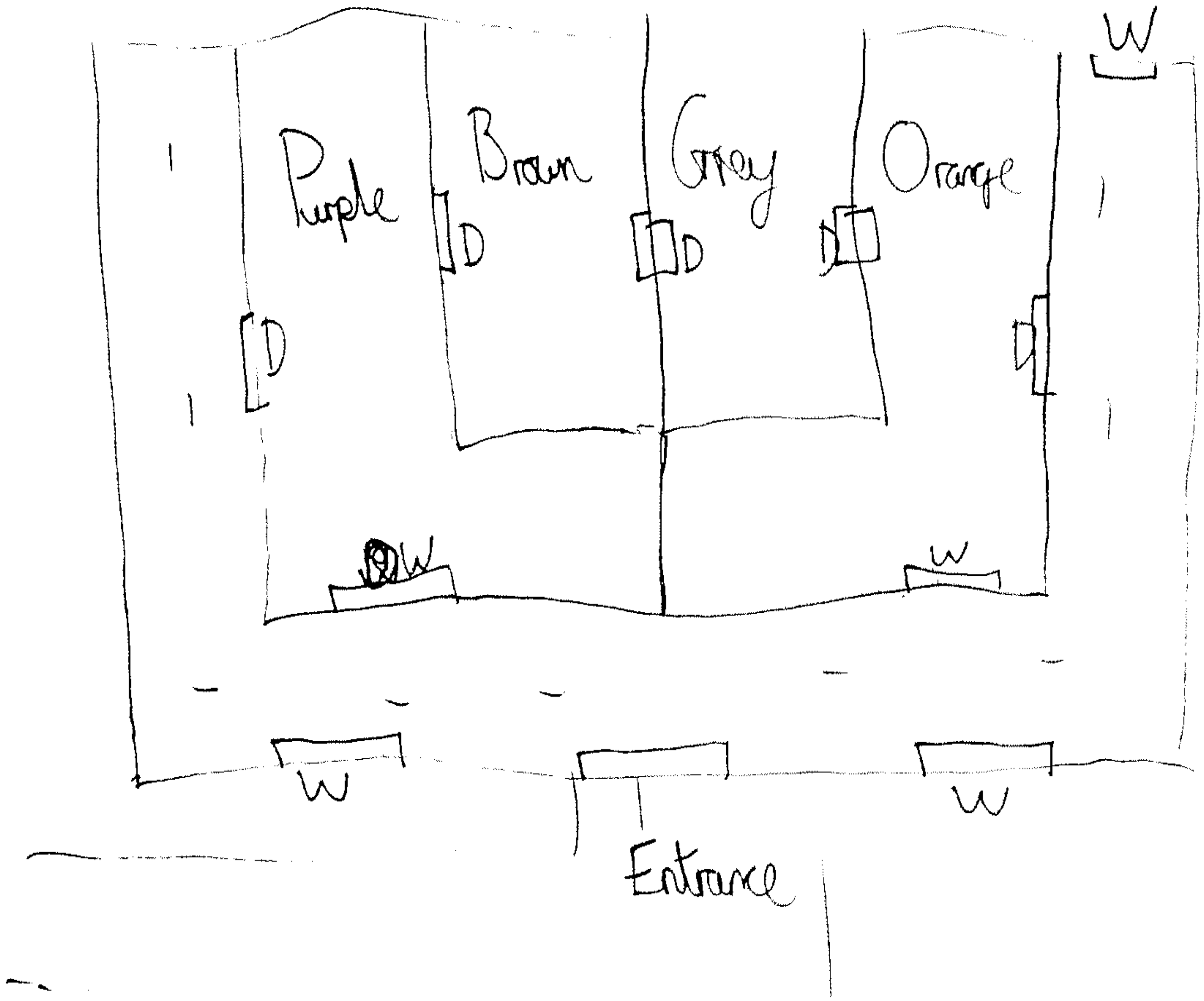

11 Map 2 Rotational Video

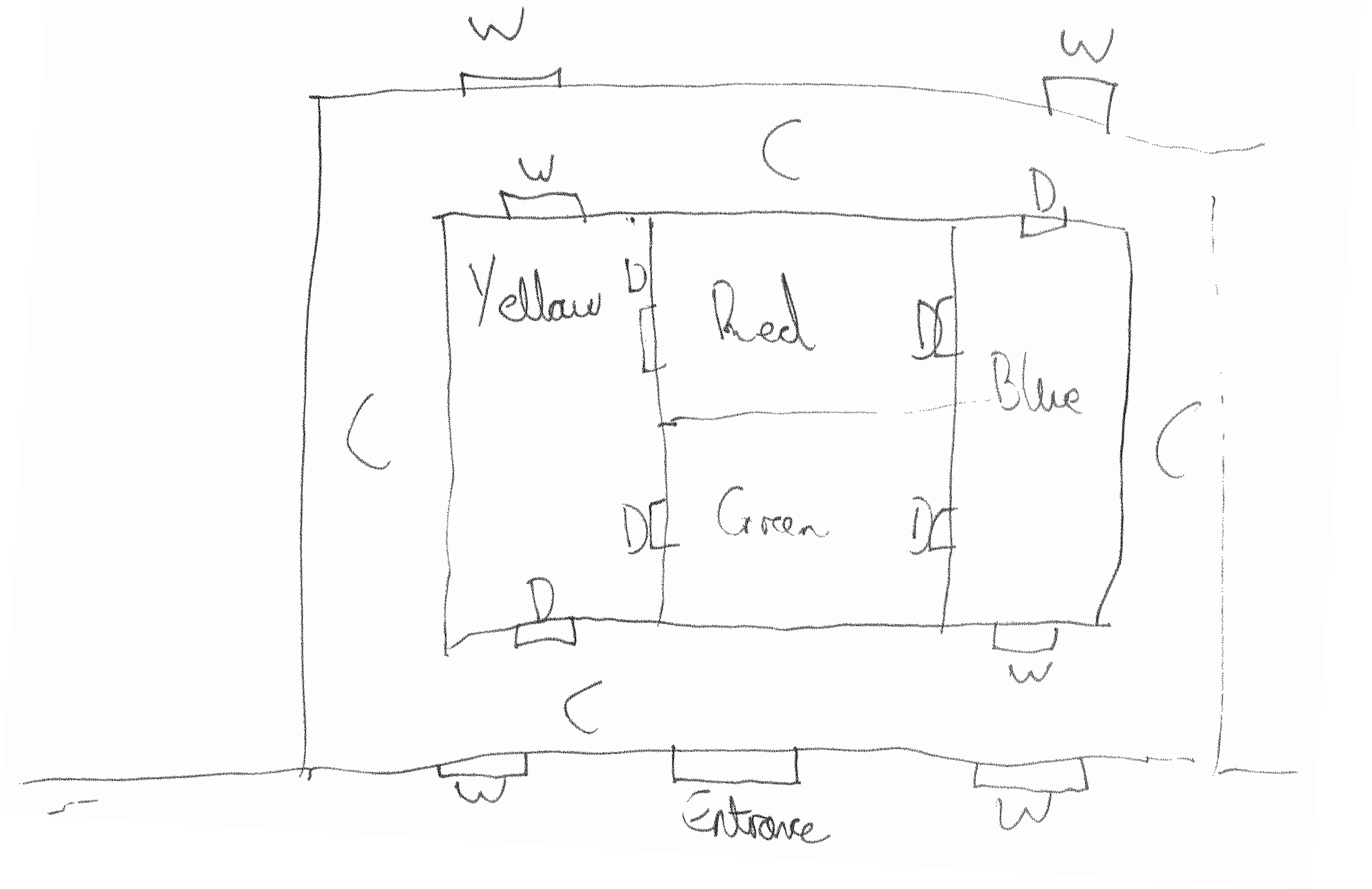

12 Map 1 Mirror Spoken

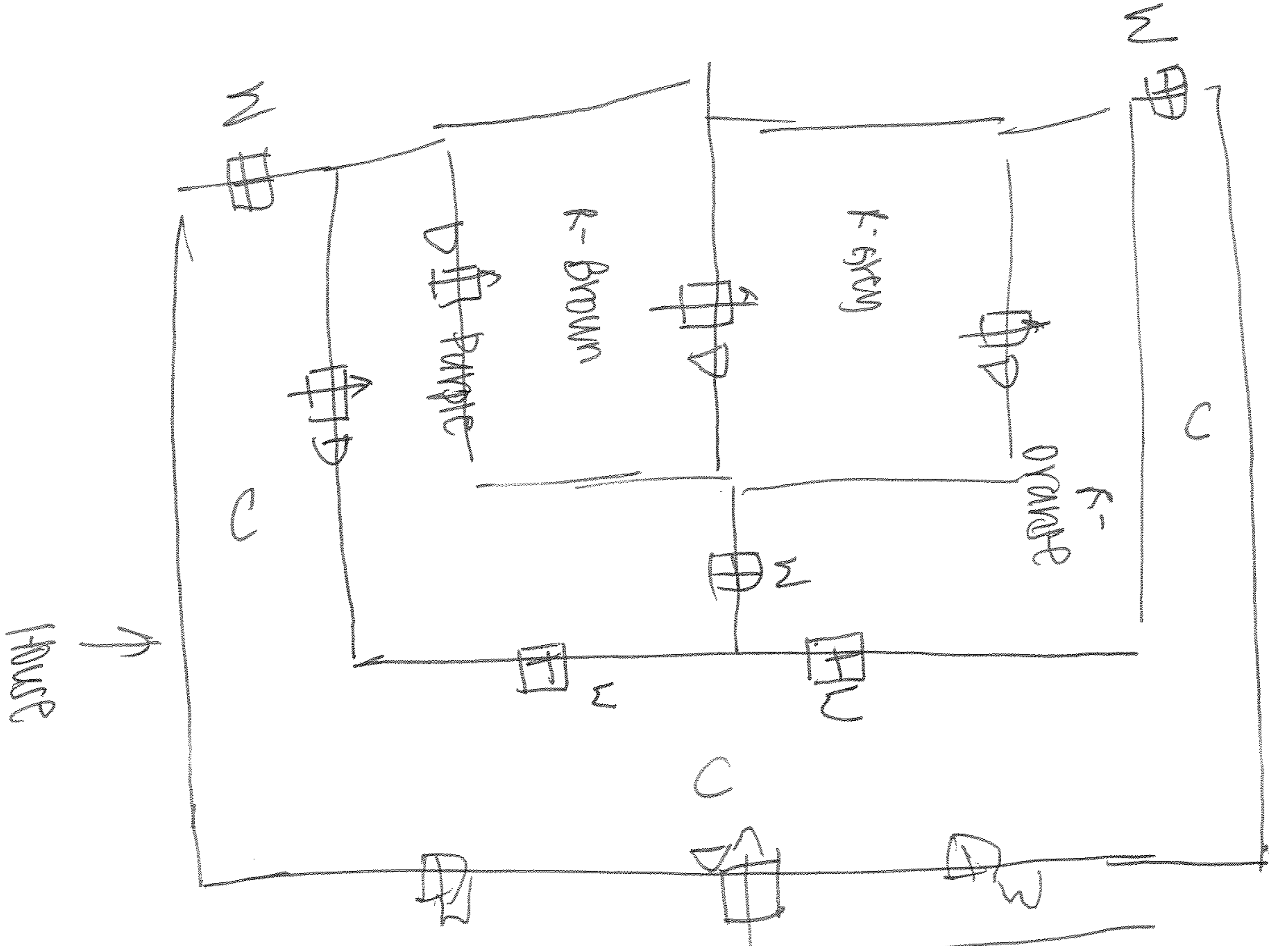

12 Map 2 Rotational Video

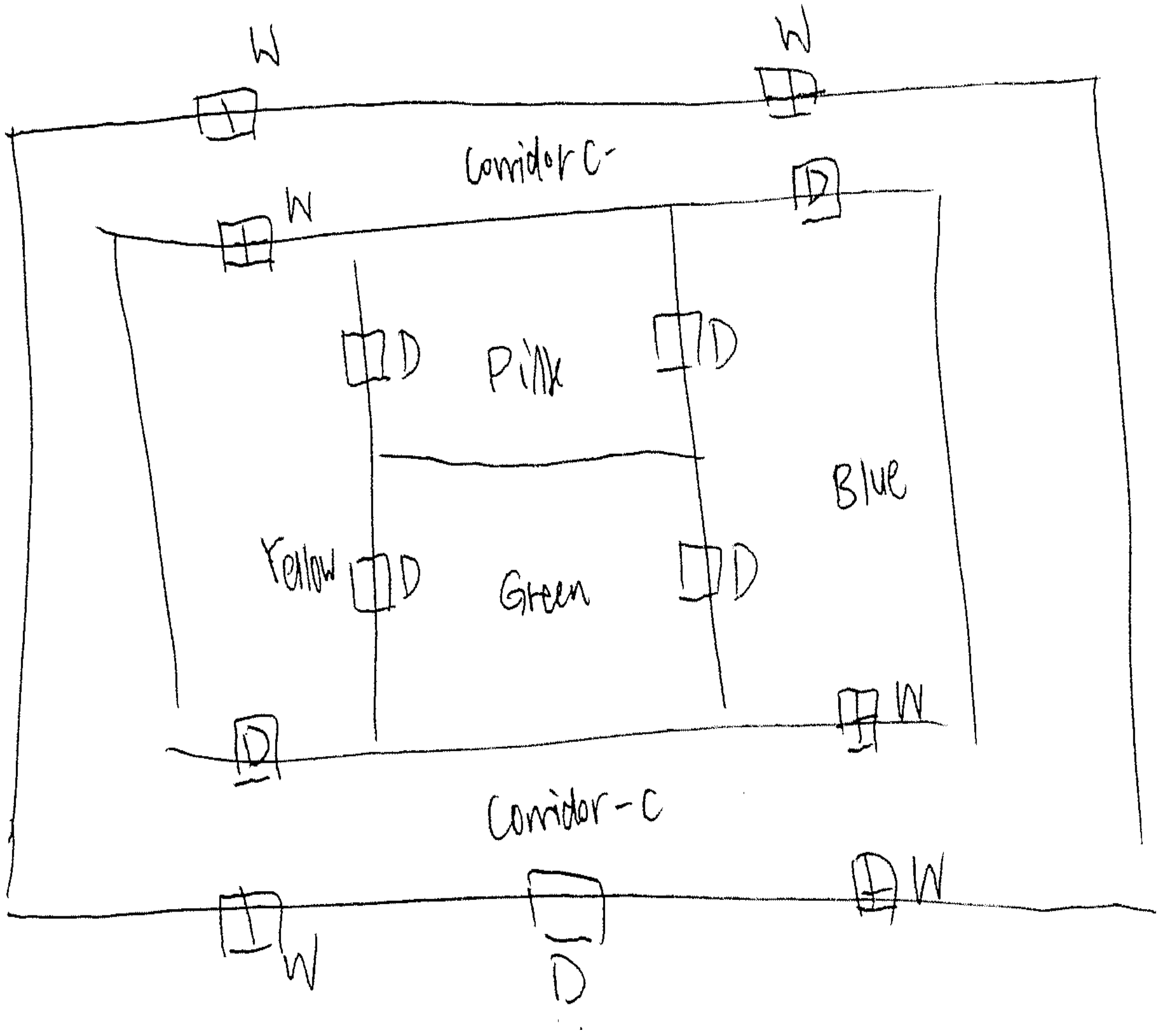

13 Map 1 Rotational Video

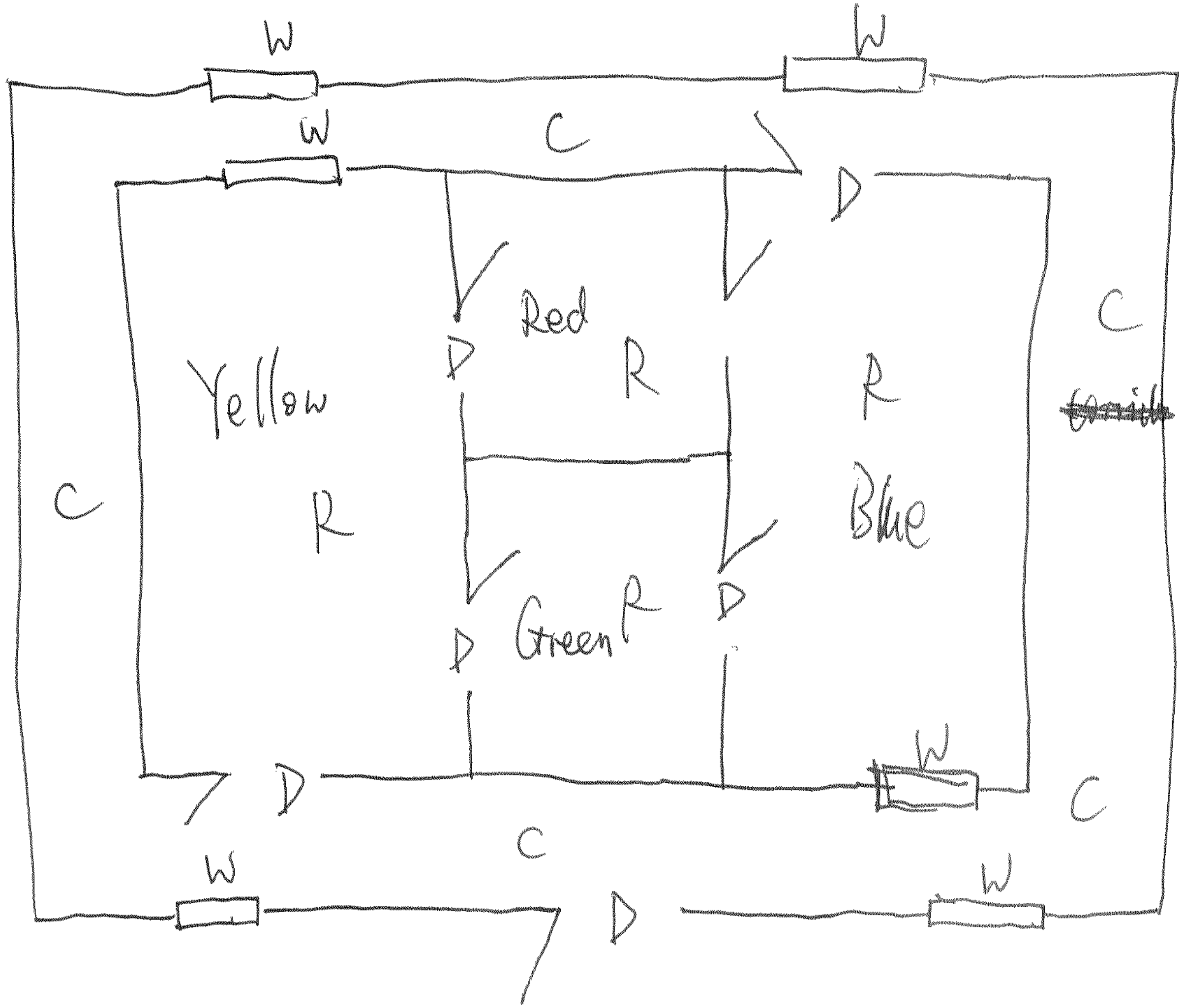

13 Map 2 Mirror Spoken

Missing

14 Map 1 Mirror Video

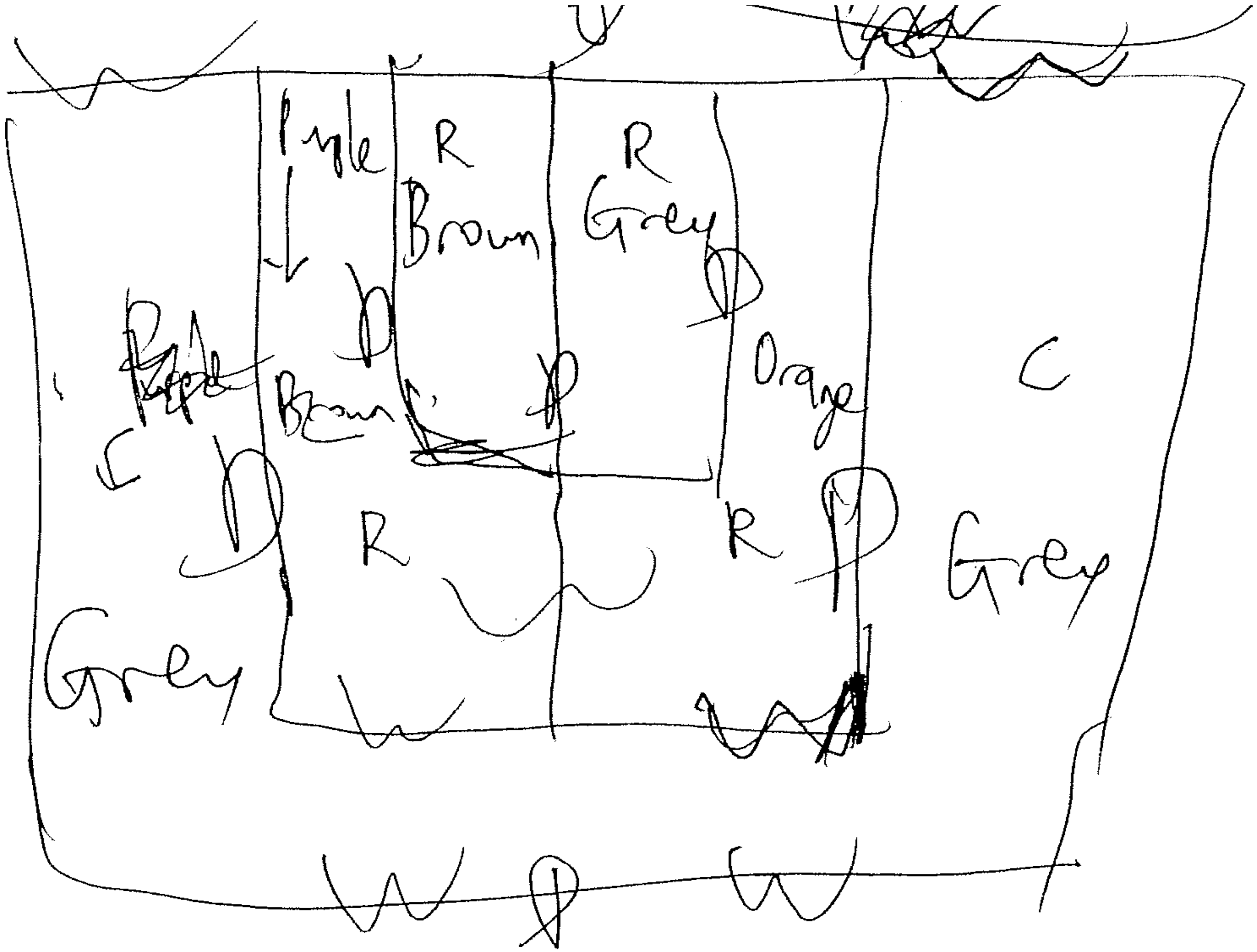

14 Map 2 Rotational Spoken

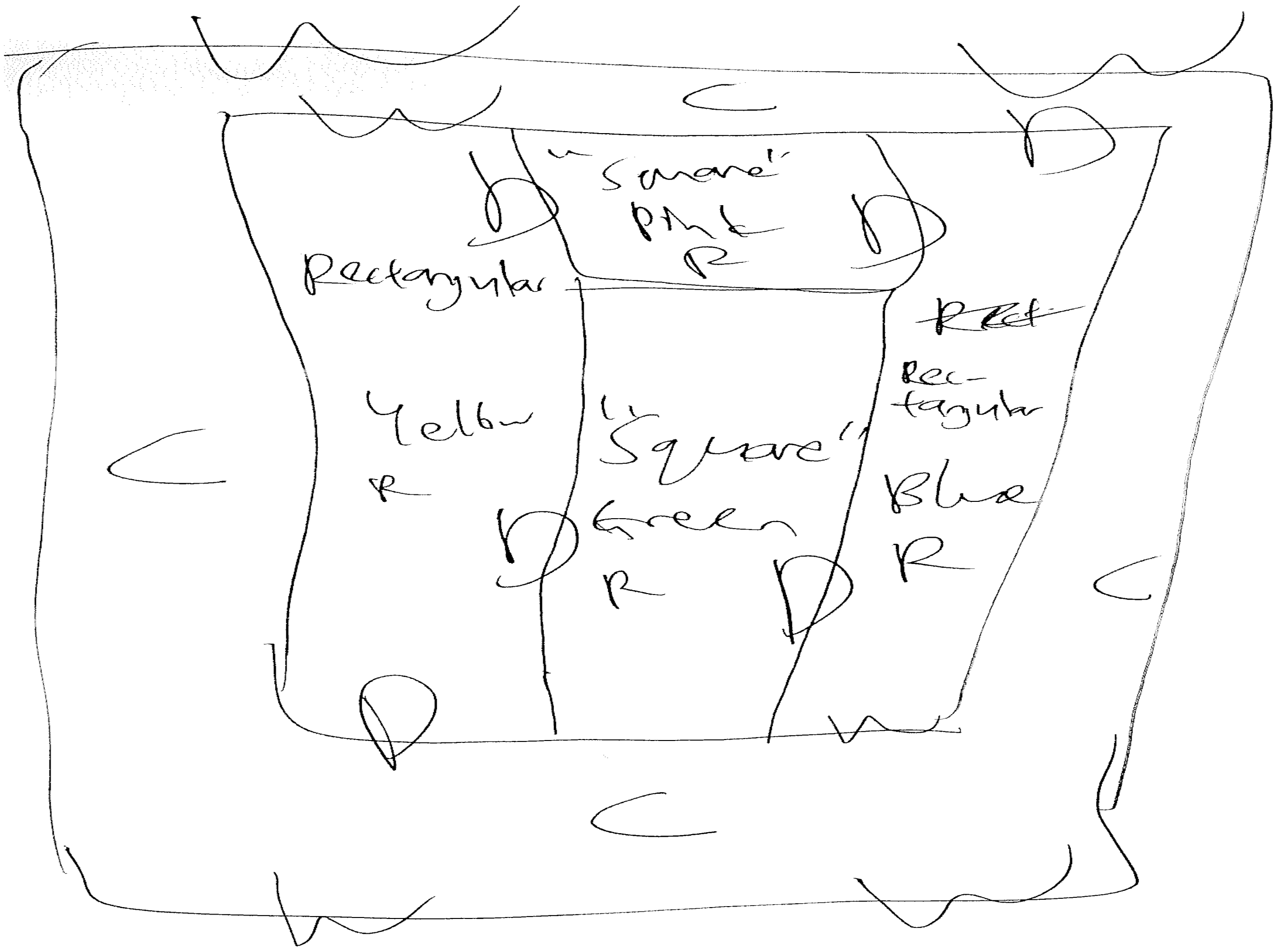

15 Map 1 Mirror Video

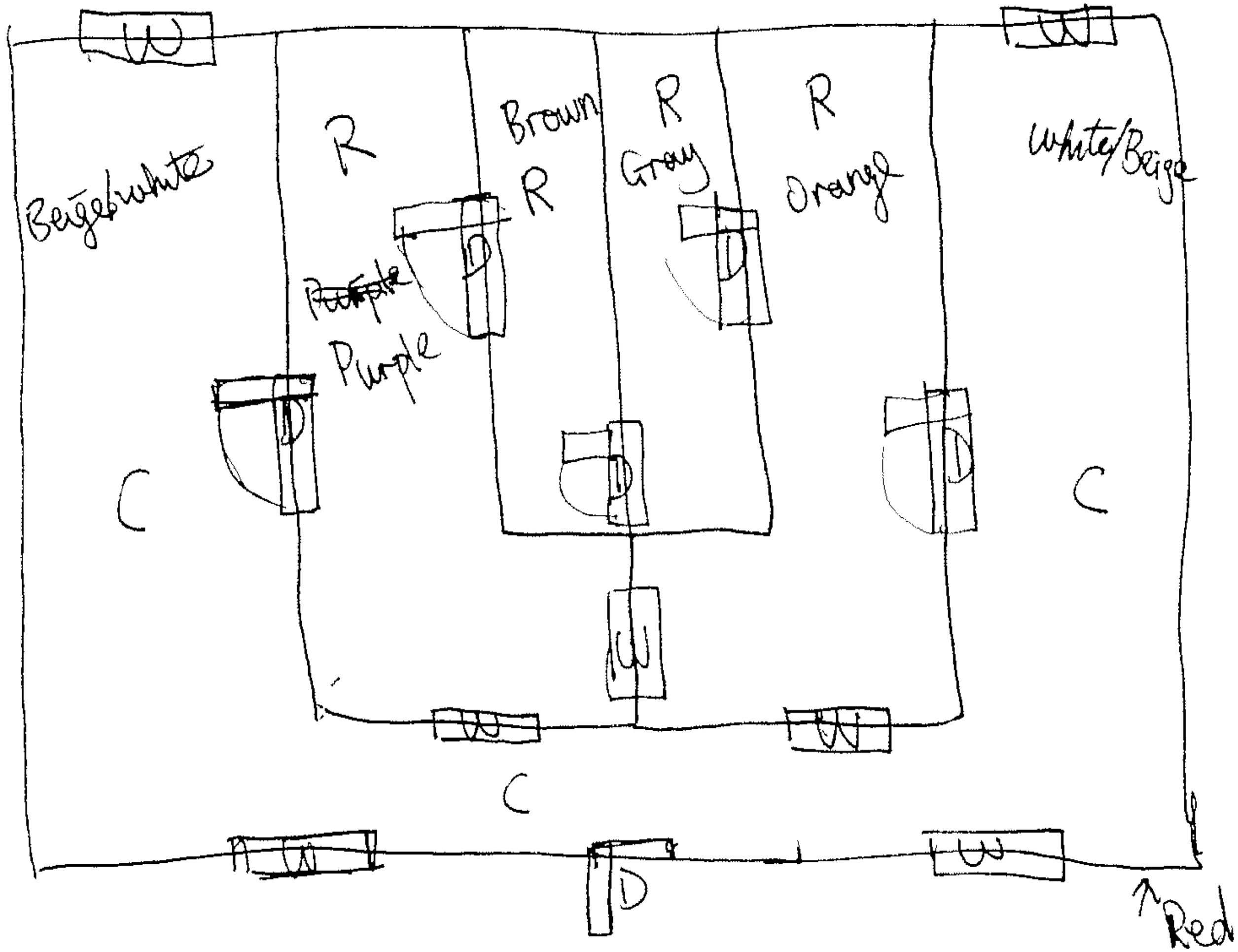

15 Map 2 Rotational Spoken

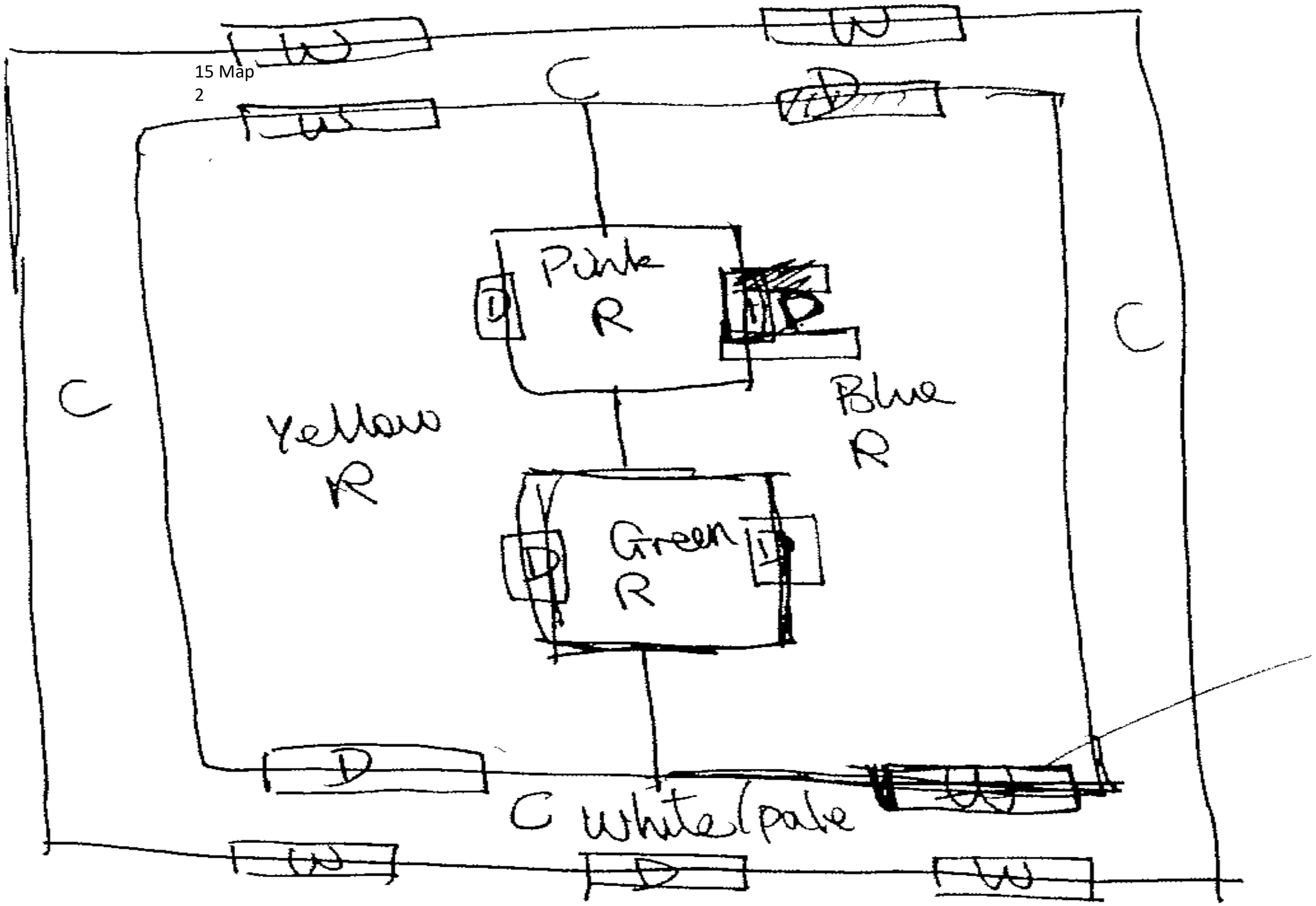

16 Map 1 Mirror Spoken

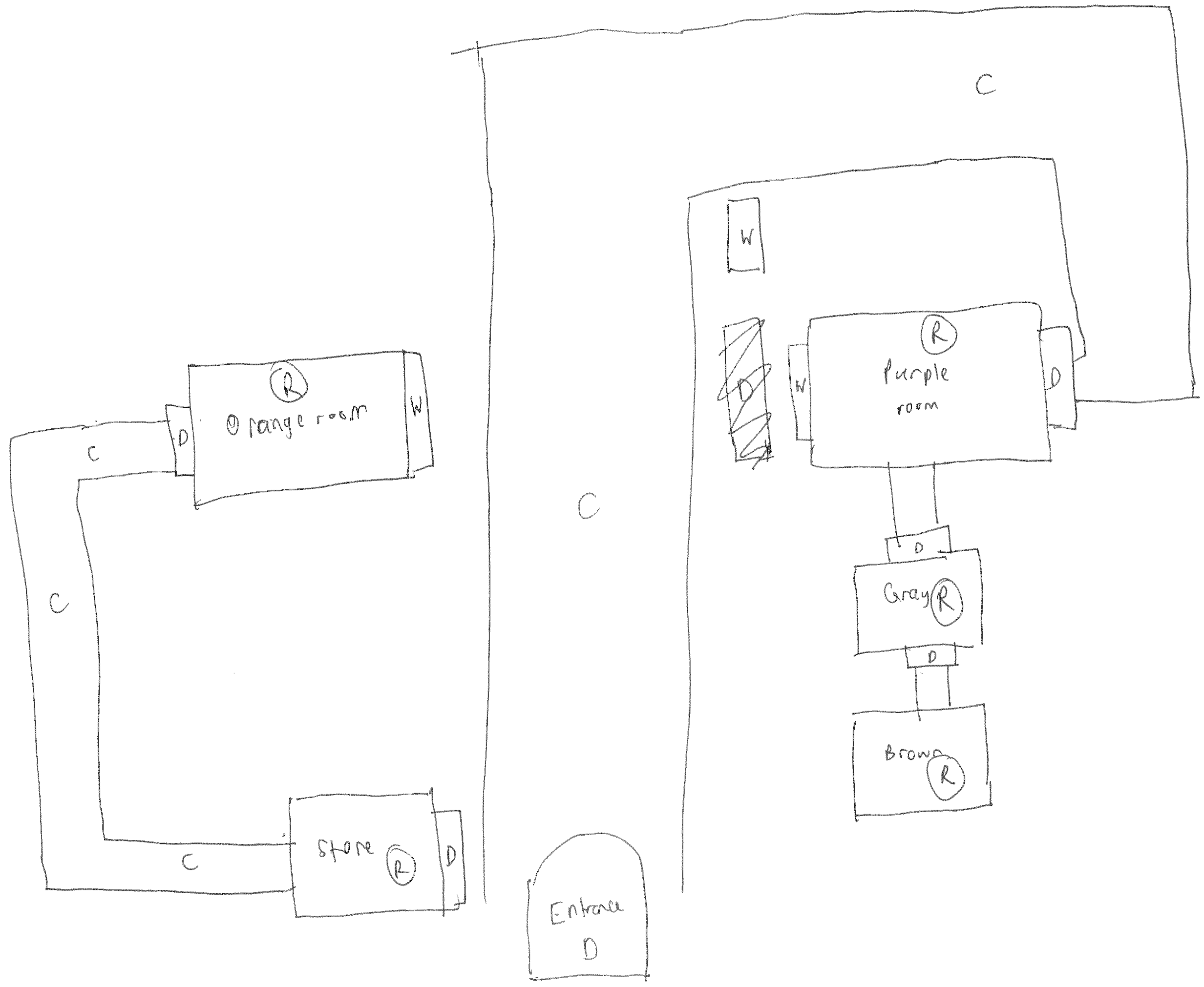

16 Map 2 Rotational Video

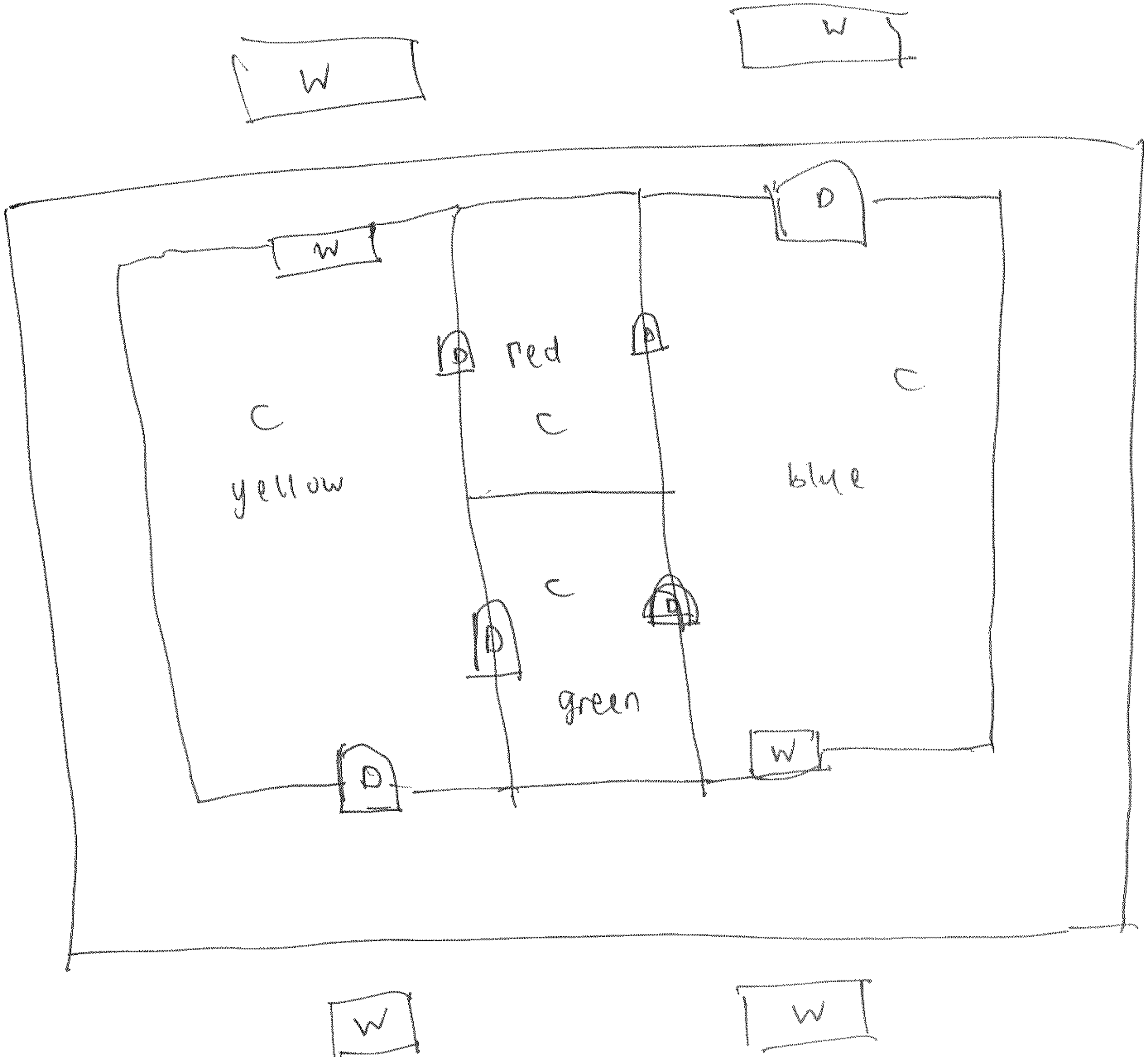

17 Map 1 Rotational Video

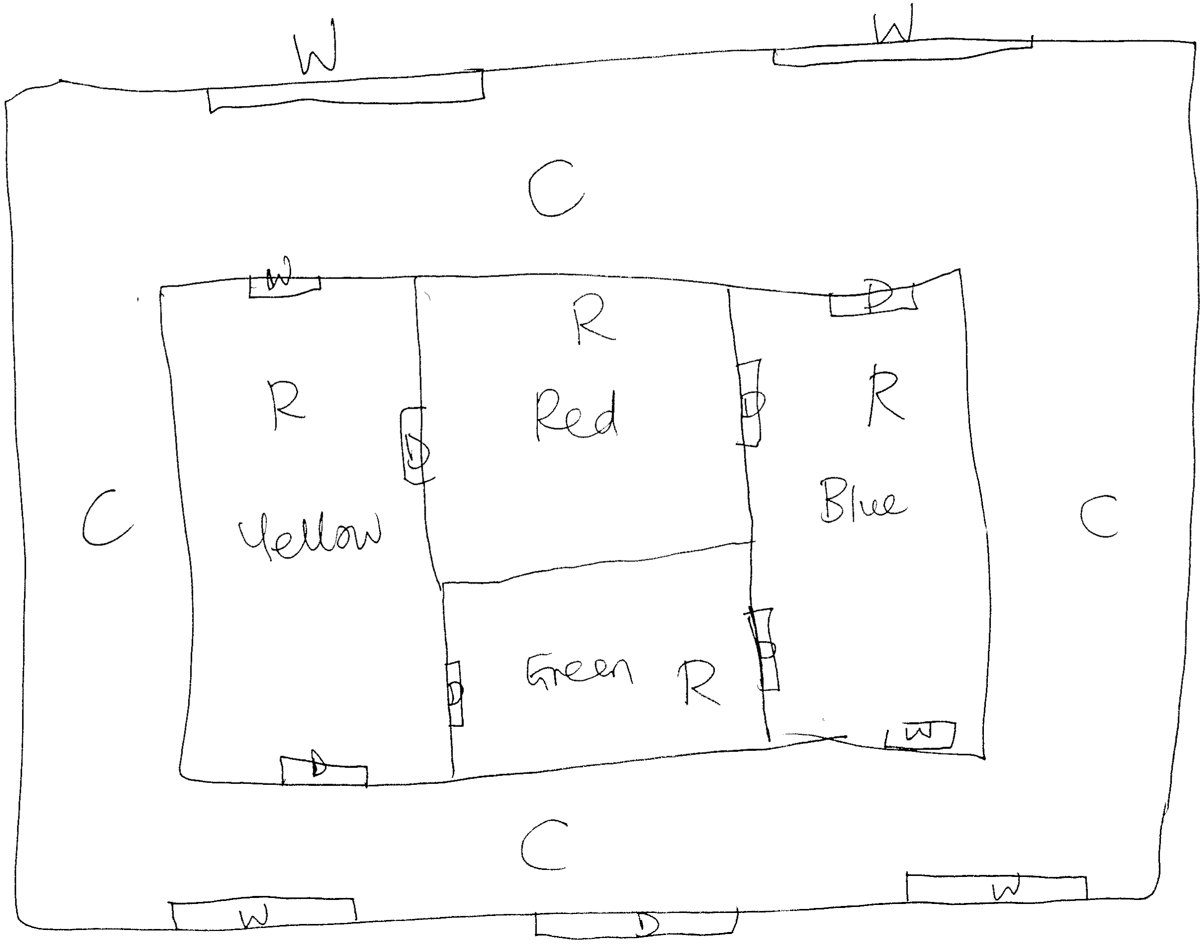

17 Map 2 Mirror Spoken

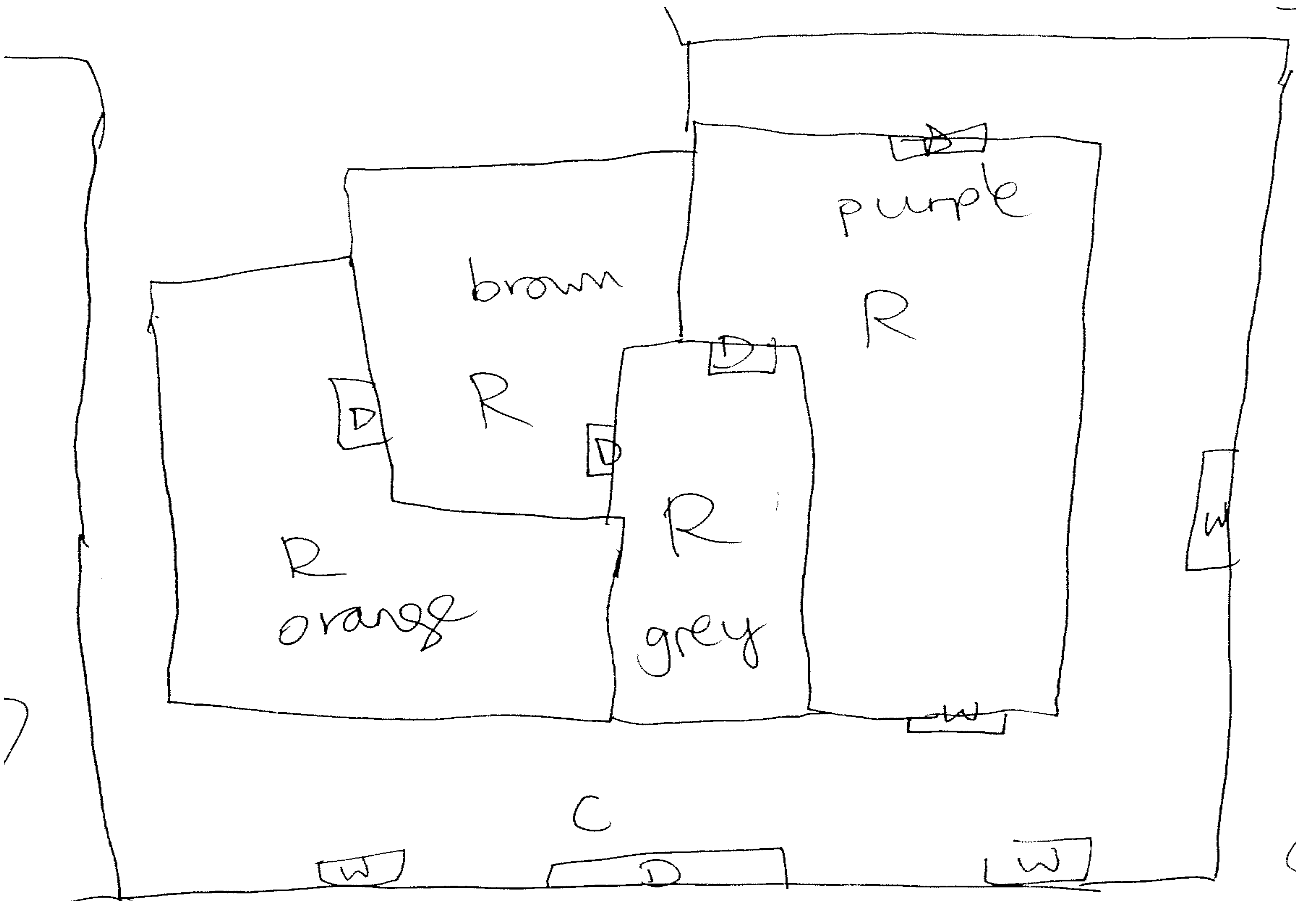

18 Map 1 Mirror Video

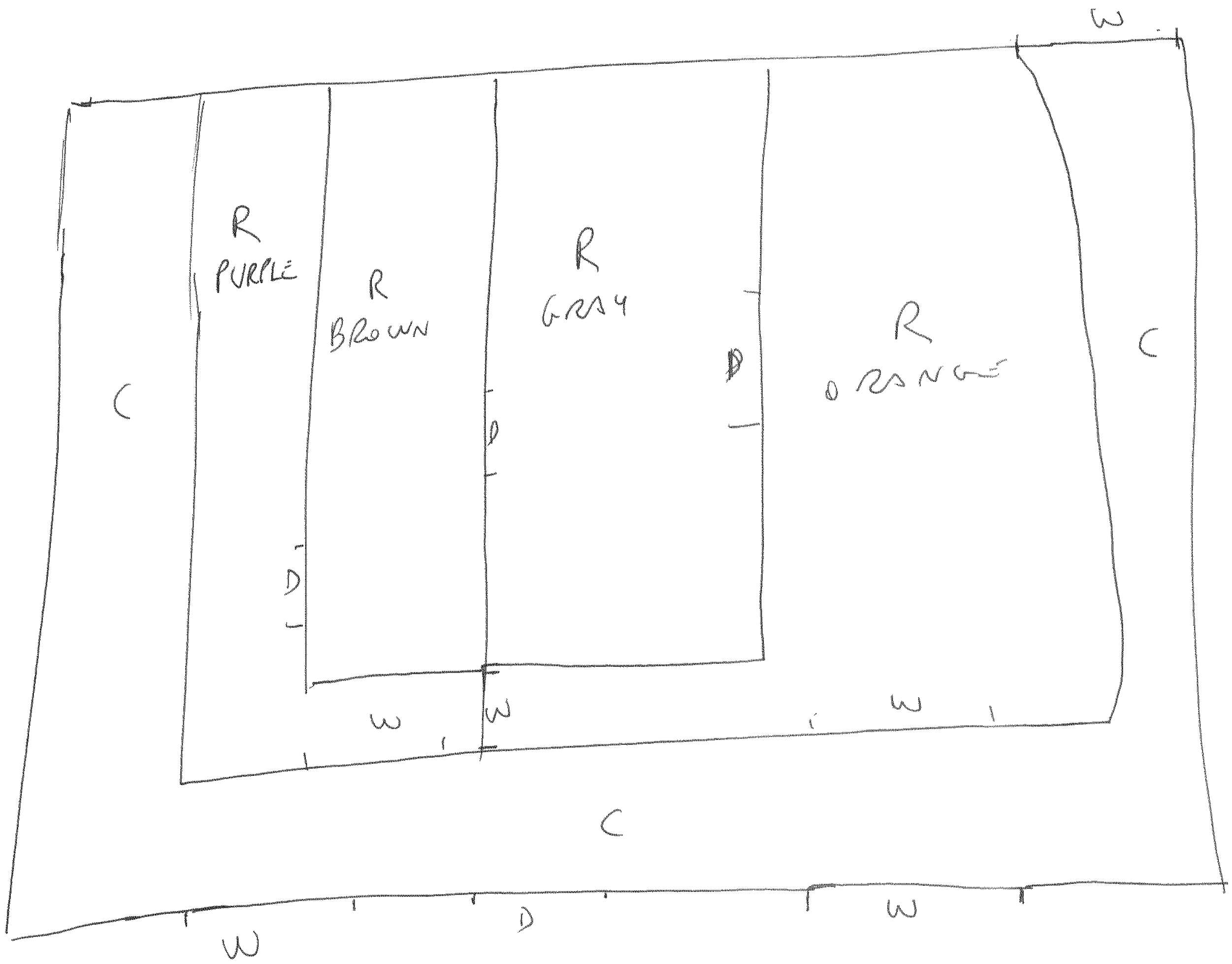

18 Map 2 Rotational Spoken

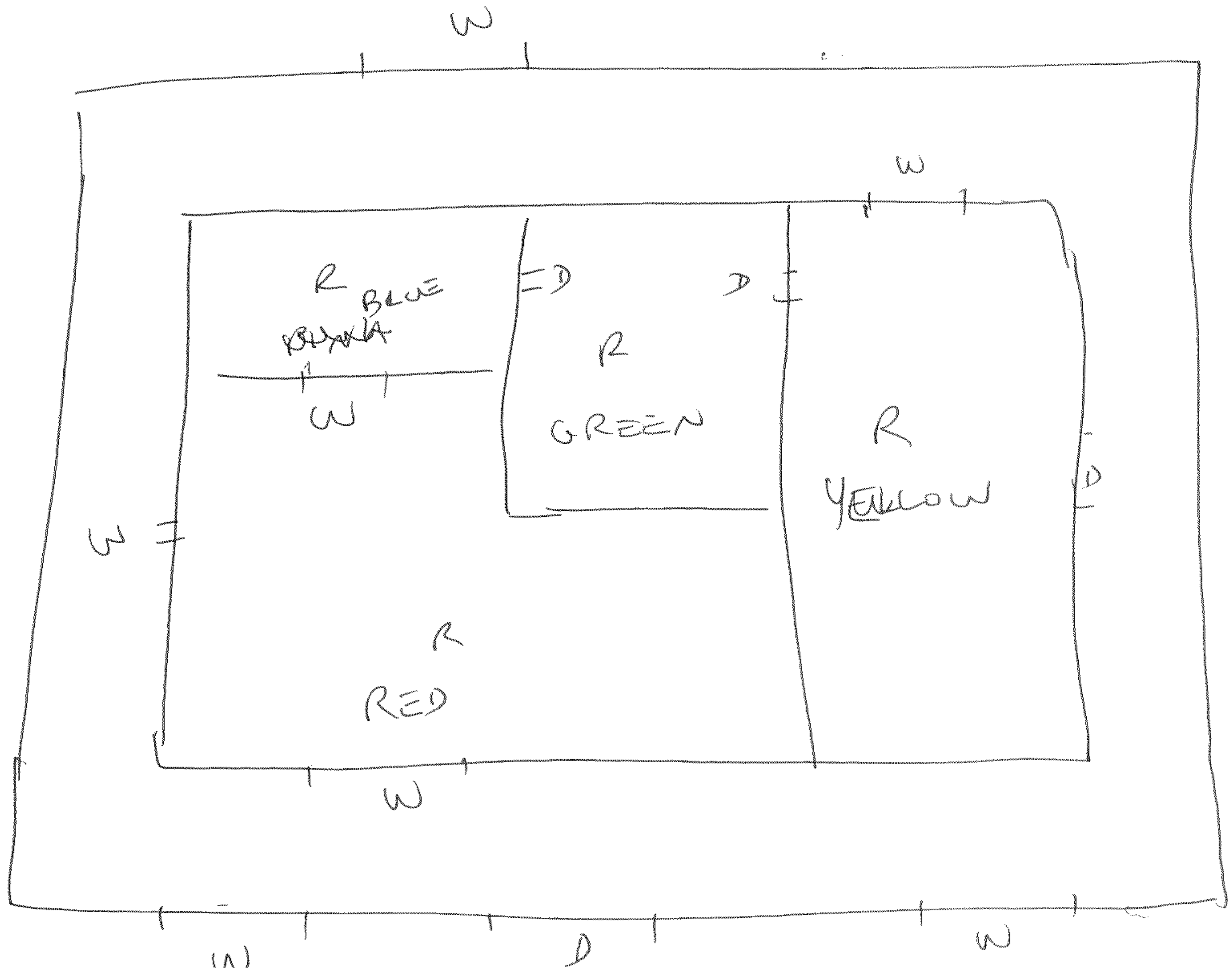

19 Map 1 Rotational Video

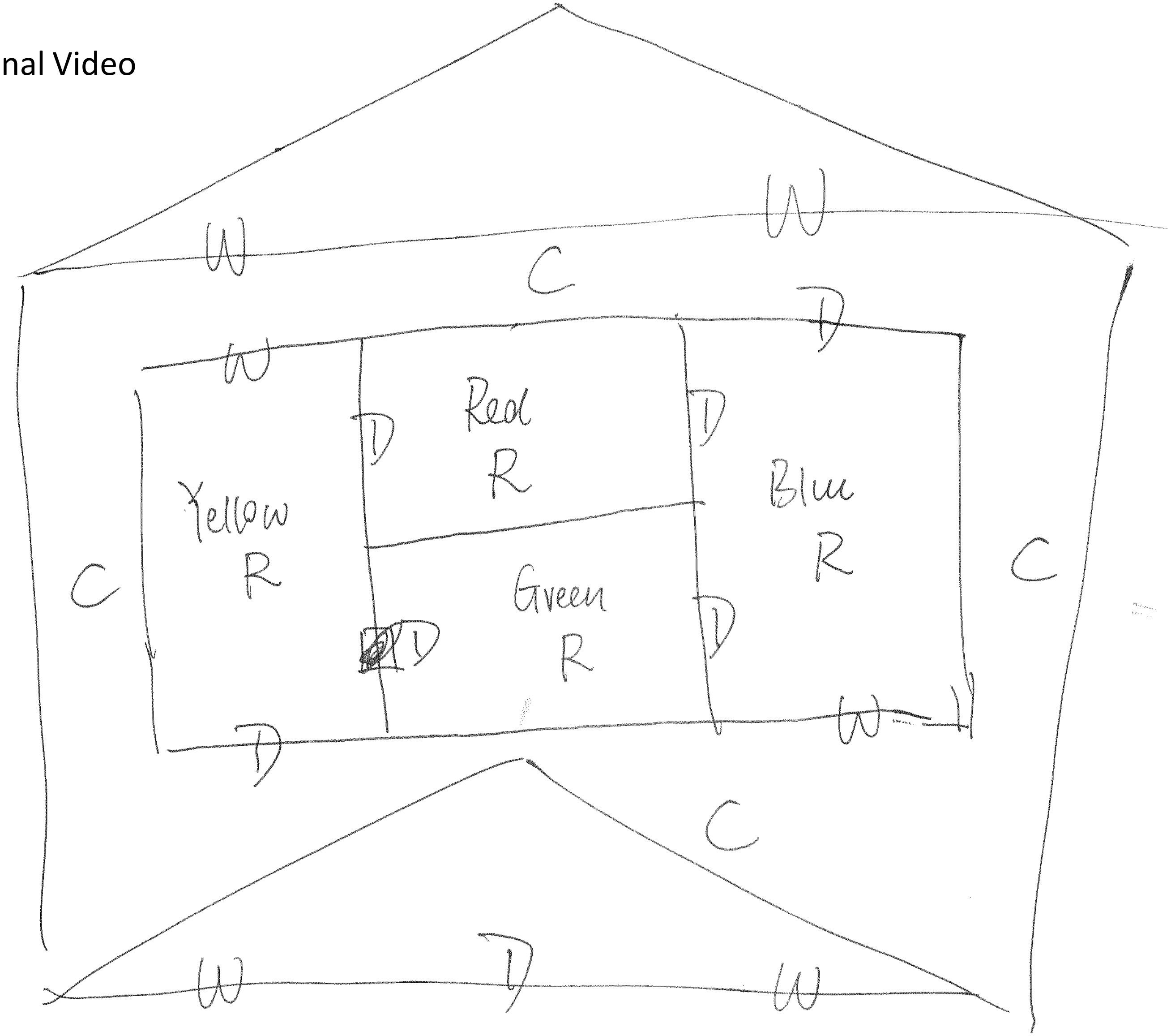

19 Map 2 Mirror Spoken

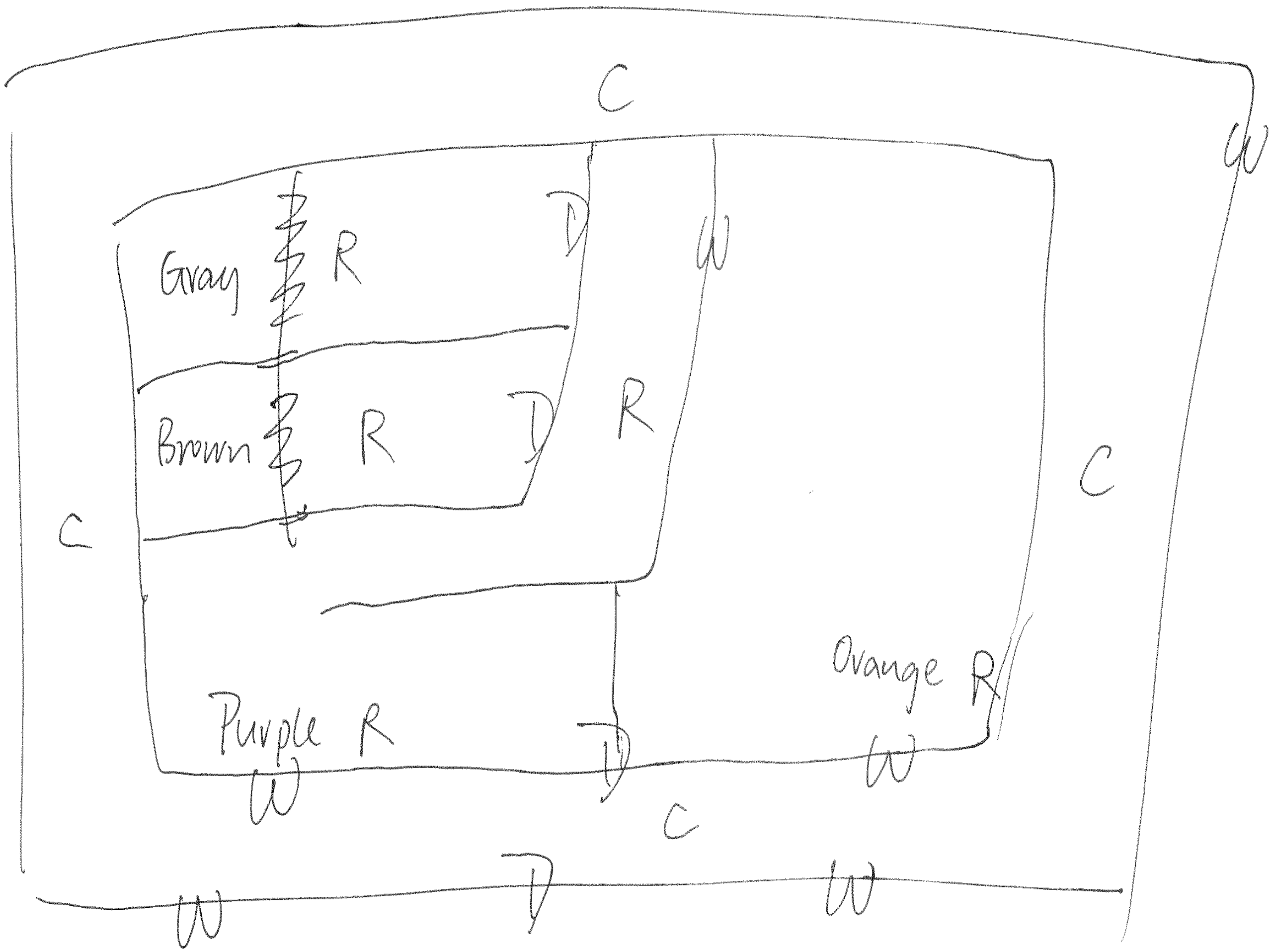

20 Map 1 Mirror Spoken

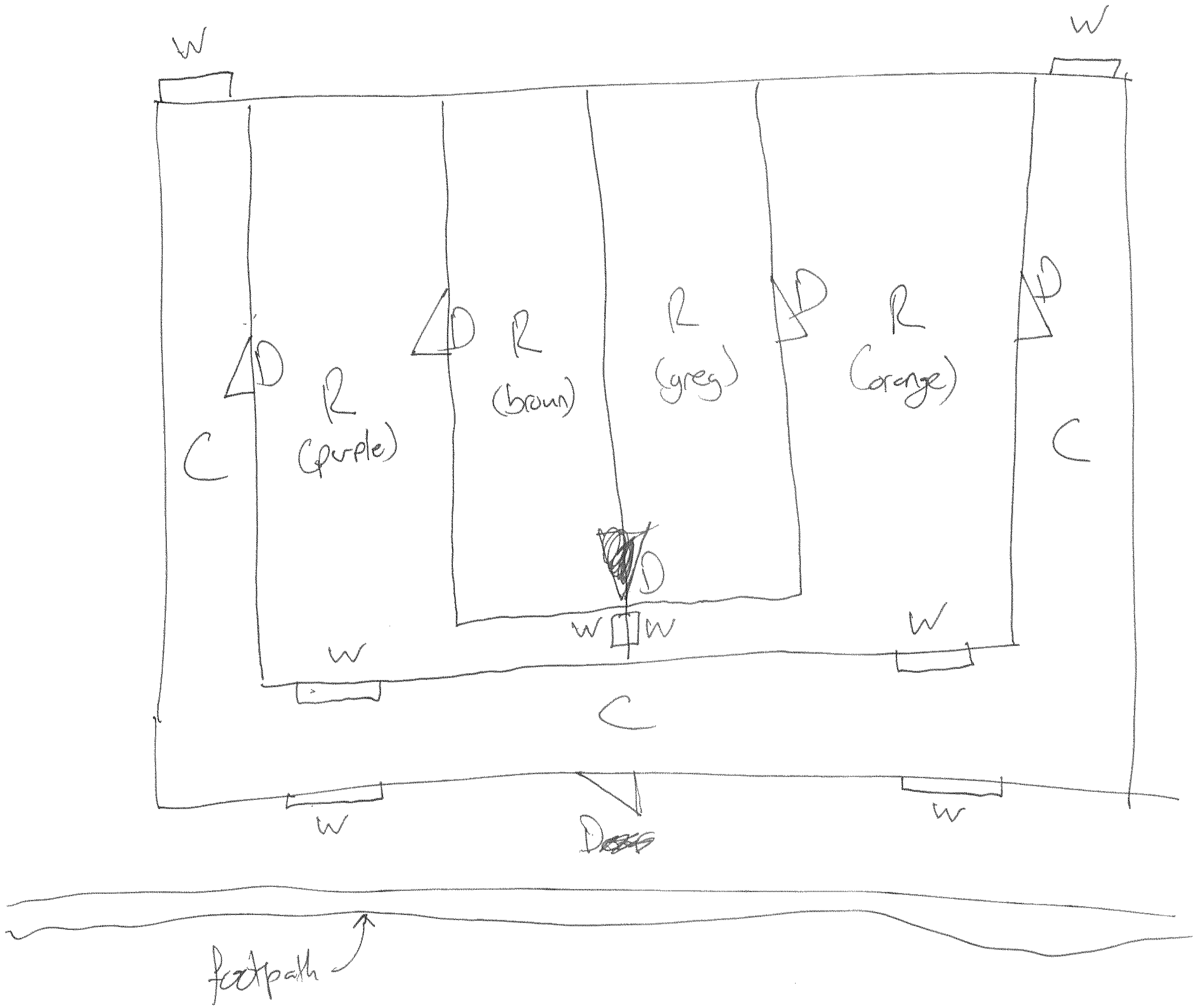

20 Map 2 Rotational Video

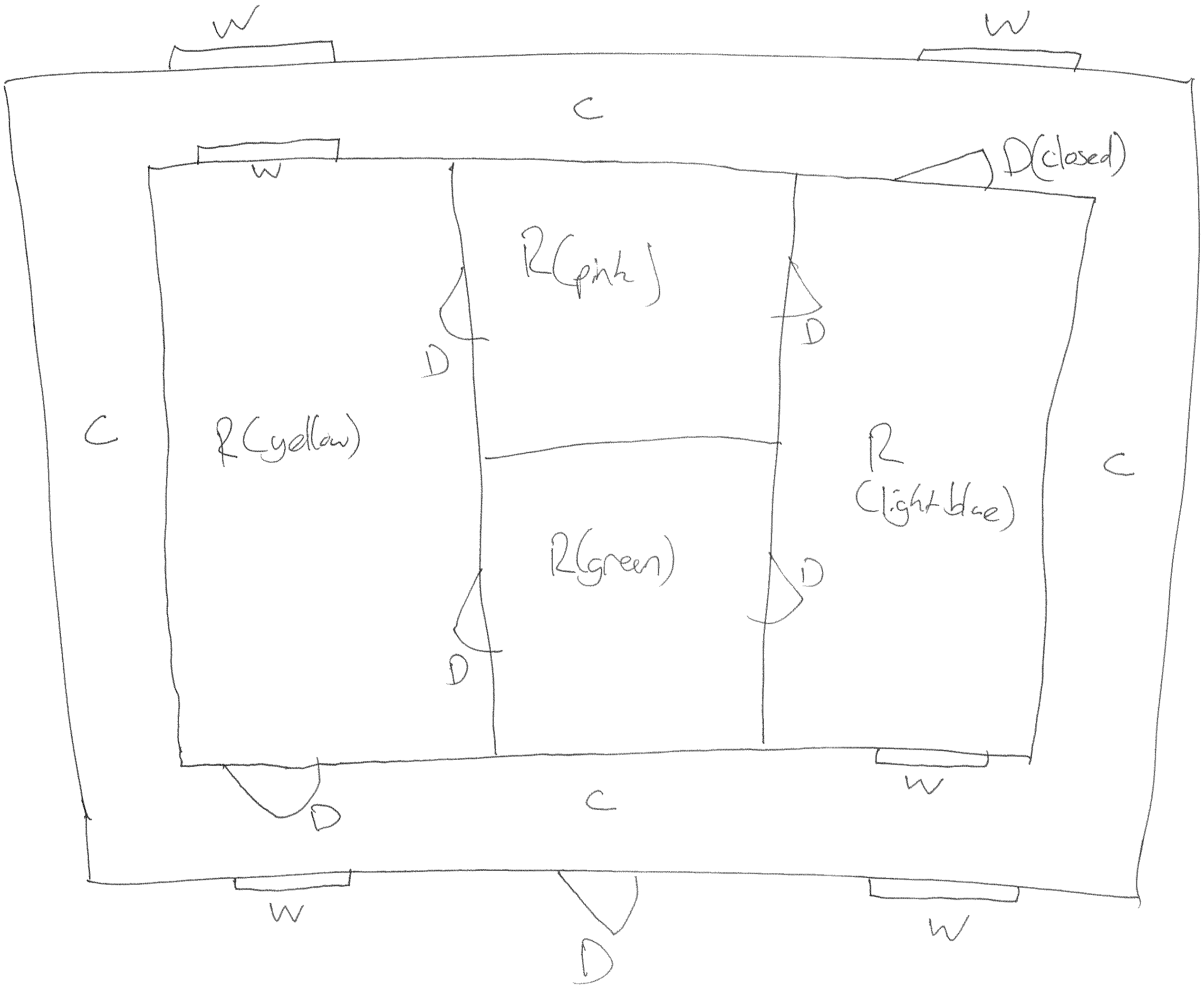

21 map 1 Mirror Spoken

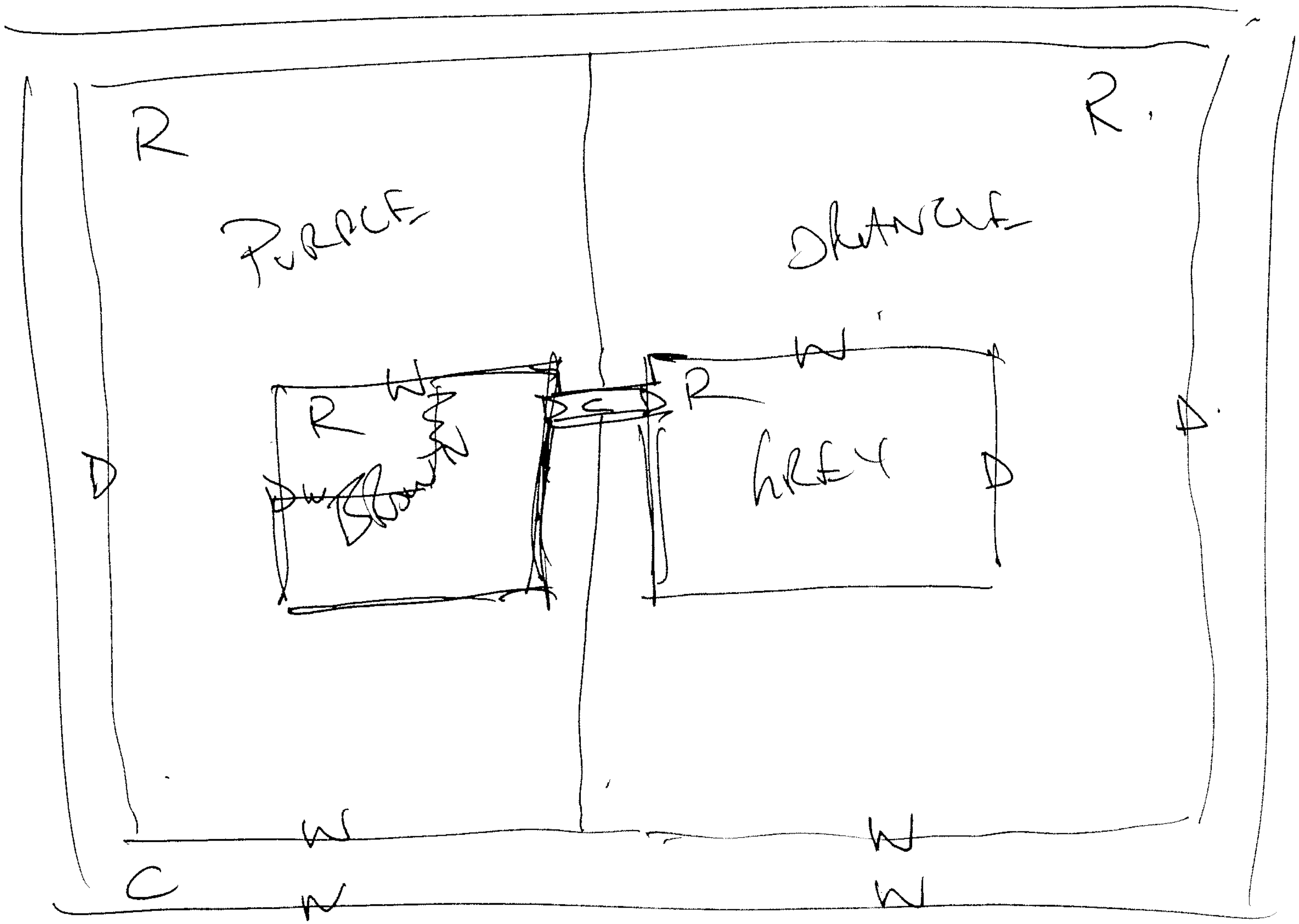

21 map 2 Rotational Video

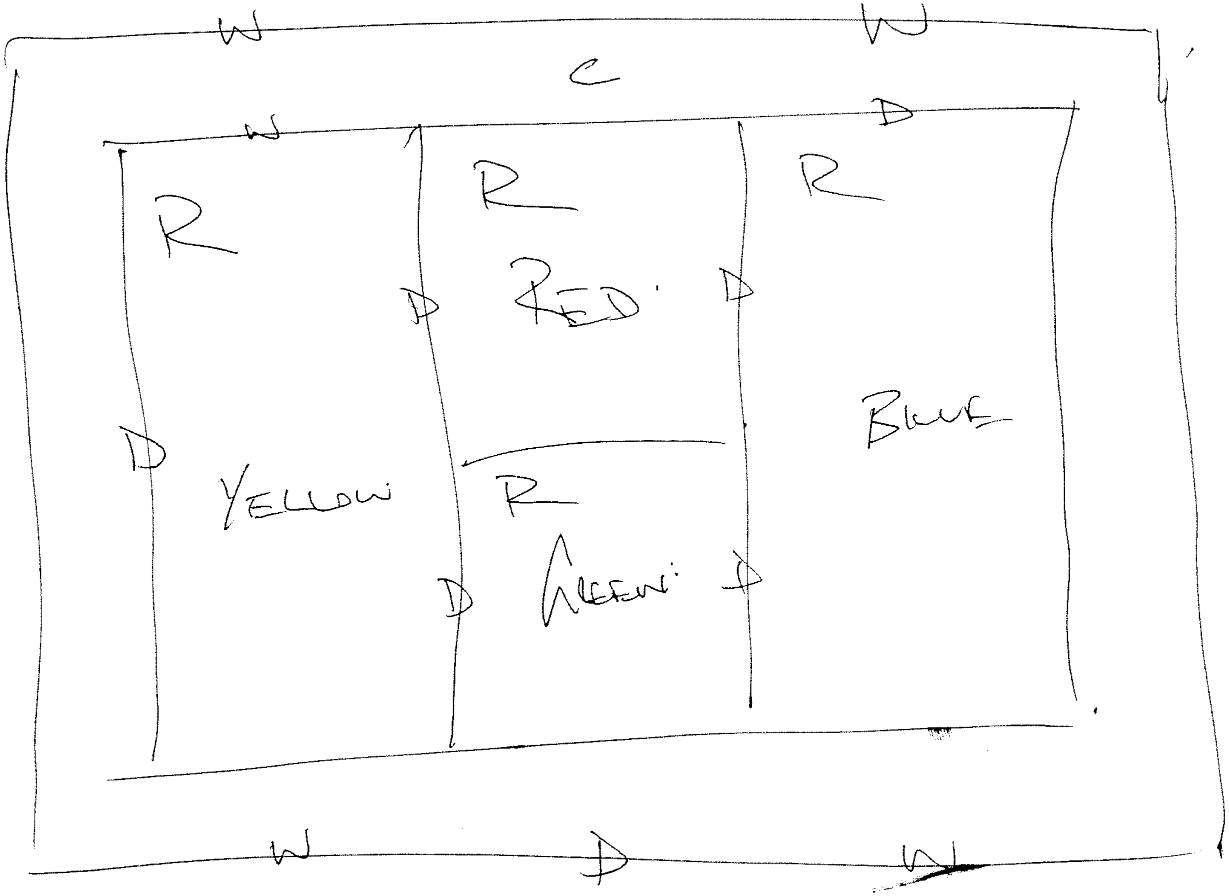

22 map 1 Rotational Video

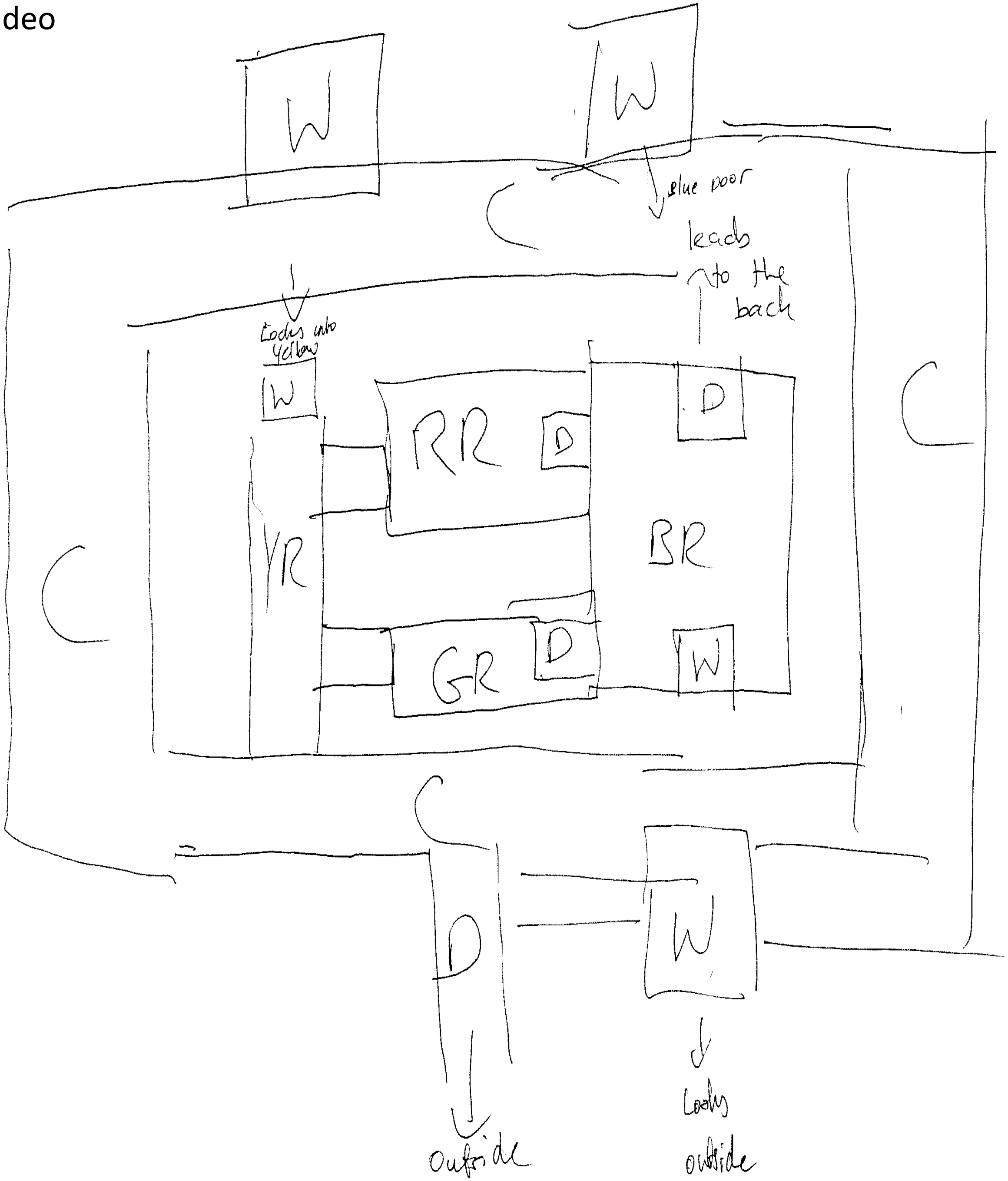

22 map 2 Mirror Spoken

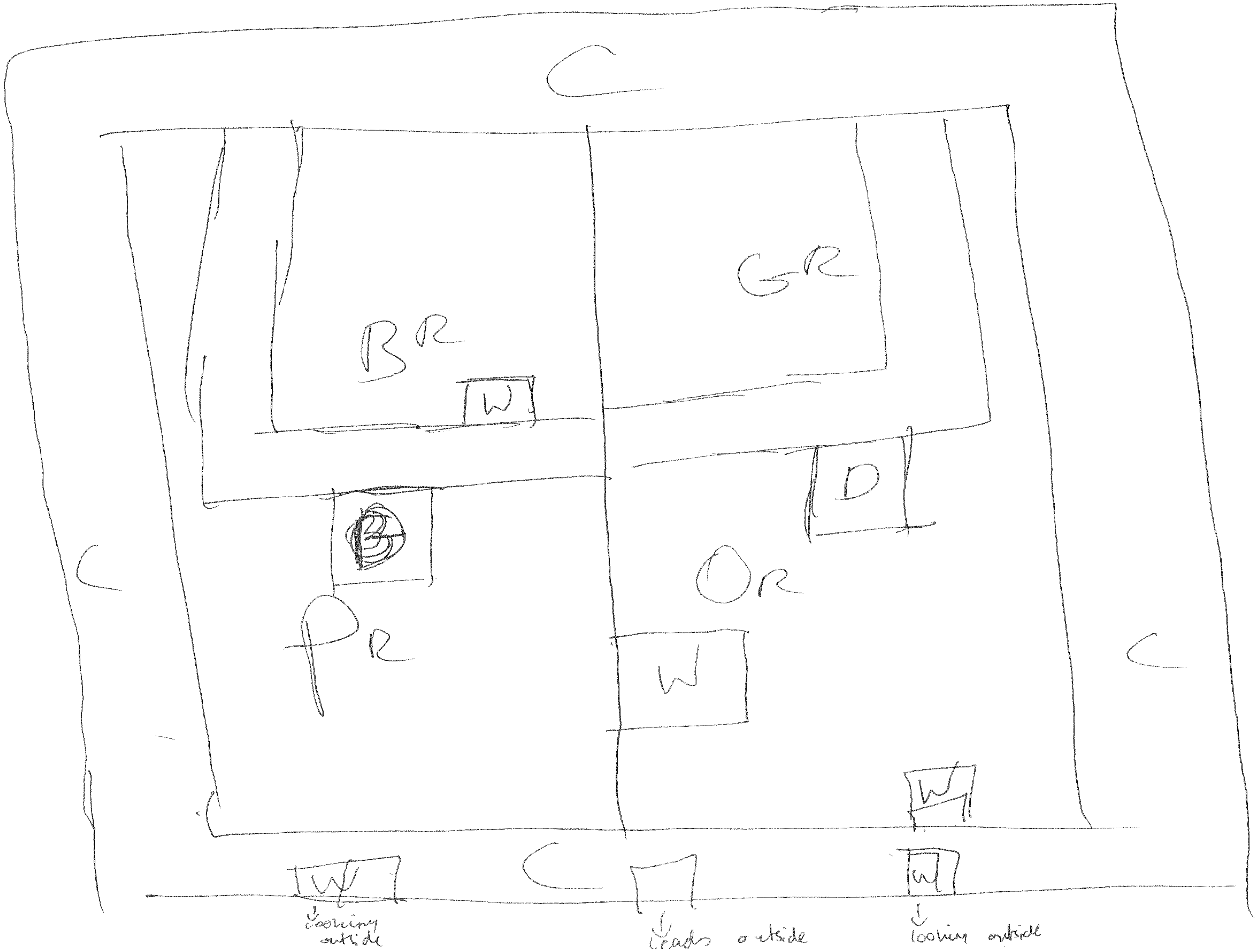

23 map 1 Mirror Video

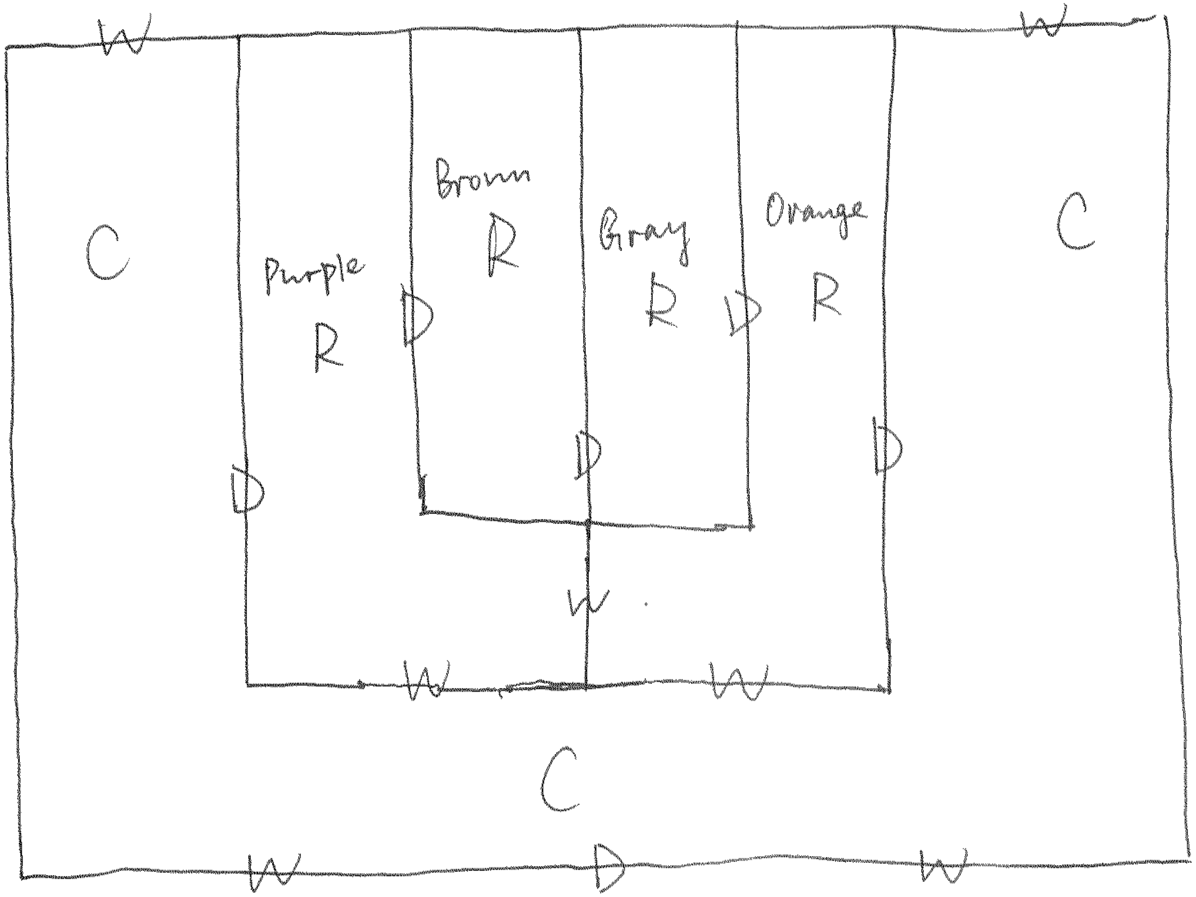

23 map 2 Rotational Spoken

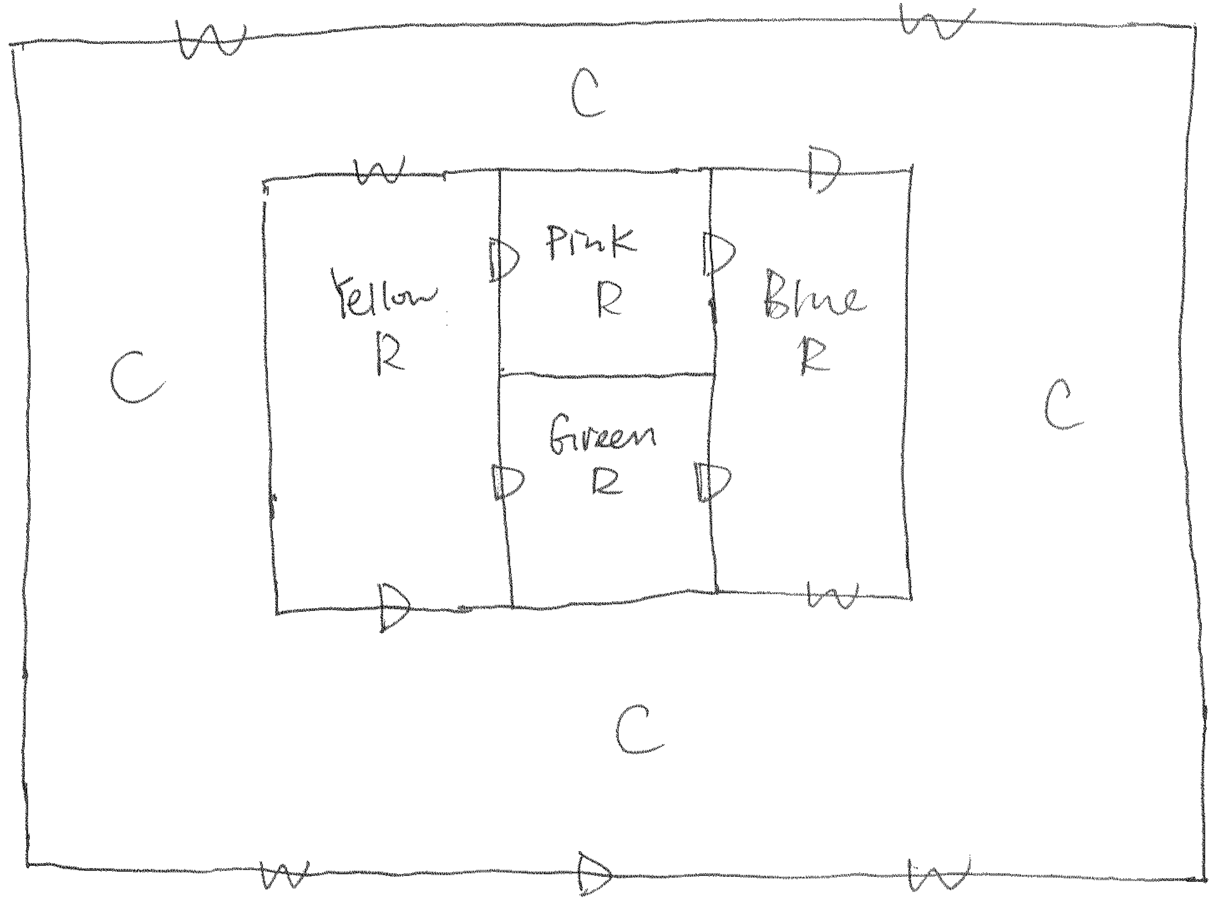

24 map 1 Rotational Spoken

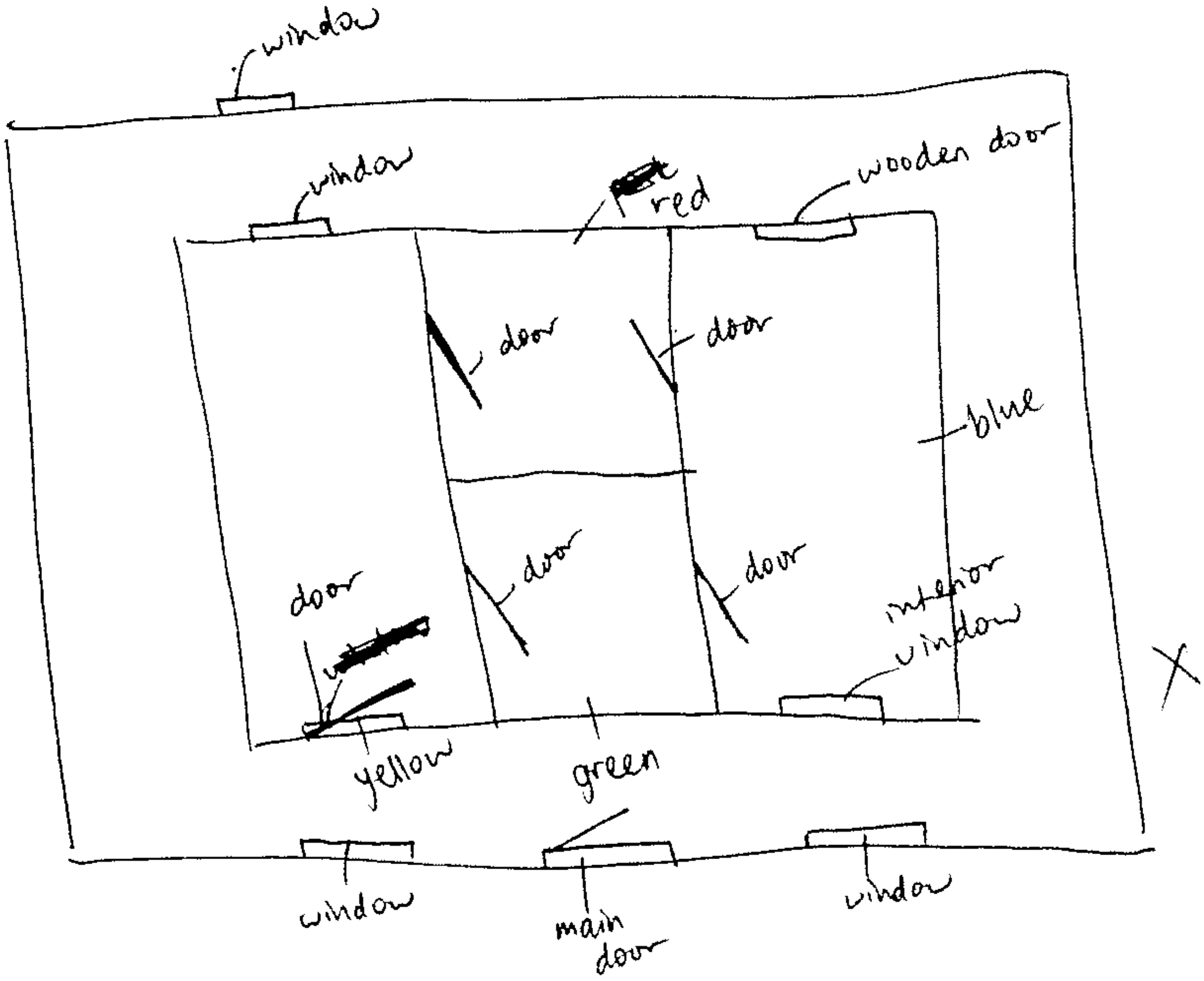

24 map 2 Mirror Video

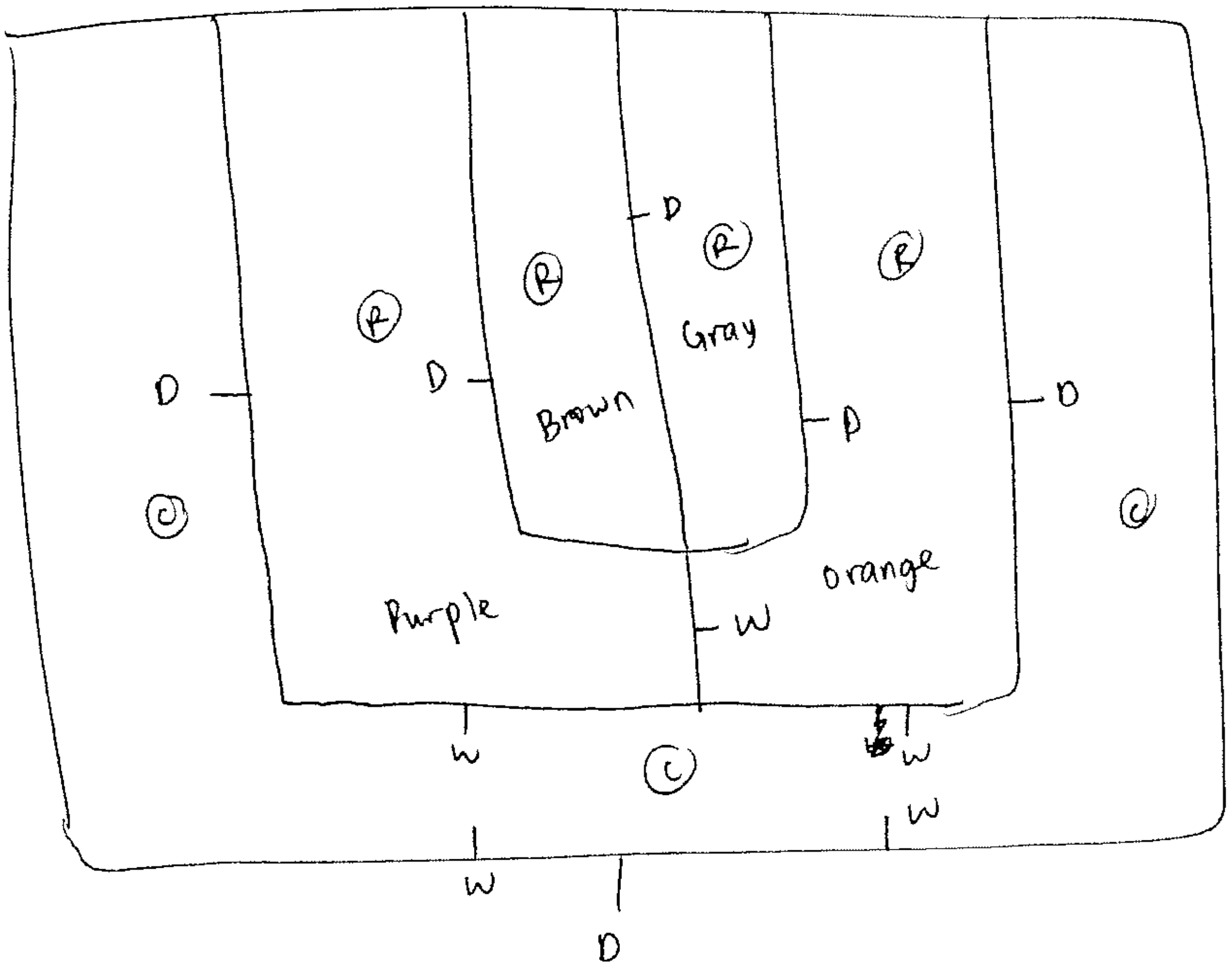

25 map 1 Rotational Spoken

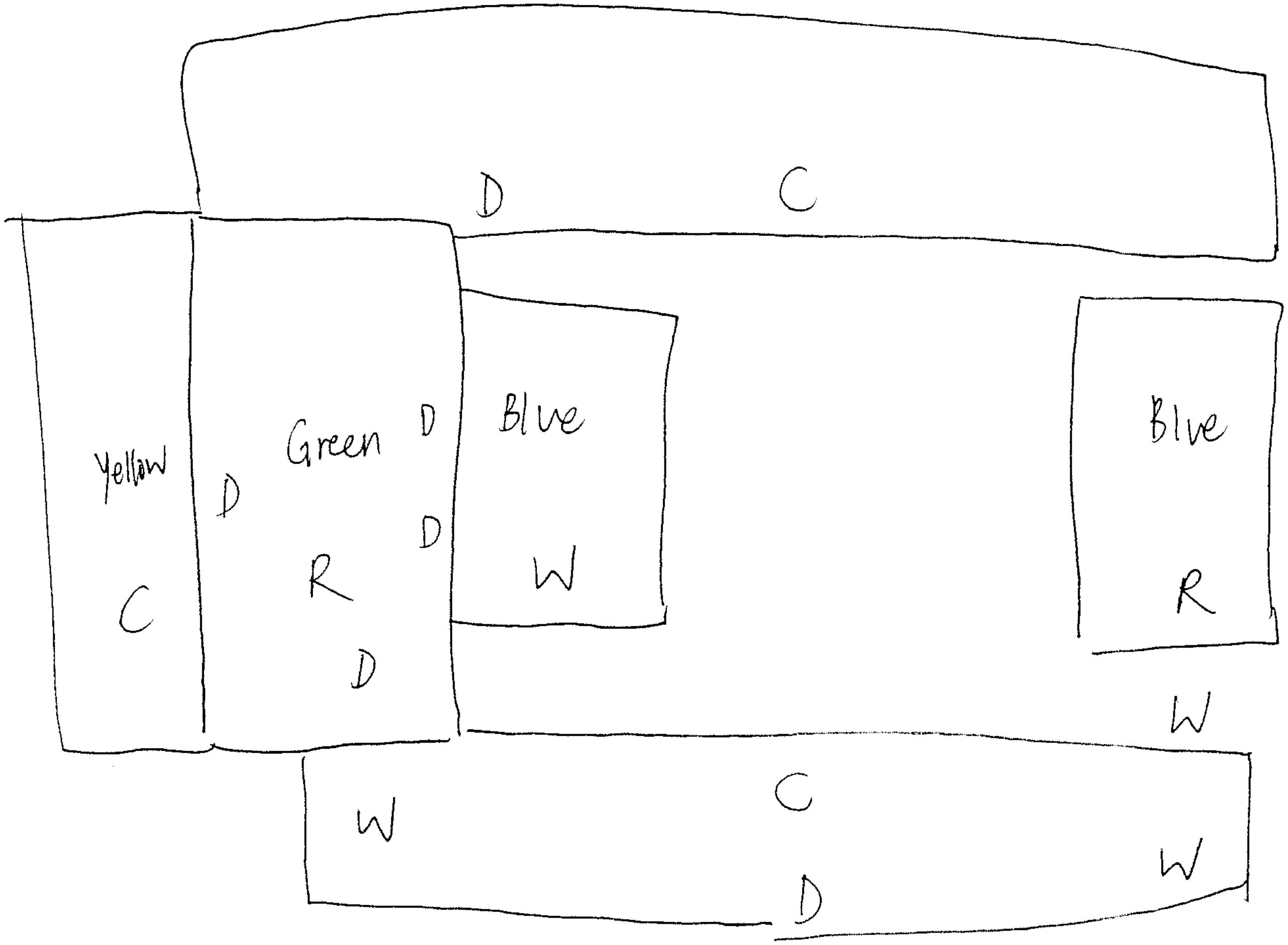

25 map 2 Mirror Video

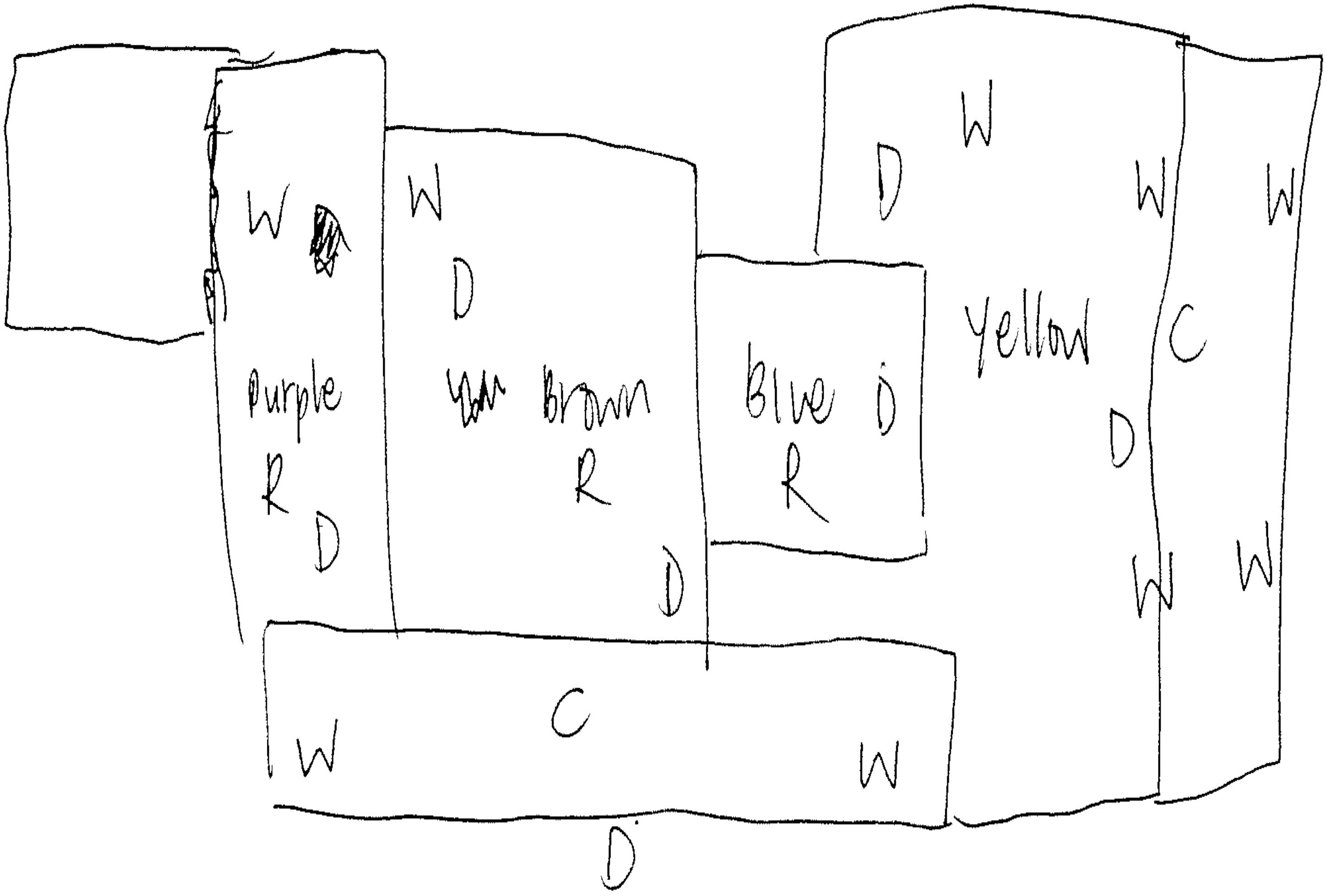

26 map 1 Rotational Spoken

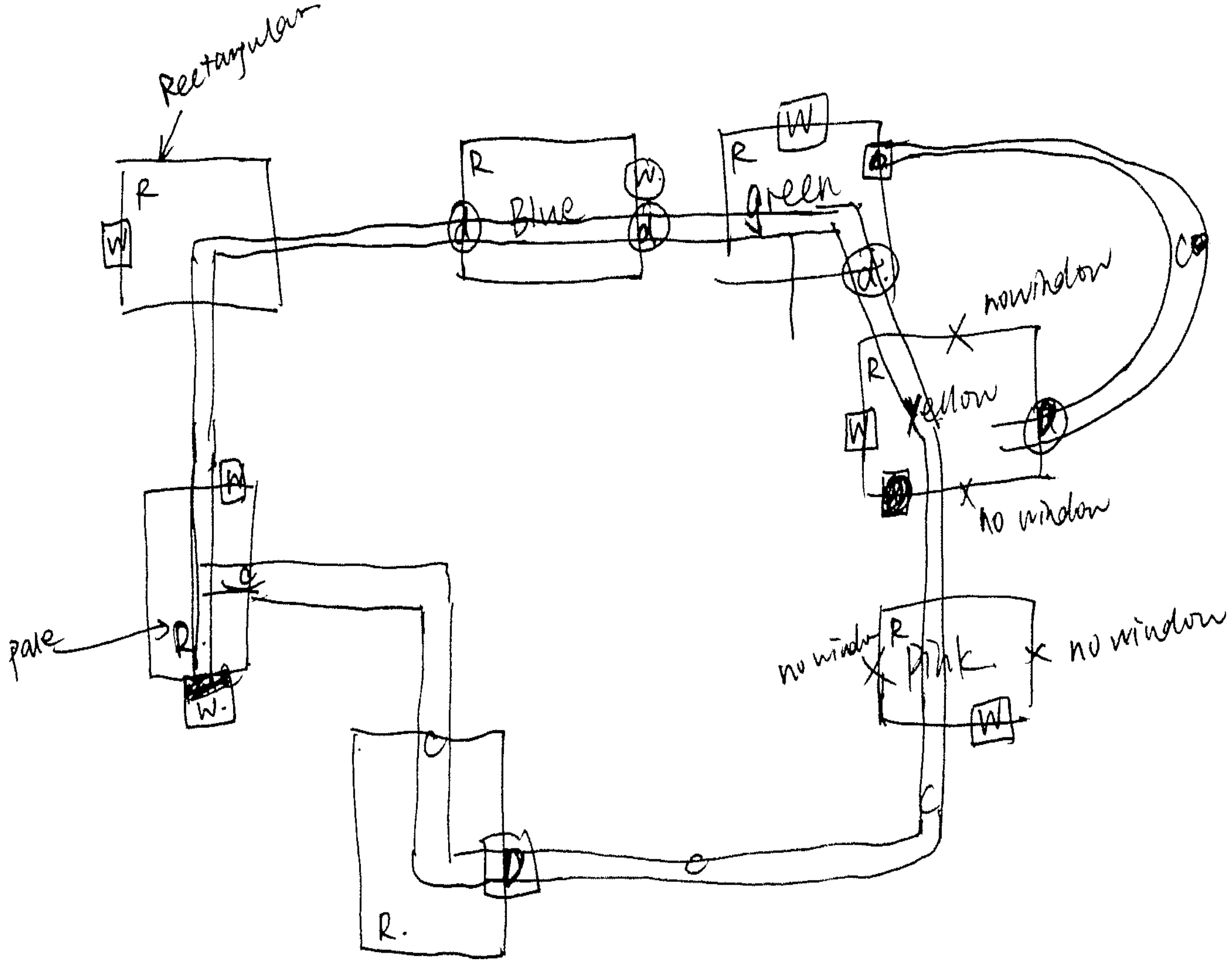

26 map 2 Mirror Video

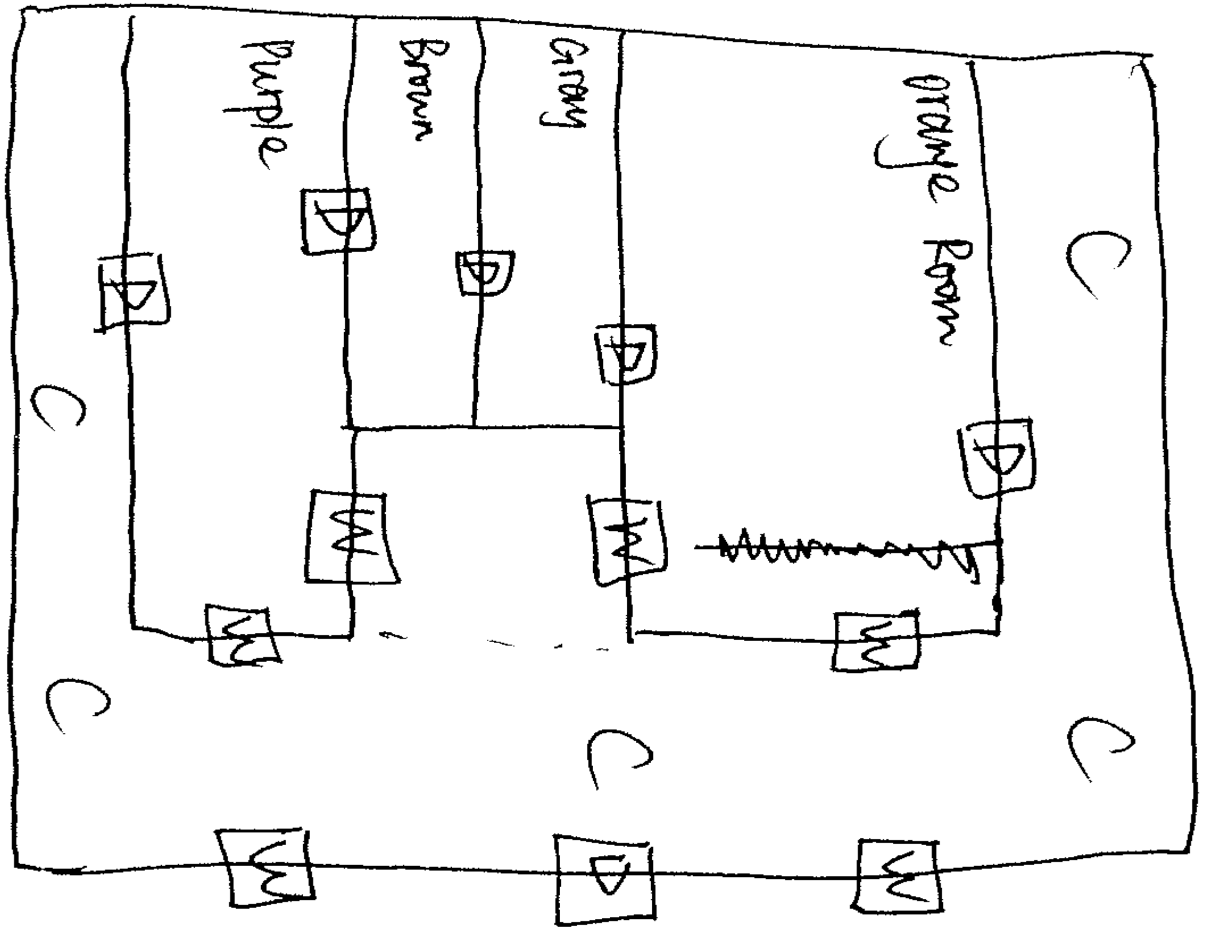

27 map 1 Mirror Video

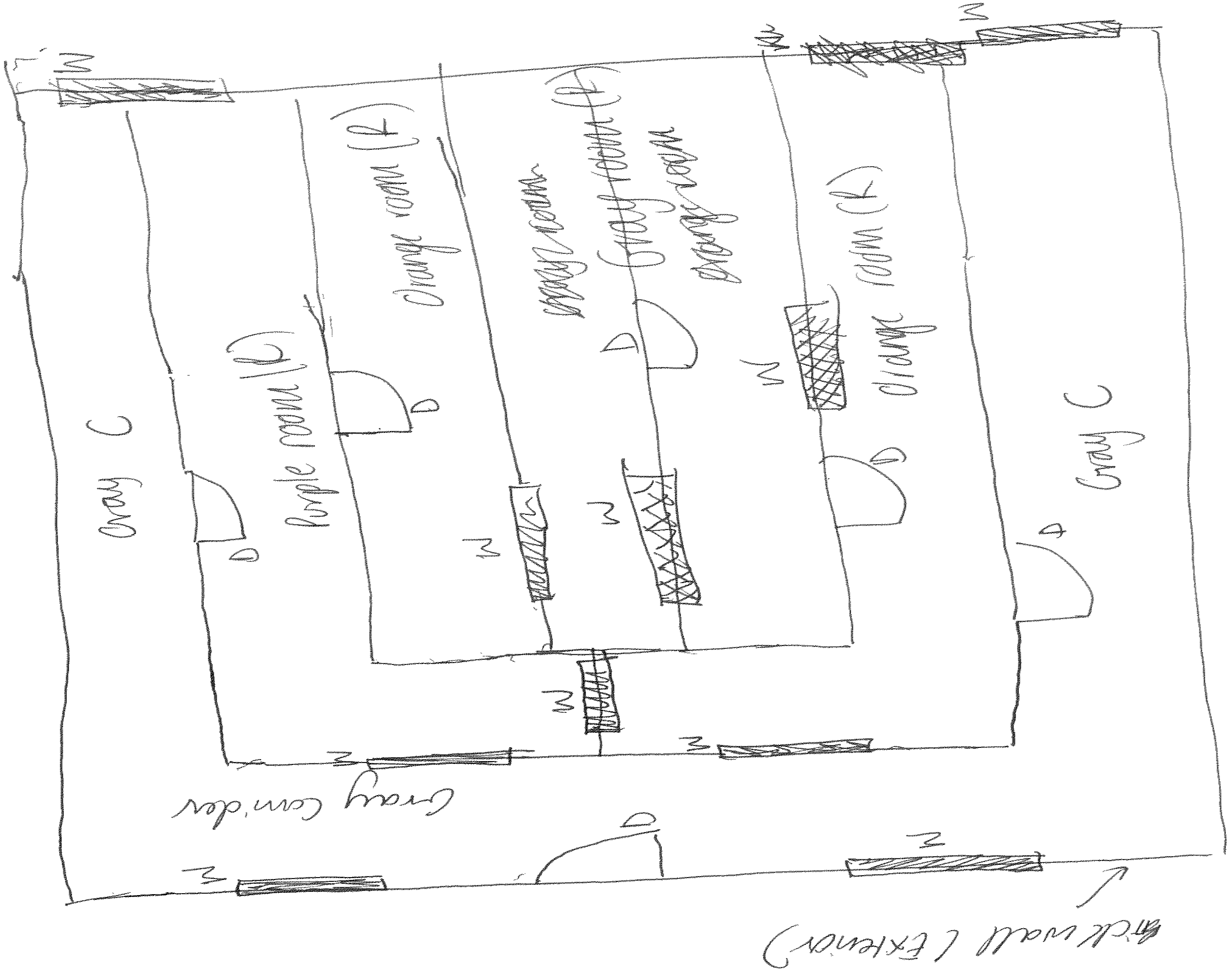

27 map 2 Rotational Spoken

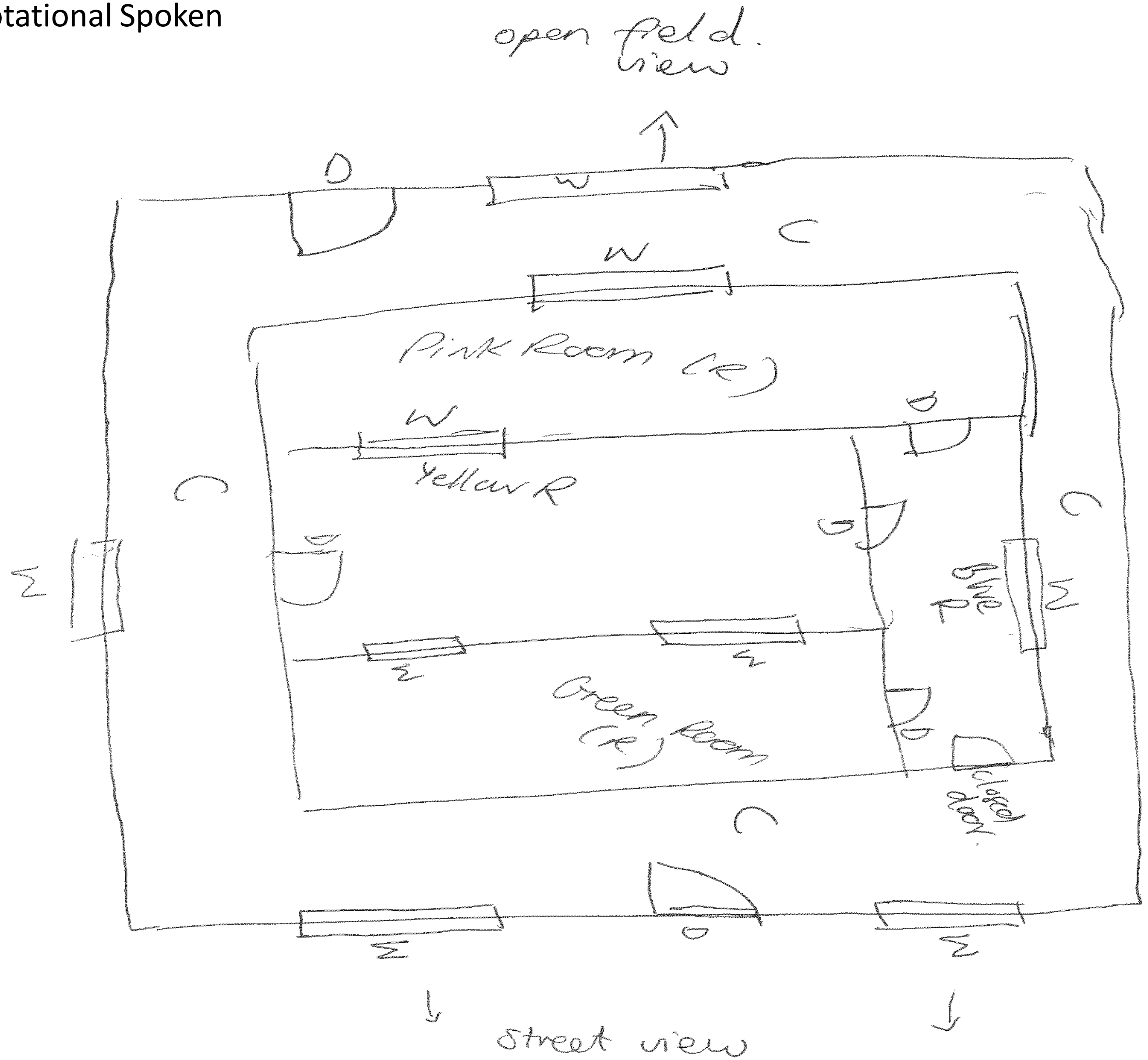

28 map 1 Rotational Spoken

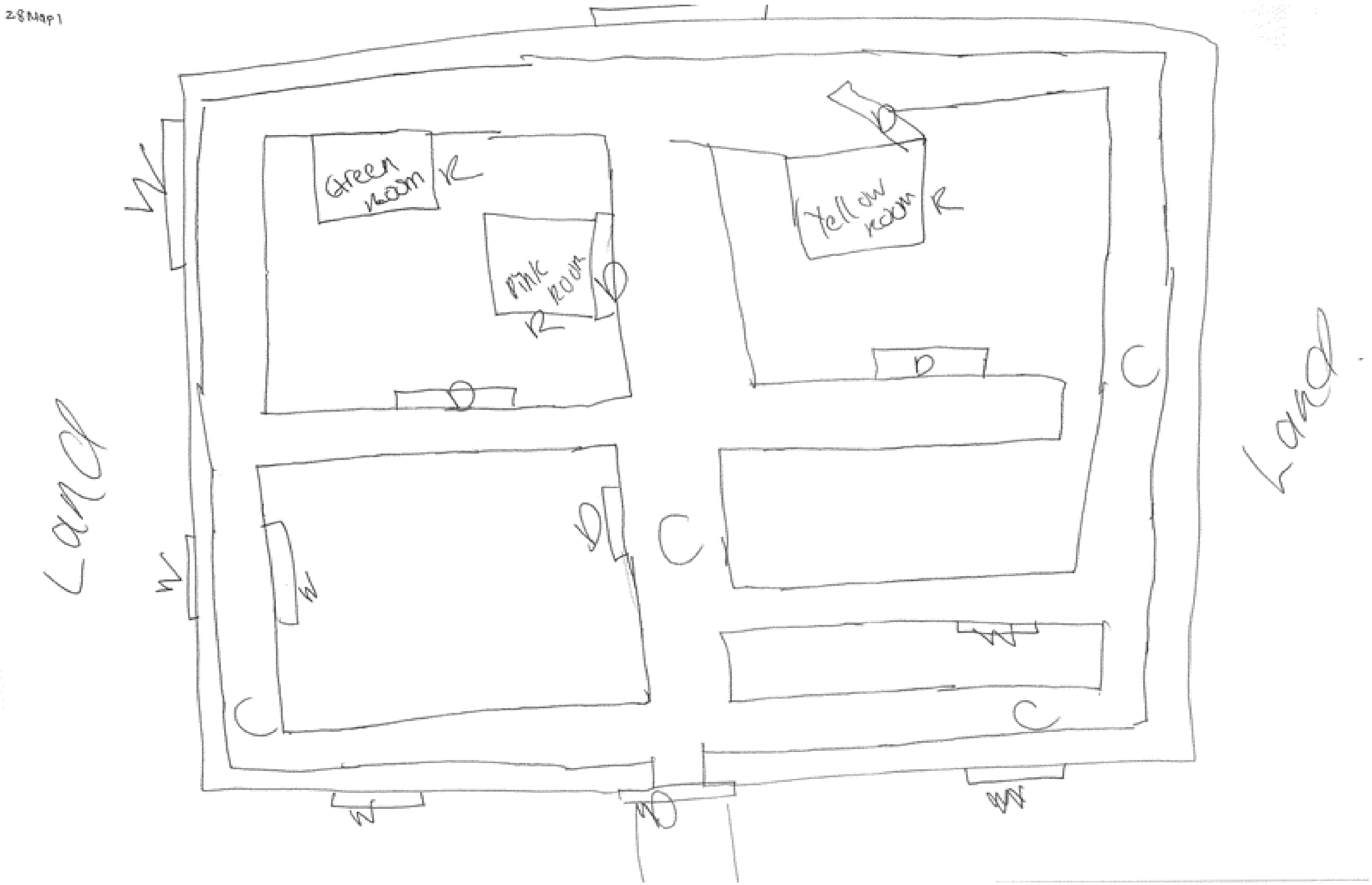

28 map 2 Mirror Video

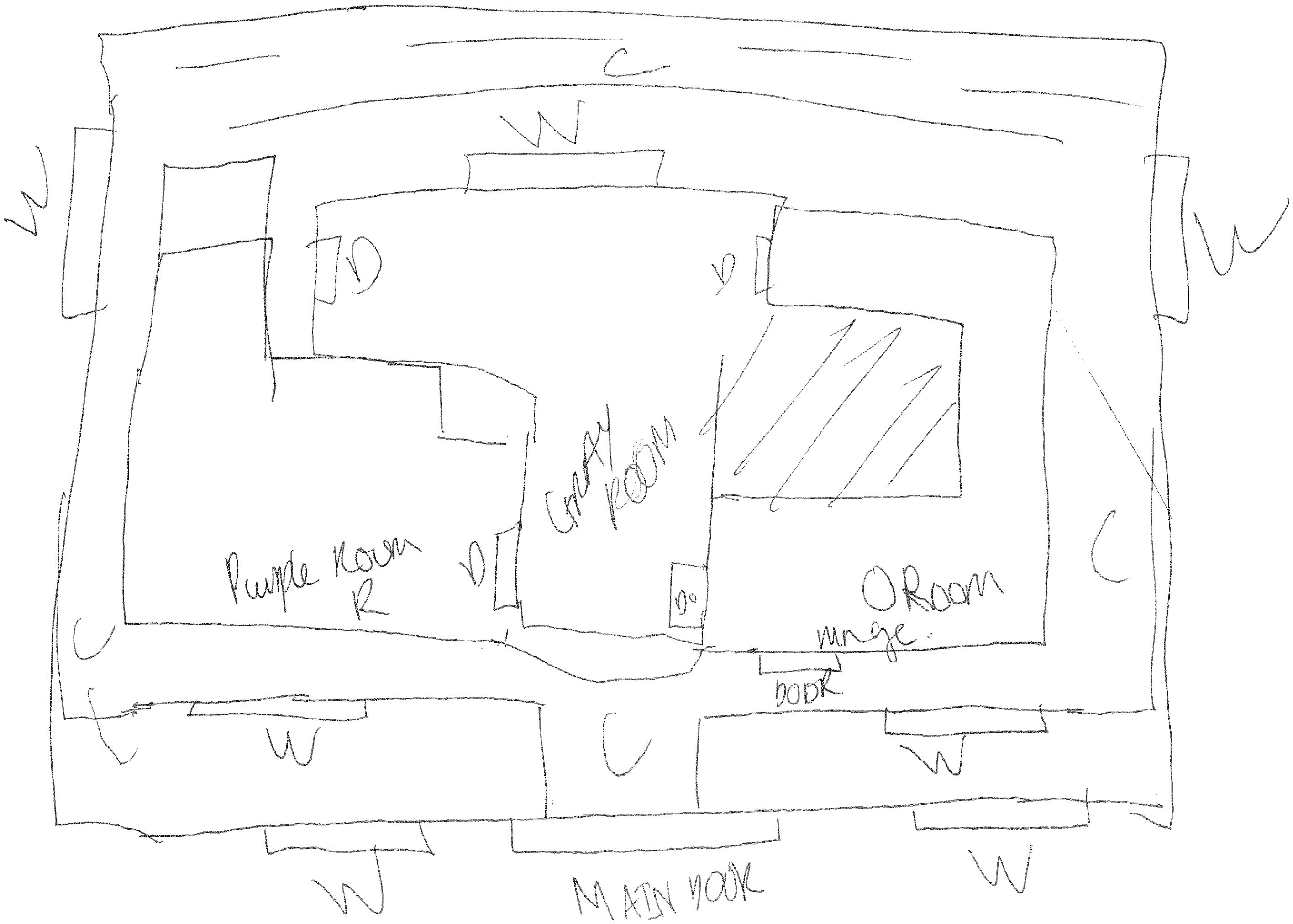

29 map 1 Mirror Video

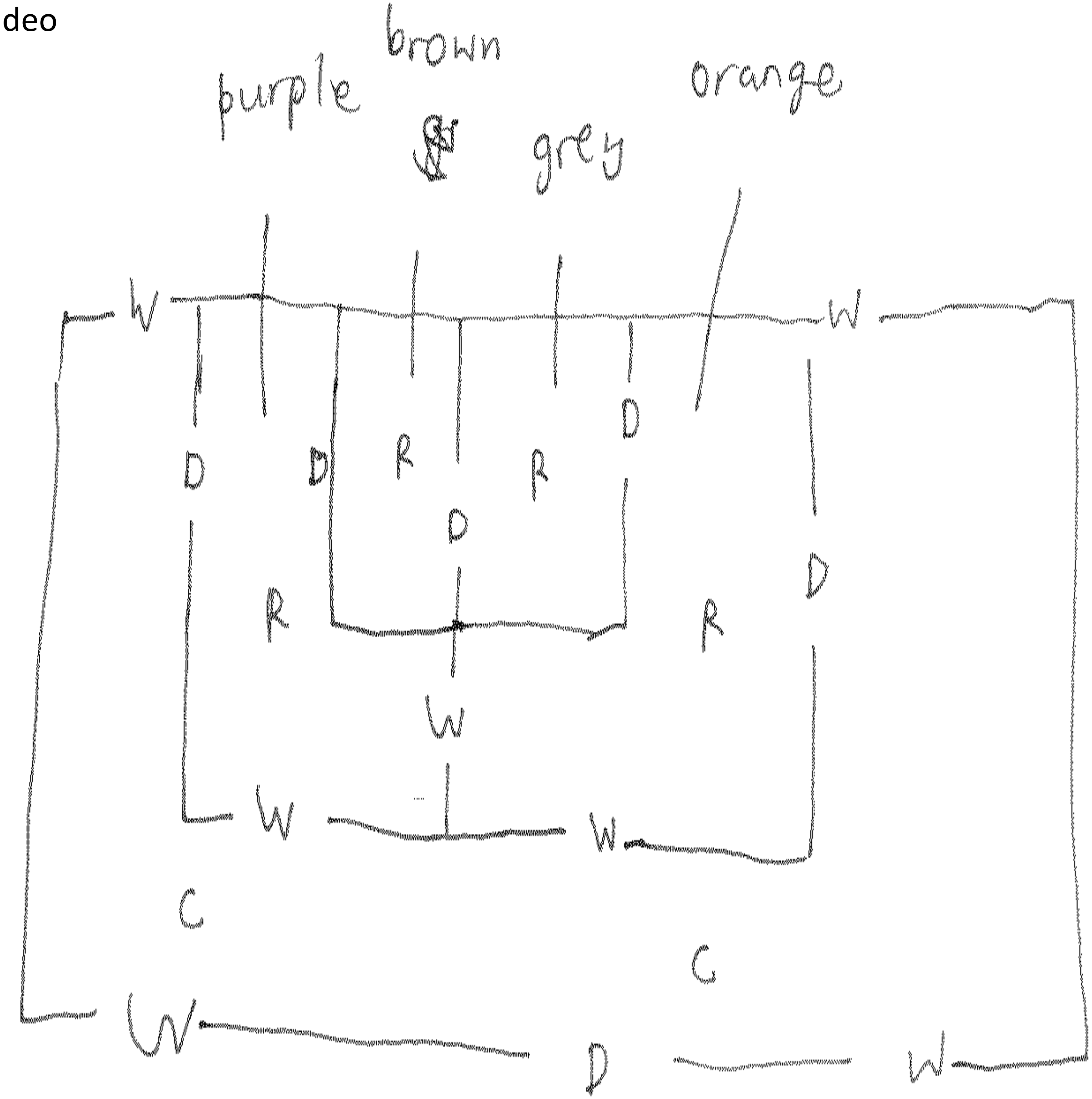

29 map 2 Rotational Spoken

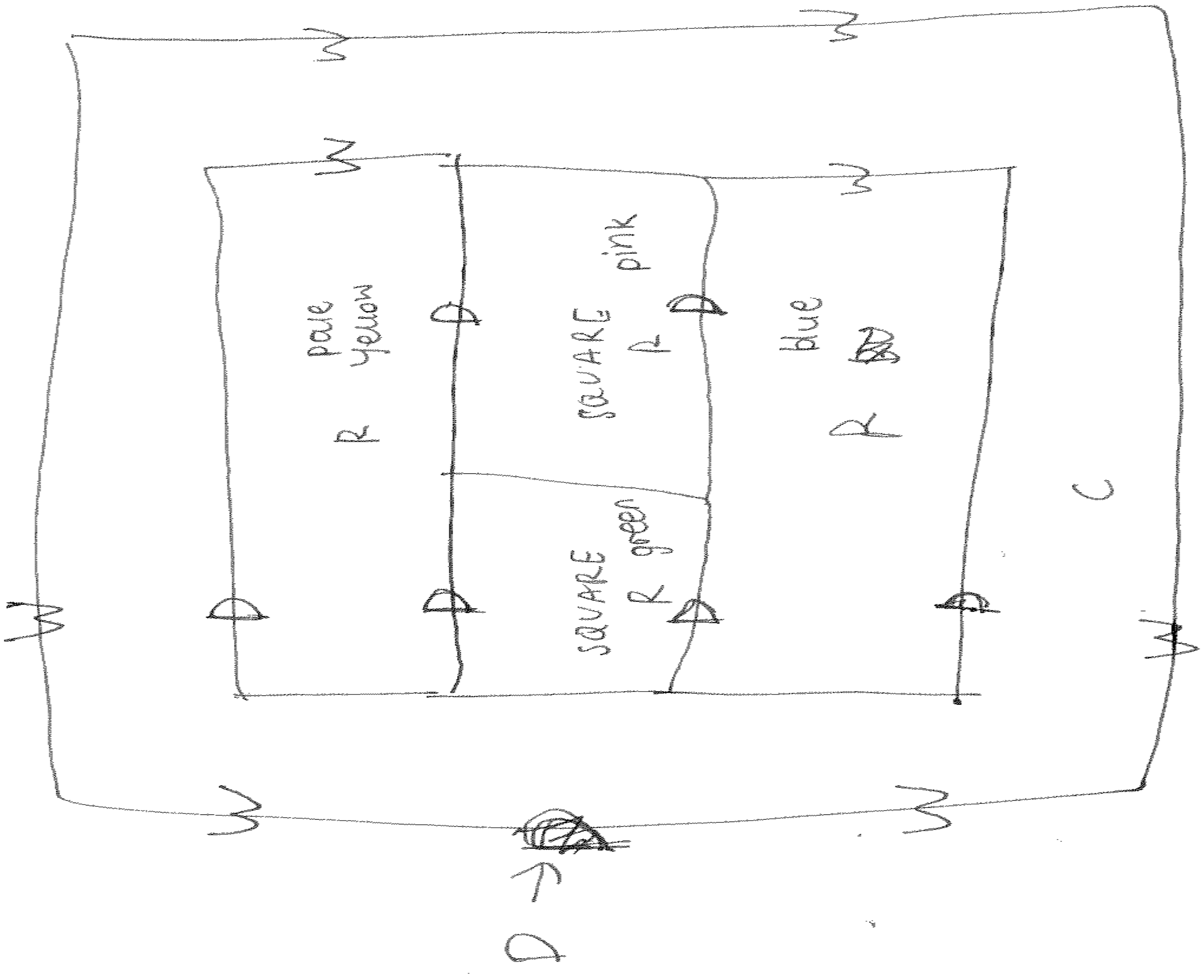

30 map 1 Rotational Spoken

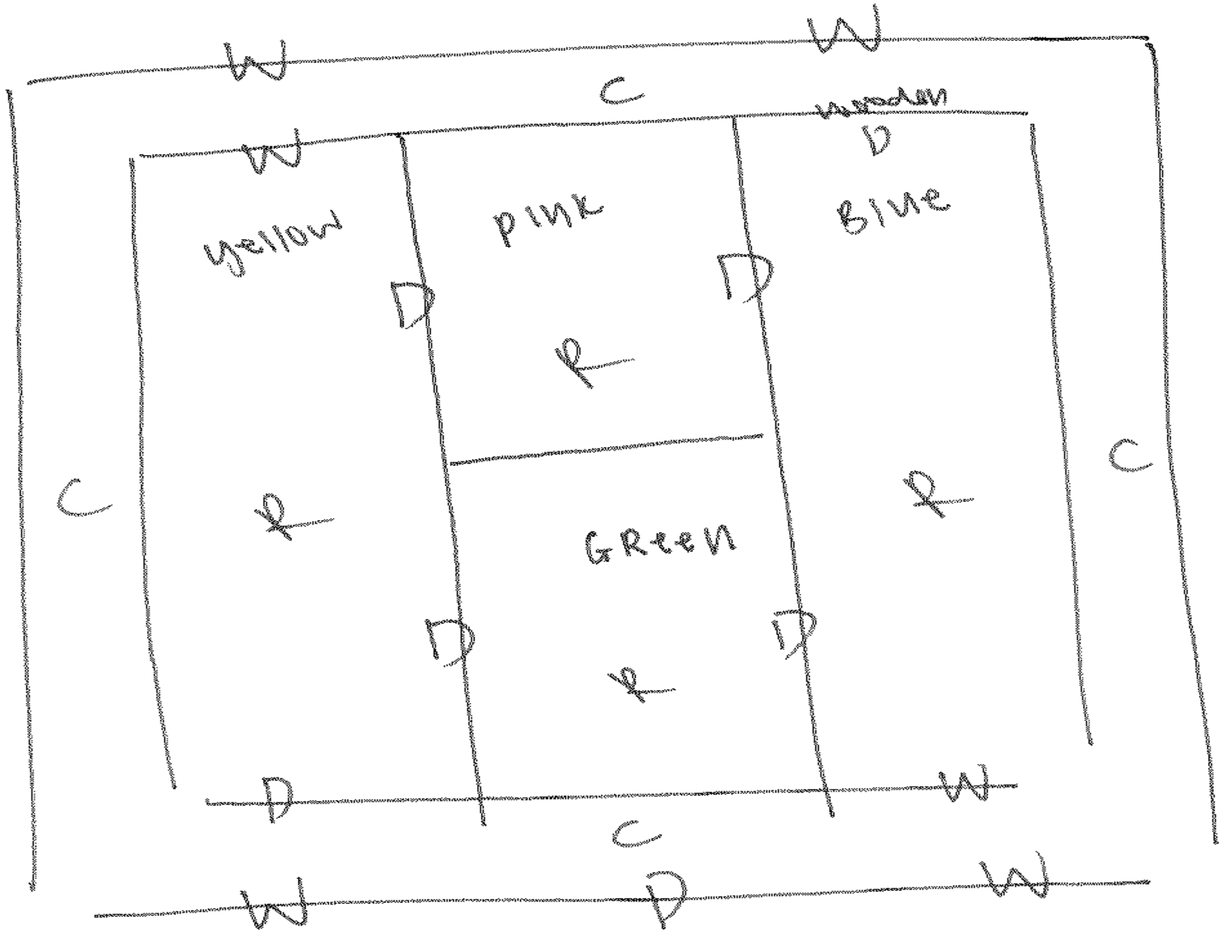

30 map 2 Mirror Video

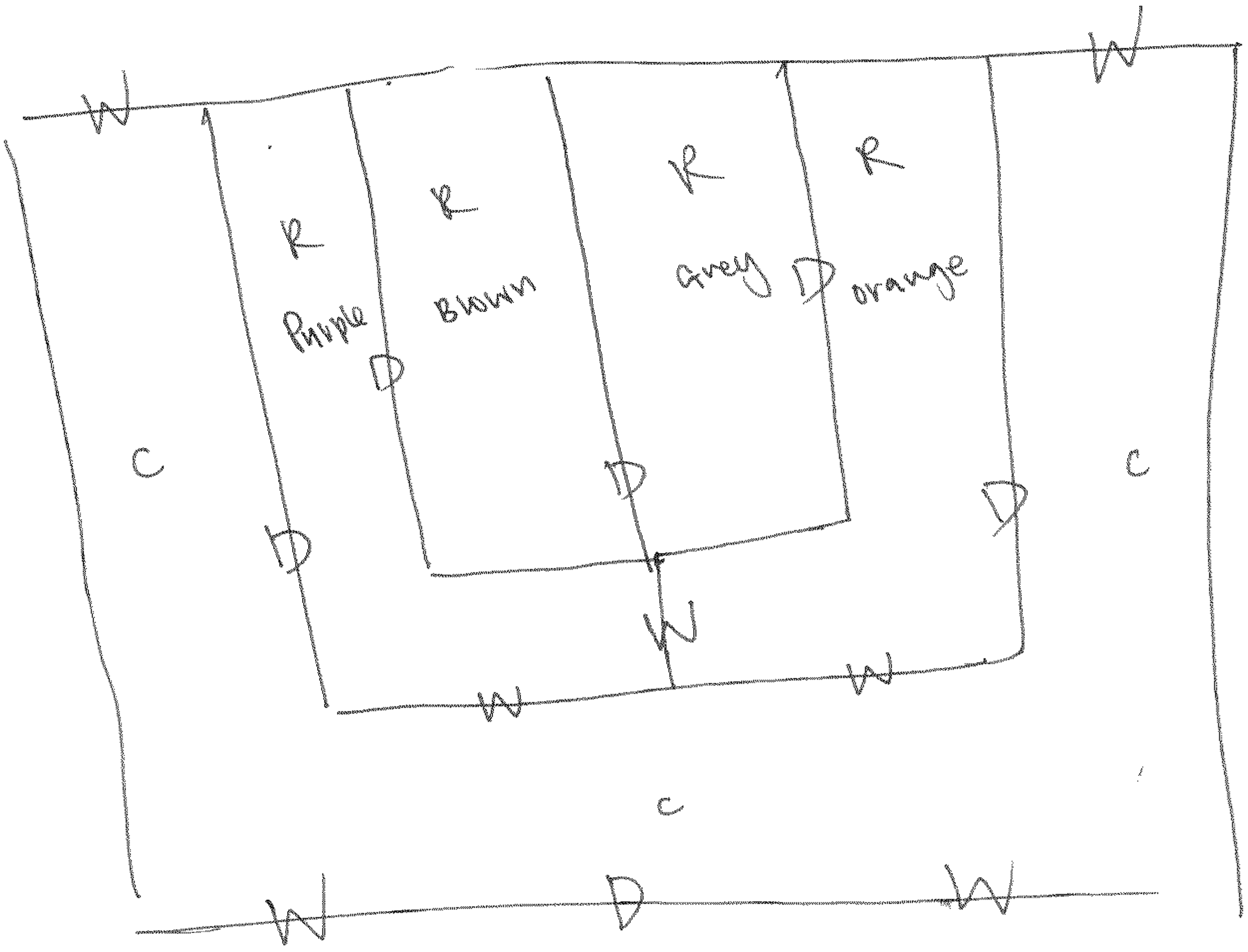

31 map 1 Mirror Video

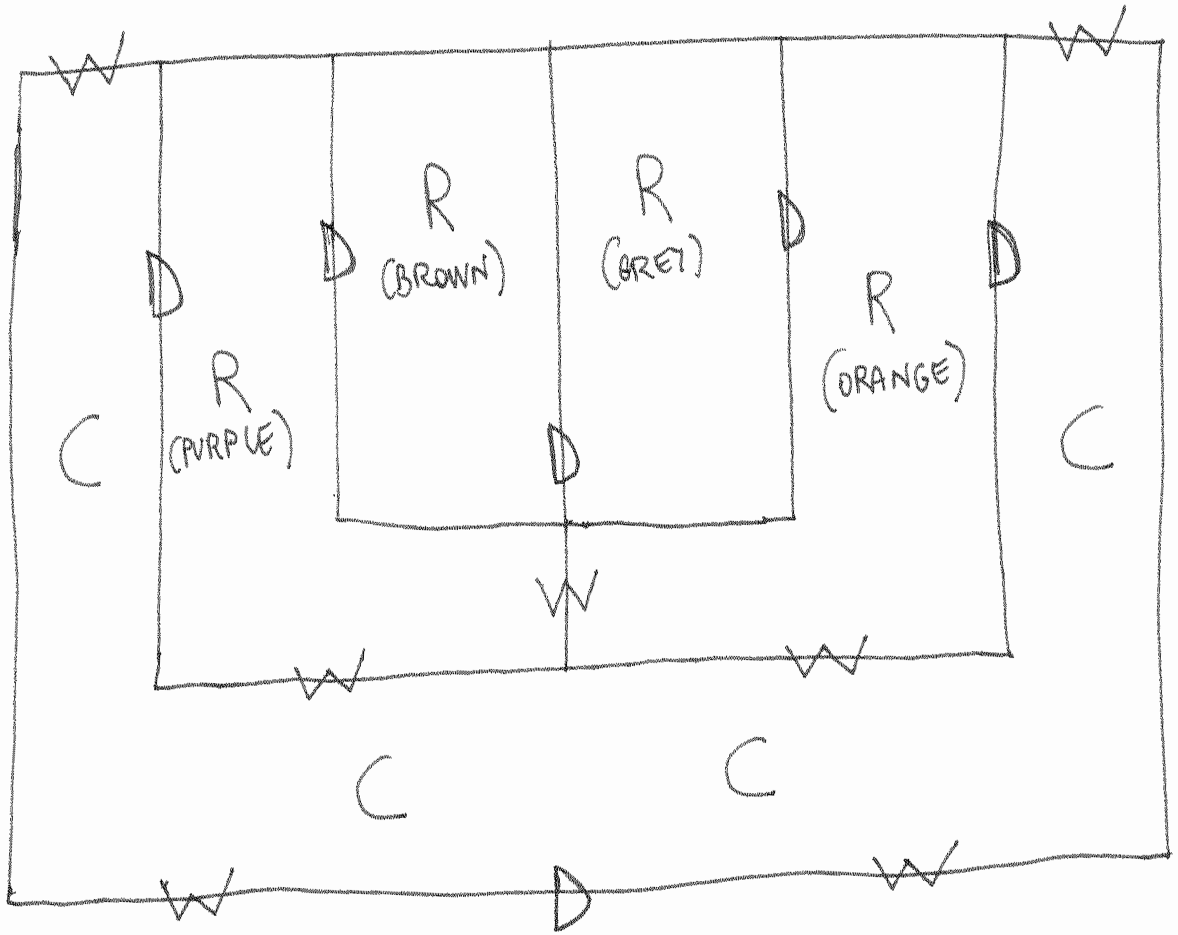

31 map 2 Rotational Spoken

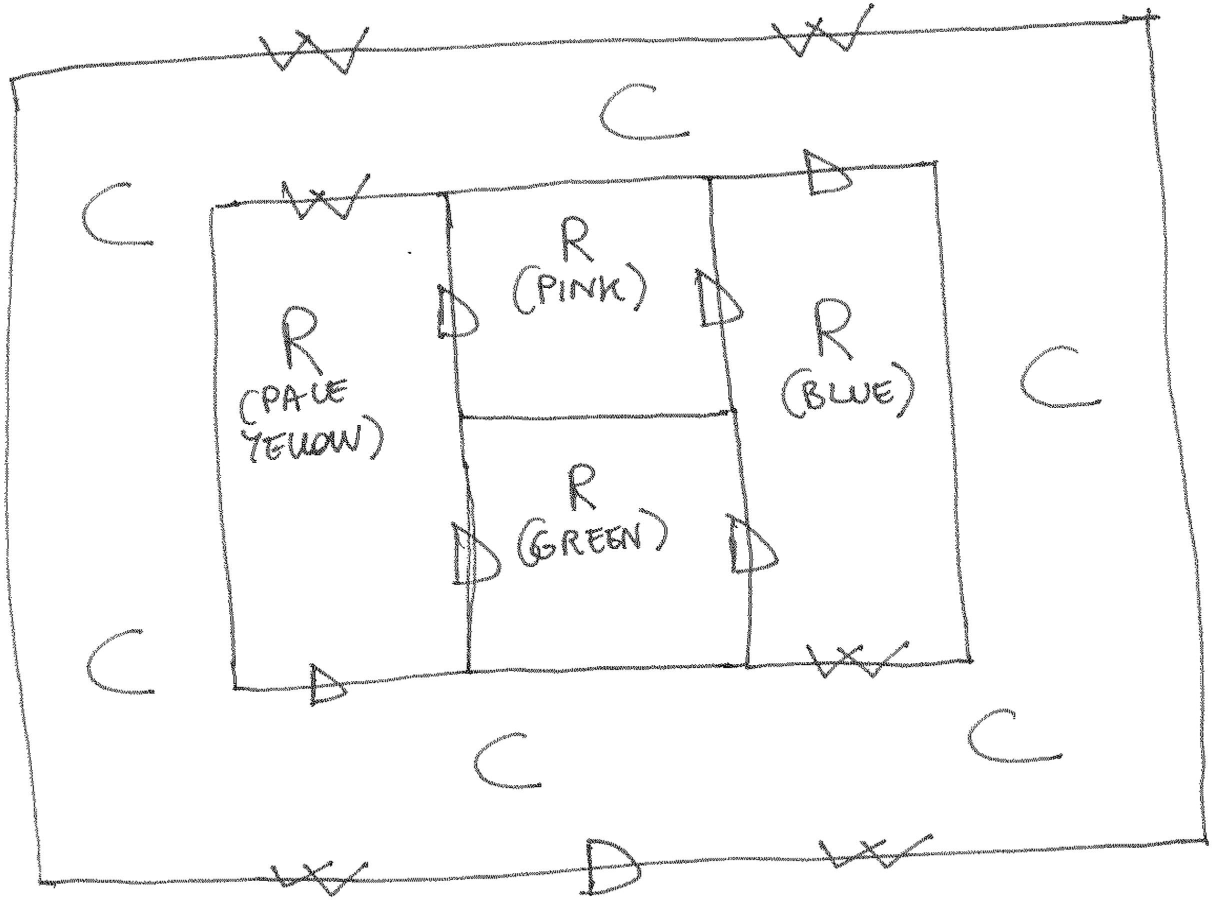

32 map 1 Rotational Spoken

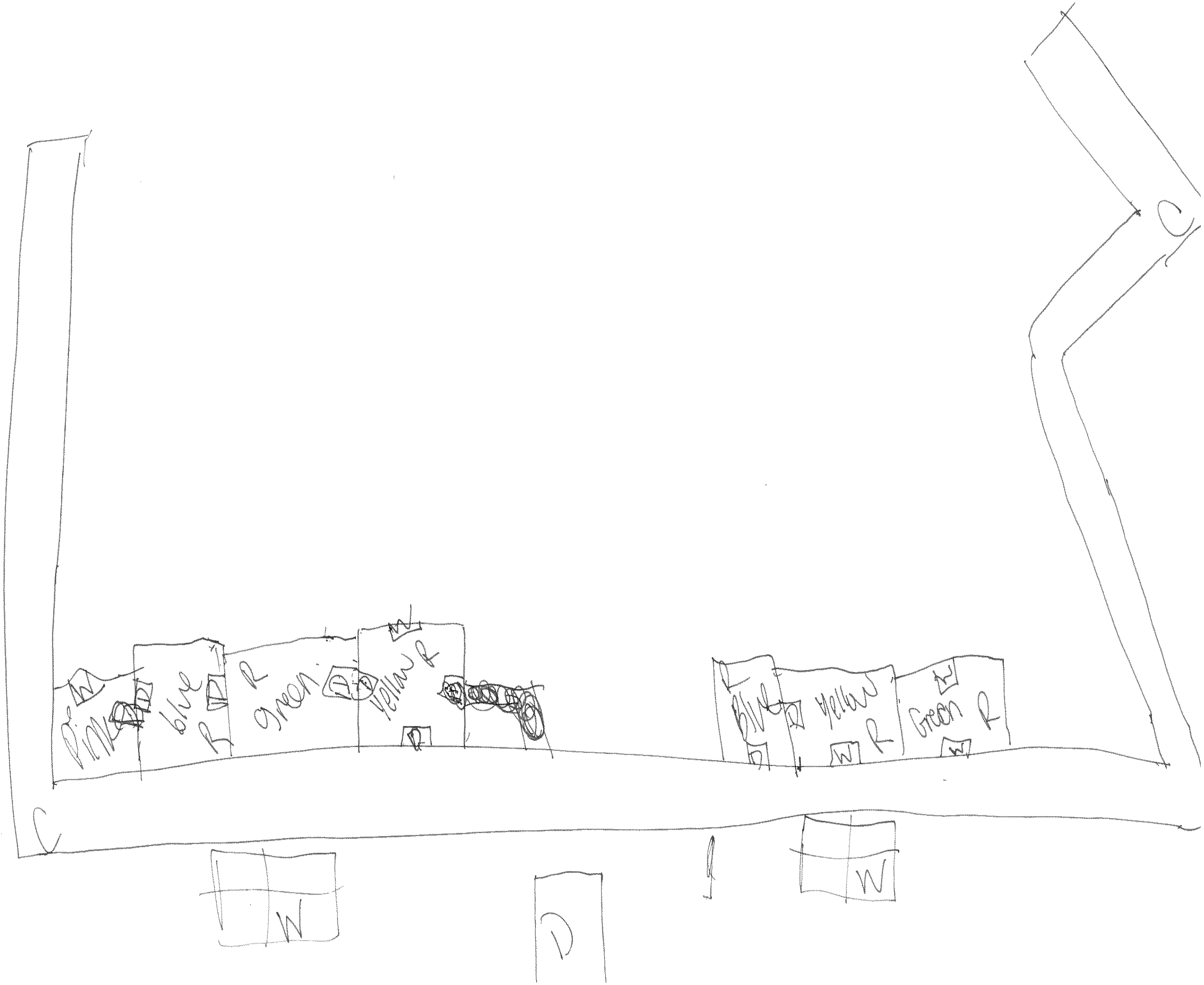

32 map 2 Mirror Video

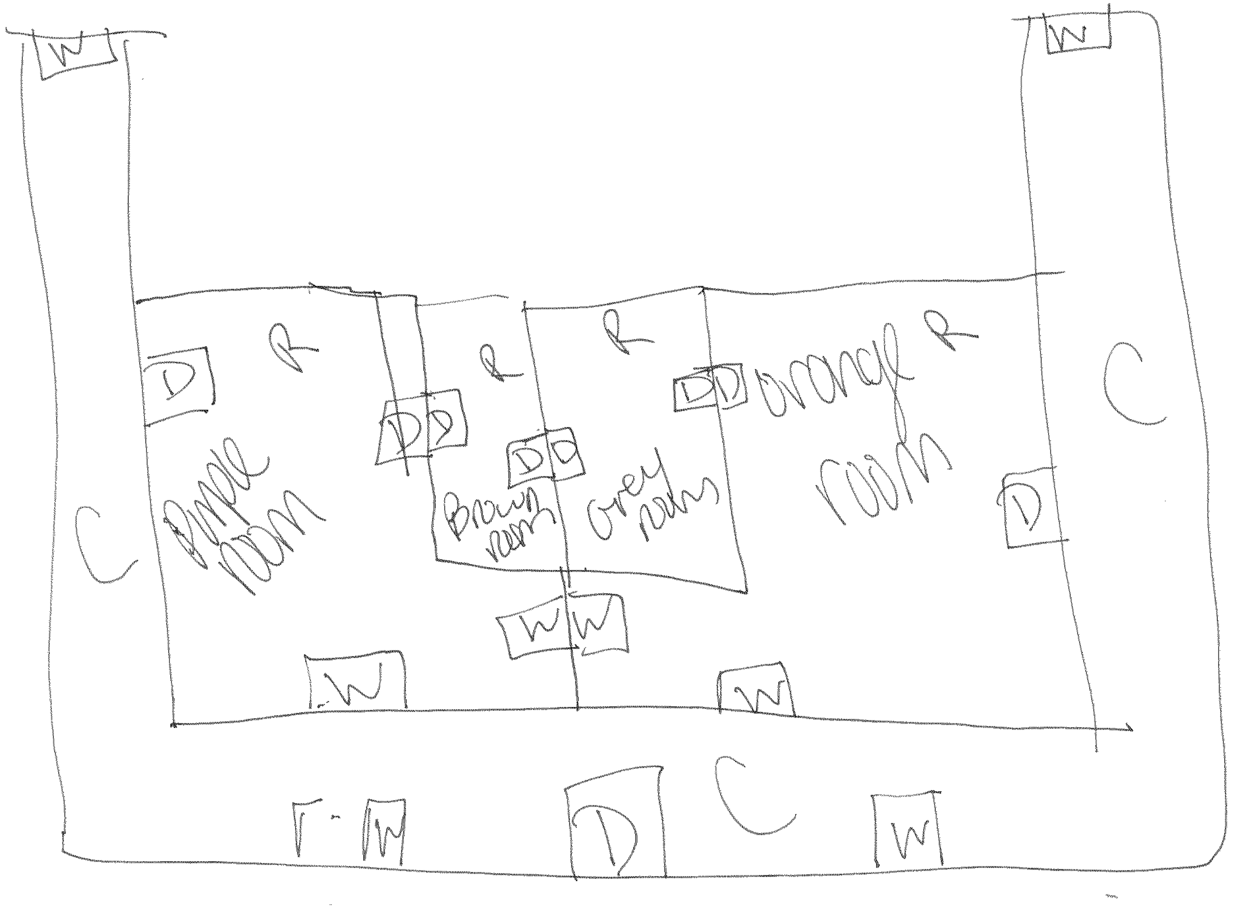

33 map 1 Rotational Spoken

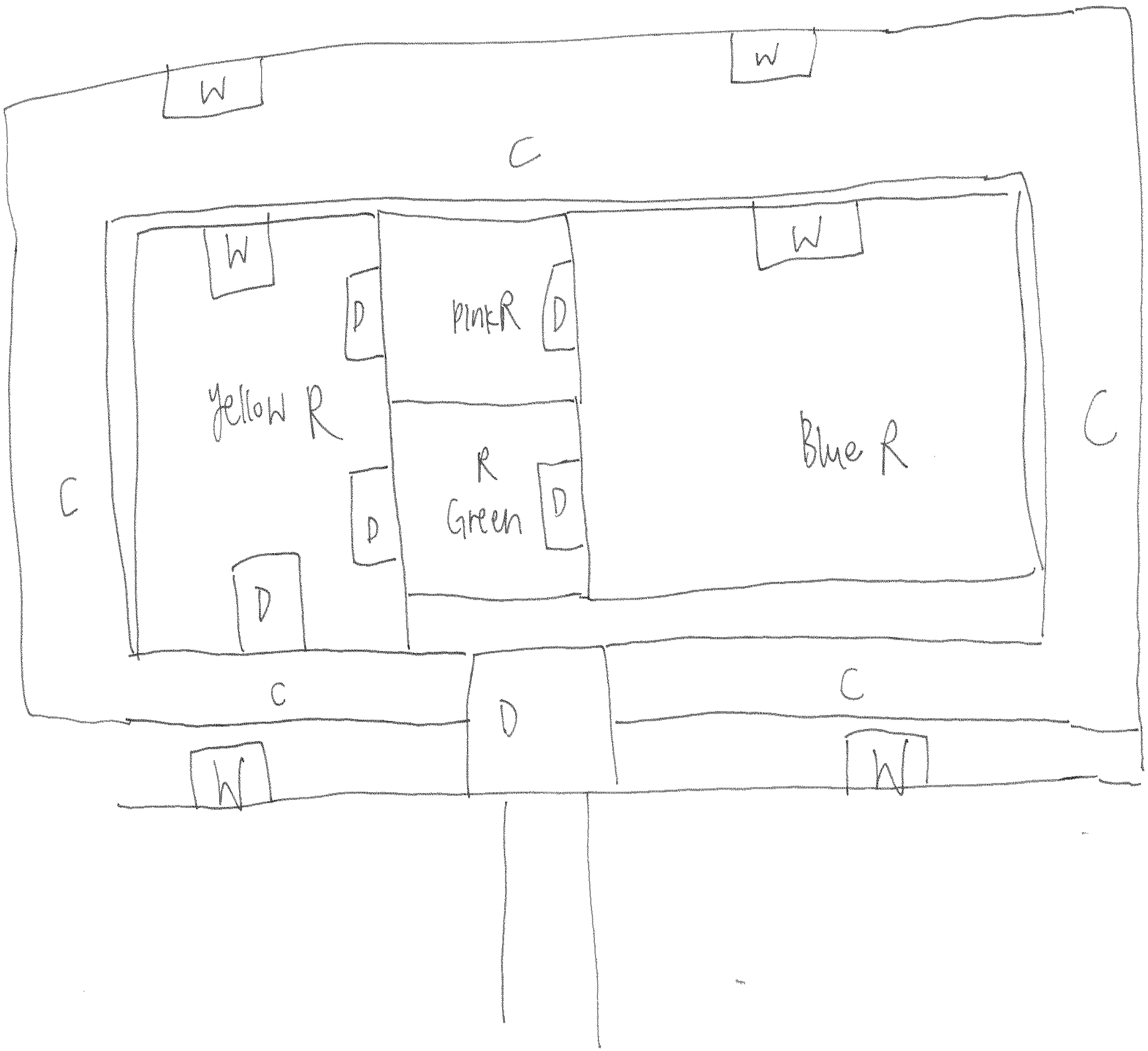

33 map 2 Mirror Video

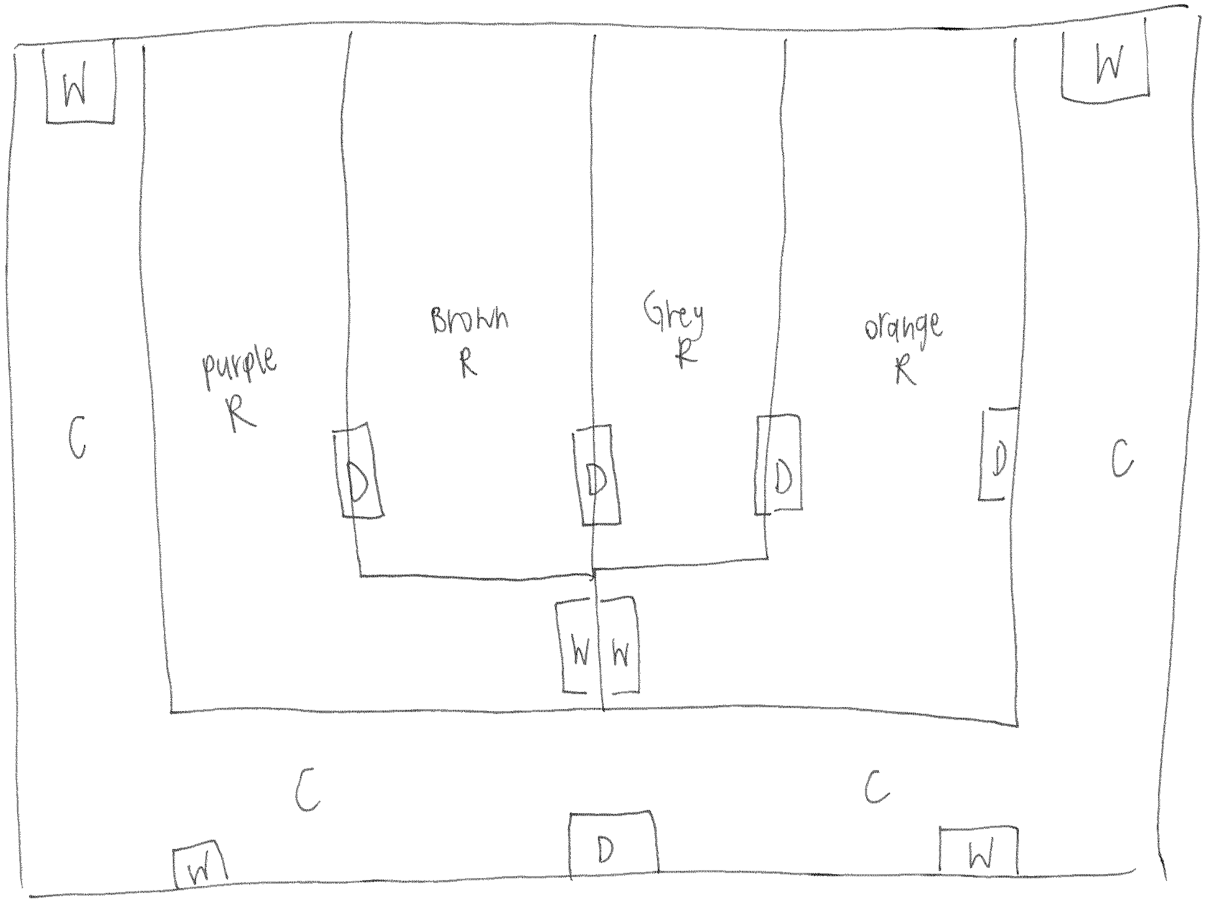

34 map 1 Rotational Video

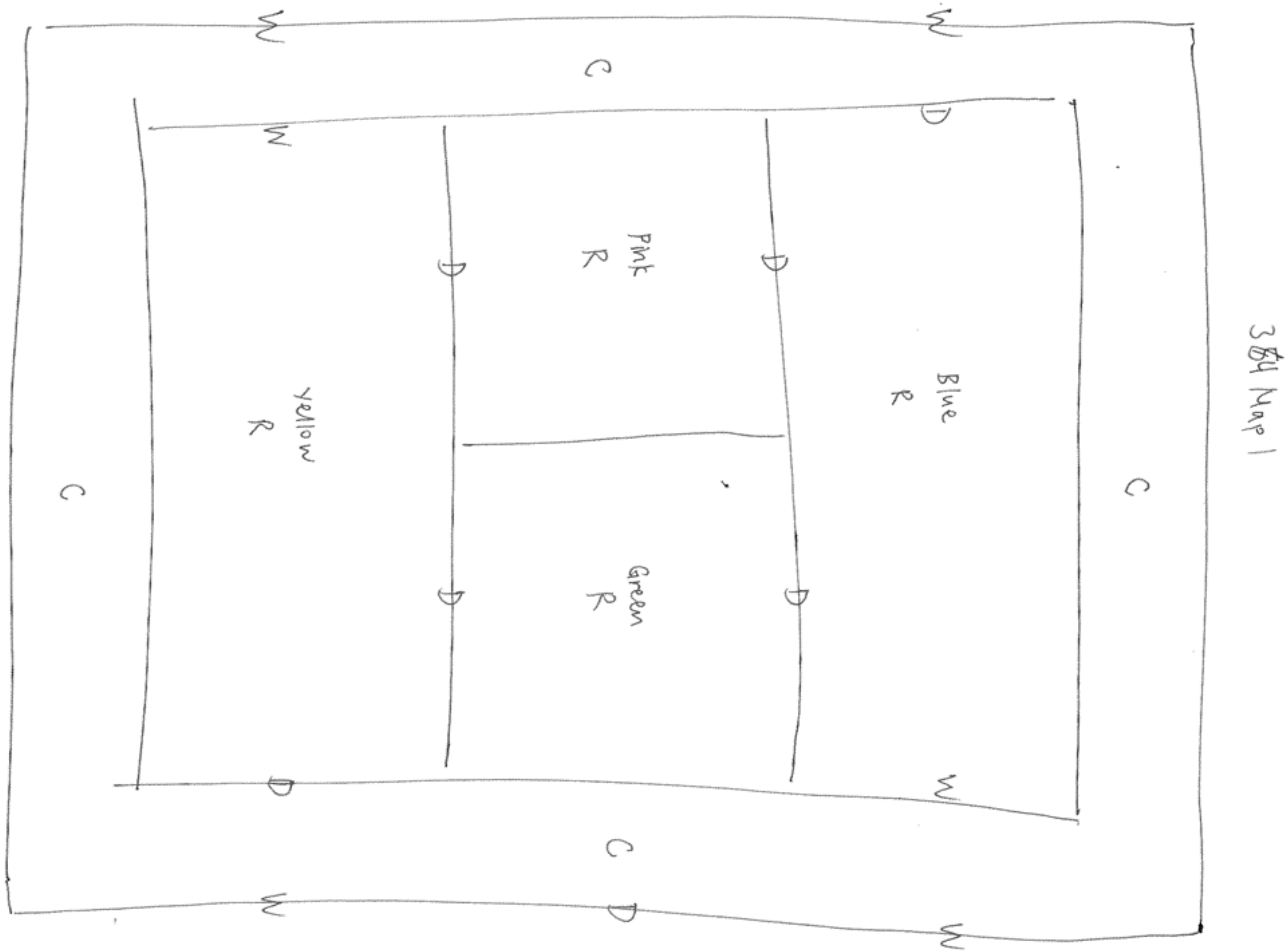

34 map 2 Mirror Spoken

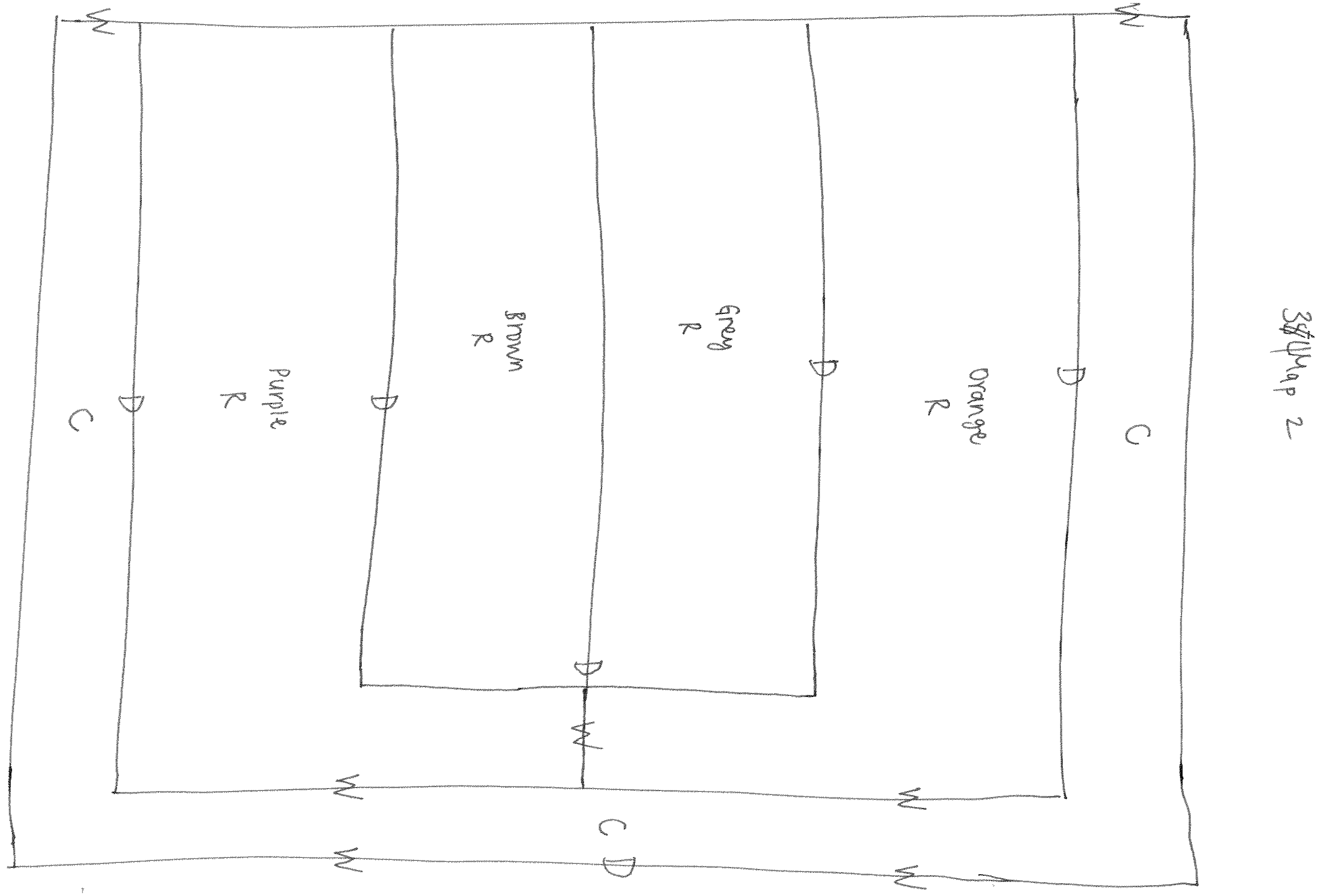

35 map 1 Mirror Video

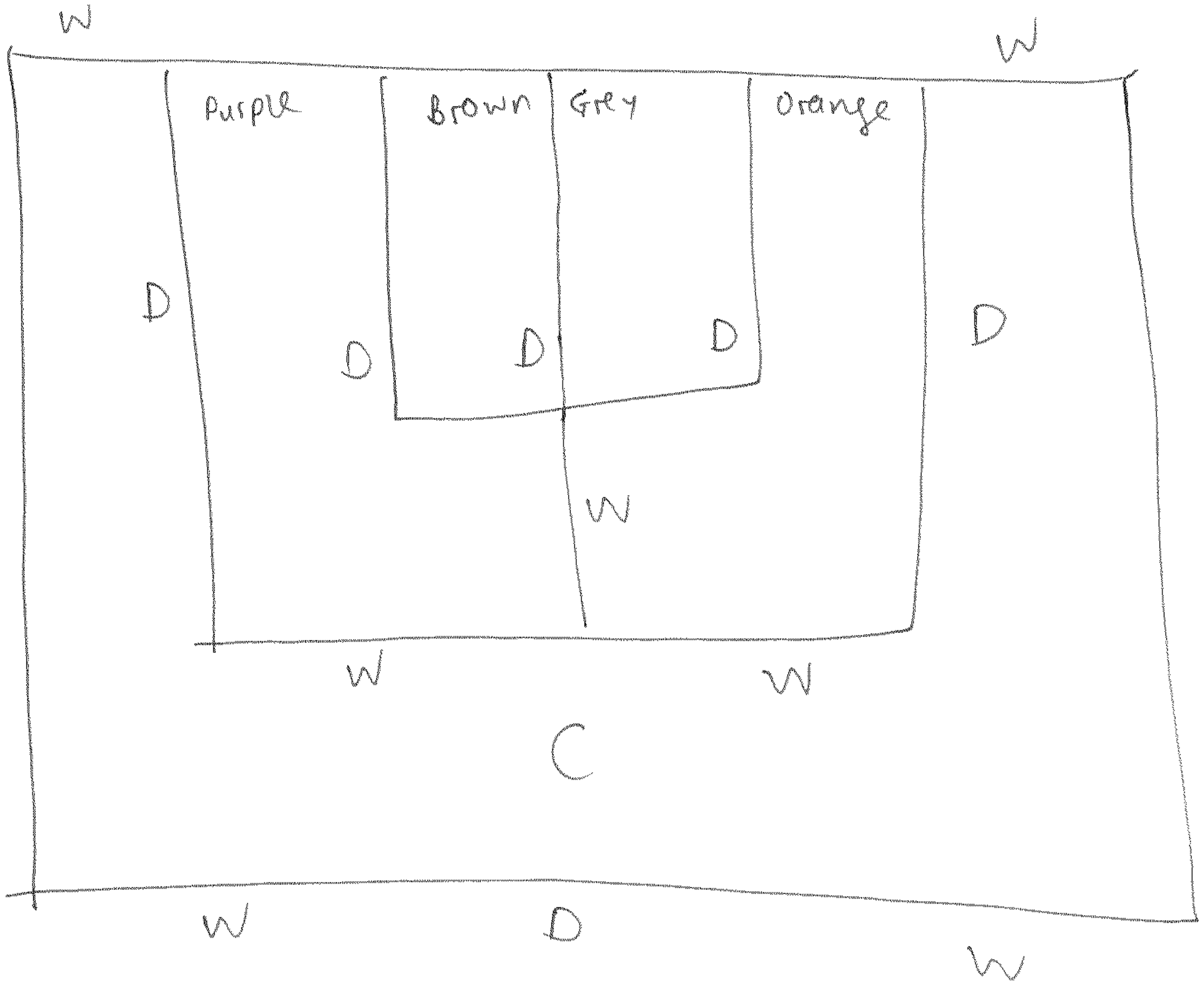

35 map 2 Rotational Spoken

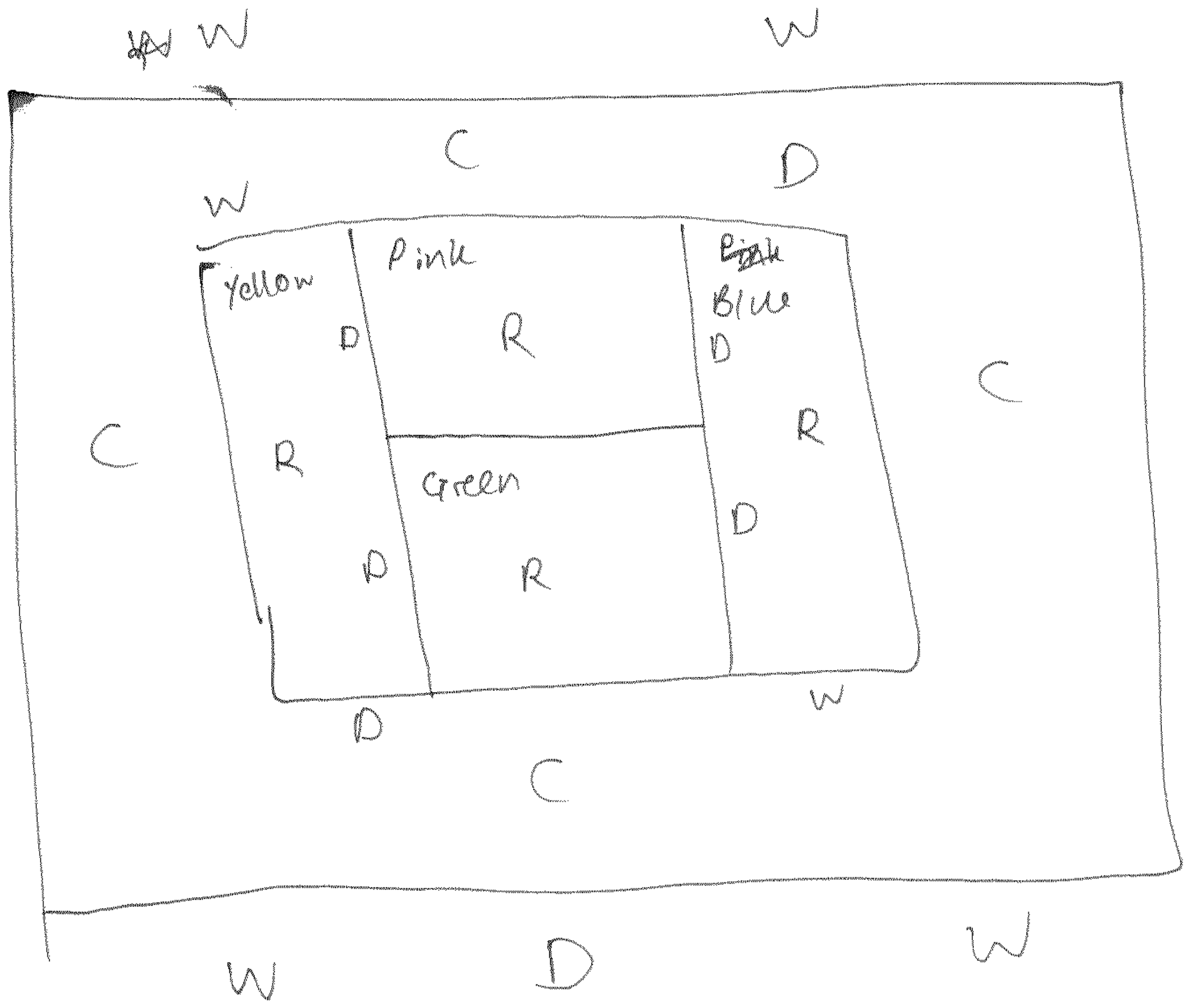

36 map1 Rotational Spoken

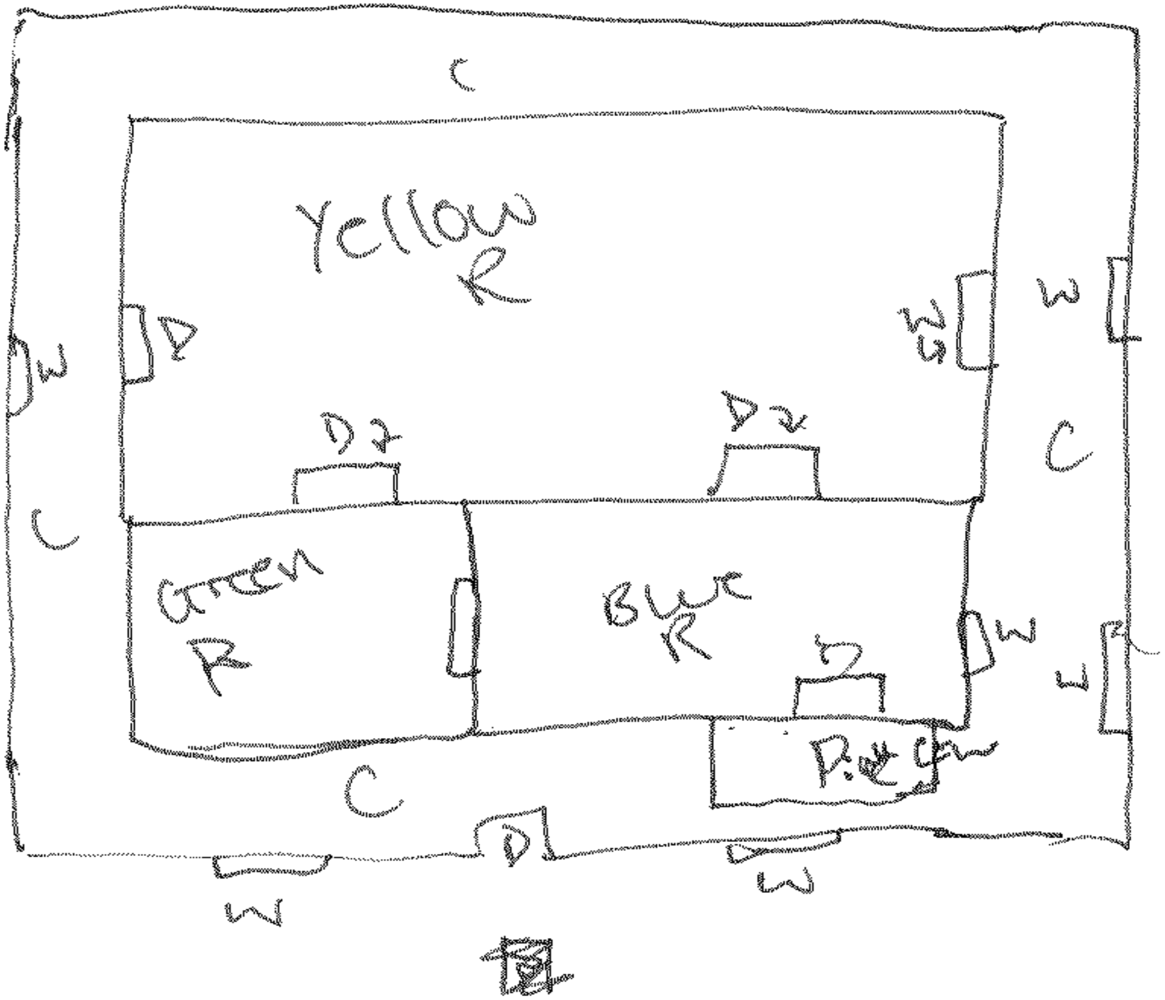

36 map 2 Mirror Video

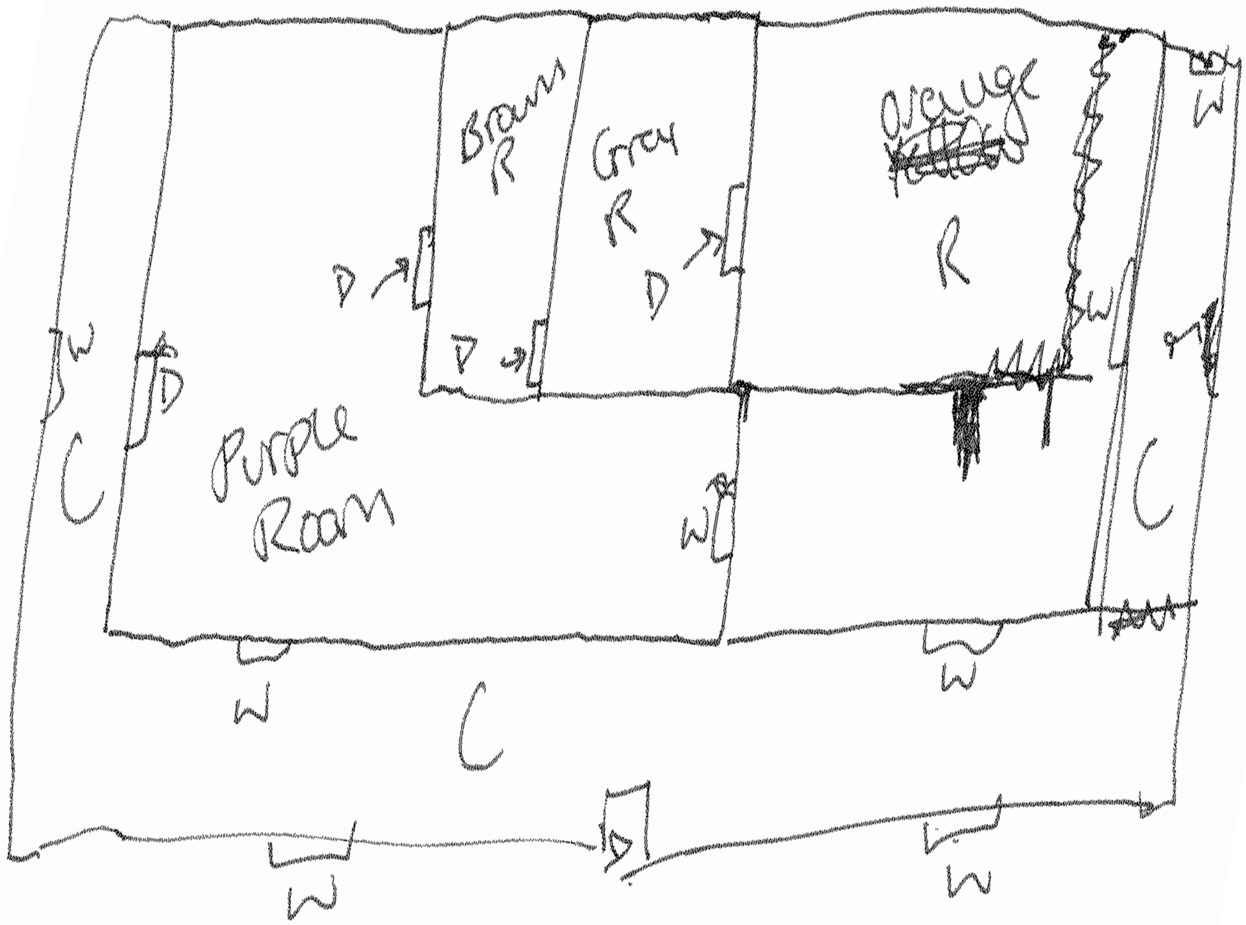

37 map 1 Rotational Video

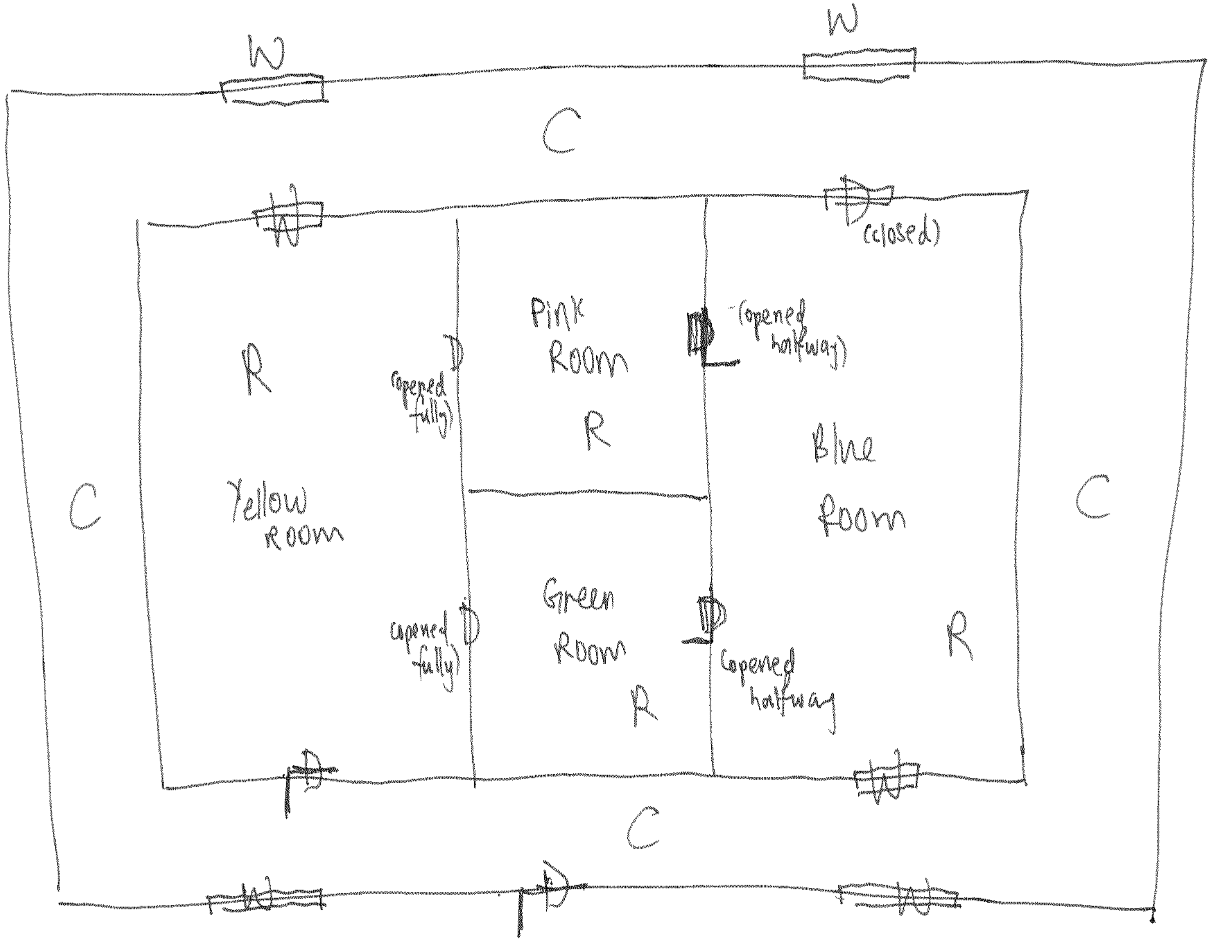

37 map 2 Mirror Spoken

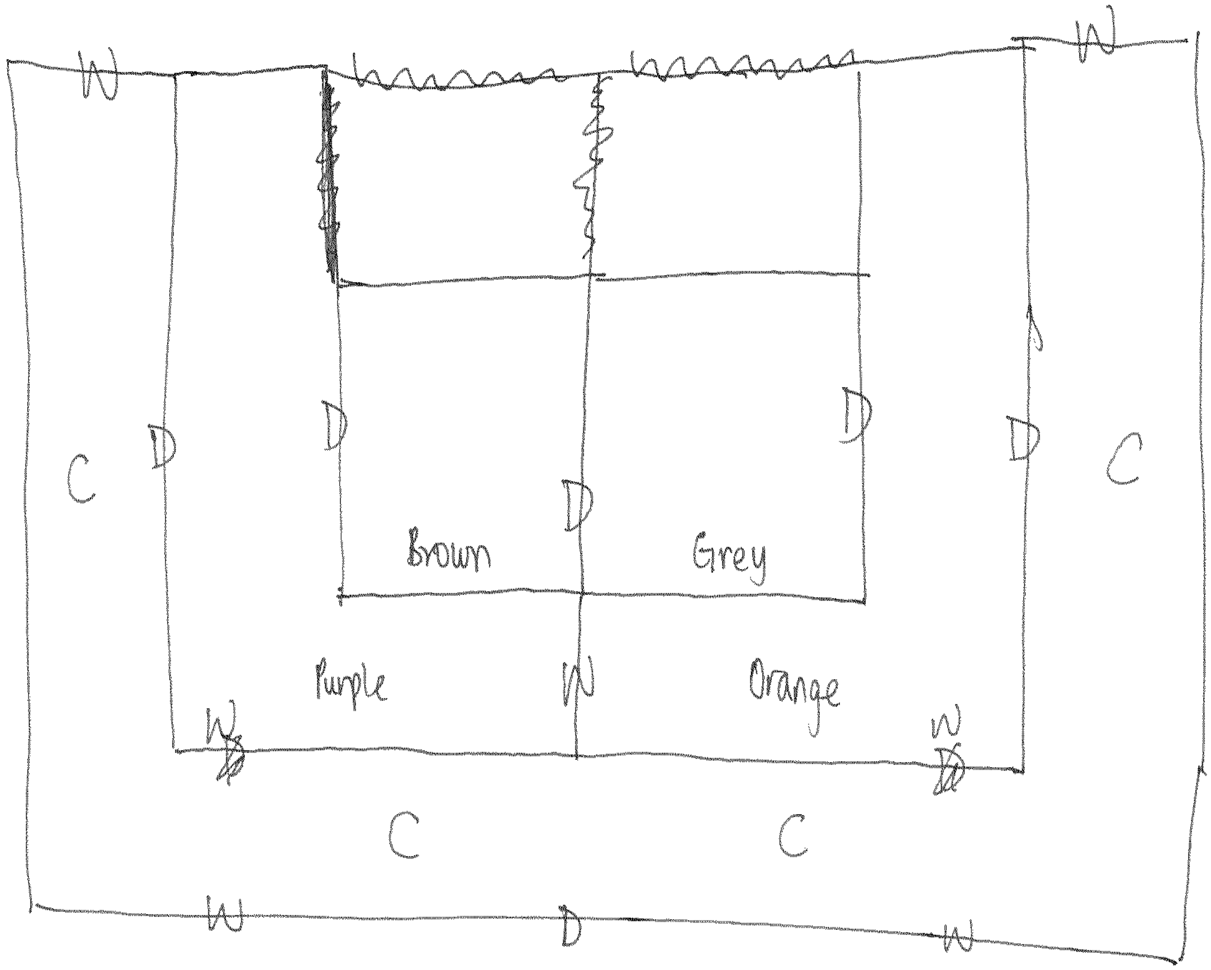

38 map 1 Rotational Spoken

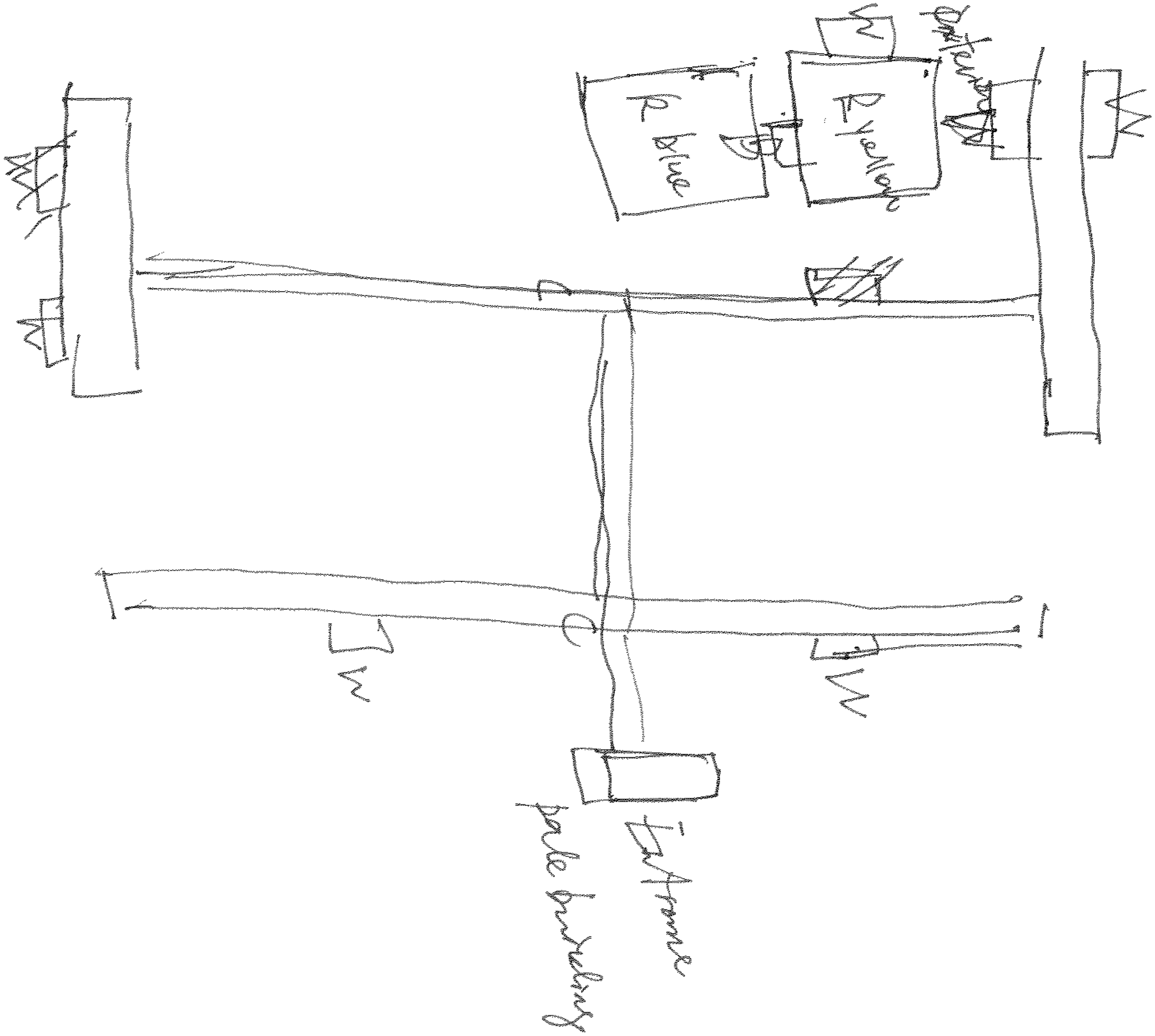

38 map 2 Mirror Video

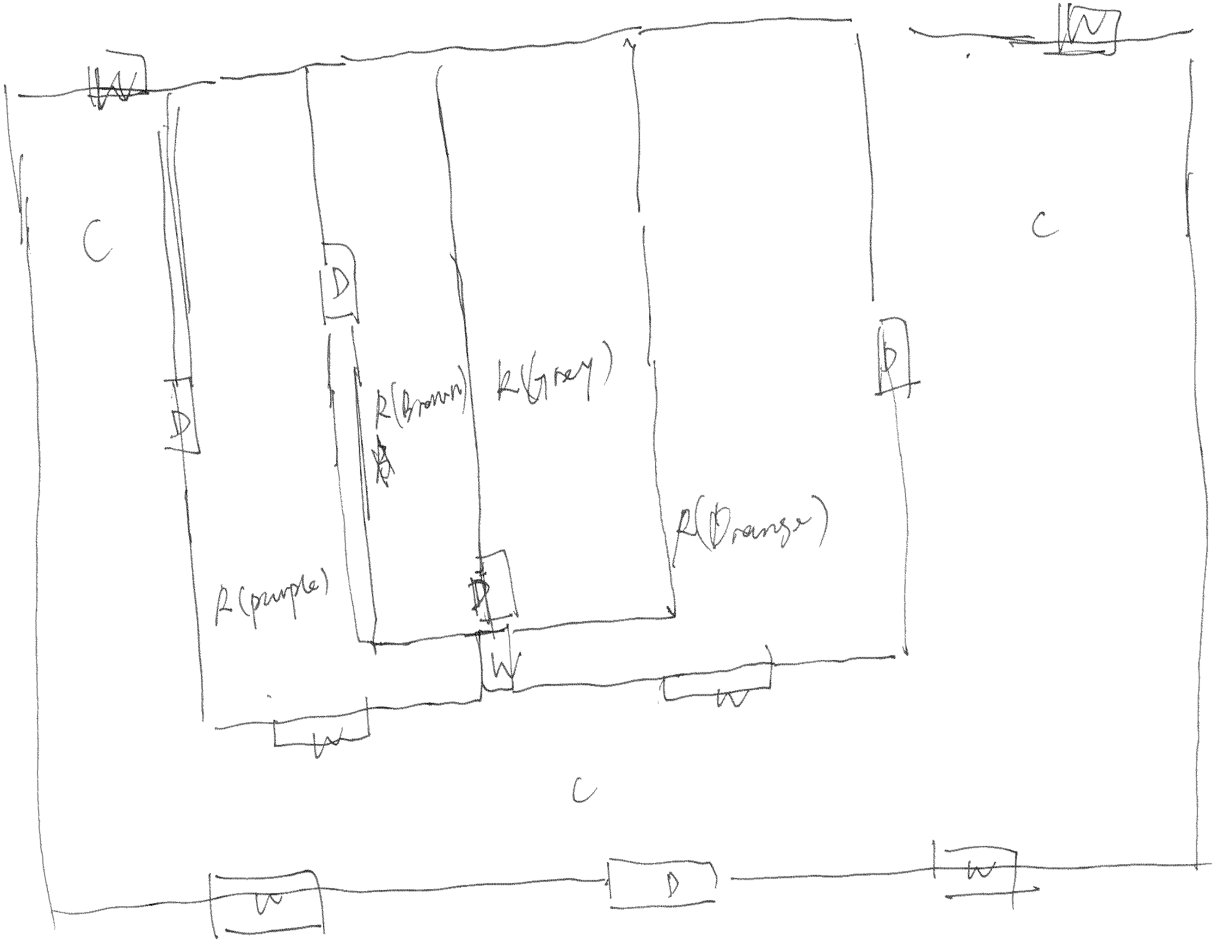

39 map 1 Mirror Video

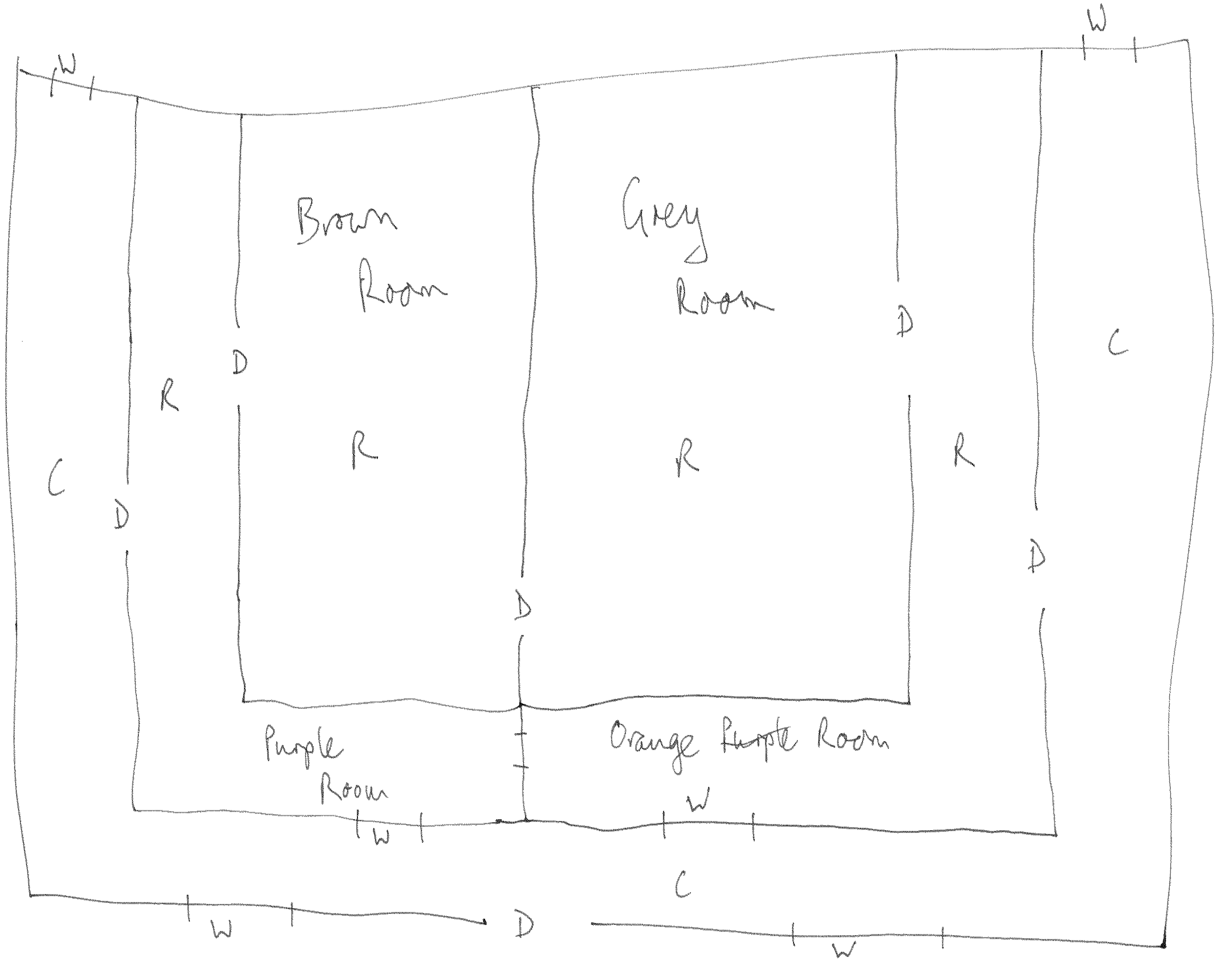

39 map 2 Rotational Spoken

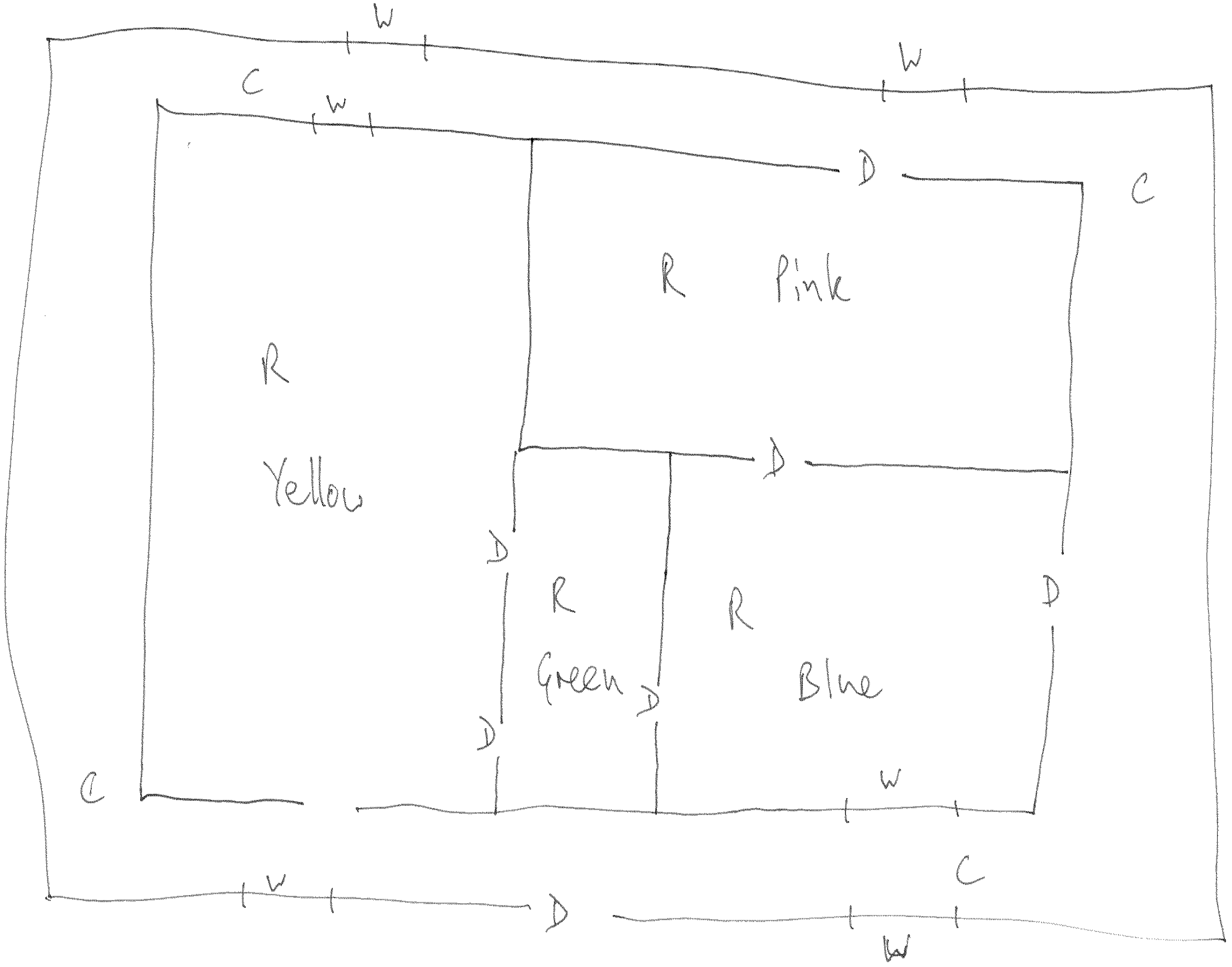

40 map 1 Rotational Spoken

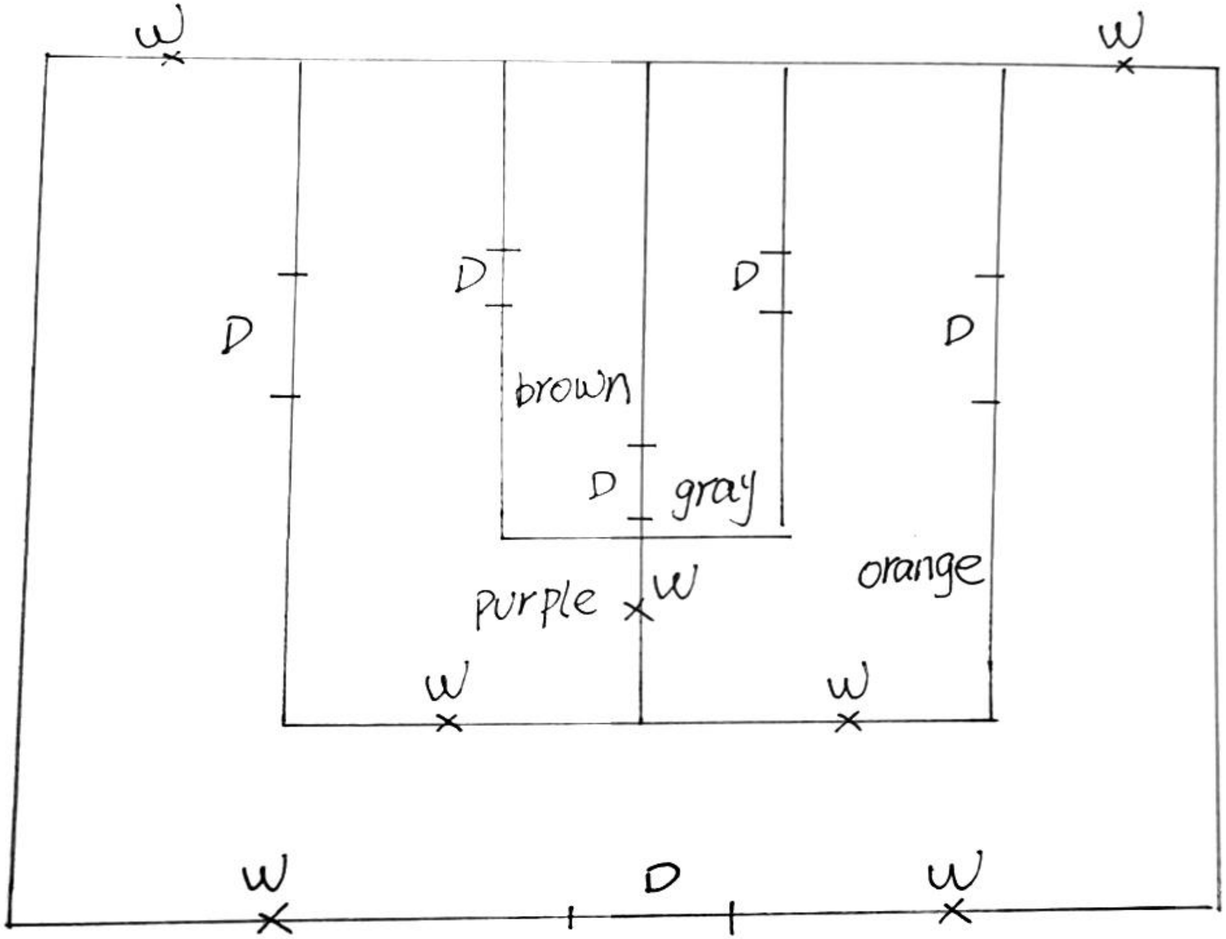

40 map 2 Mirror Video

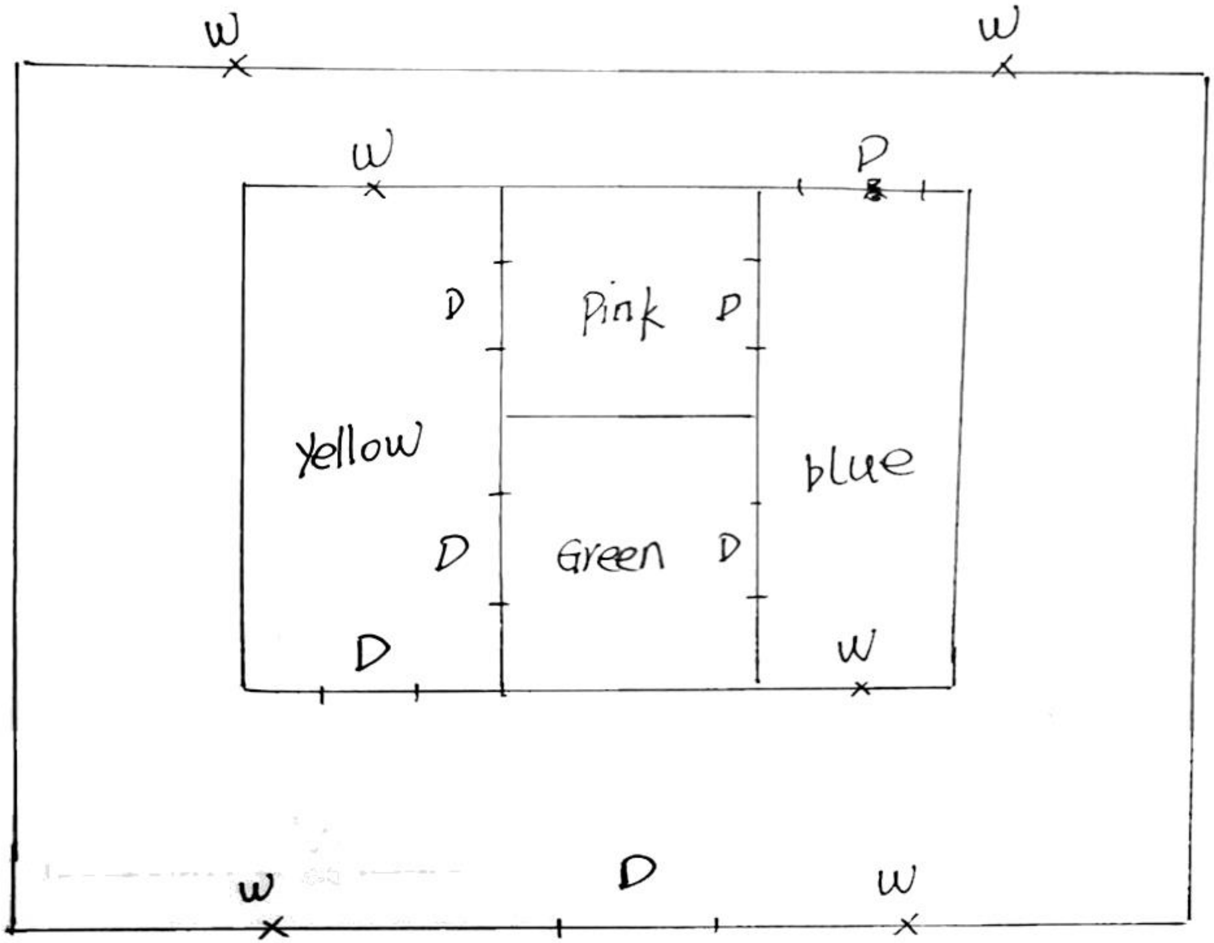

41 map 1 Mirror Written

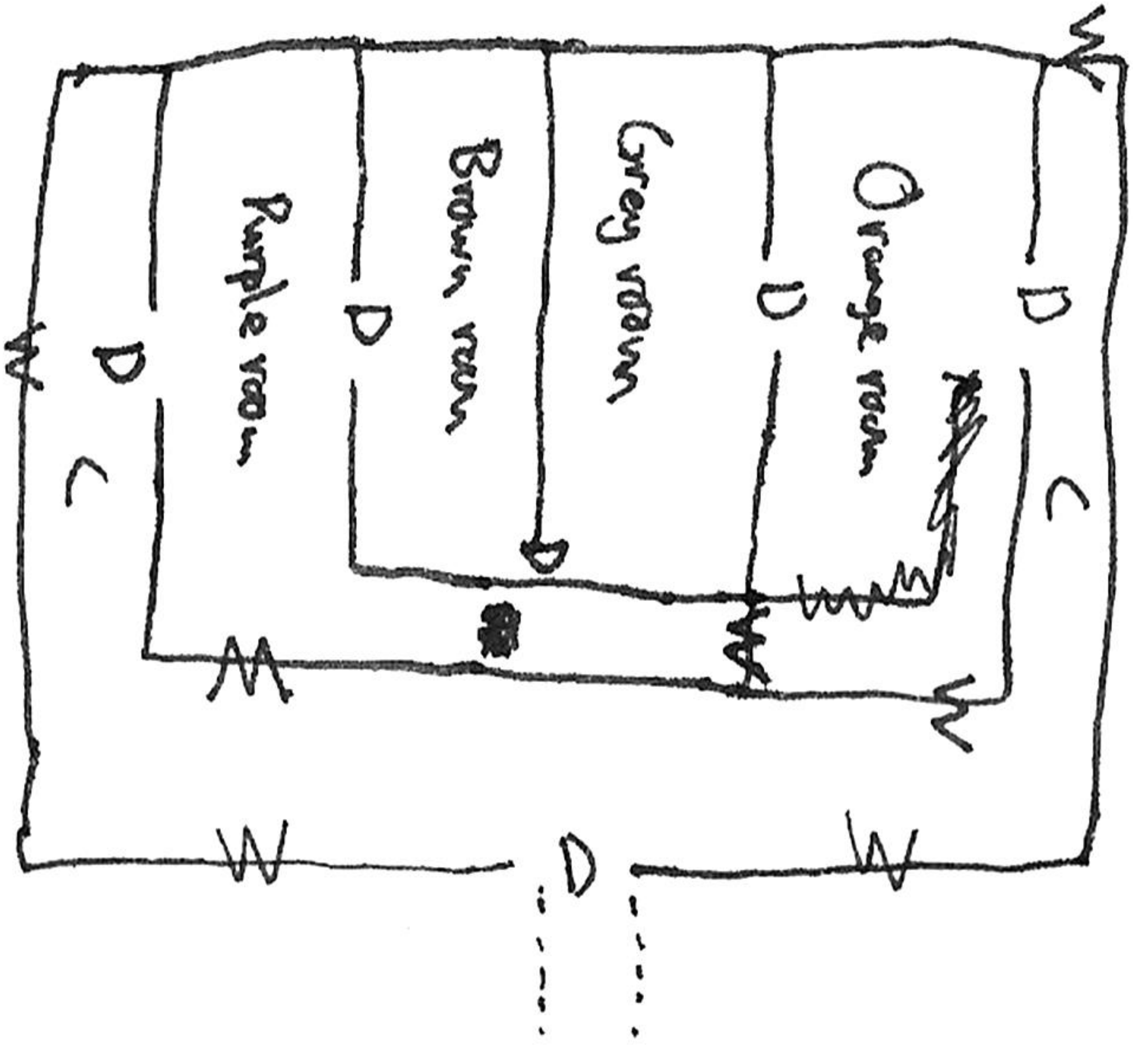

41 map 2 Rotational Video

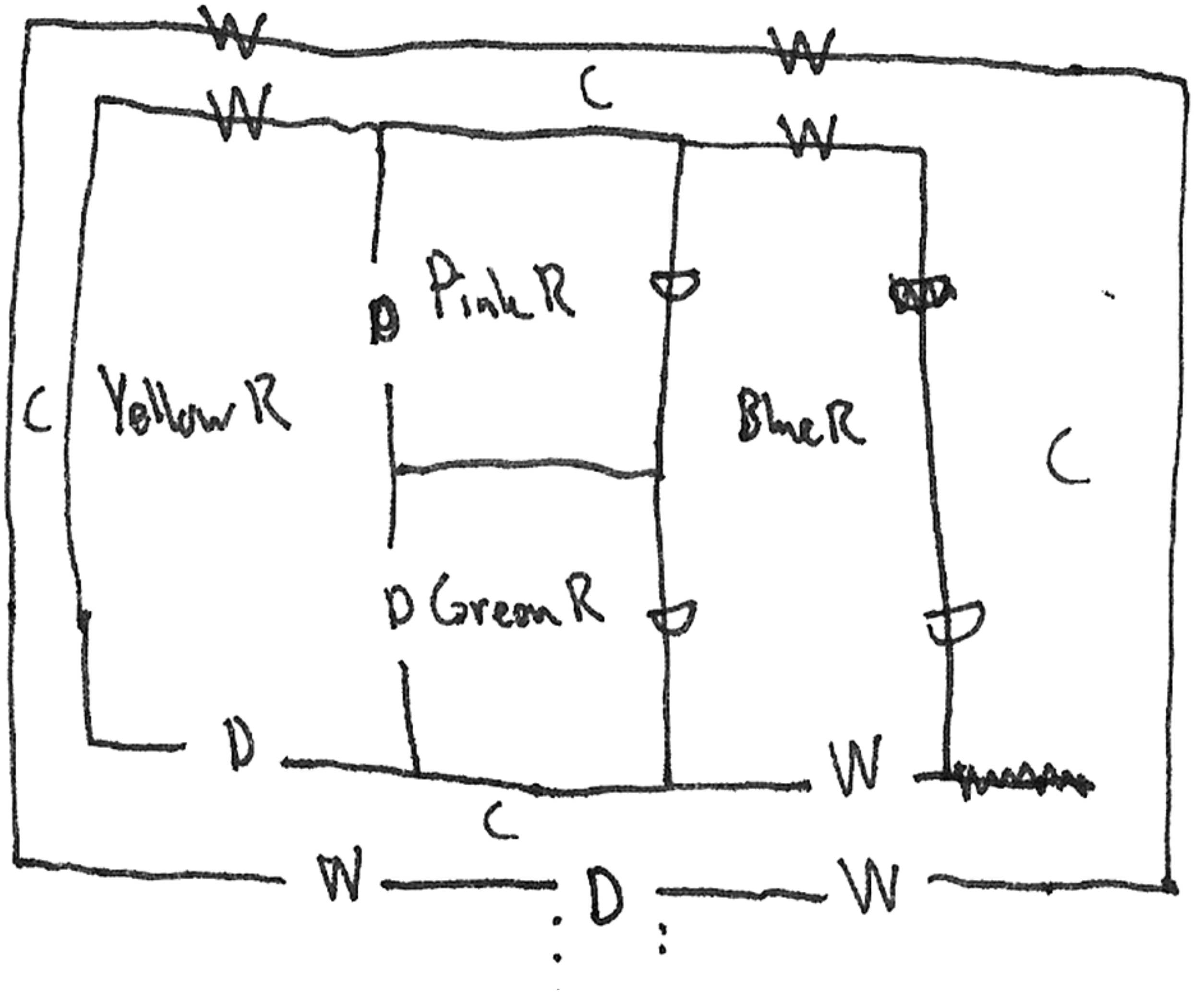

42 map 1 Rotational Video

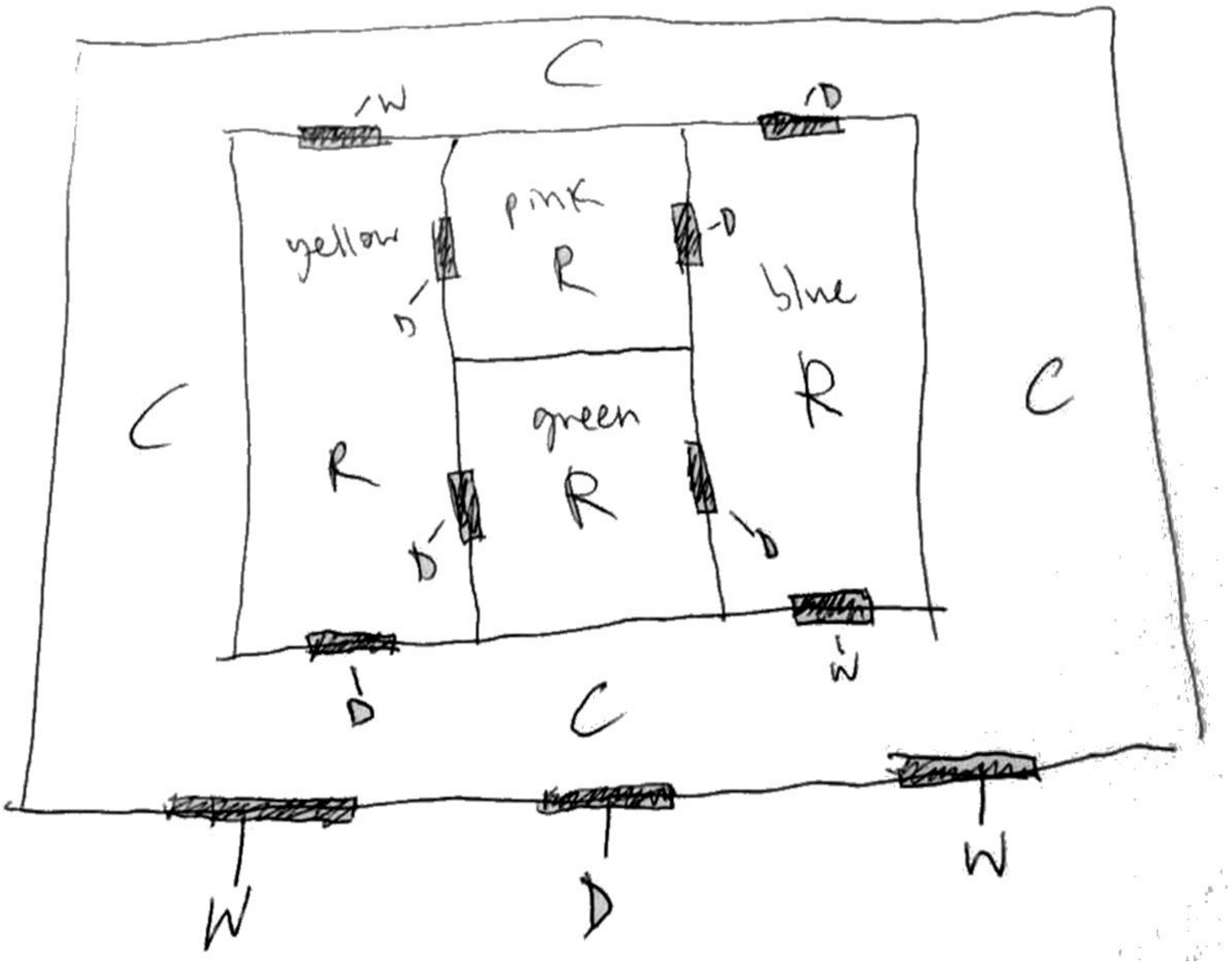

42 map 2 Mirror Written

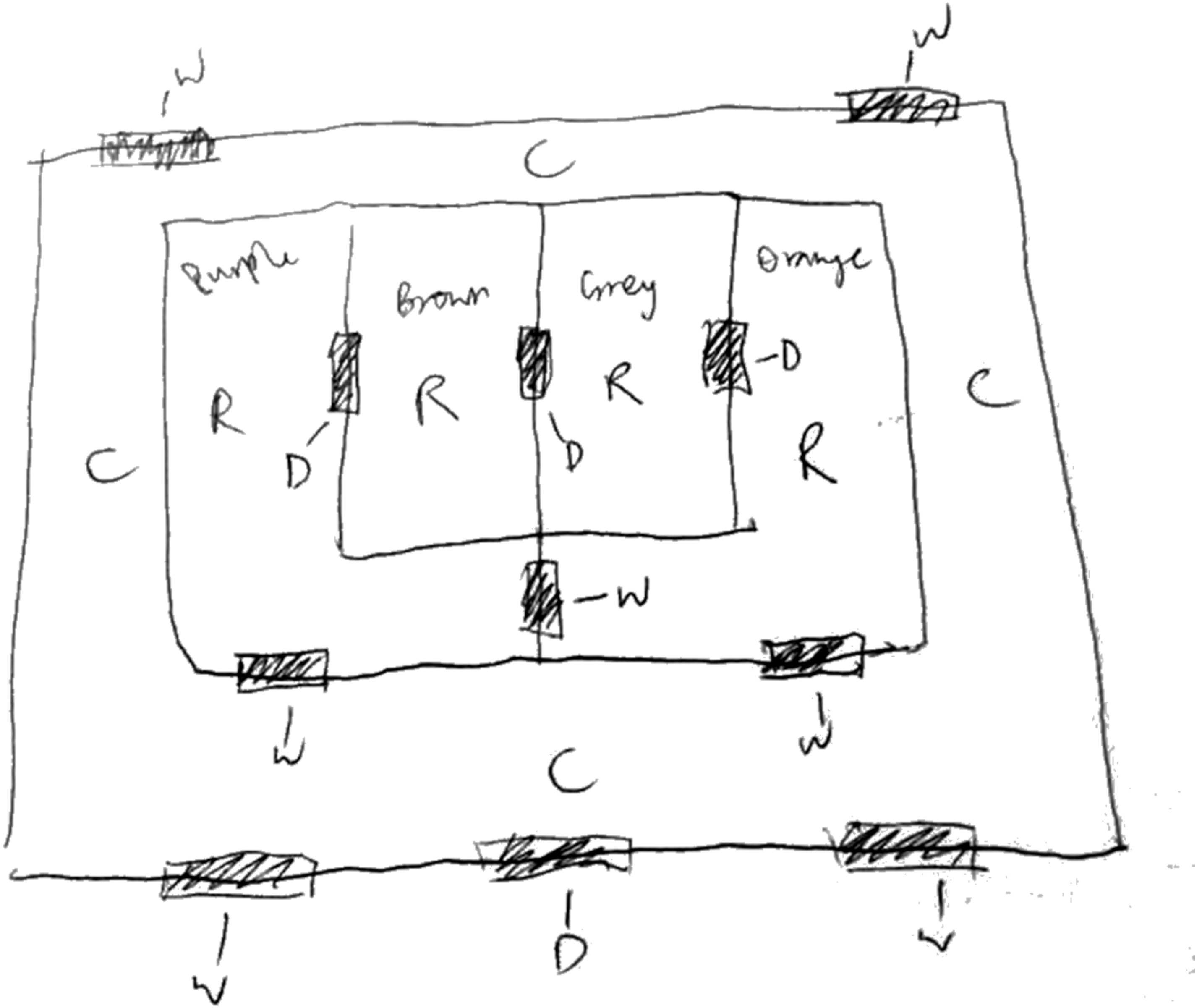

43 map 1 Mirror Video

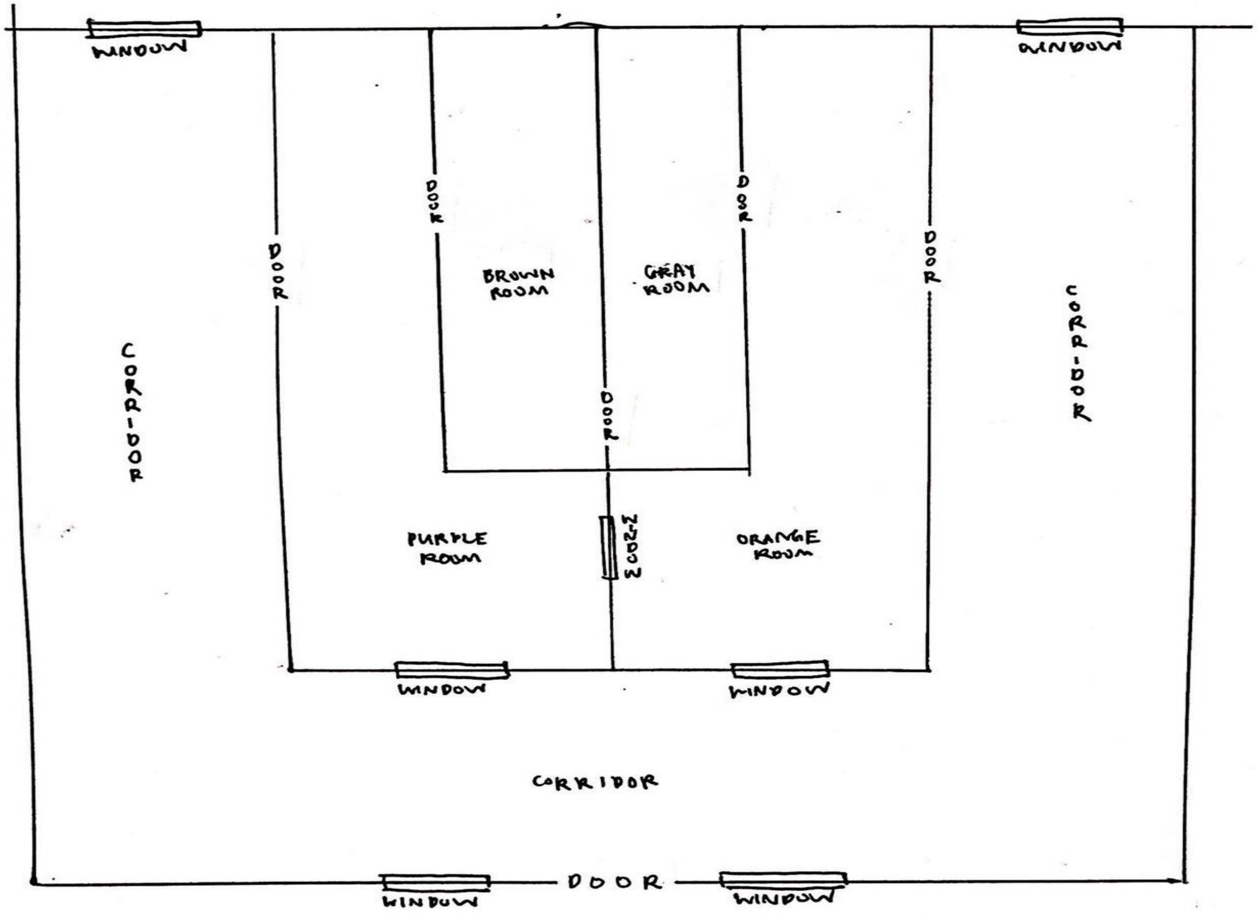

43 map 2 Rotational Written

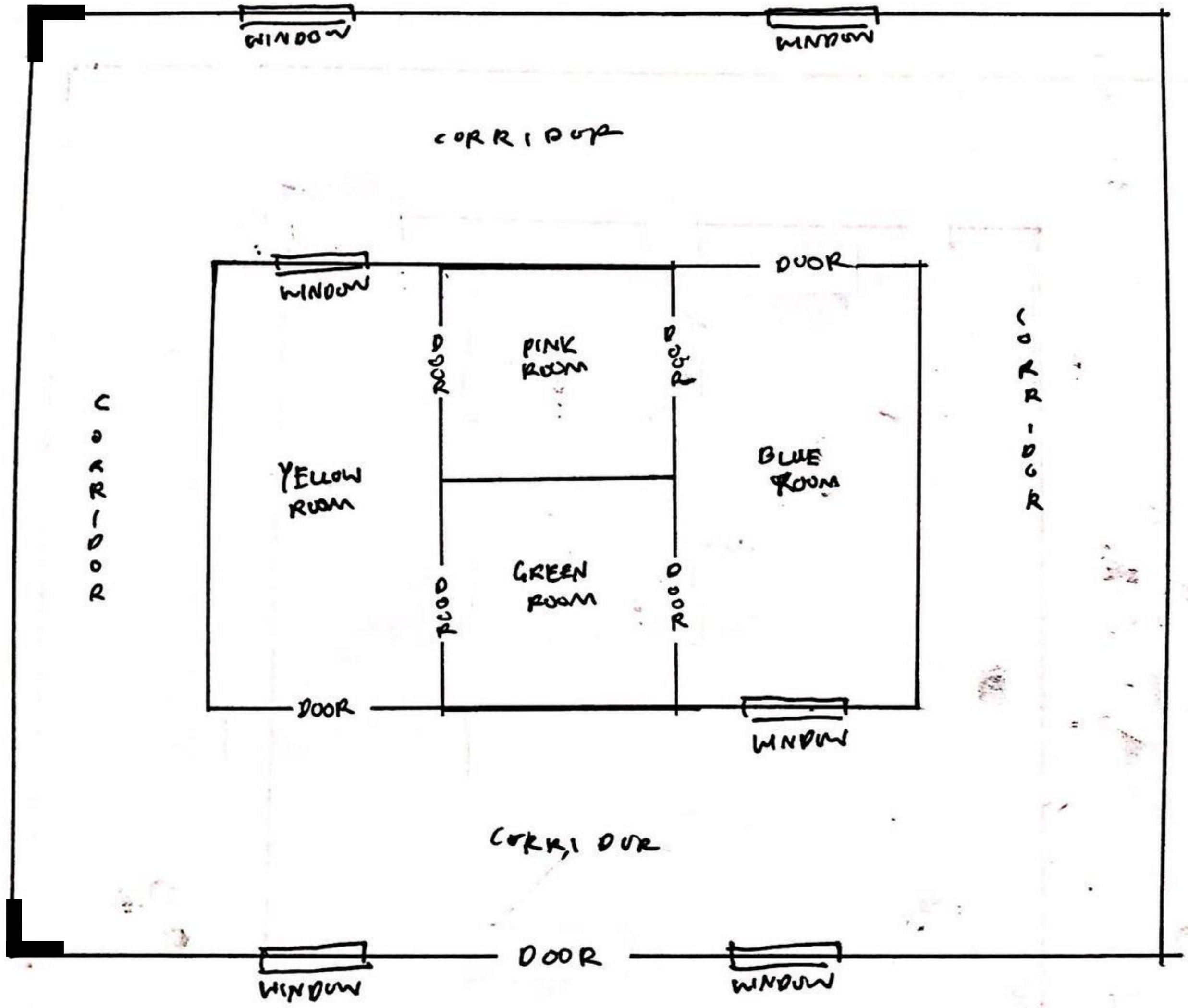

44 map 1 Rotational Written

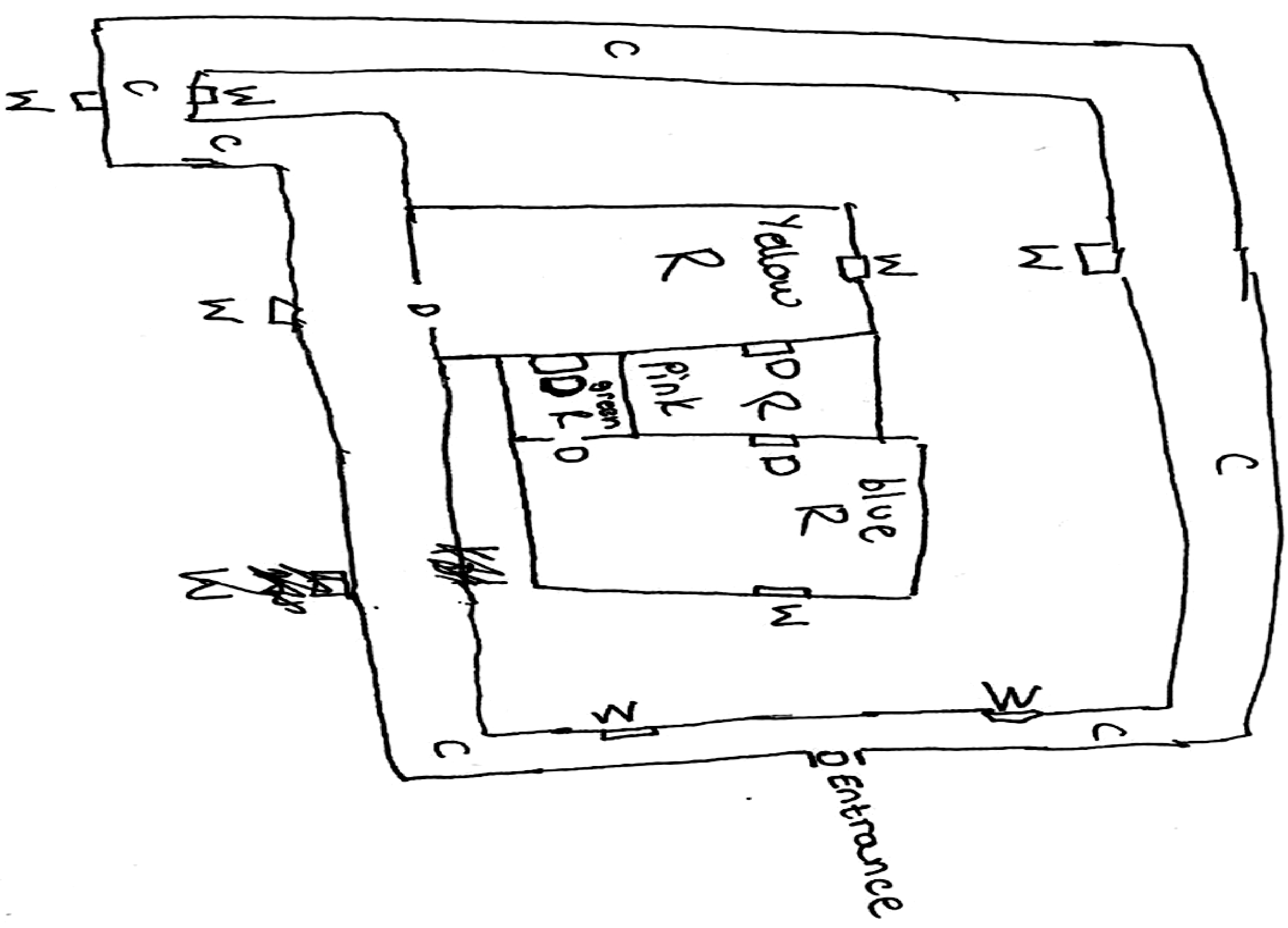

44 map 2 Mirror Video

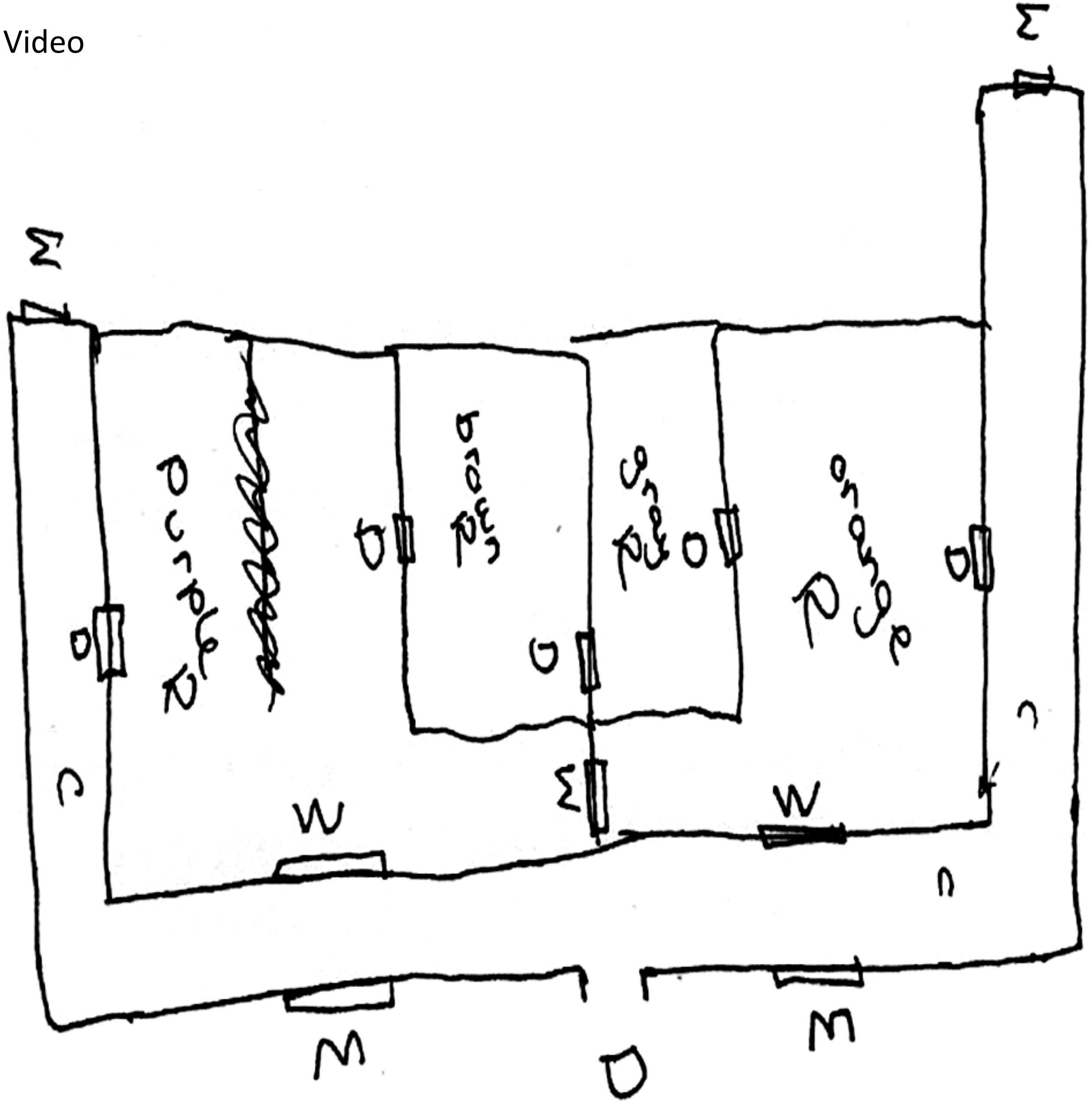

45 map 1 Mirror Video

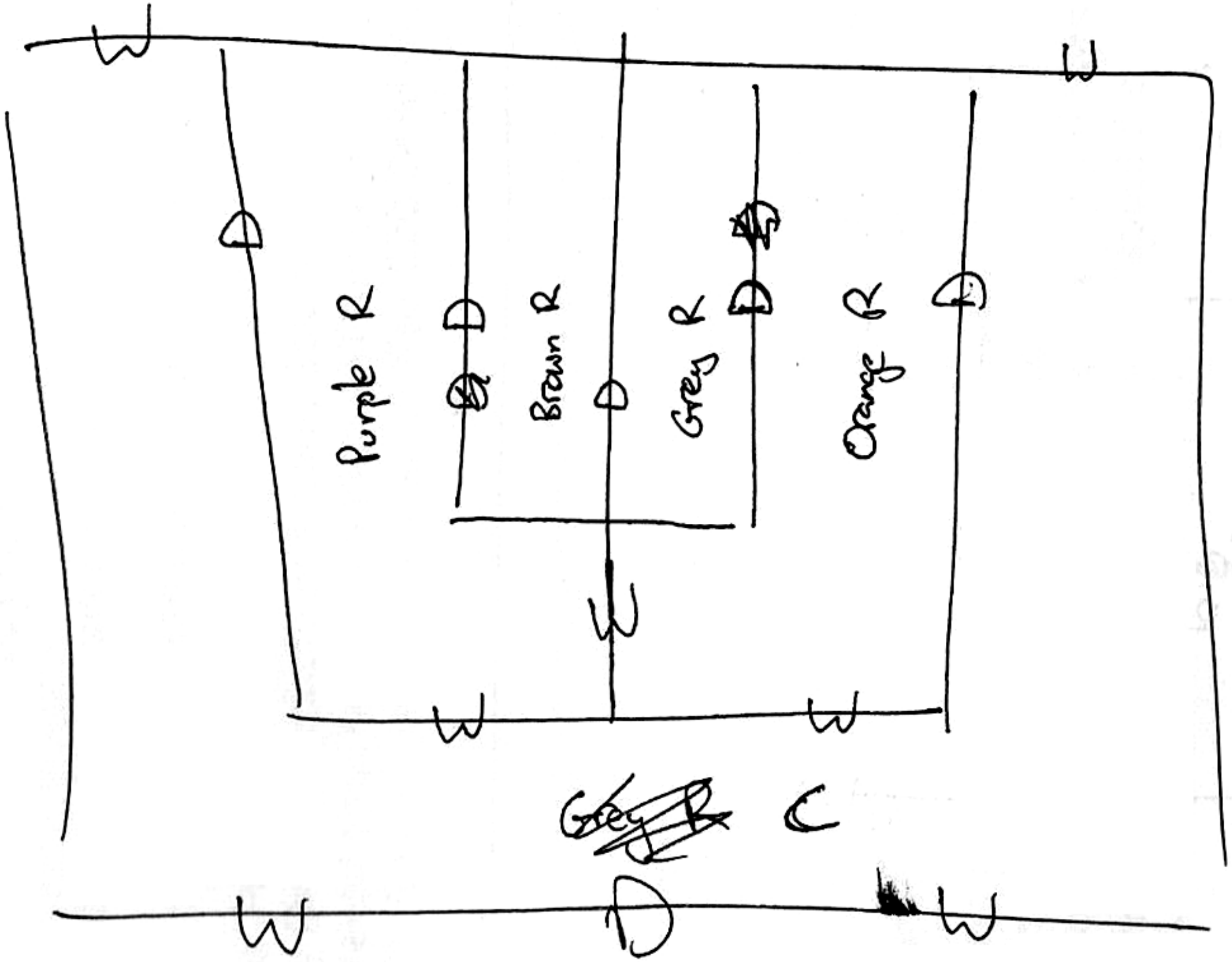

45 map 2 Rotational Written

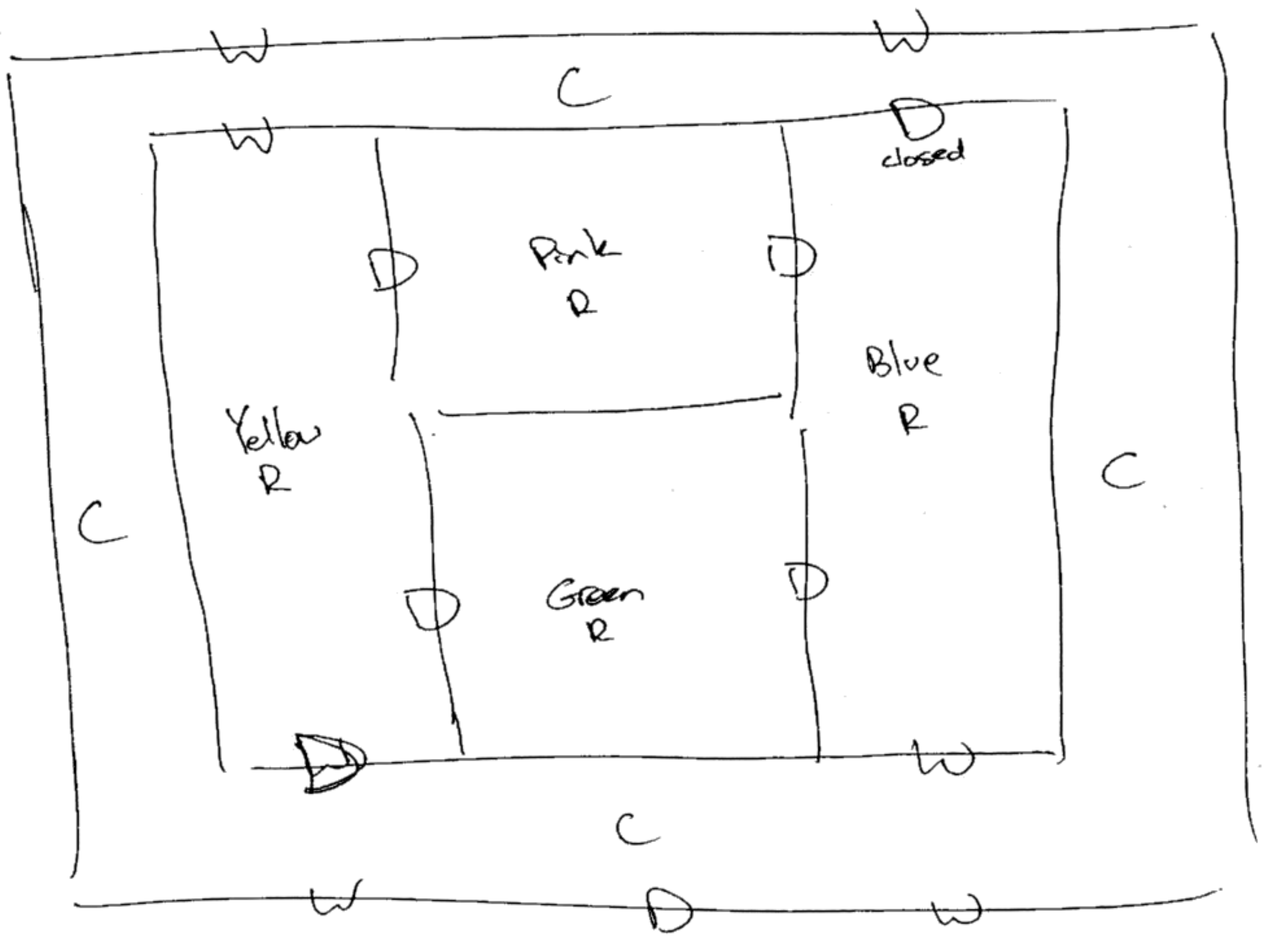

46 map 1 Rotational Video

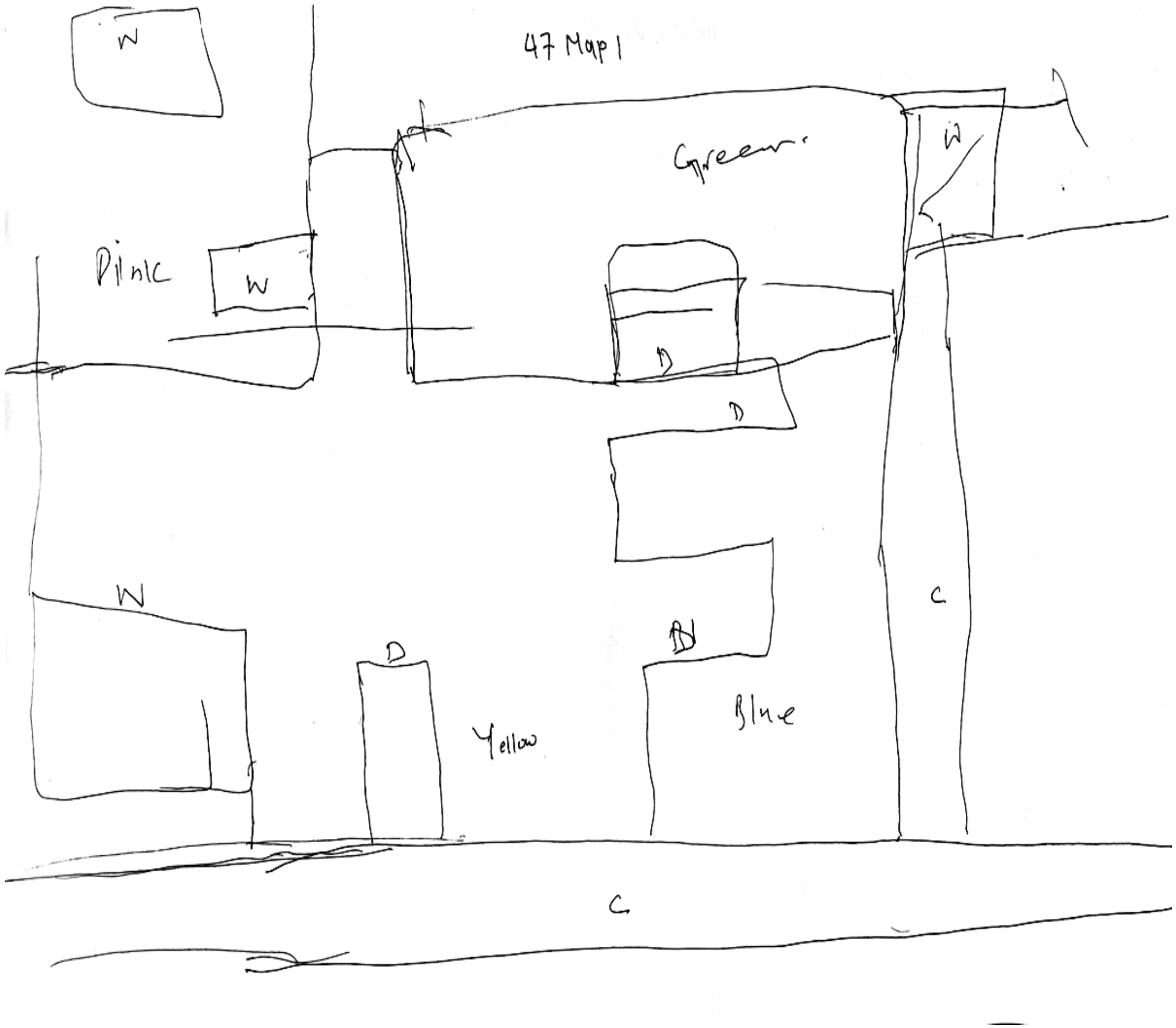

46 map 2 Mirror Written

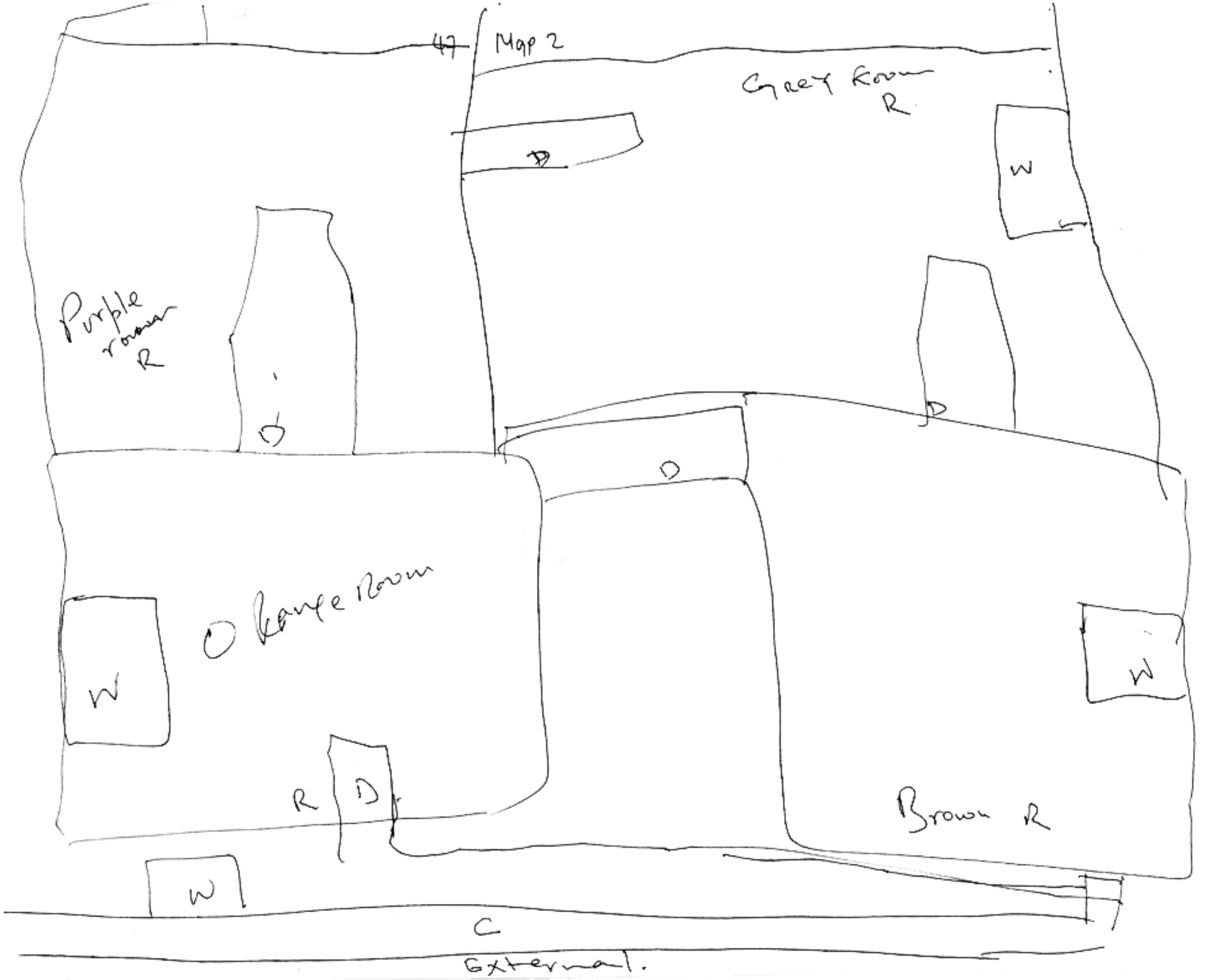

47 map 1 Rotational Written

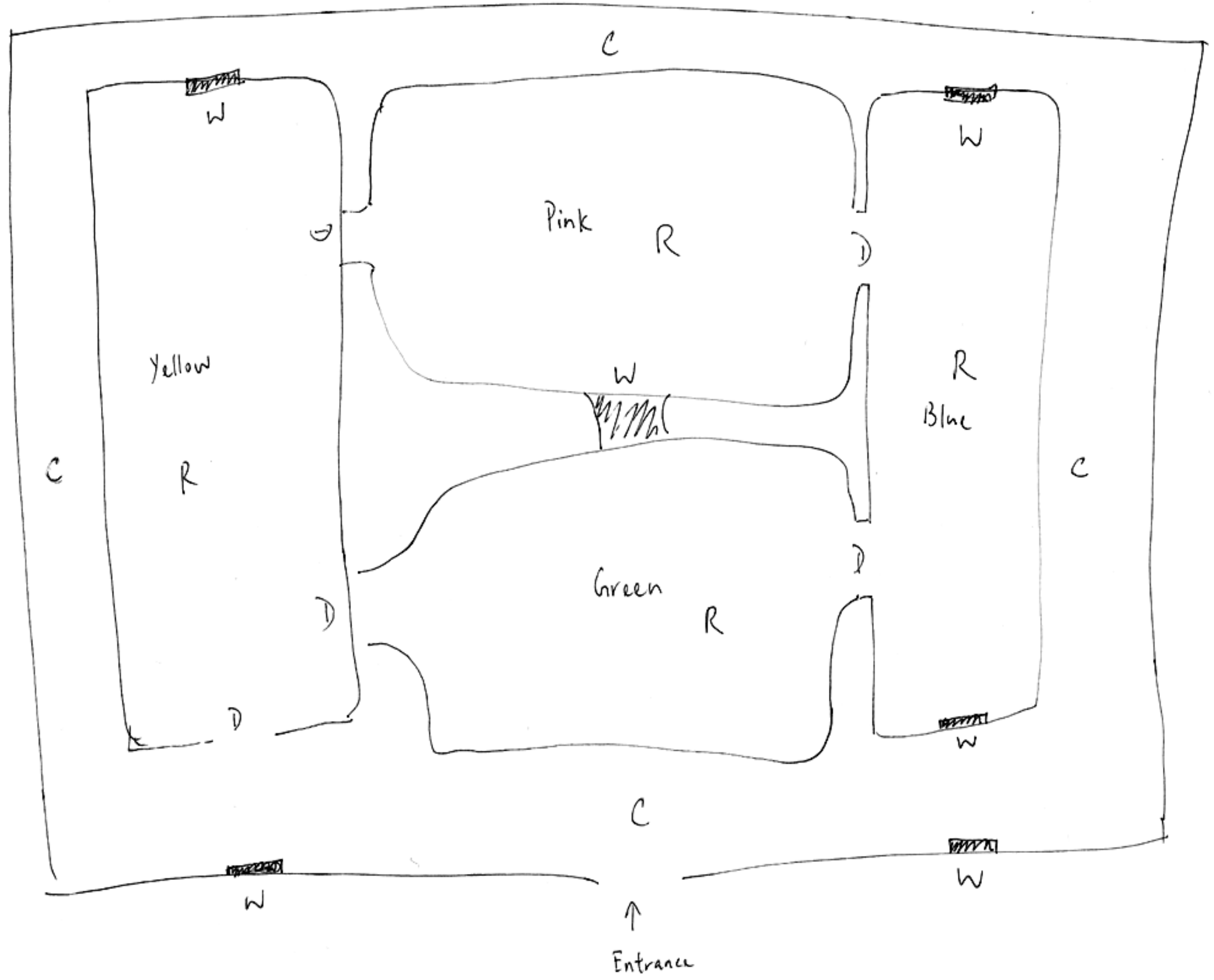

47 map 2 Mirror Video

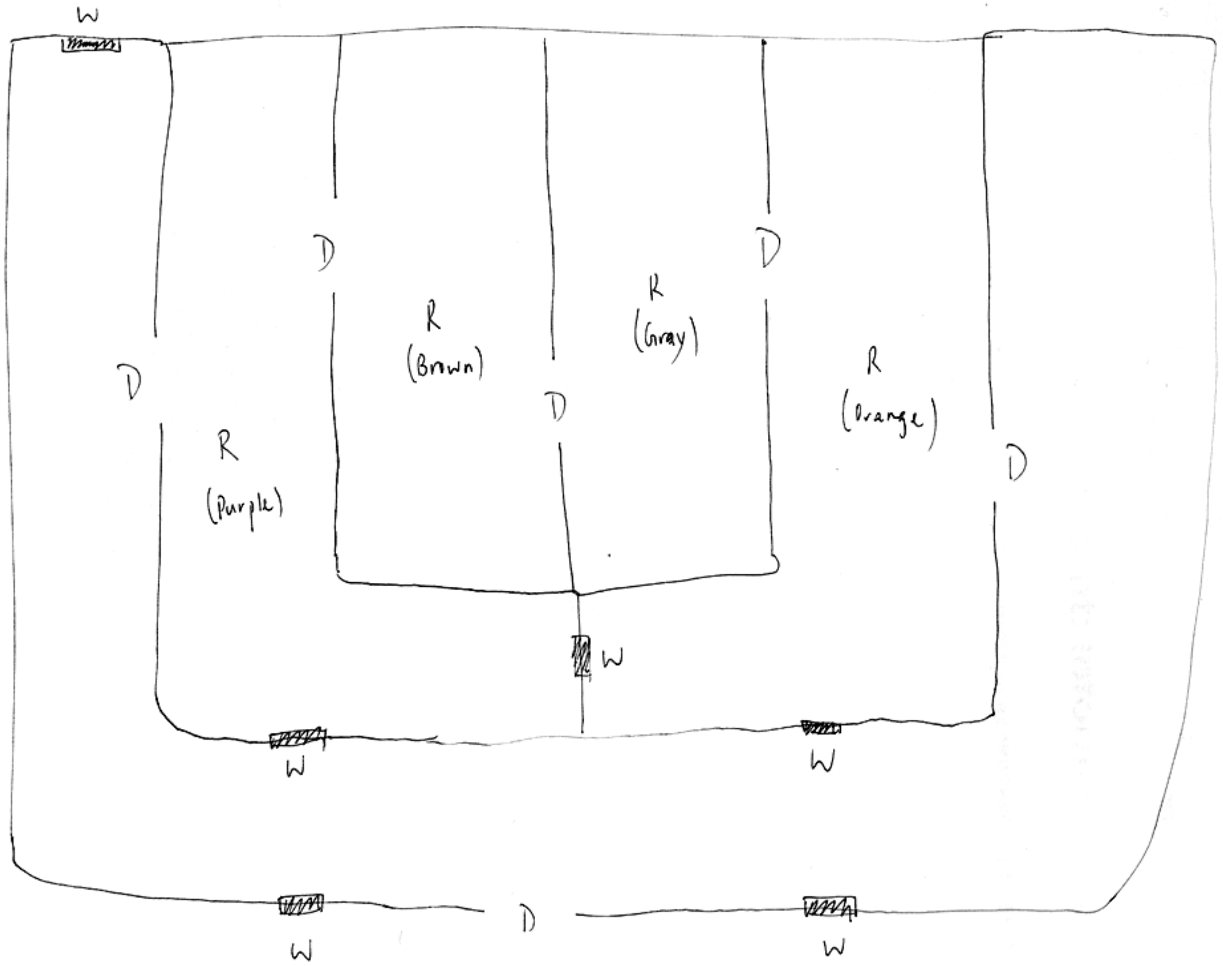

48 map 1 Mirror Written

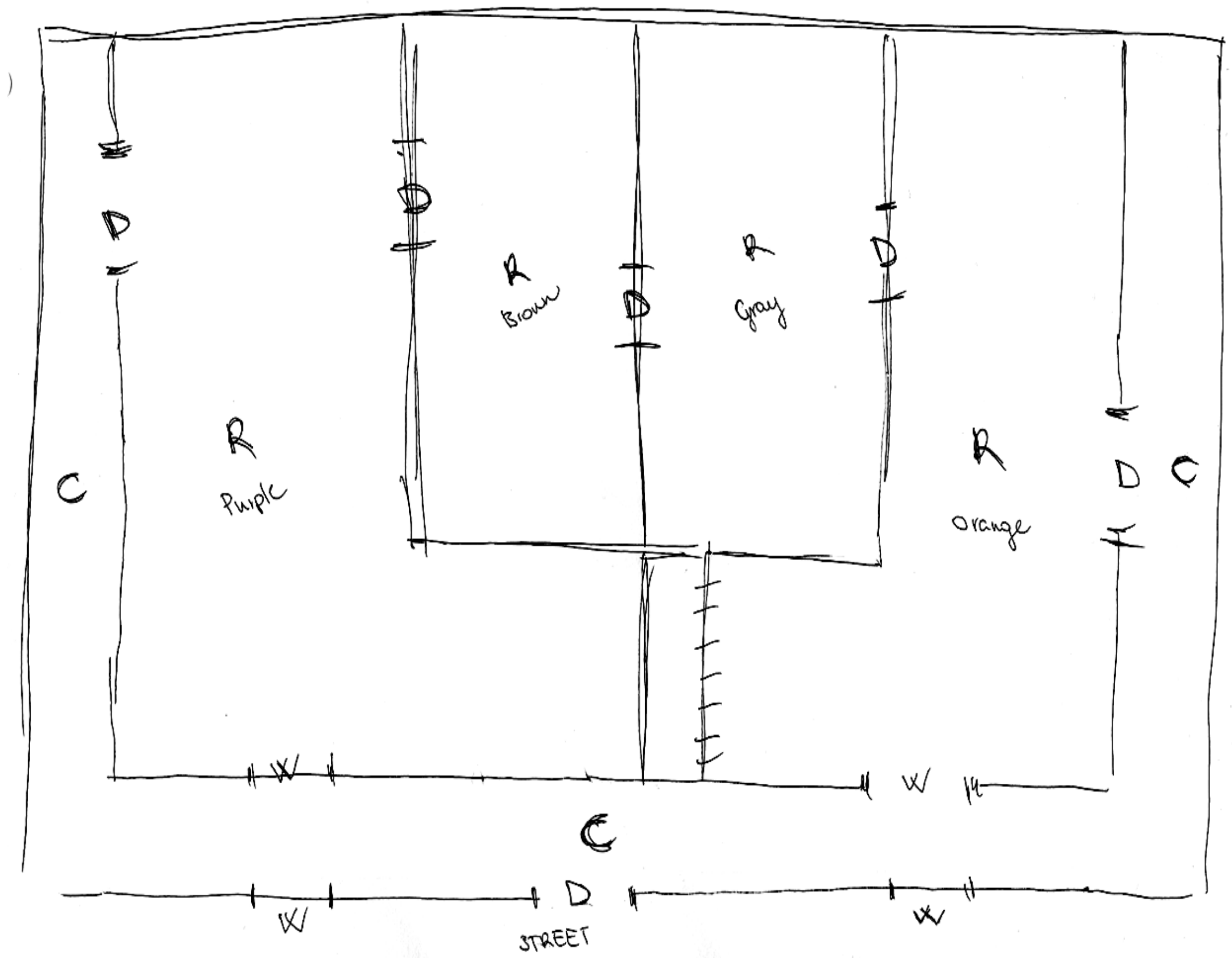

48 map 2 Rotational Video

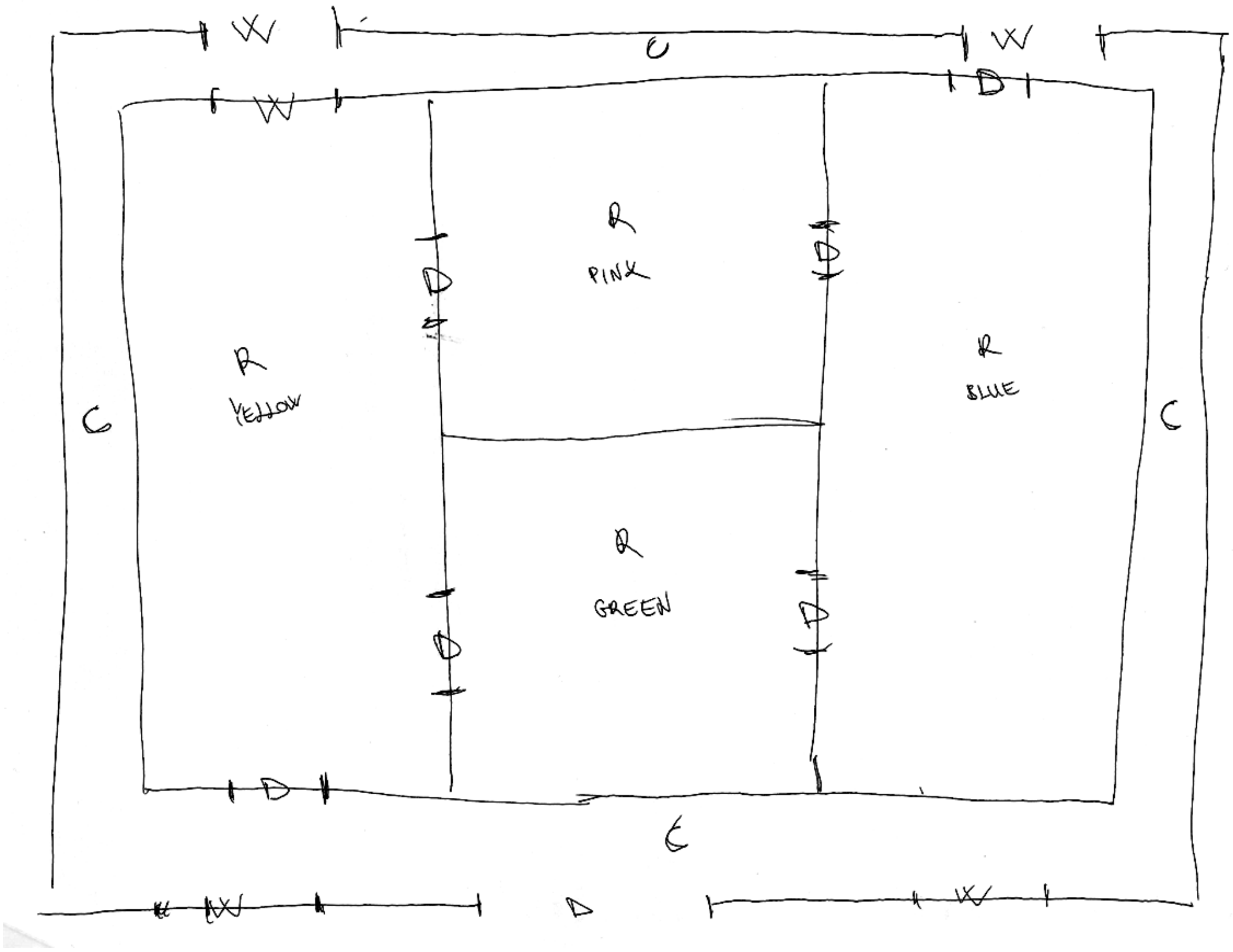

49 map 1 Mirror Video

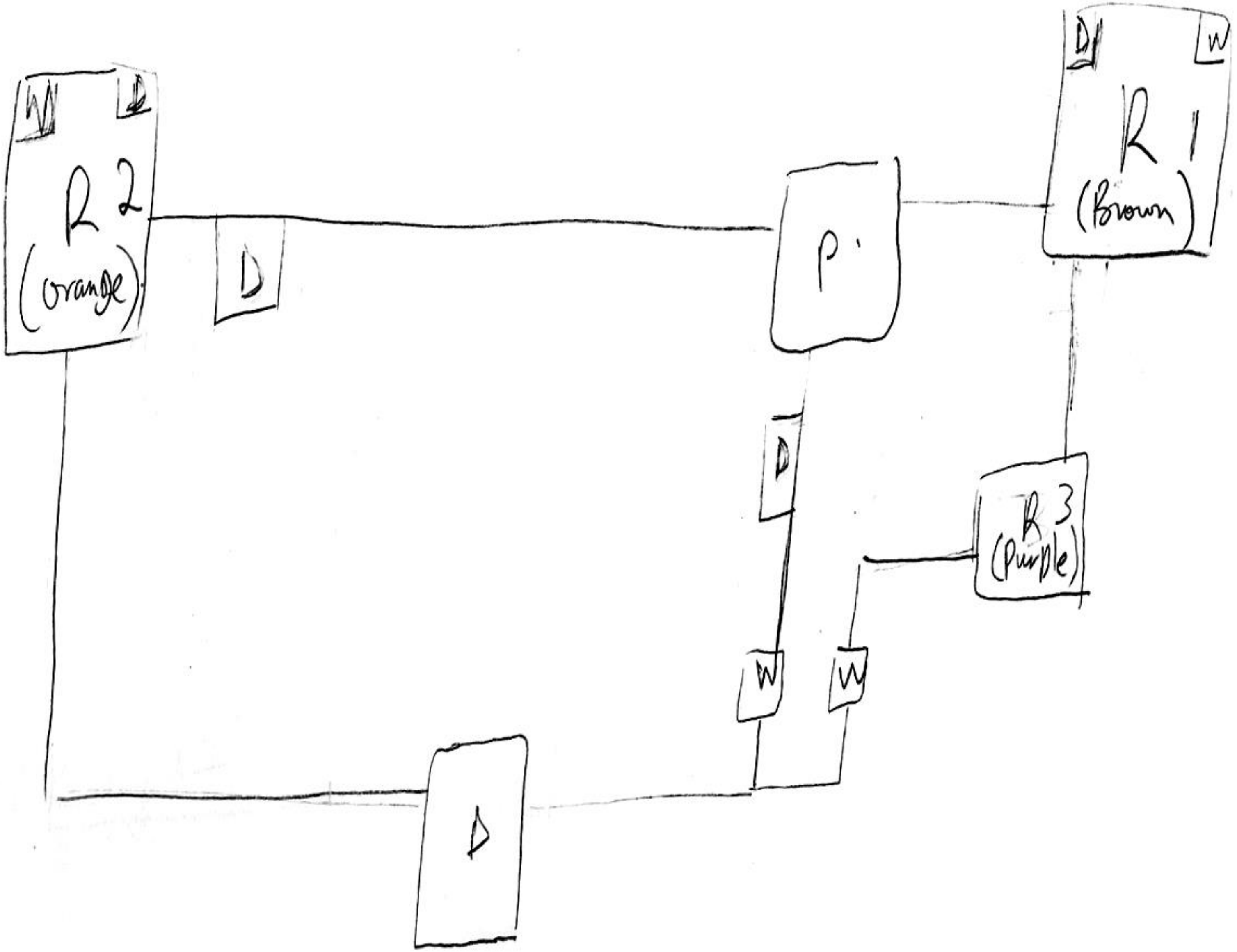

49 map 2 Rotational Written

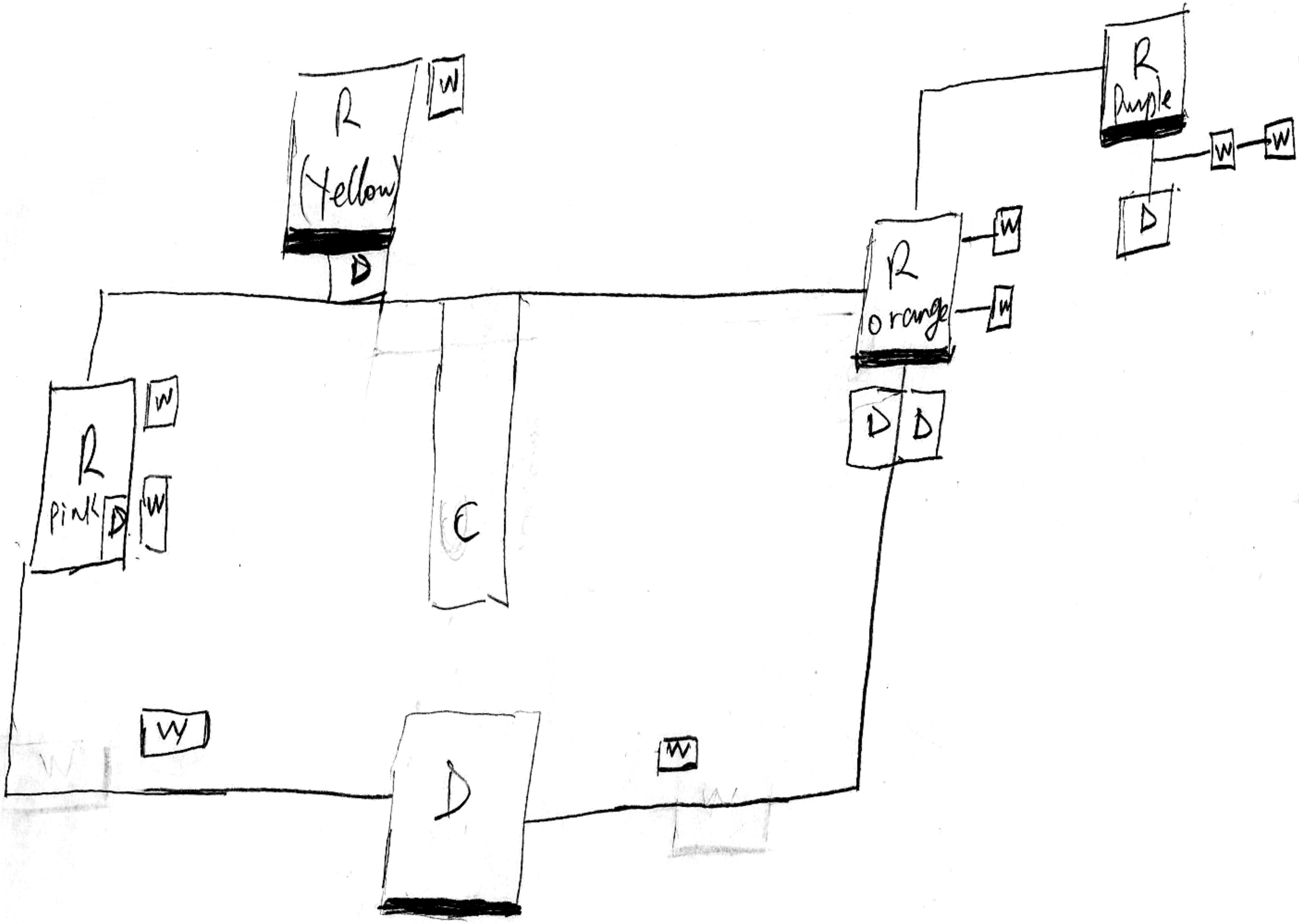

50 map 1 Rotational Video

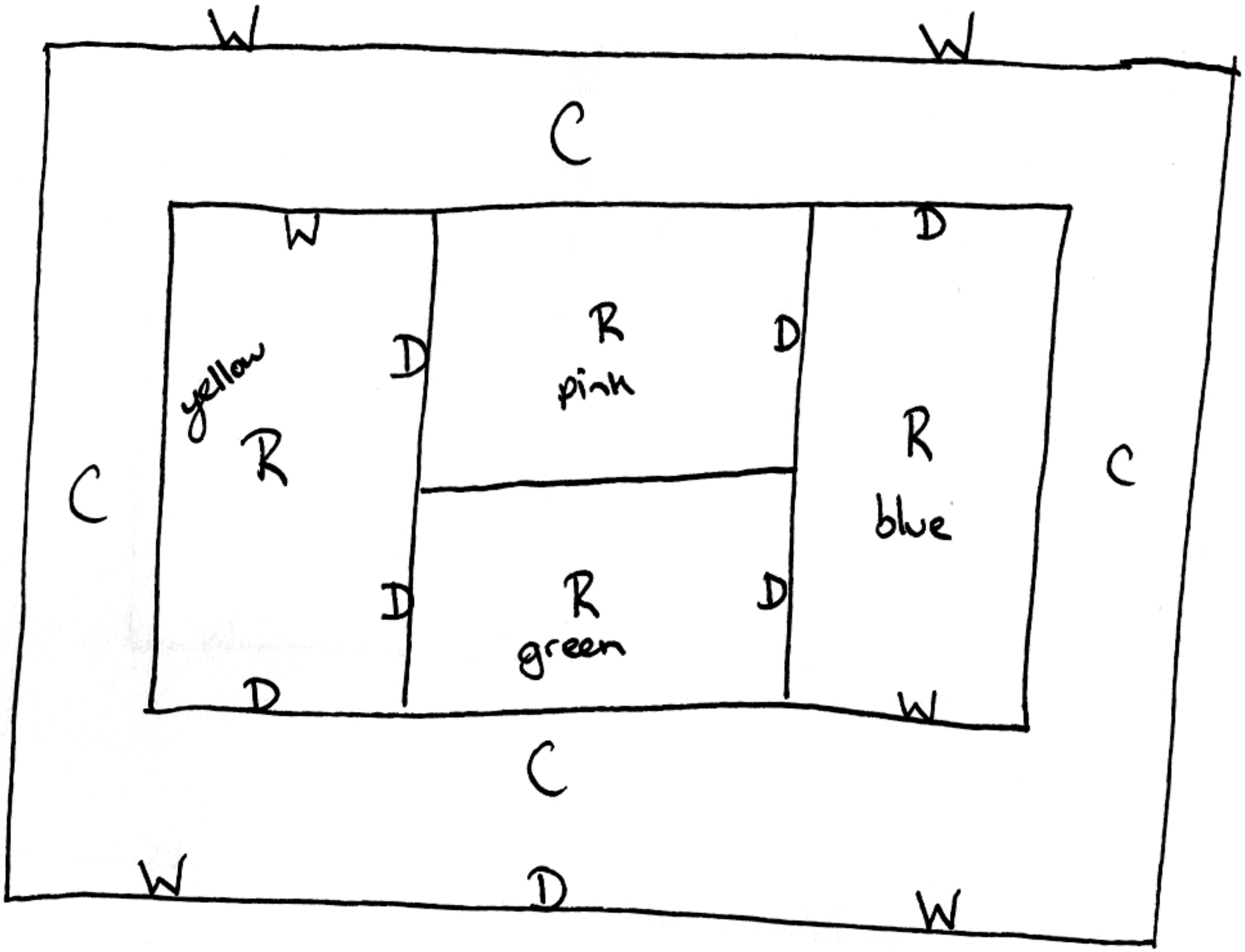

50 map 2 Mirror Written

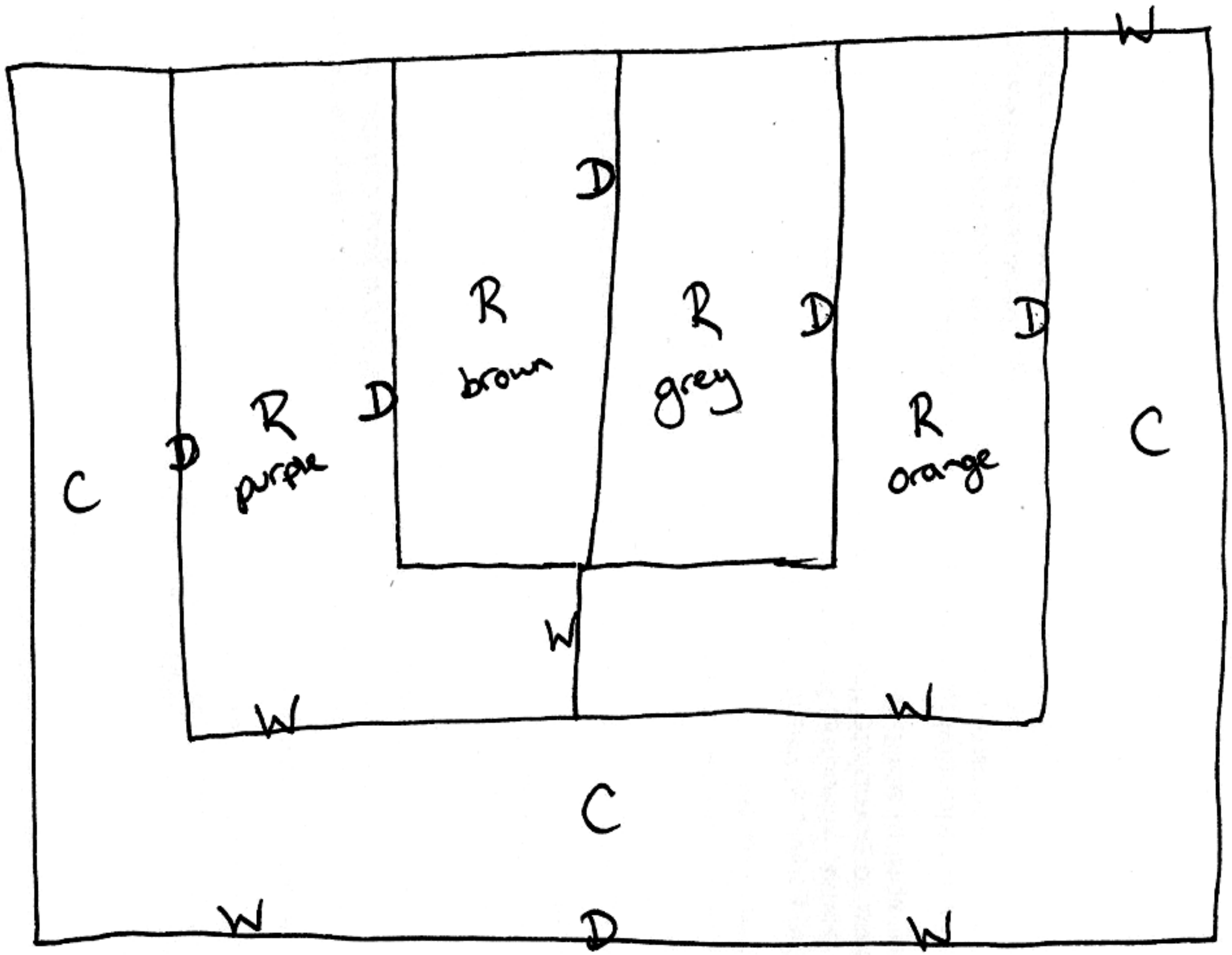

51 map 1 Rotational Written

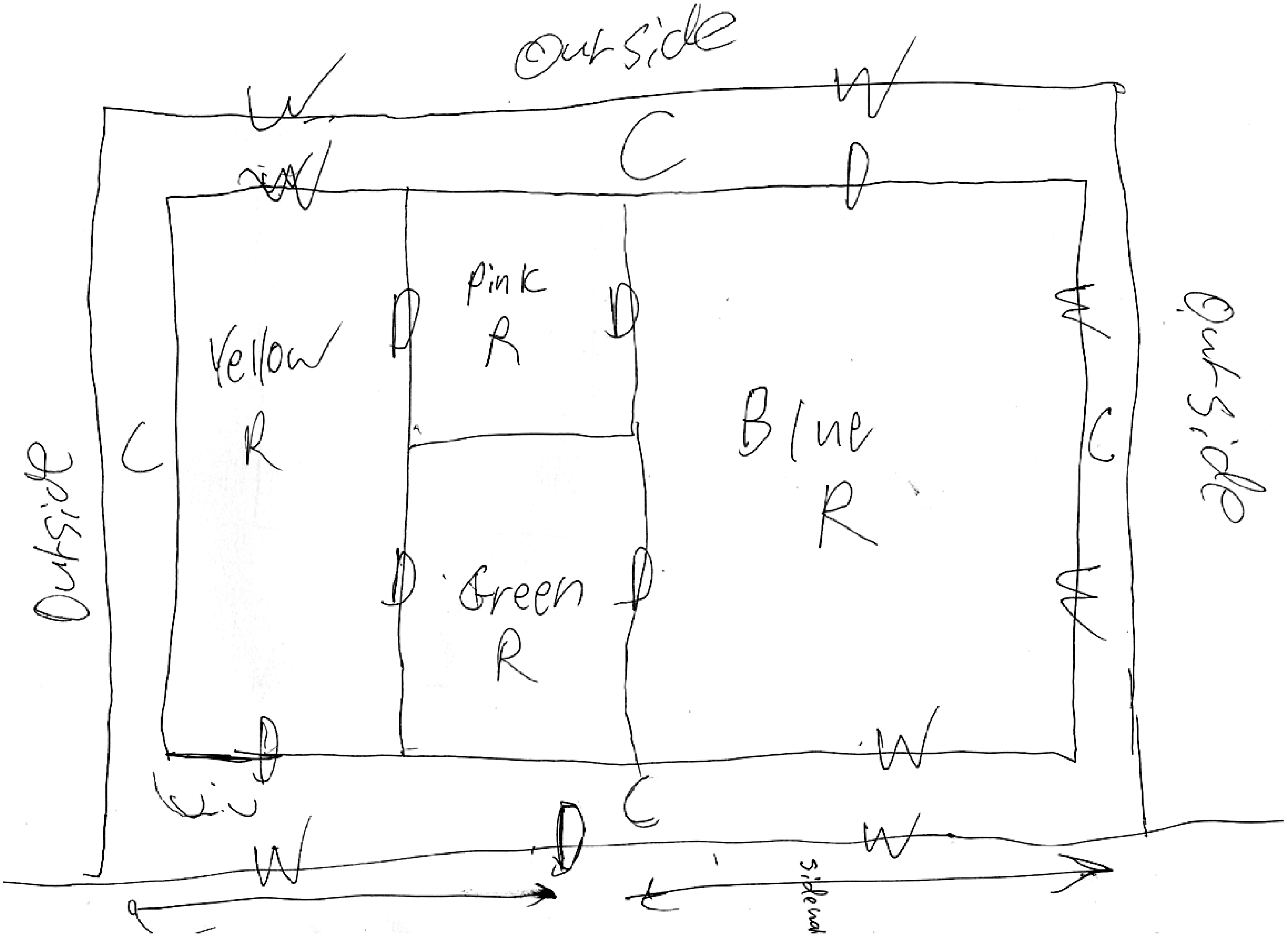

51 map 2 Mirror Video

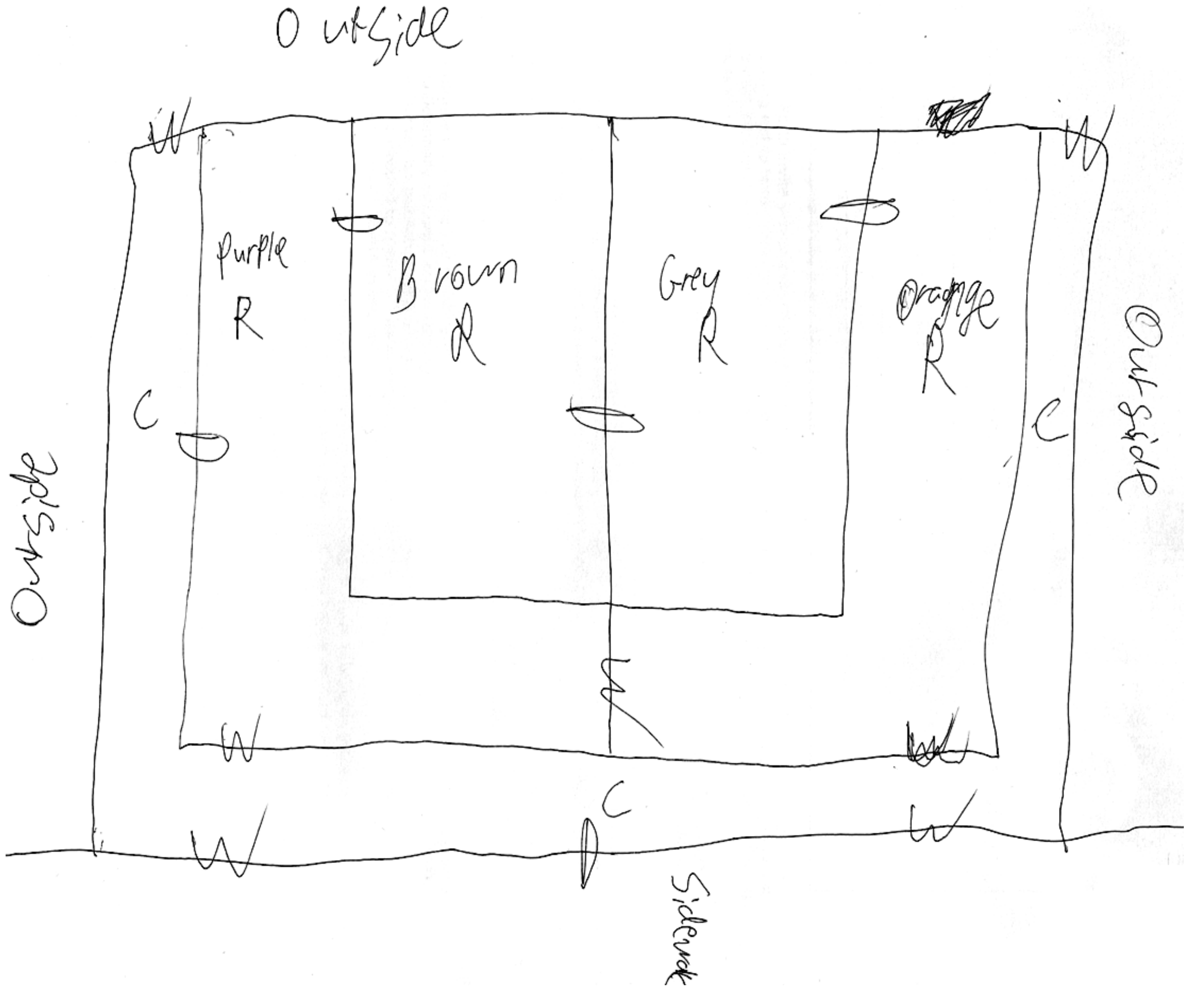

52 map 1 Mirror Video

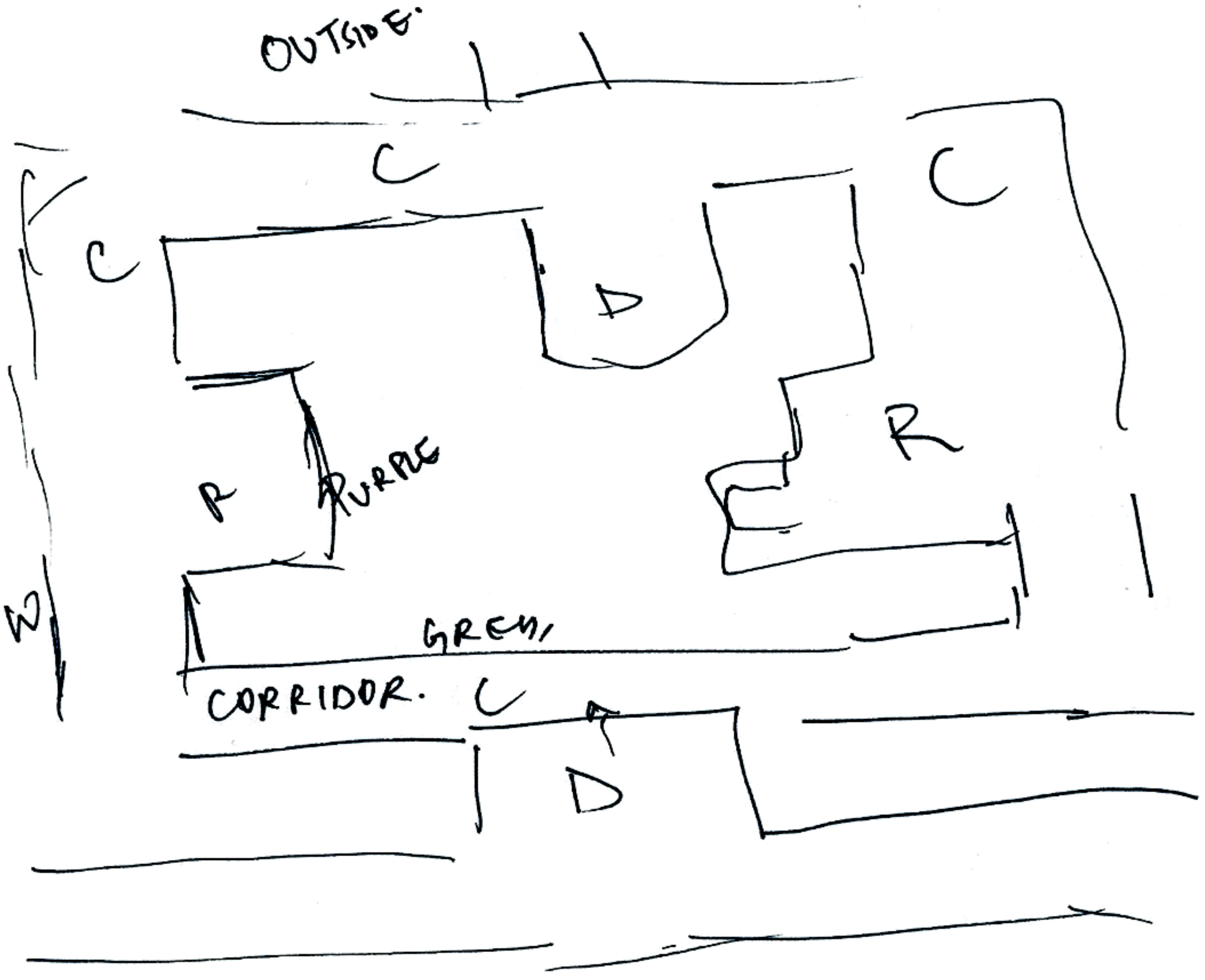

52 map 2 Rotational Written

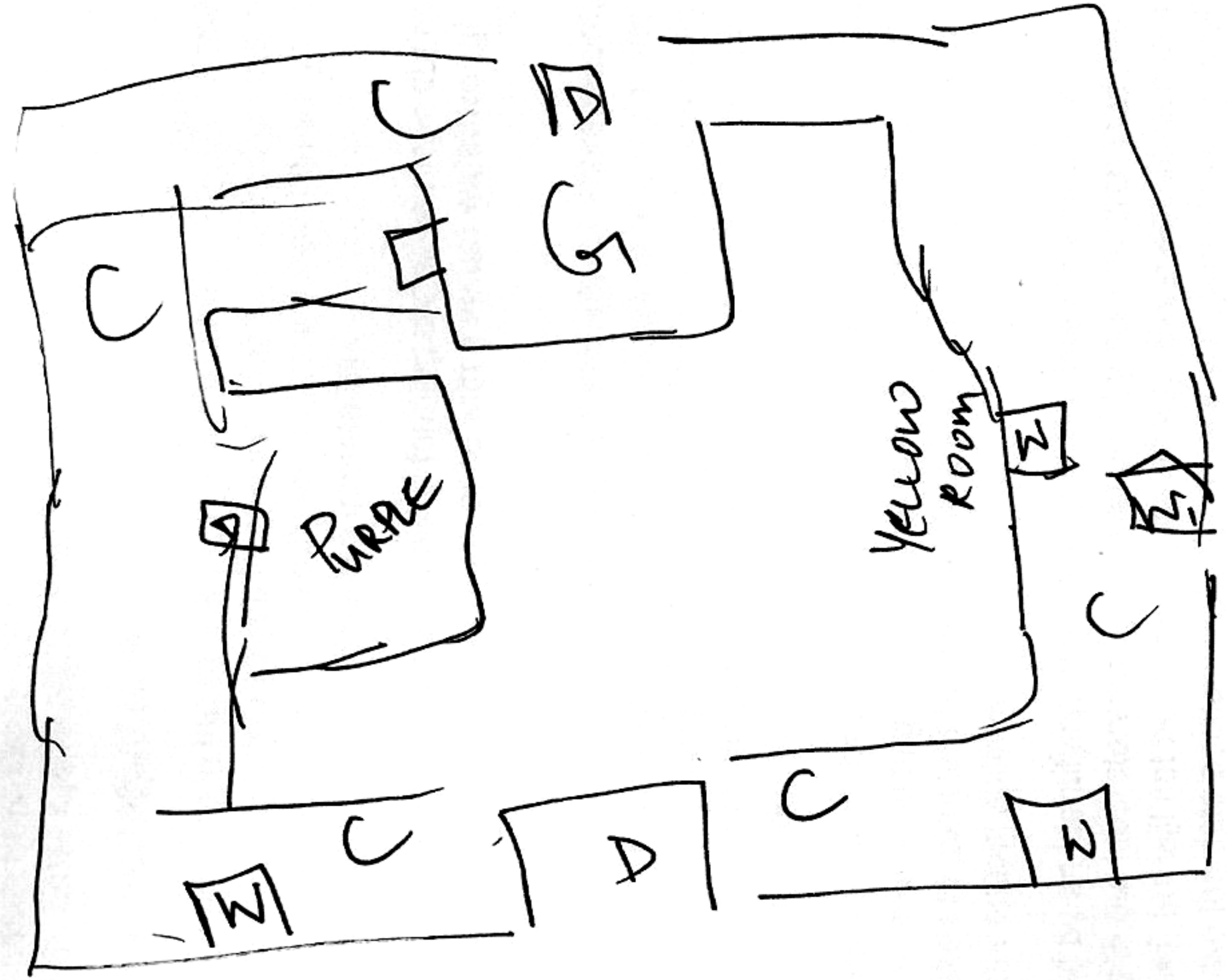

53 map 1 Rotational Video

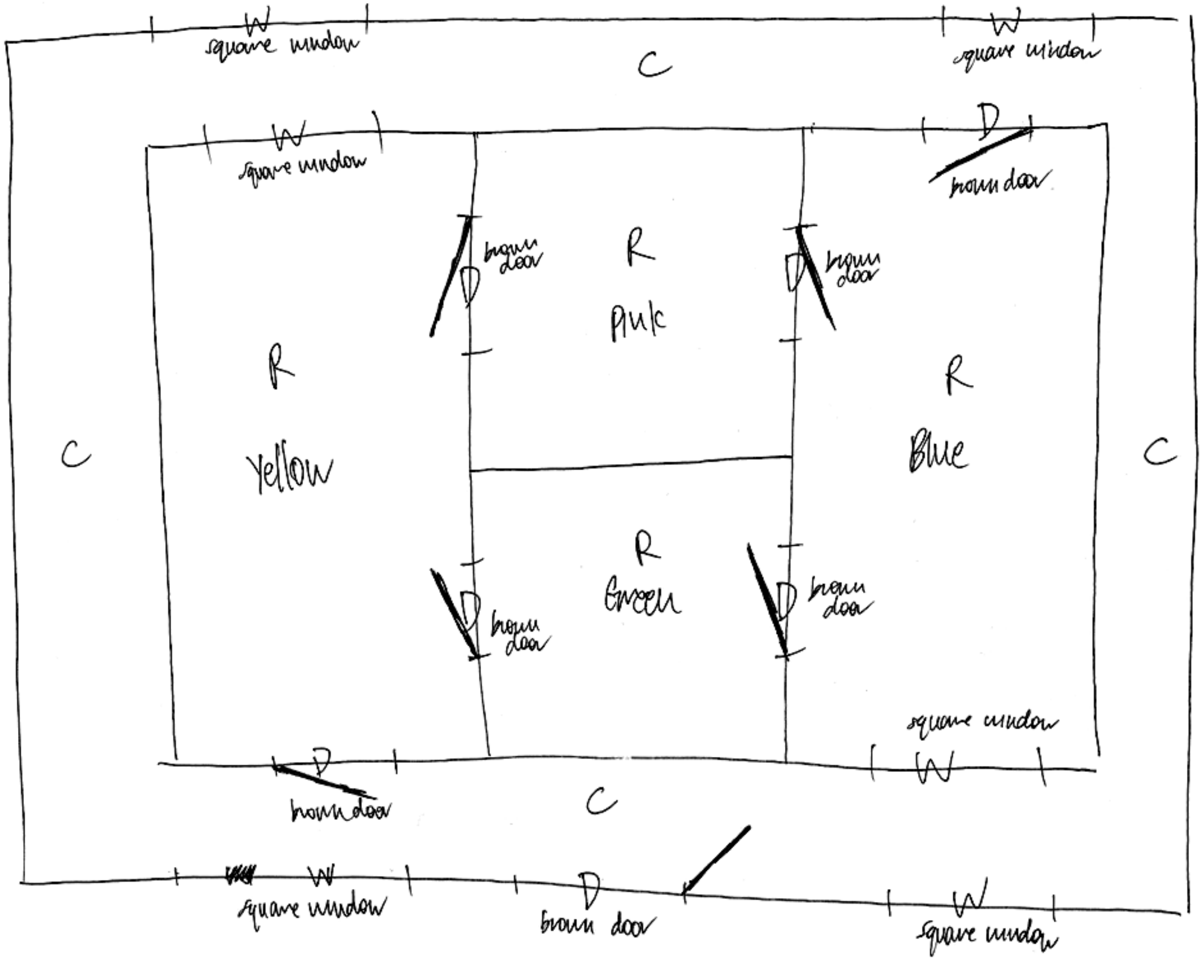

53 map 2 Mirror Written

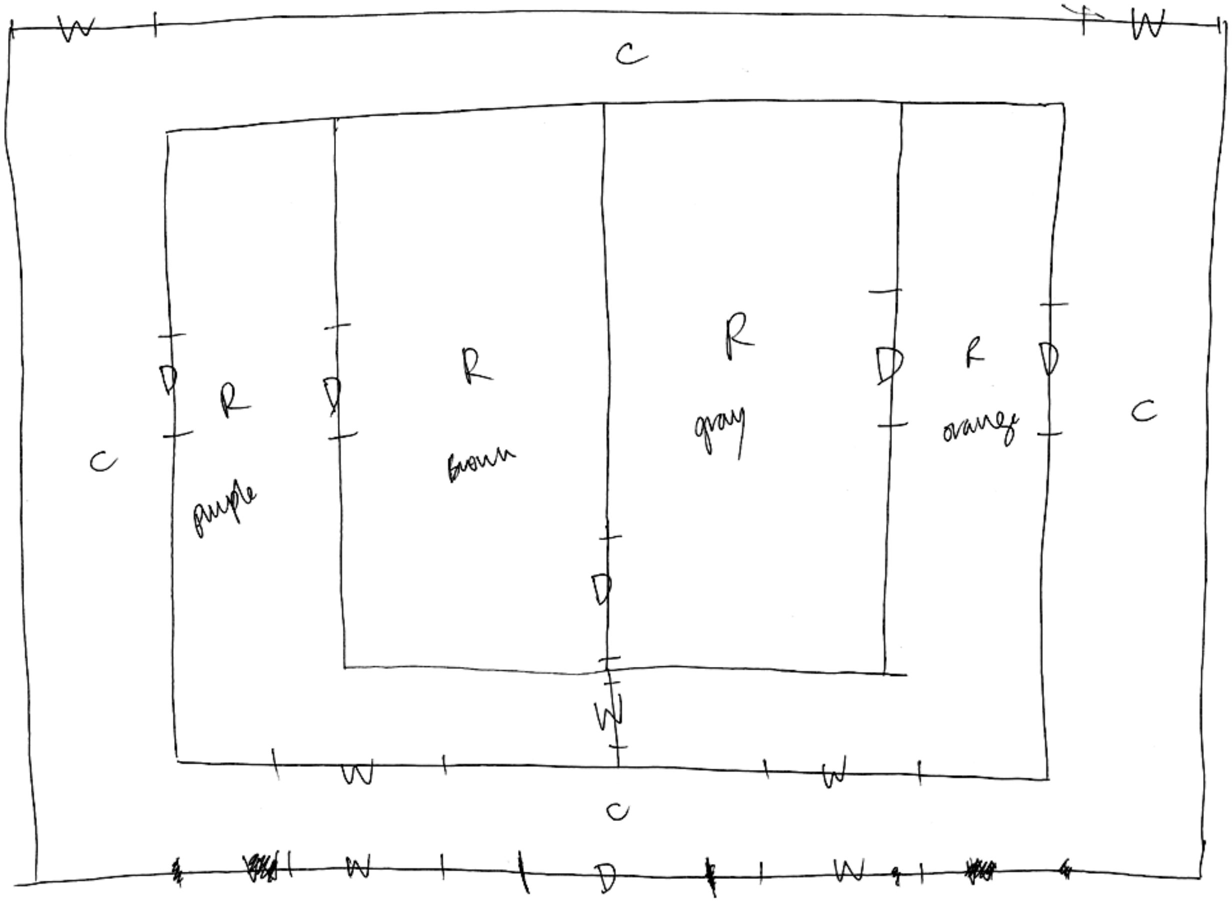

54 map 1 Mirror Written

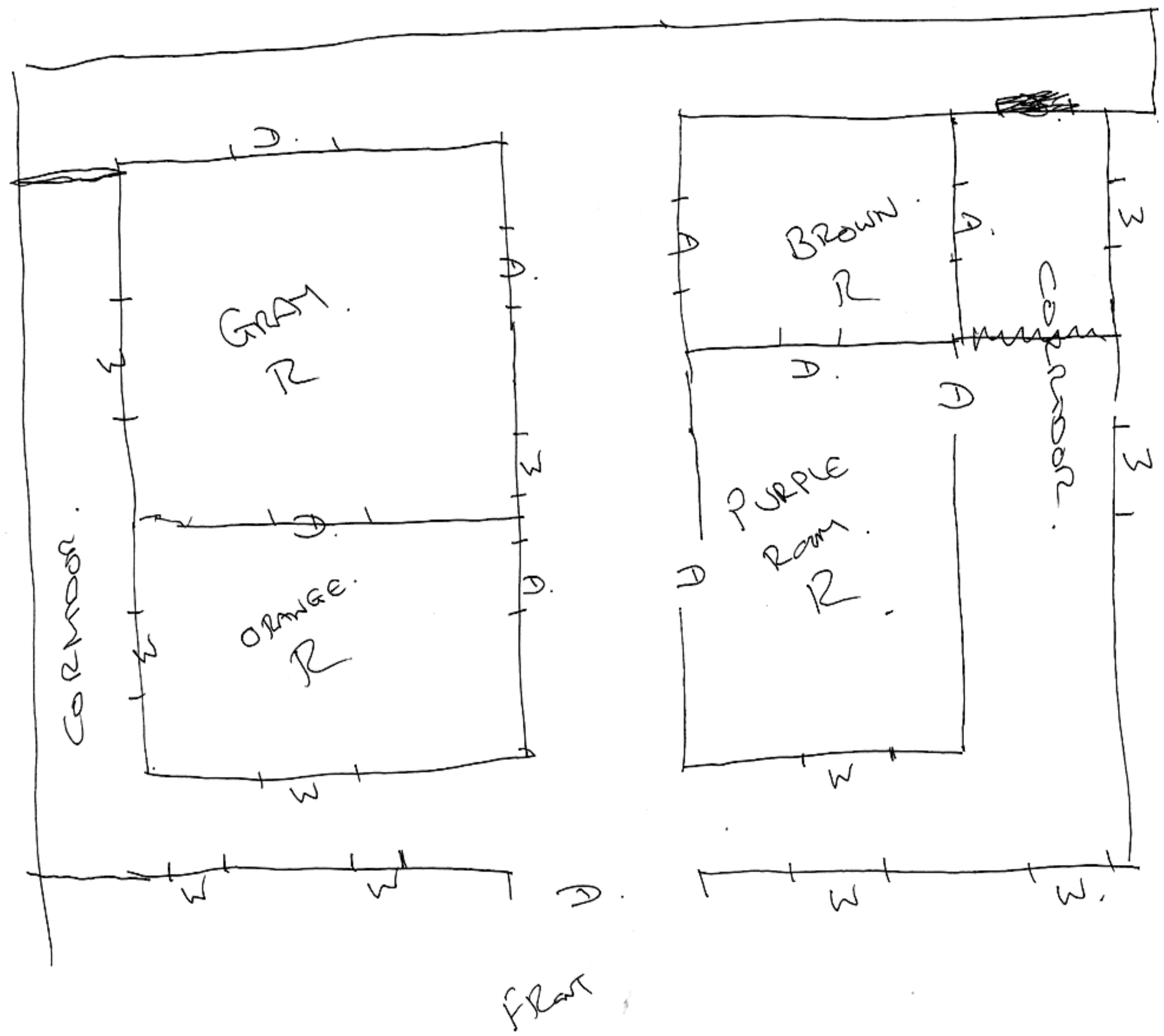

54 map 2 Rotational Video

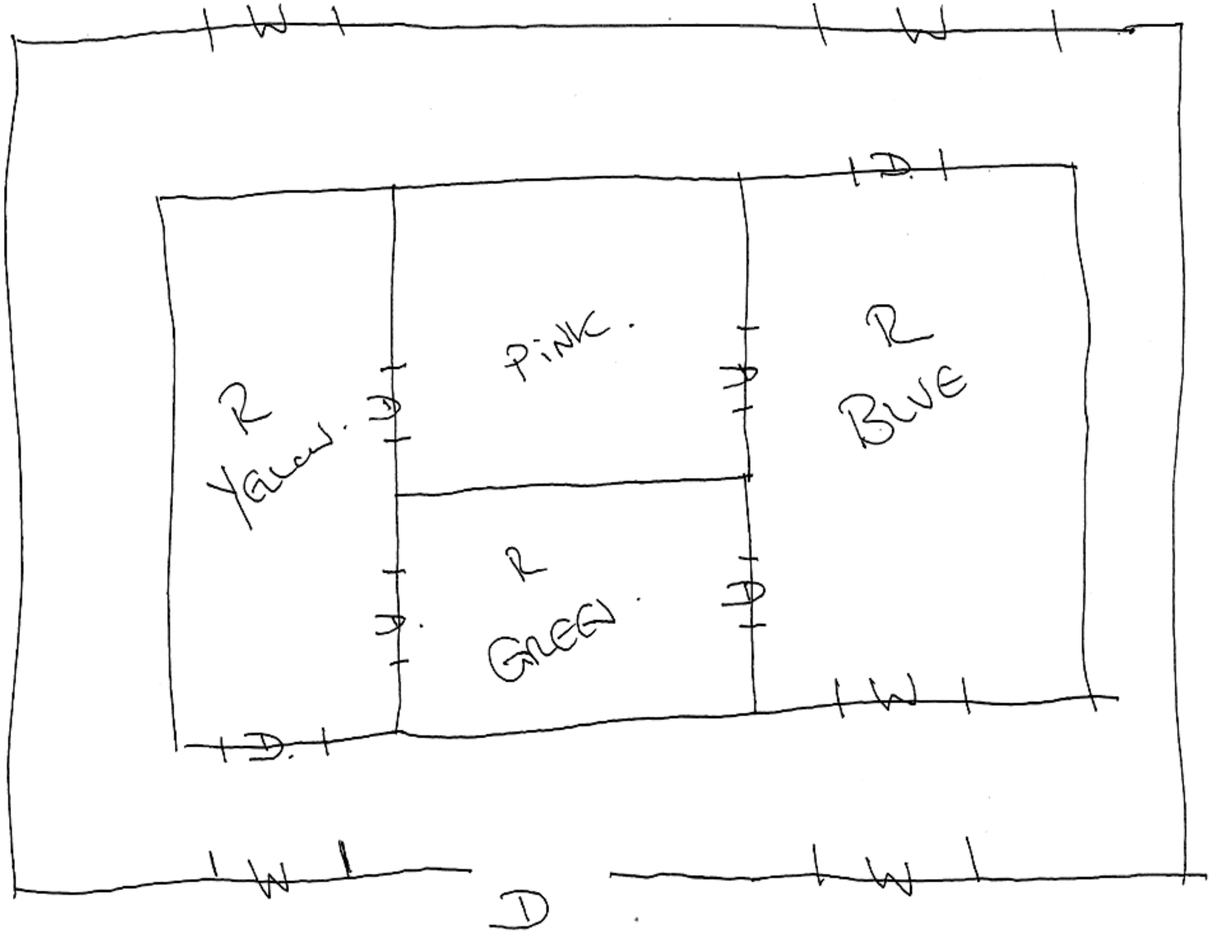

55 map 1 Mirror Video

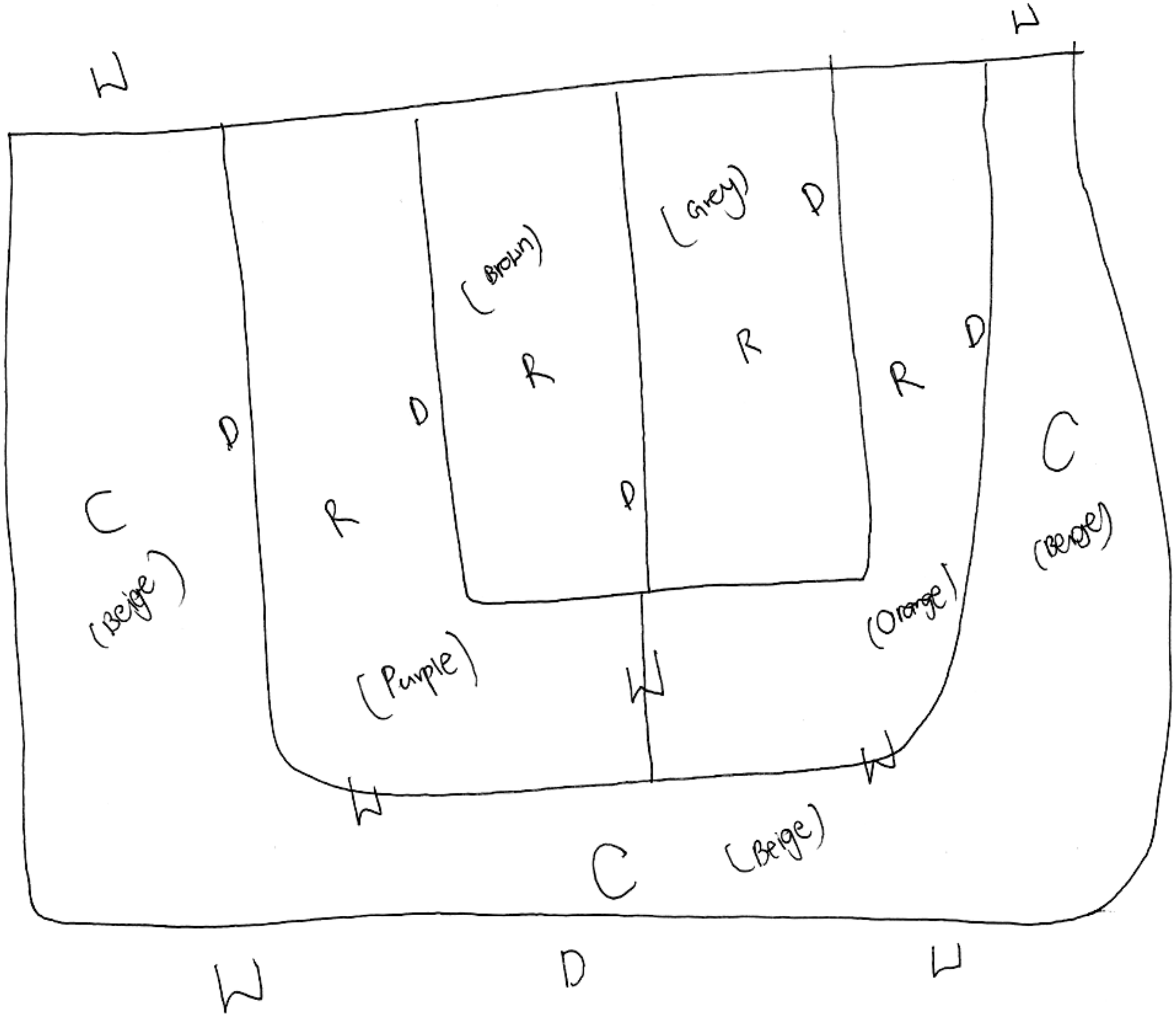

55 map 2 Rotational Written

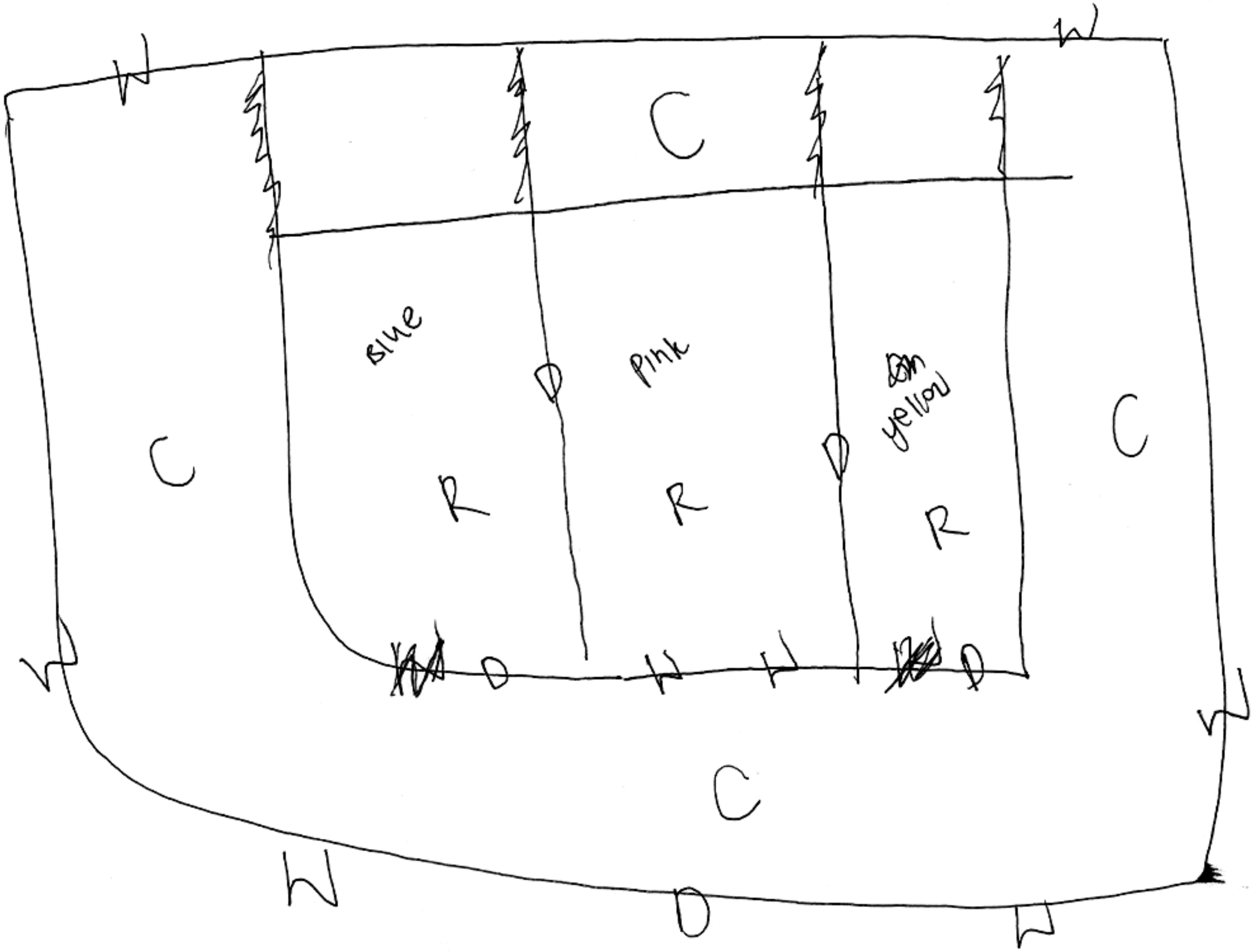

56 map 1 Mirror Written

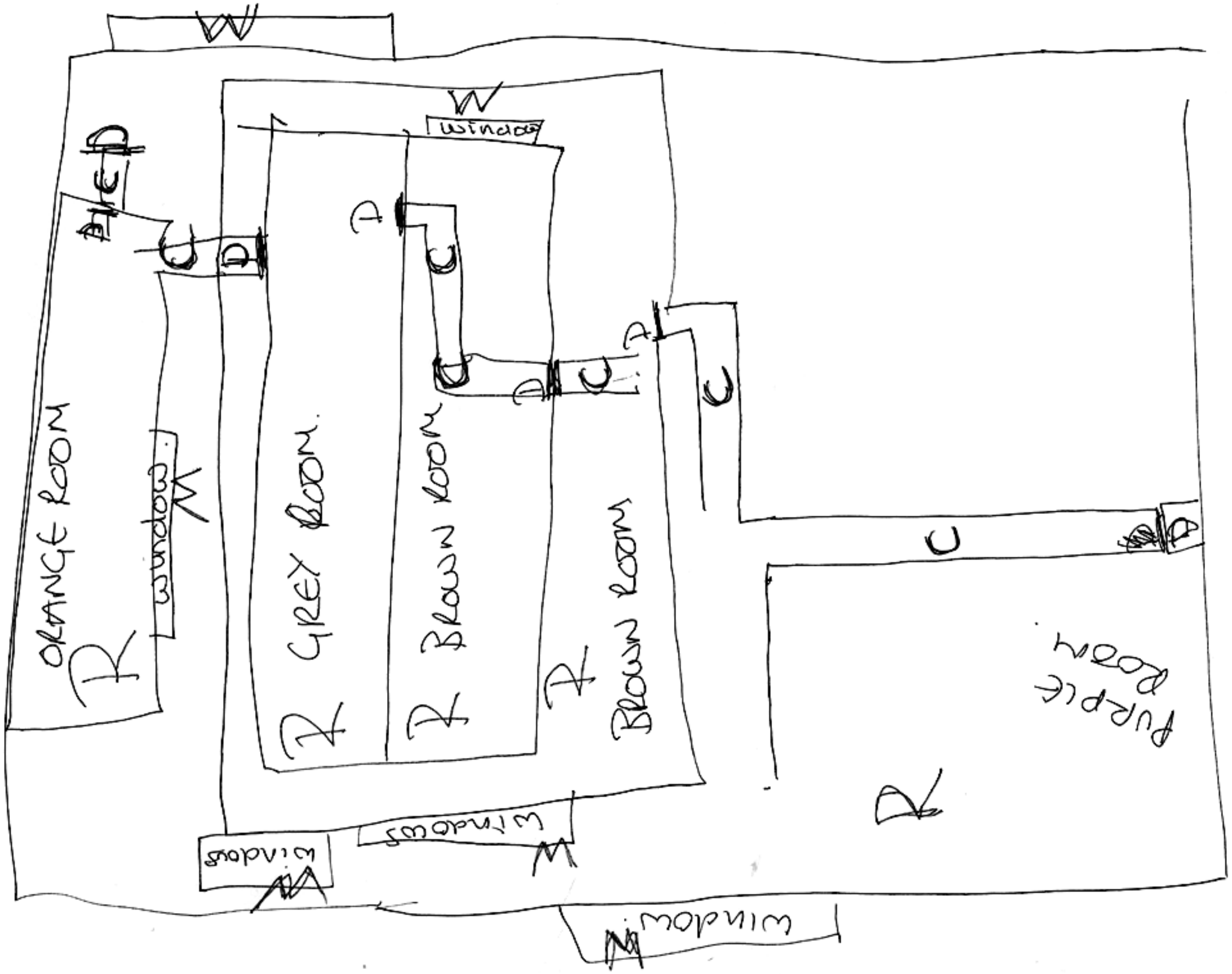

56 map 2 Rotational Video

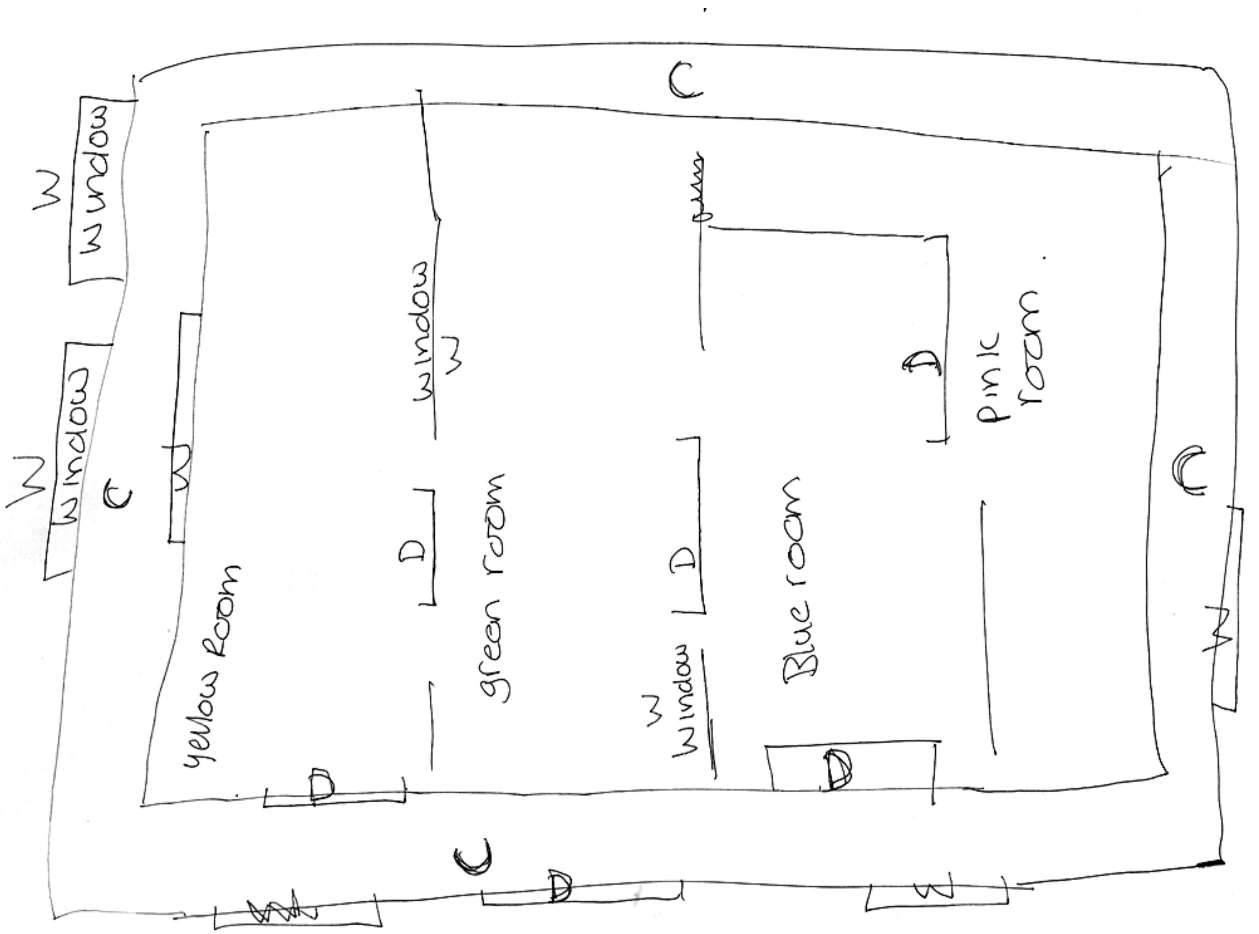

57 map 1 Rotational Video

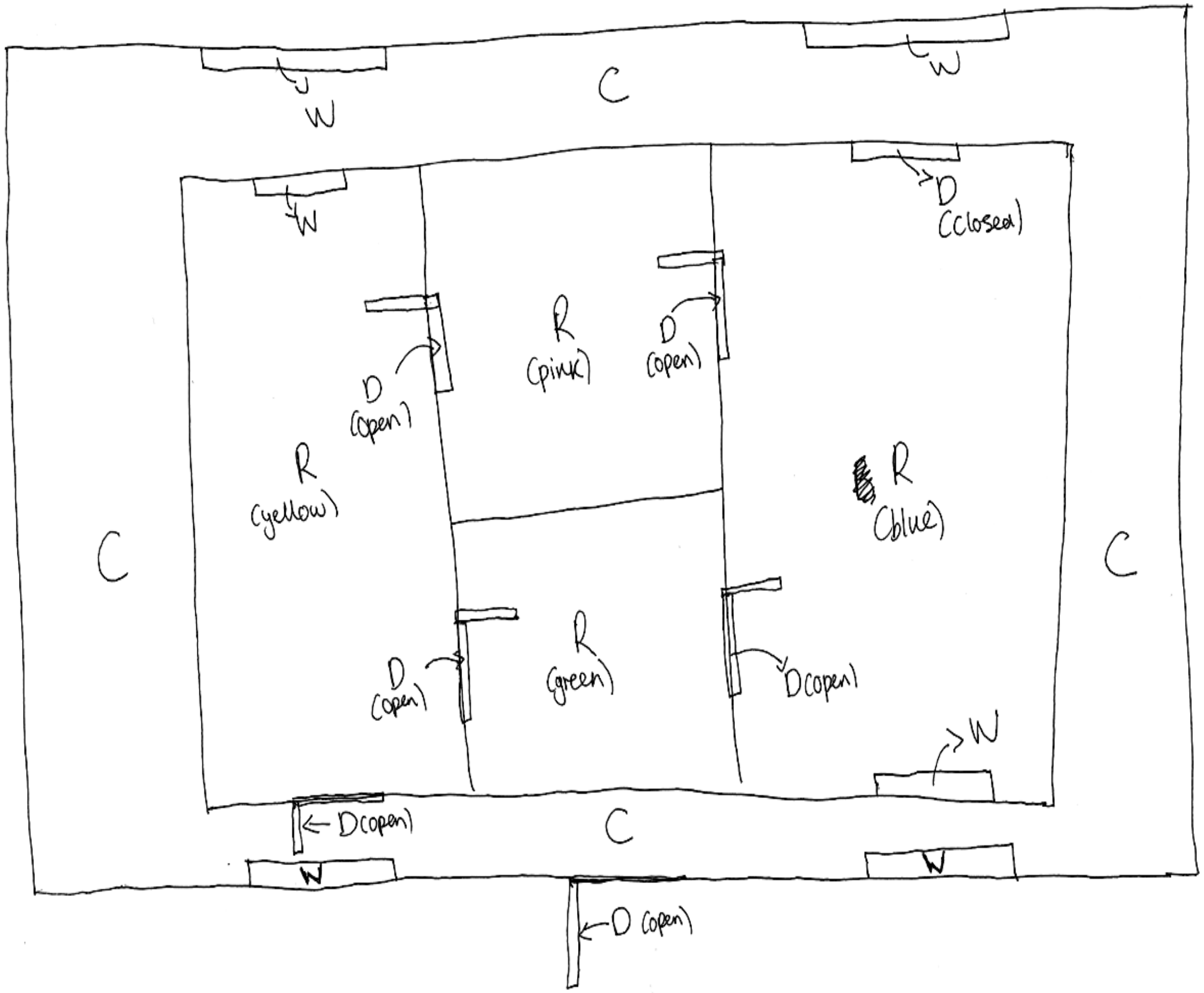

57 map 2 Mirror Written

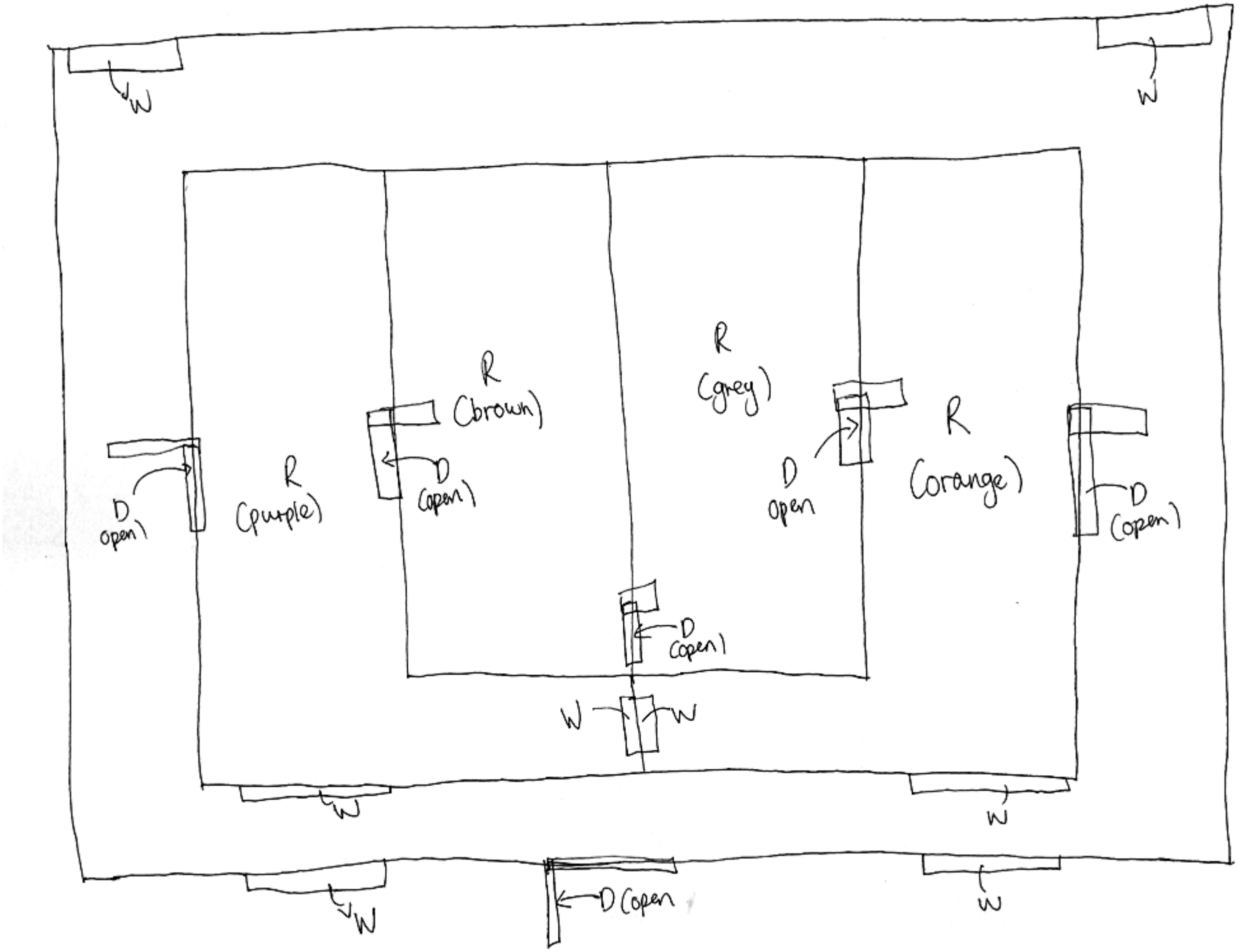

58 map 1 Rotational Written

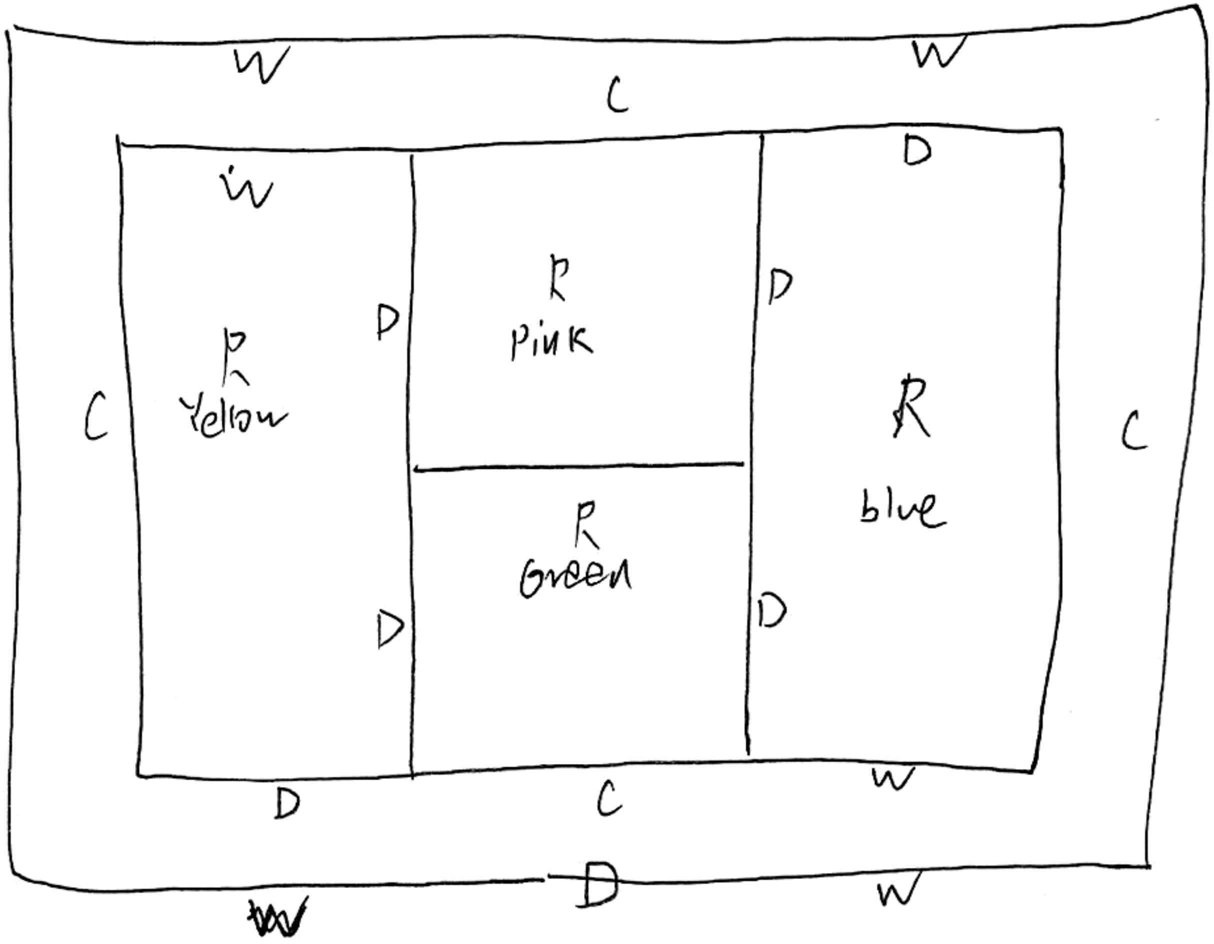

58 map 2 Mirror Video

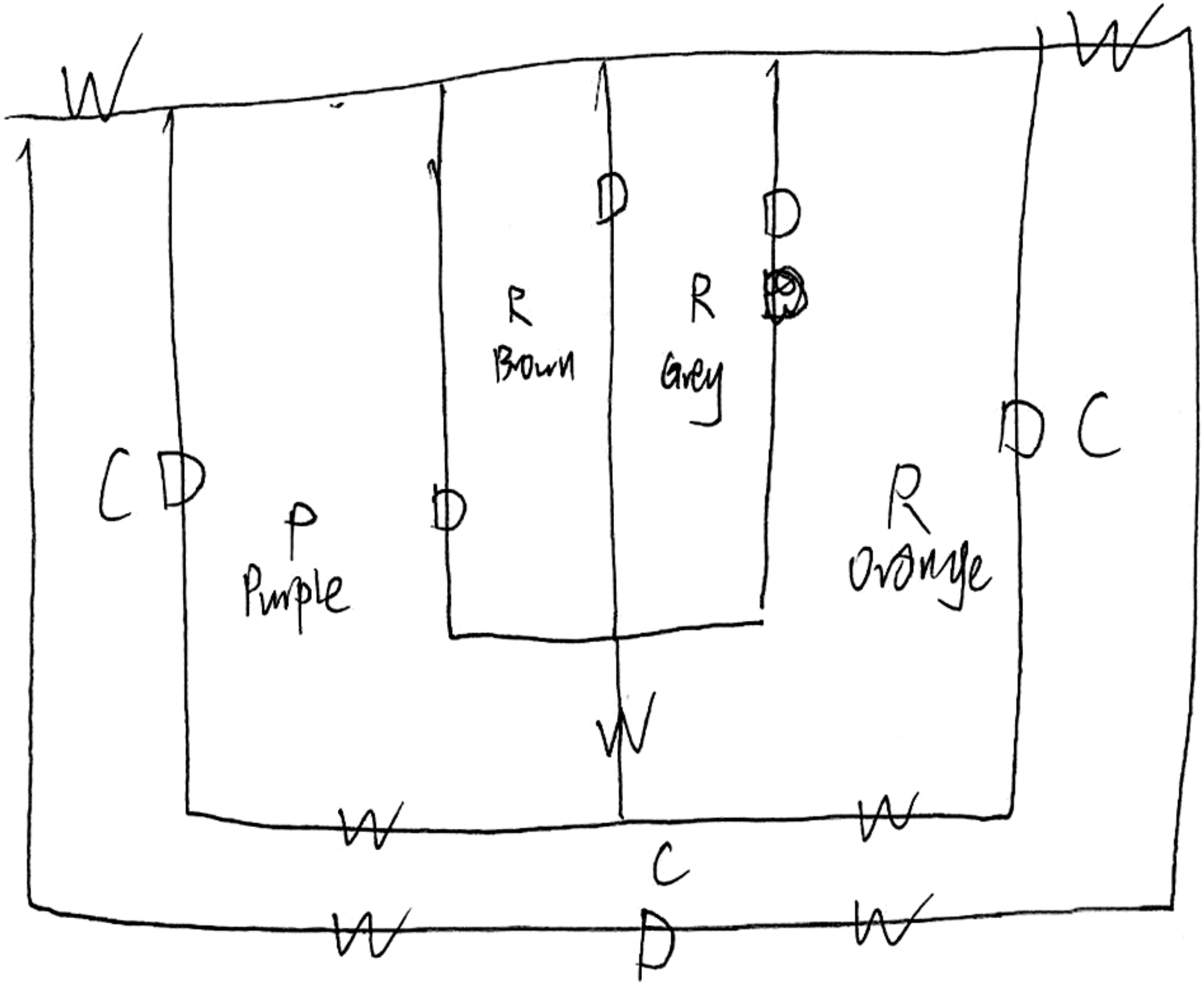

59 map 1 Mirror Written

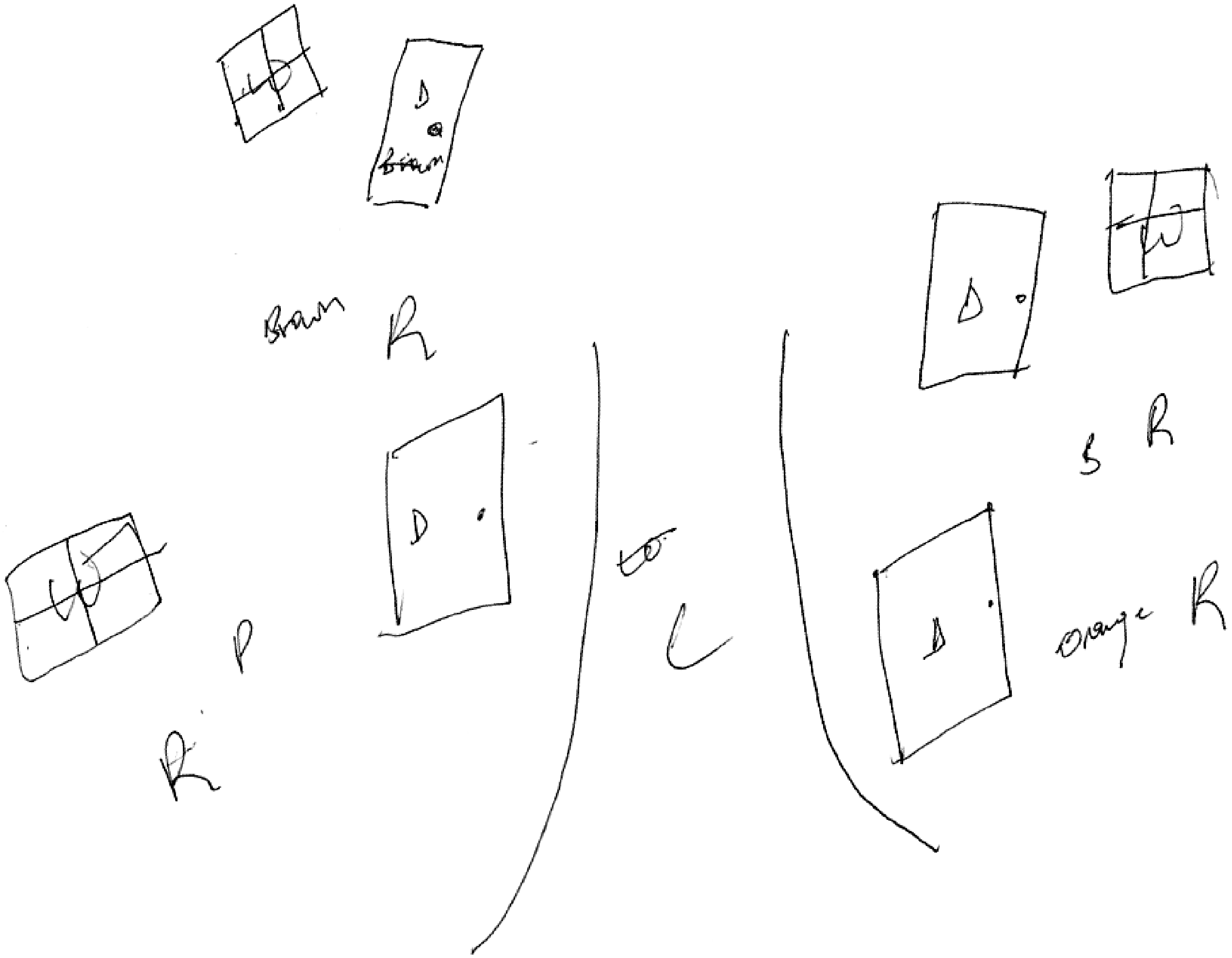

59 map 2 Rotational Video

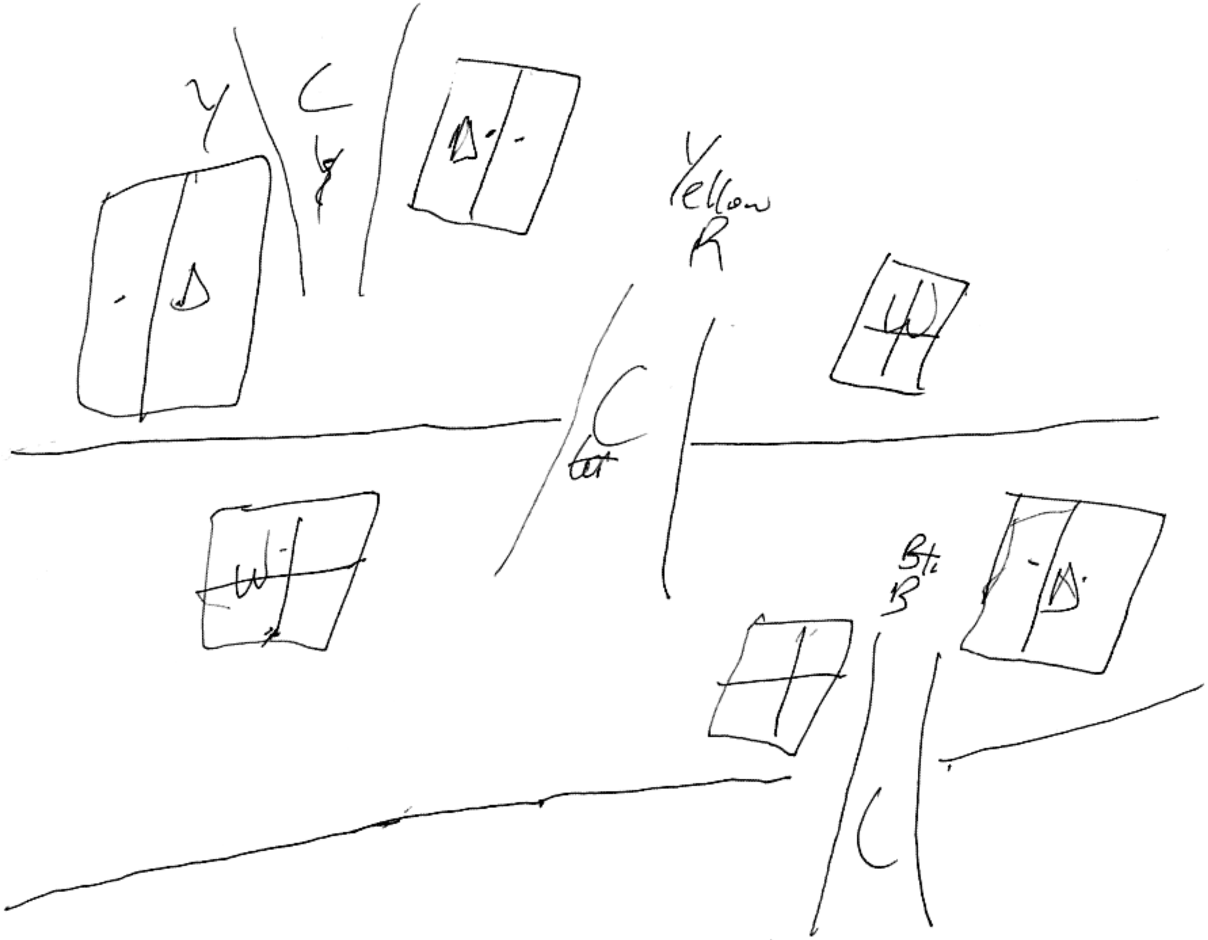

60 map 1 Mirror Video

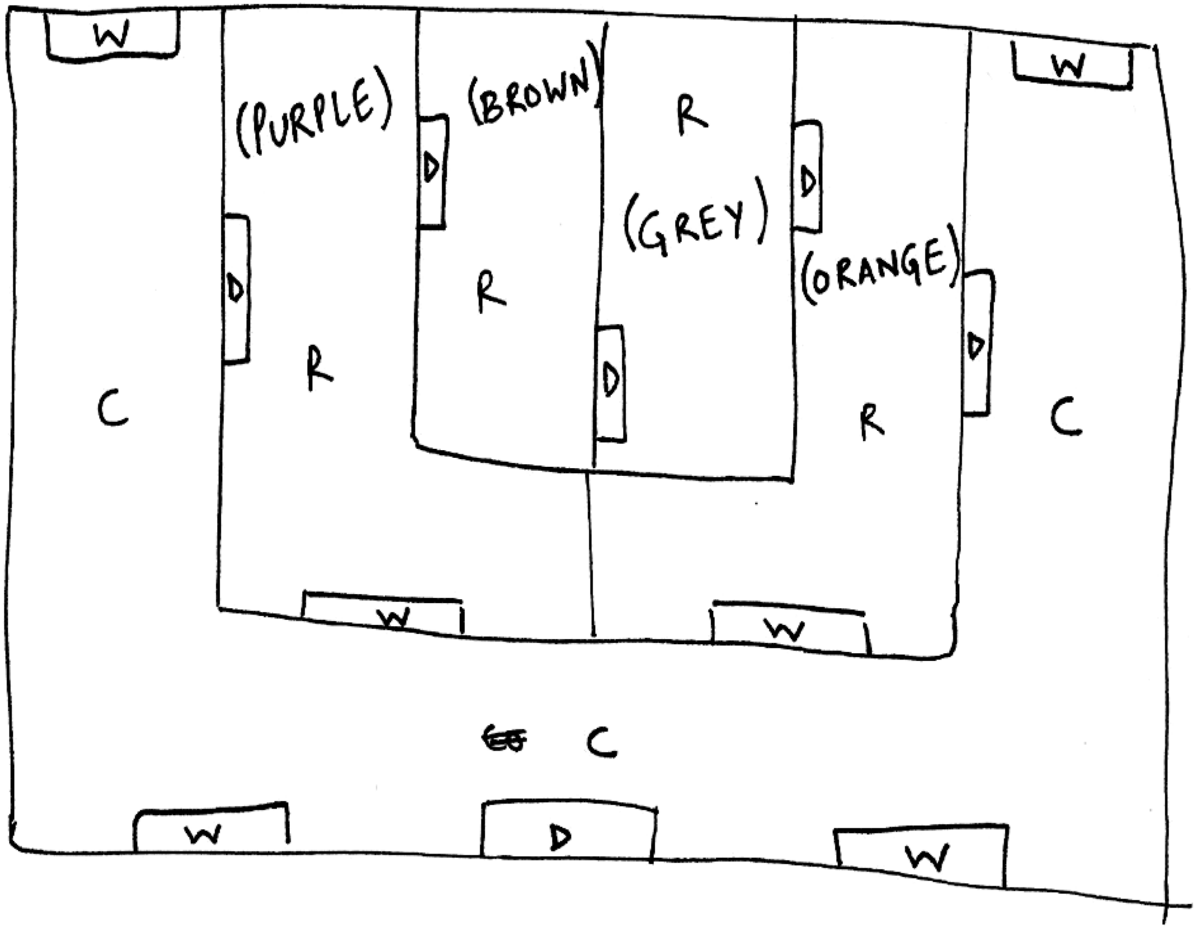

60 map 2 Rotational Written

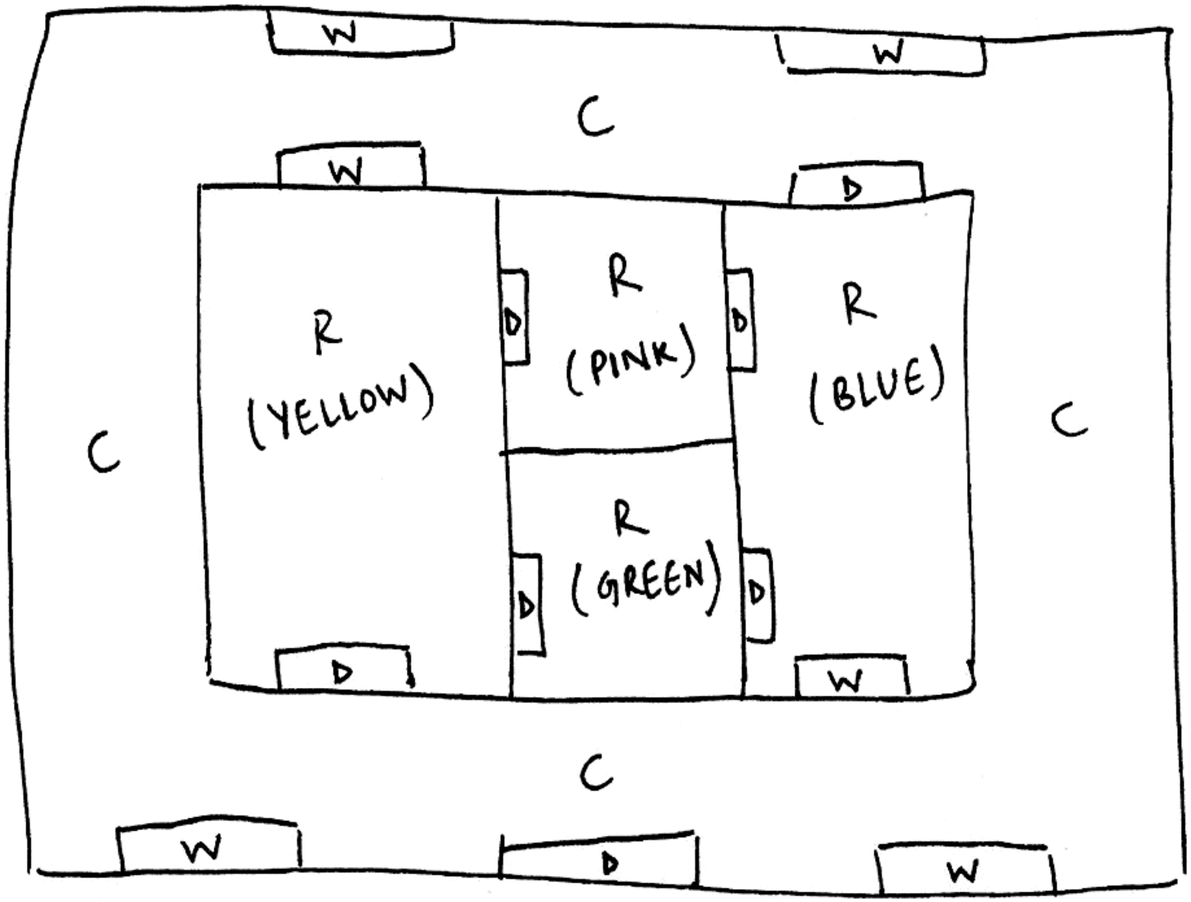

61 map 1 Rotational Written

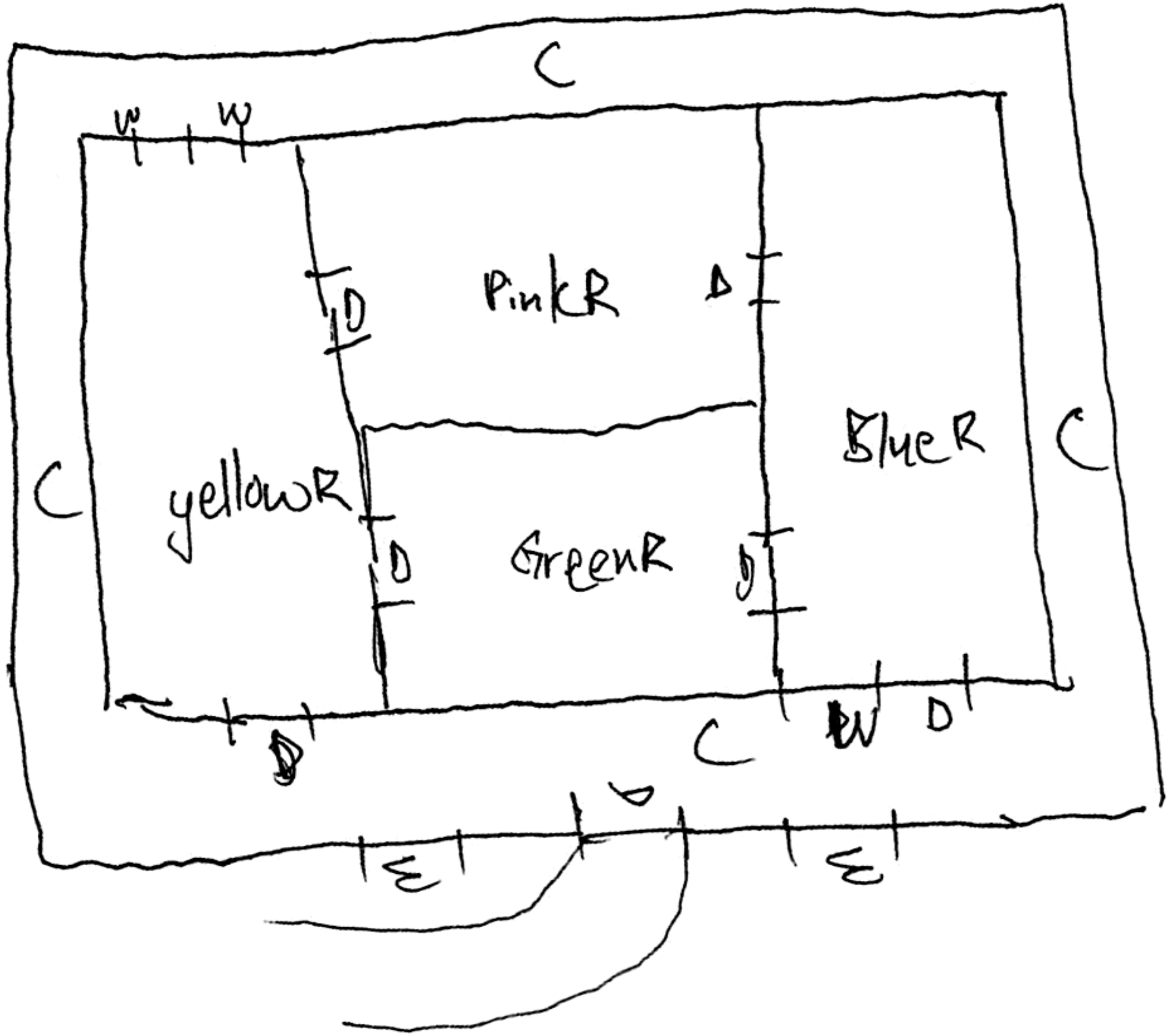

61 map Mirror Video 2

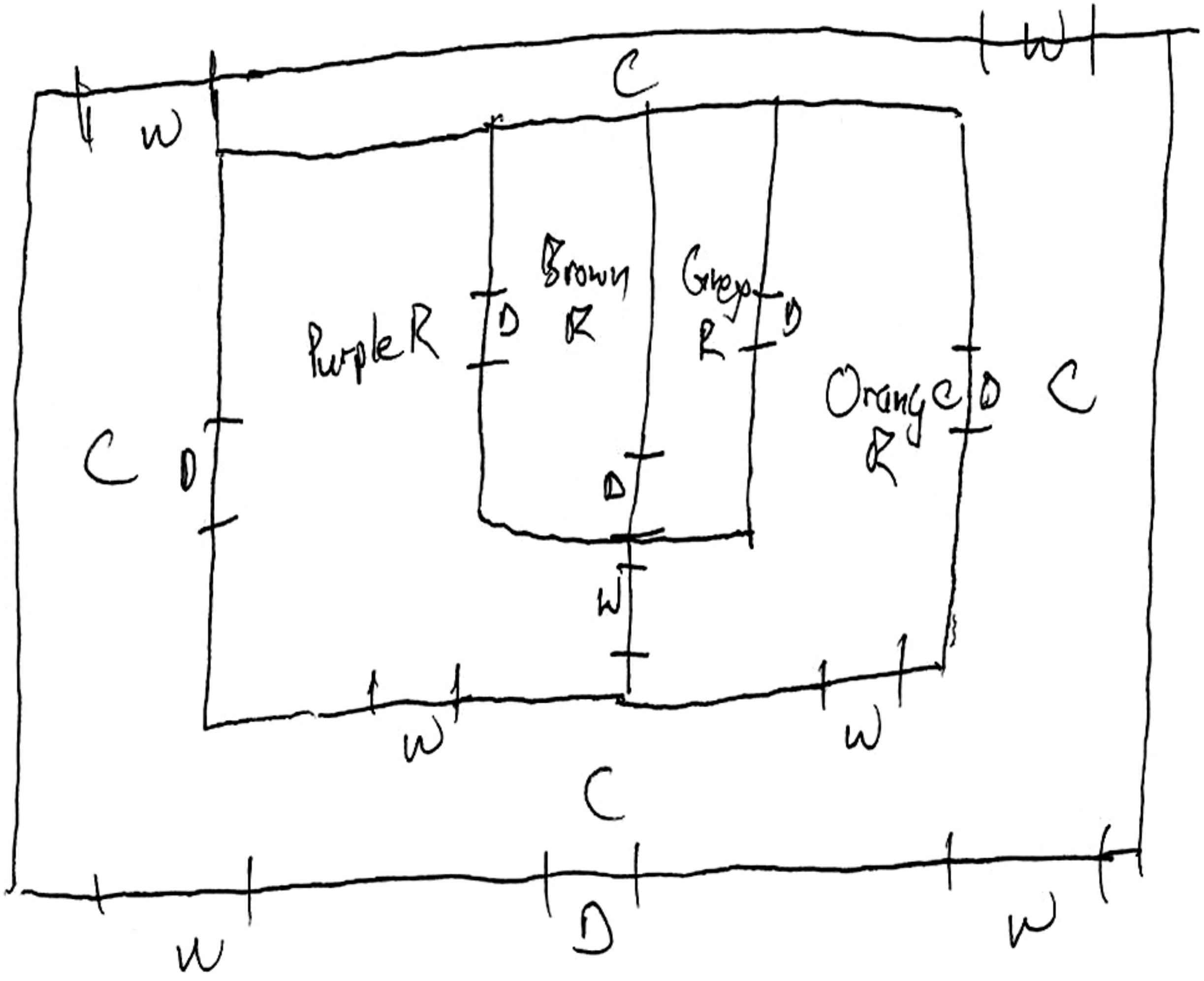

62 map 1 Rotational Video

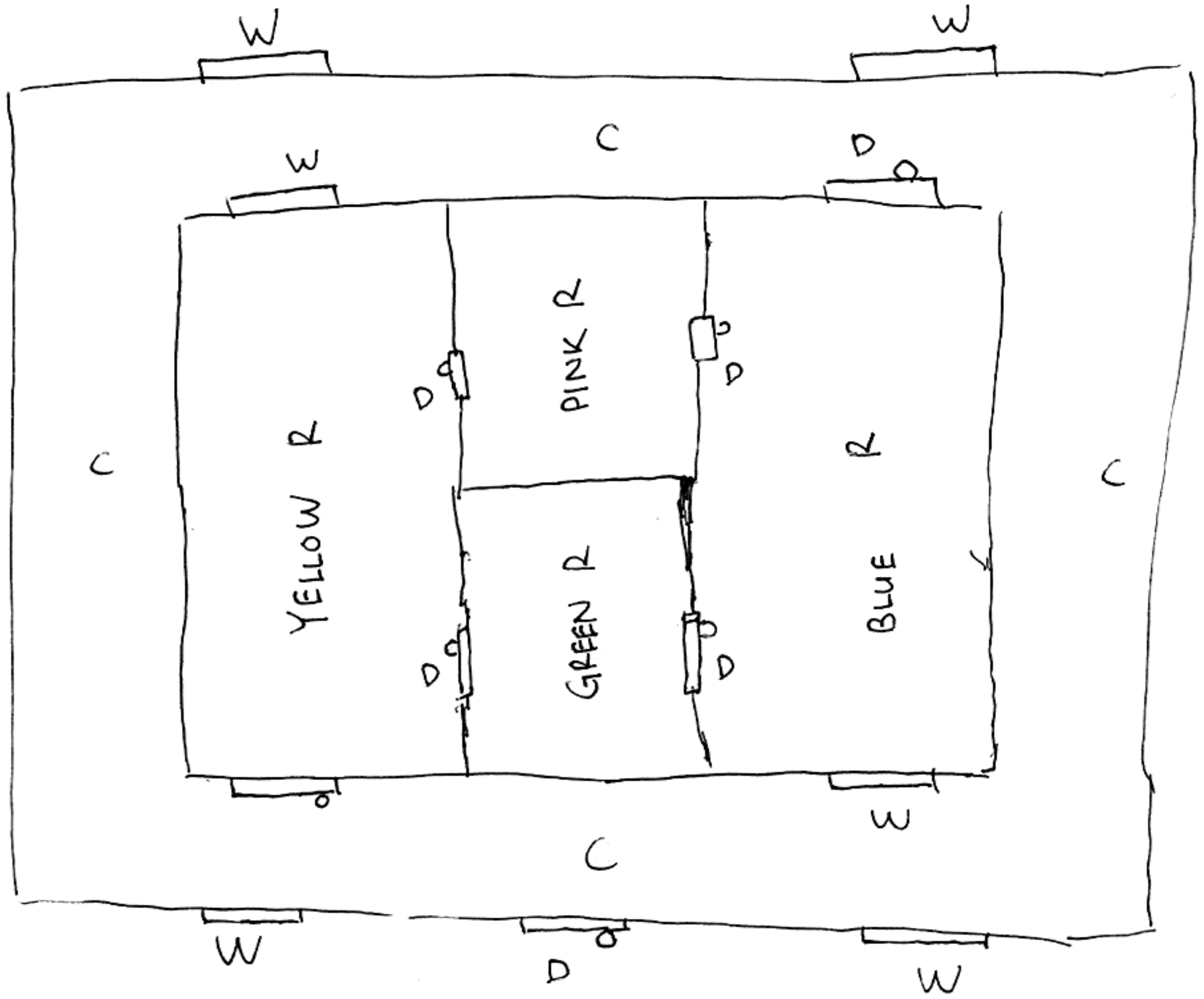

62 map 2 Mirror Written

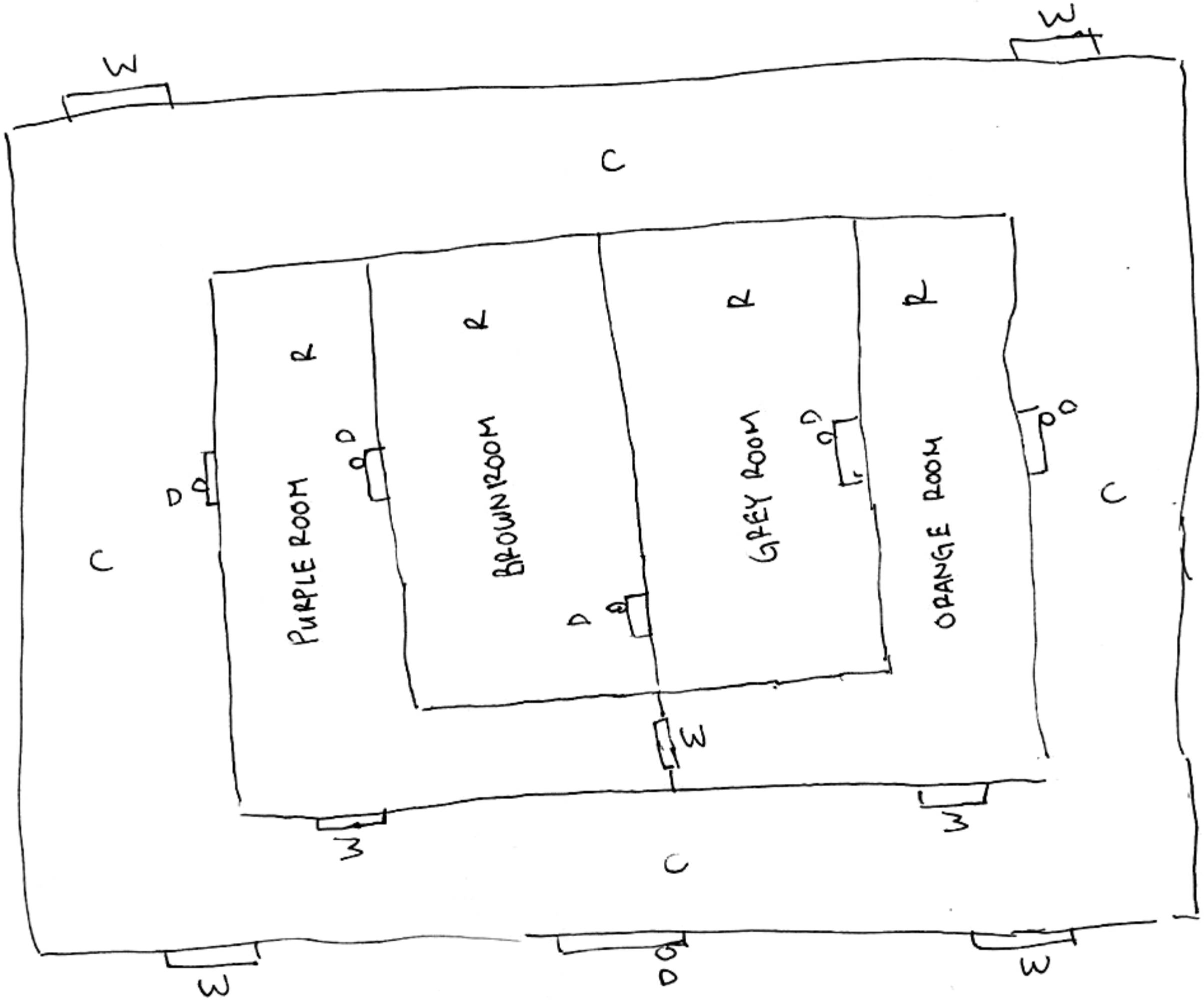

63 map 1 Mirror Written

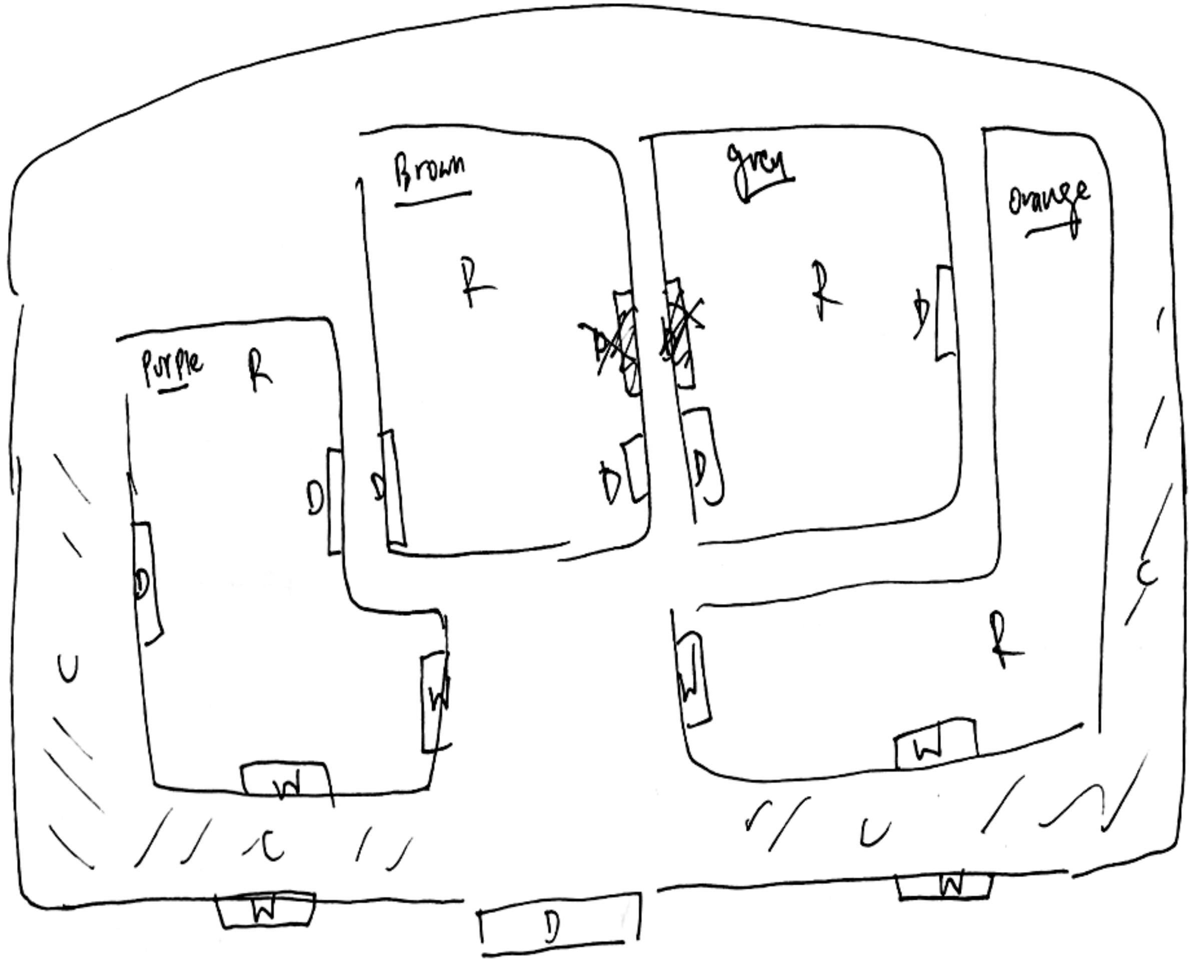

63 map 2 Rotational Video

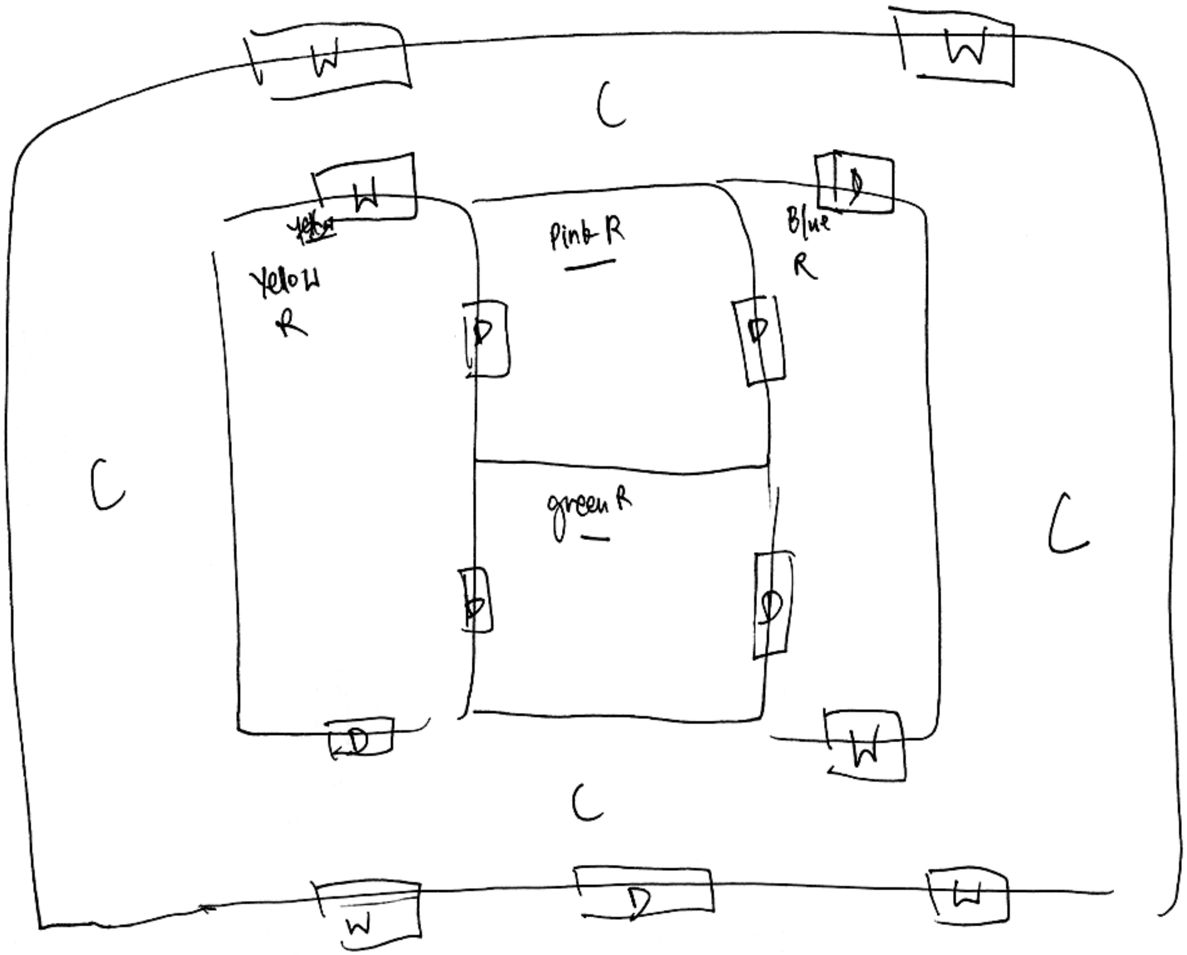

64 map 1

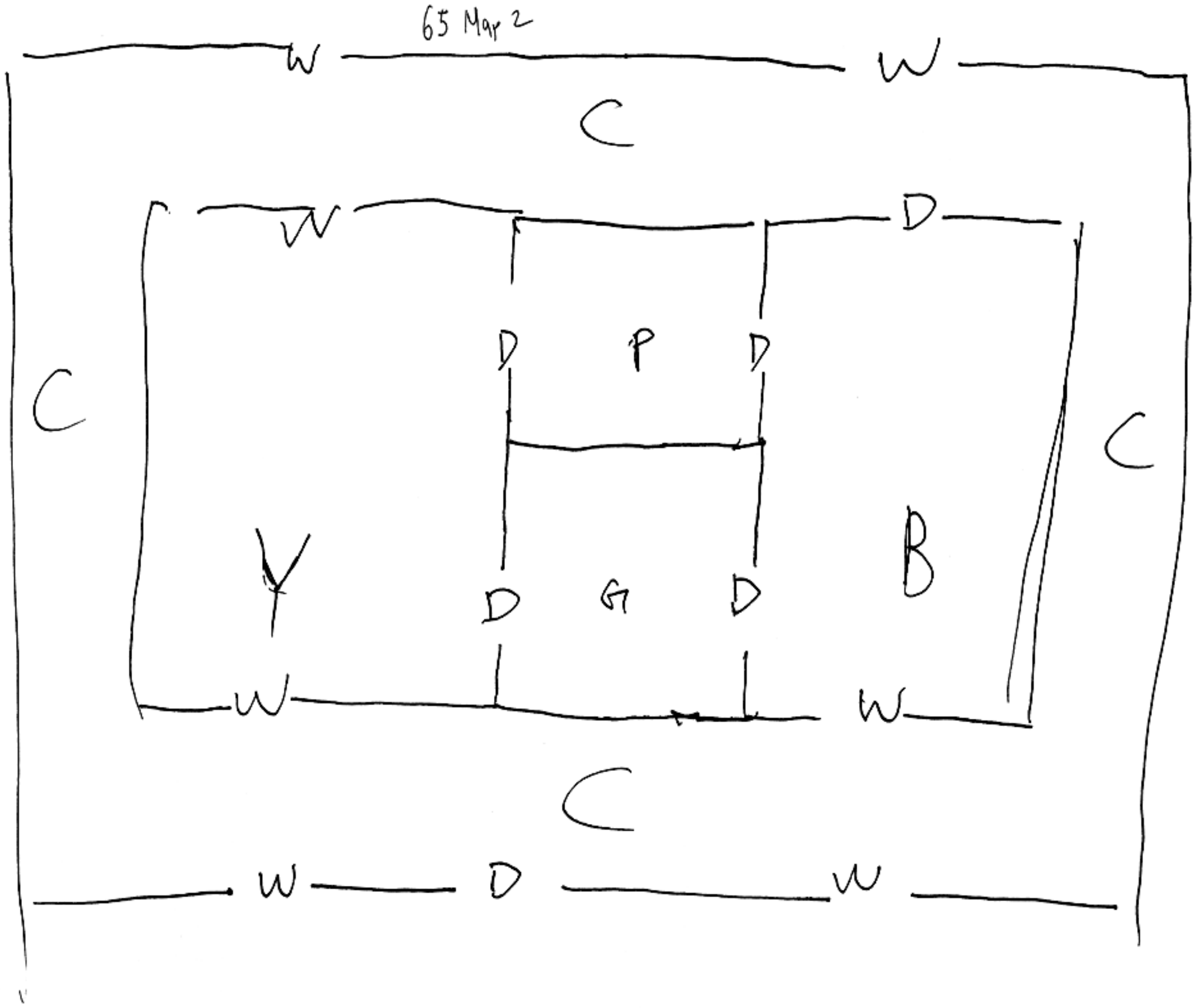

64 map 2

Missing

65 map 1 Mirror Video

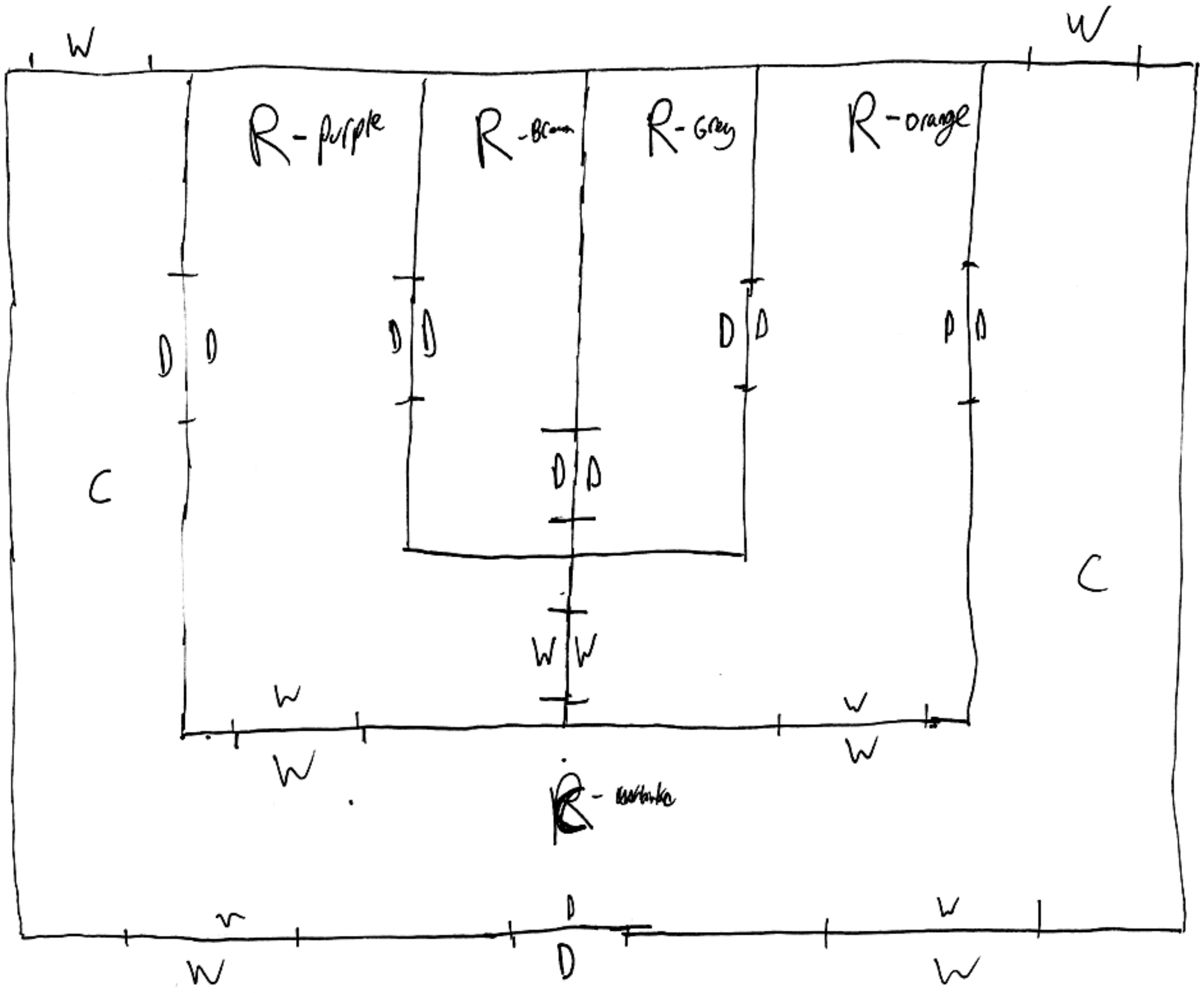

65 map 2 Rotational Written

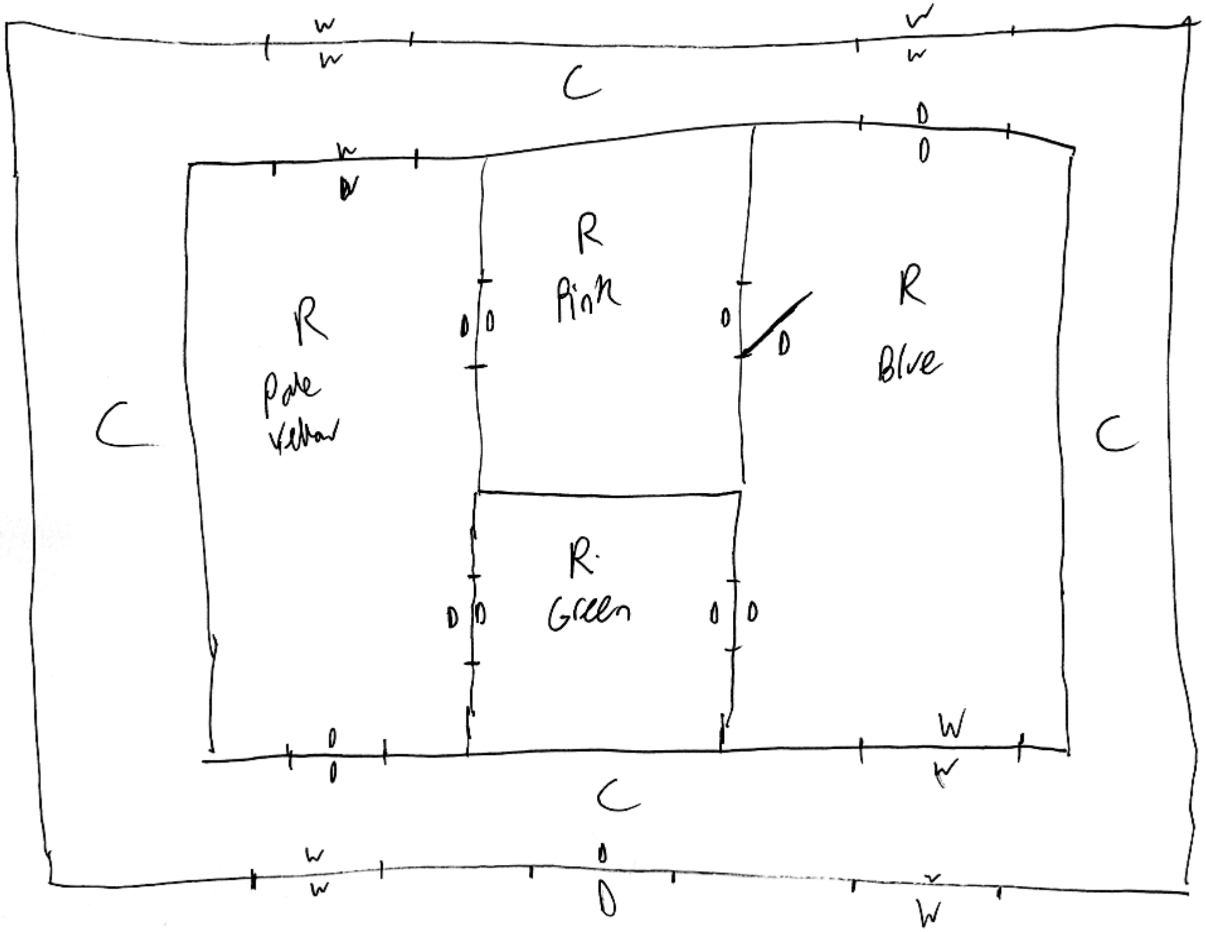

66 map 1 Rotational Written

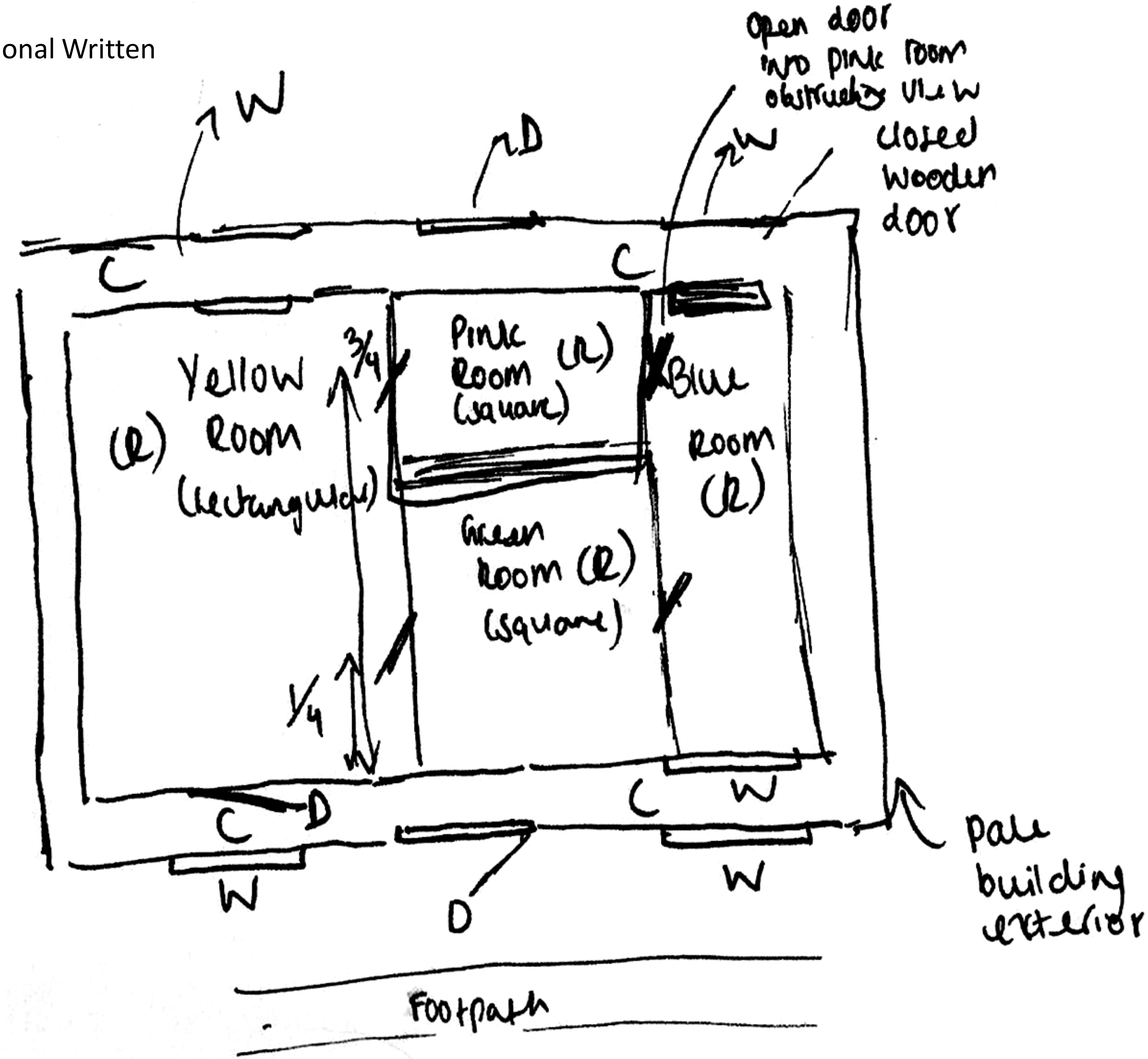

66 map 2 Mirror Video

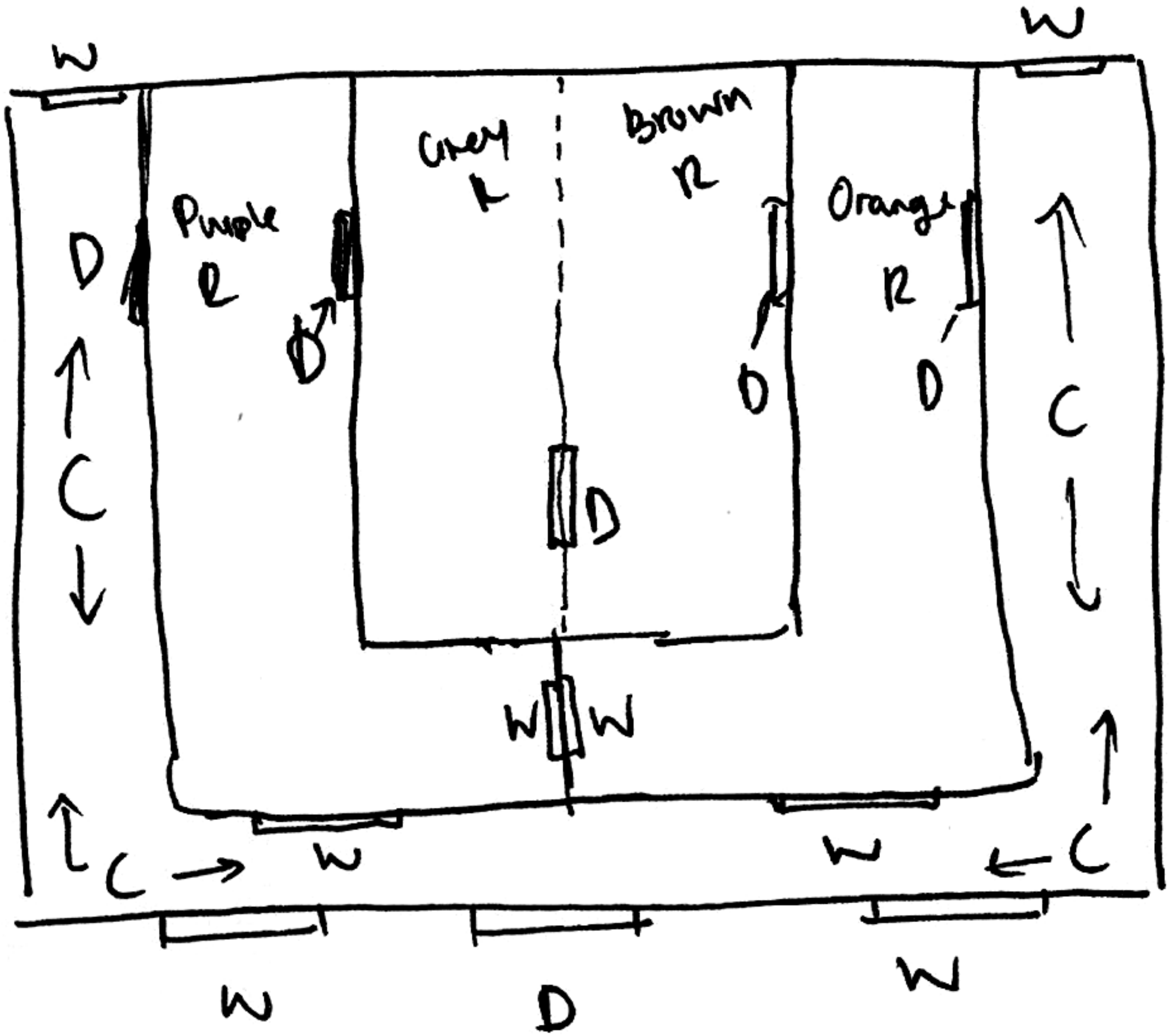

67 map 1 Rotational Written

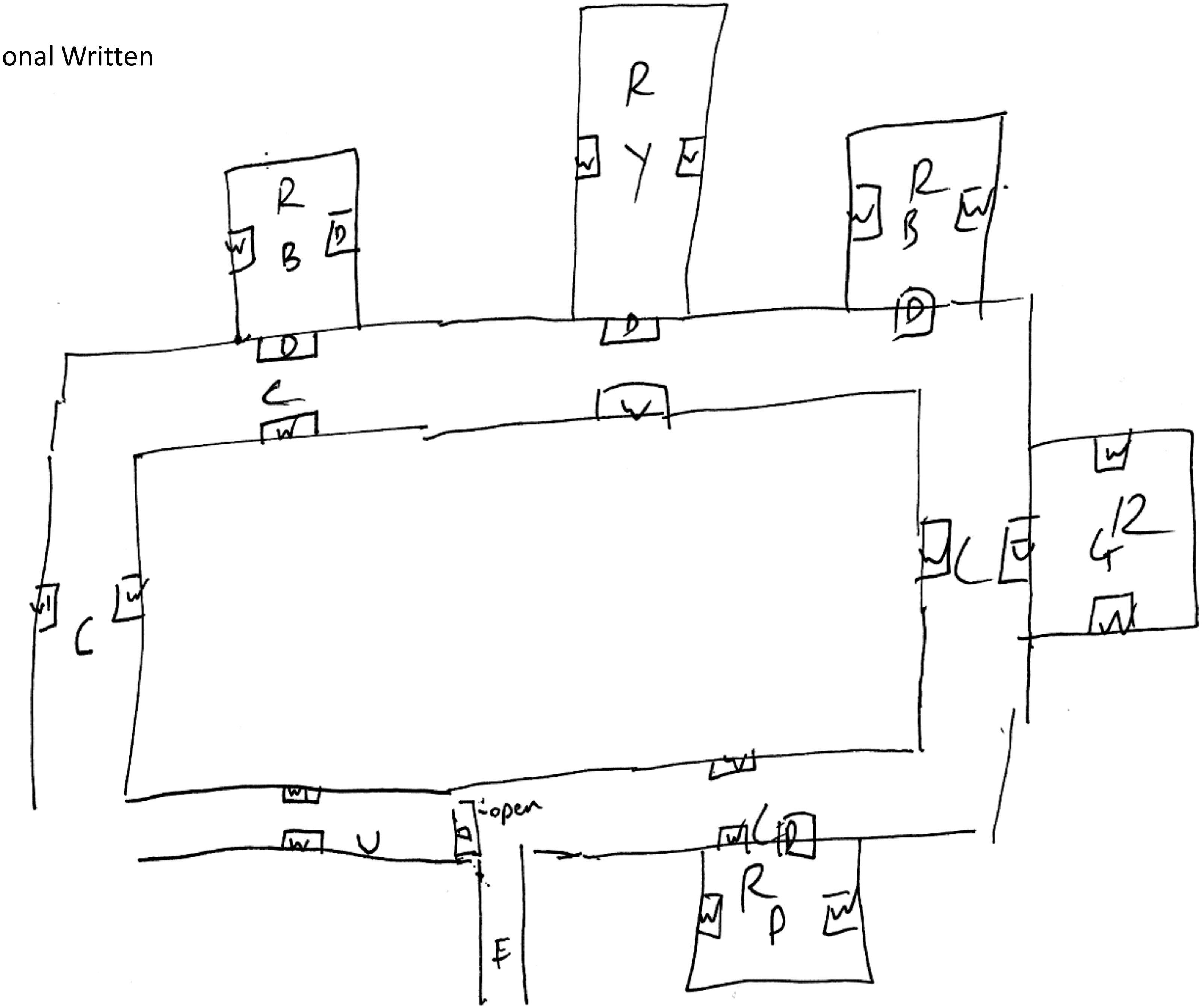

67 map 2 Mirror Video

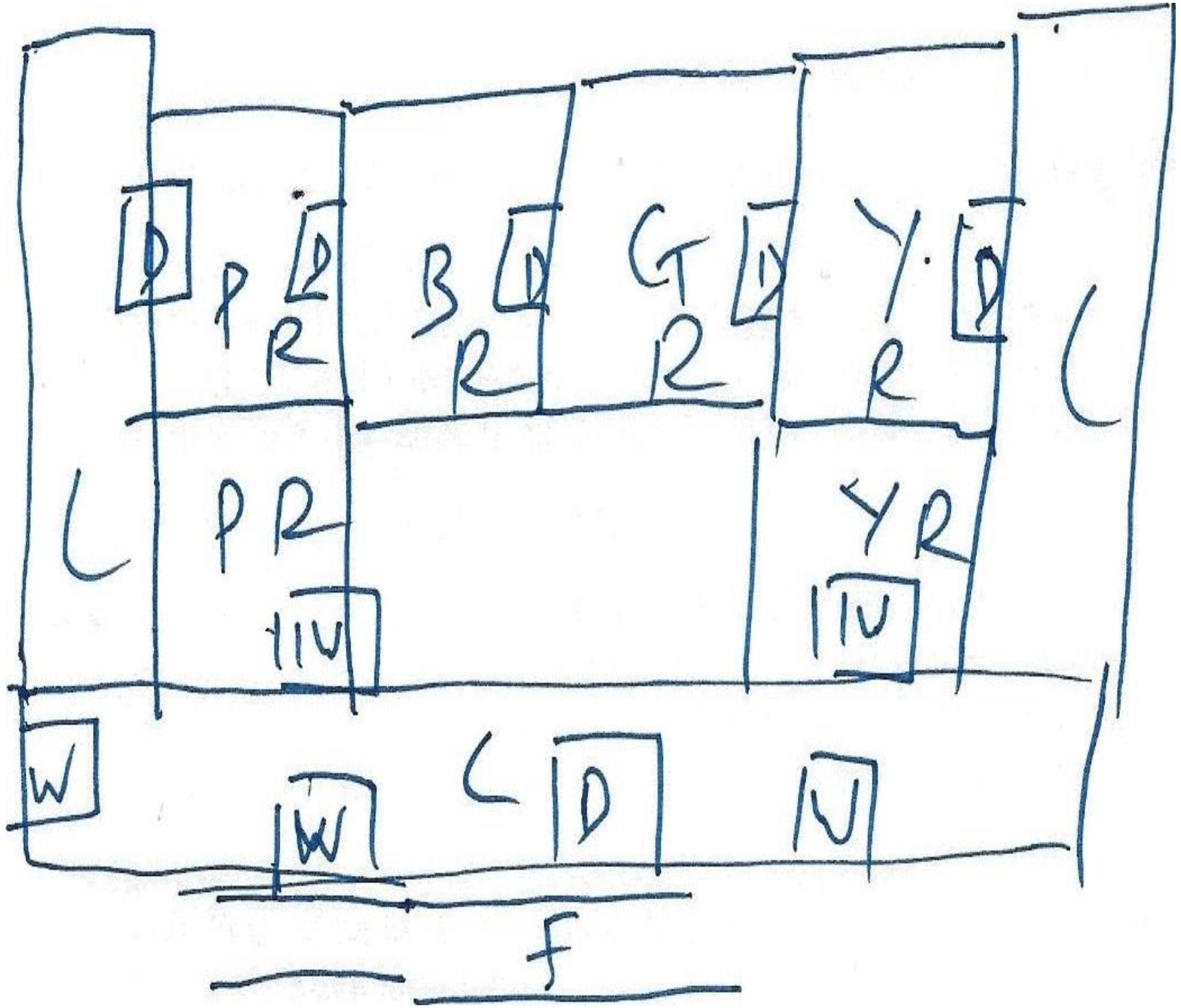

68 map 1 Mirror Written

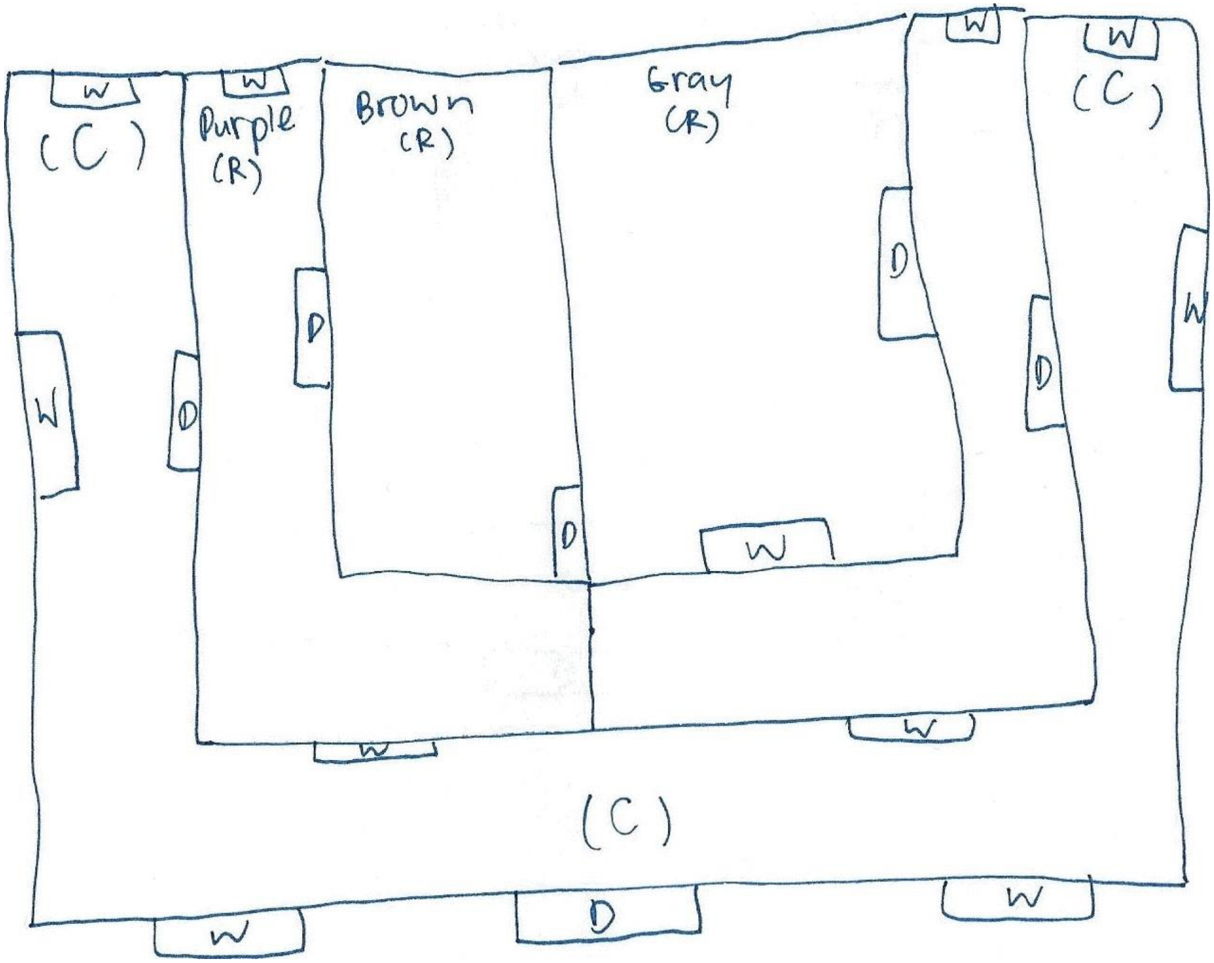

68 map 2Rotational Video

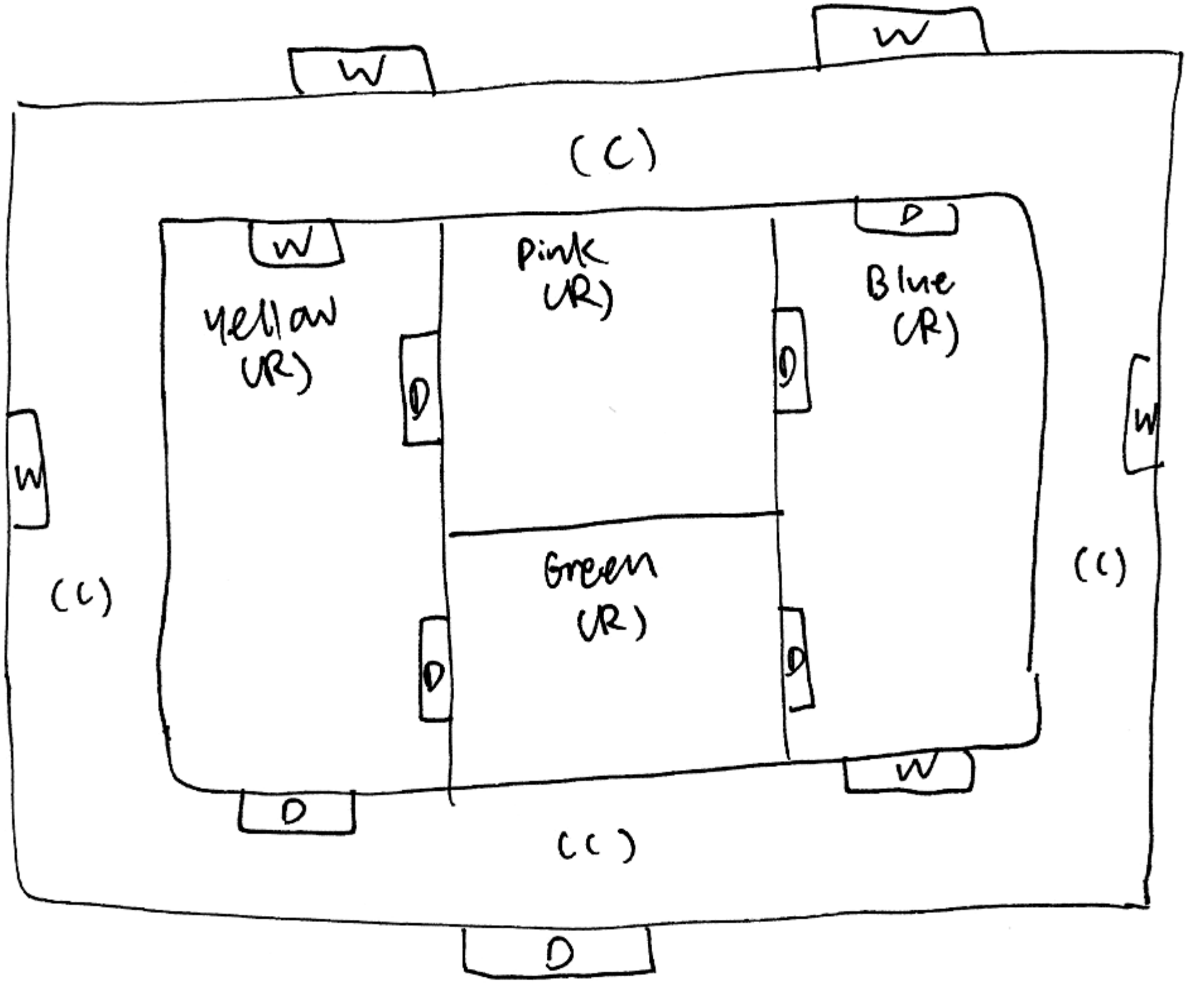

69 map 1 Rotational Video

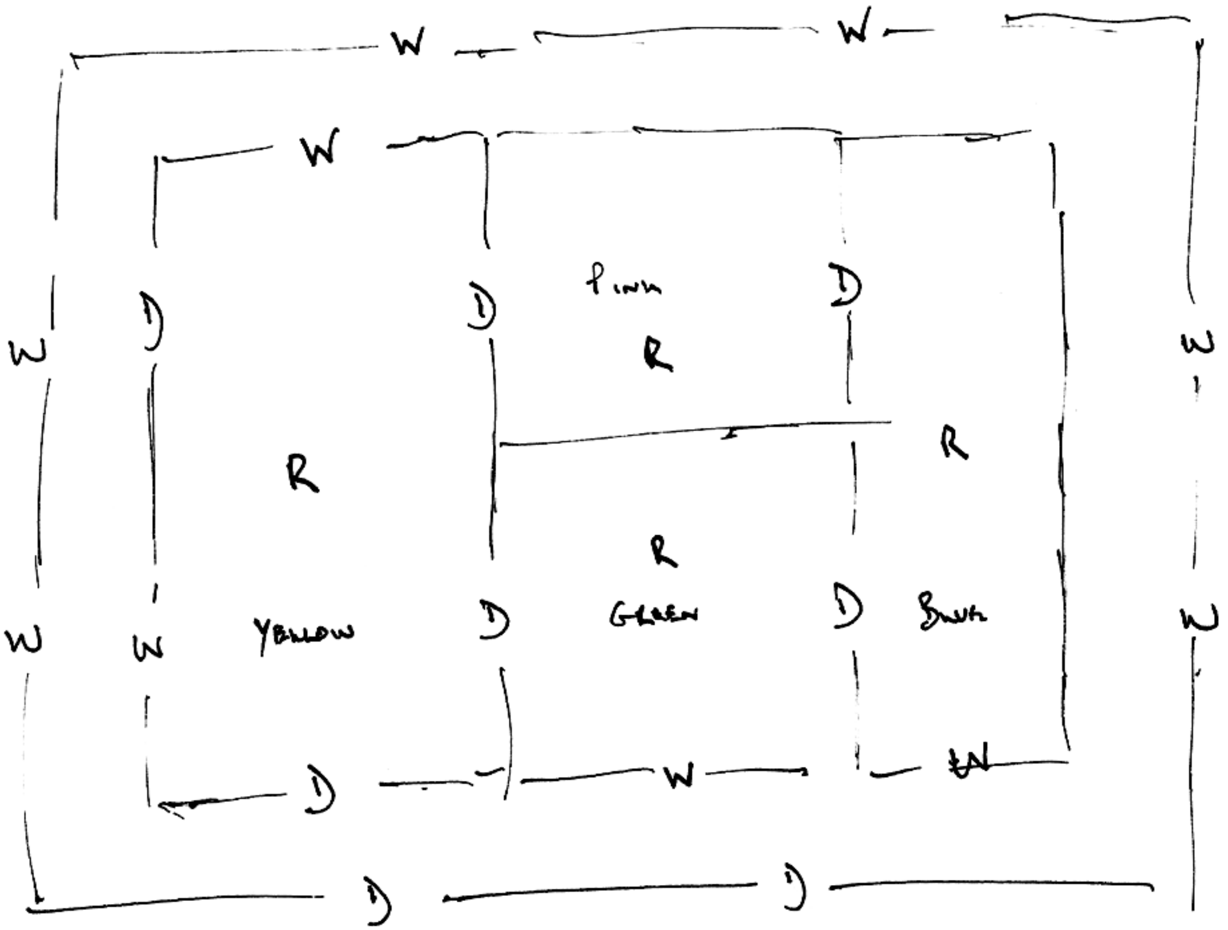

69 map 2 Mirror Written

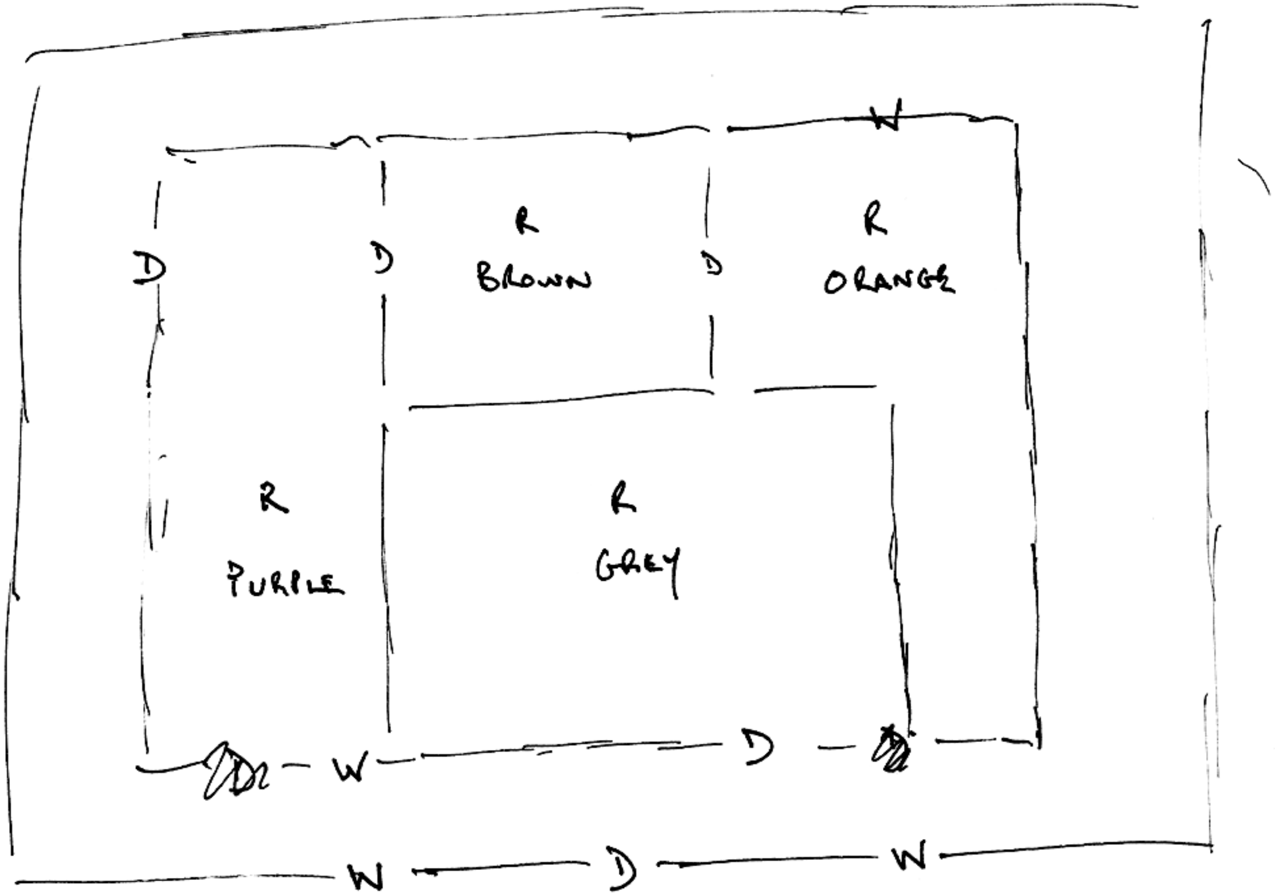

70 map 1 Mirror Written

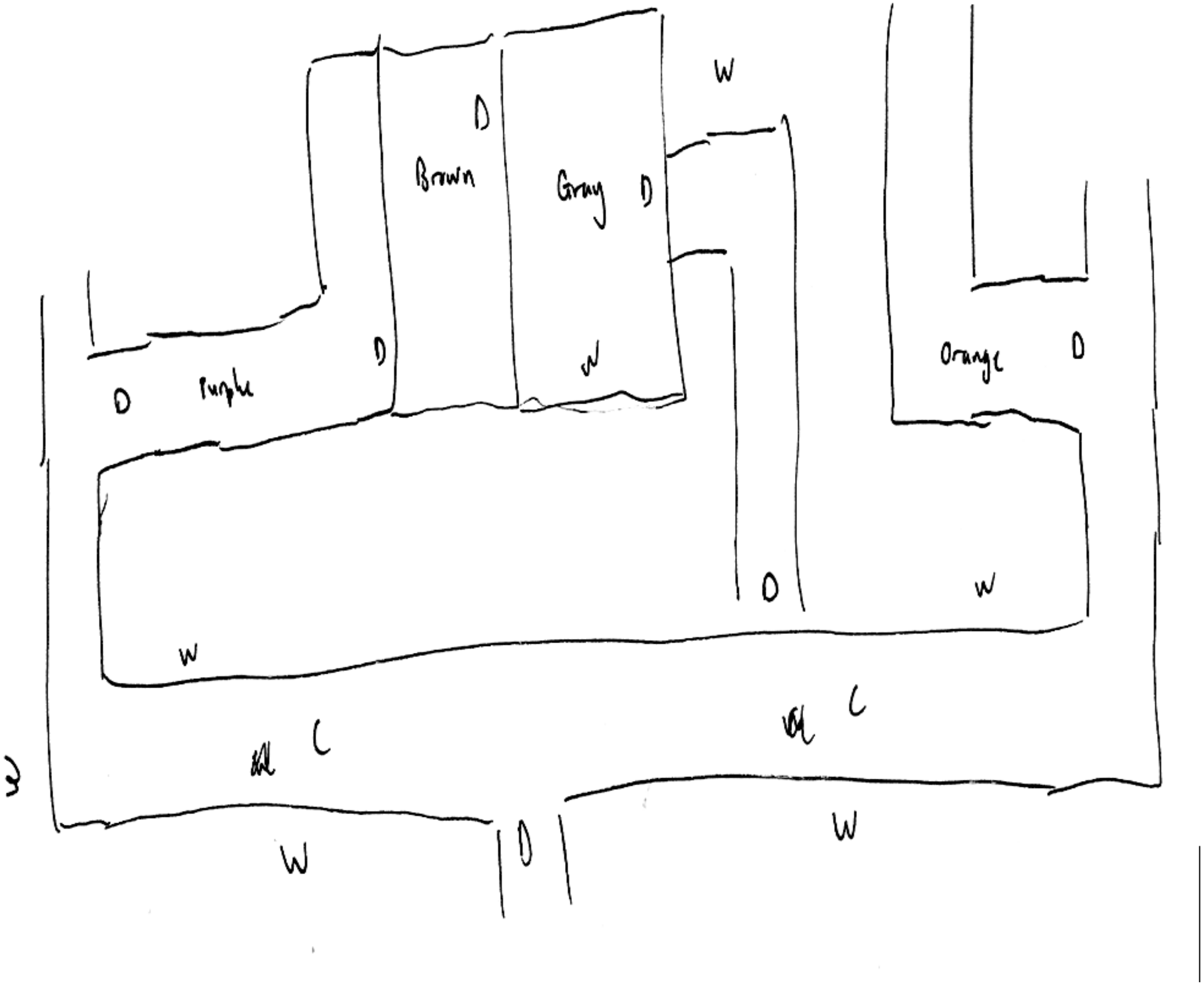

70 map 2 Rotational Video

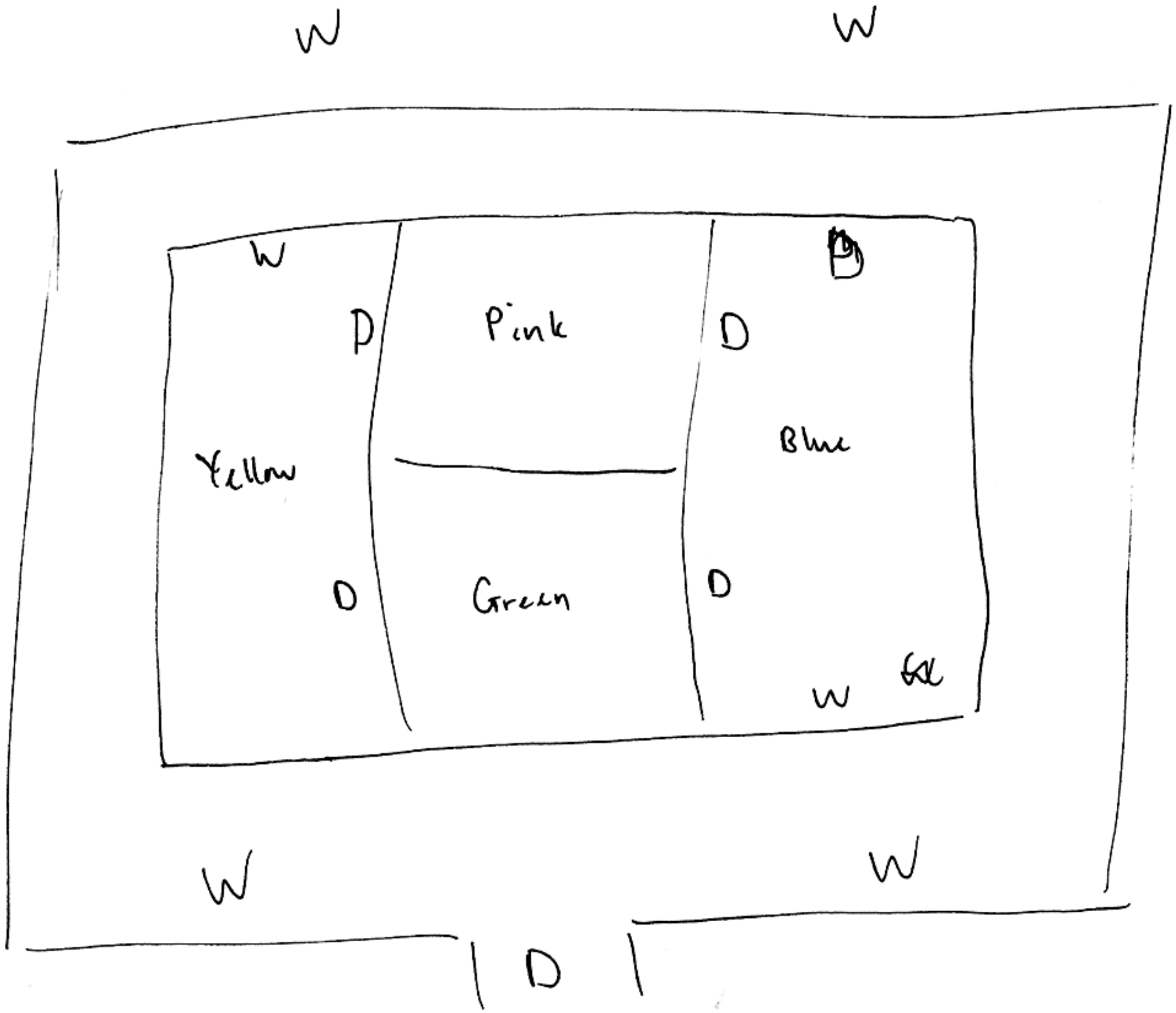

71 map 1 Rotational Written

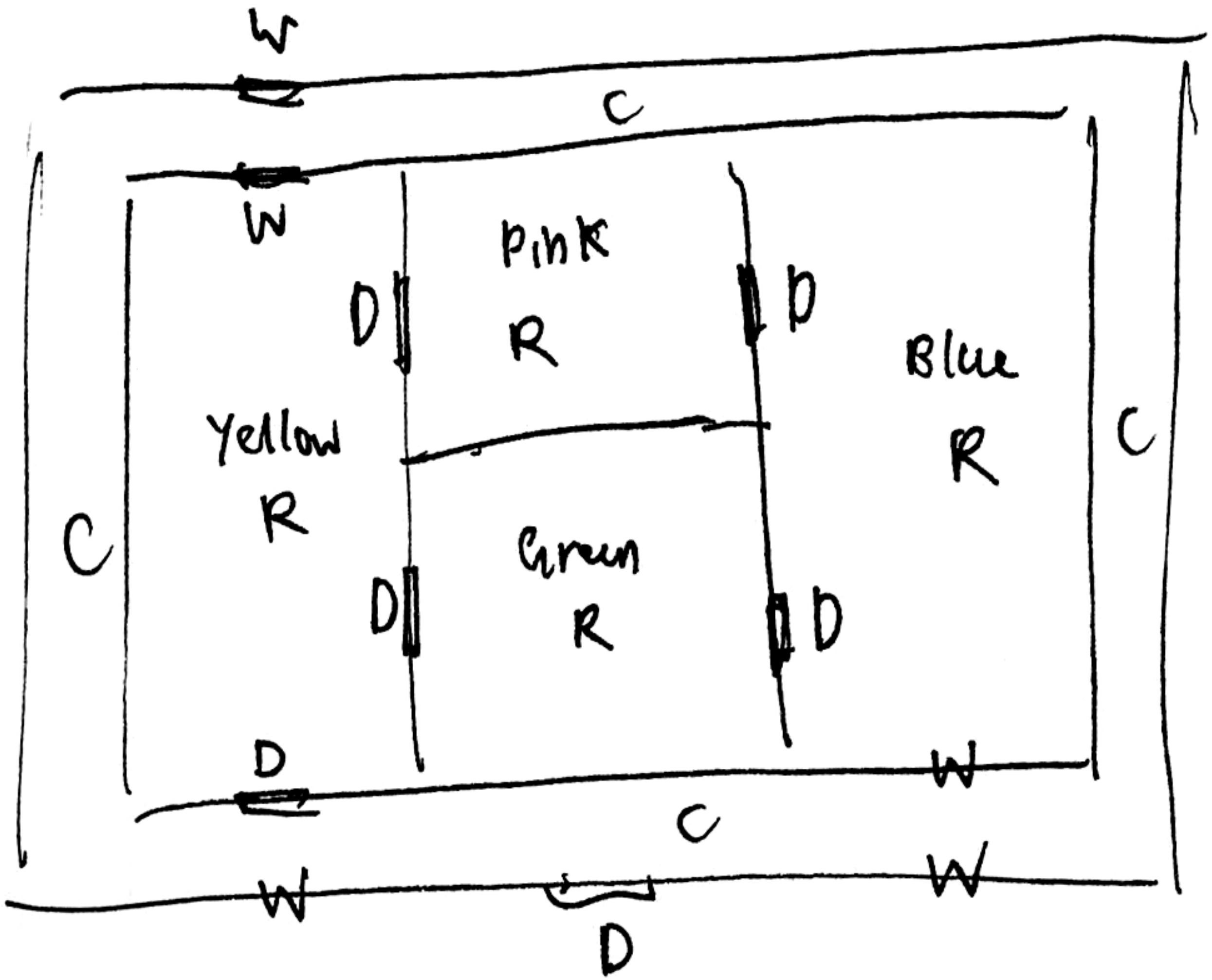

71 map 2 Mirror Video

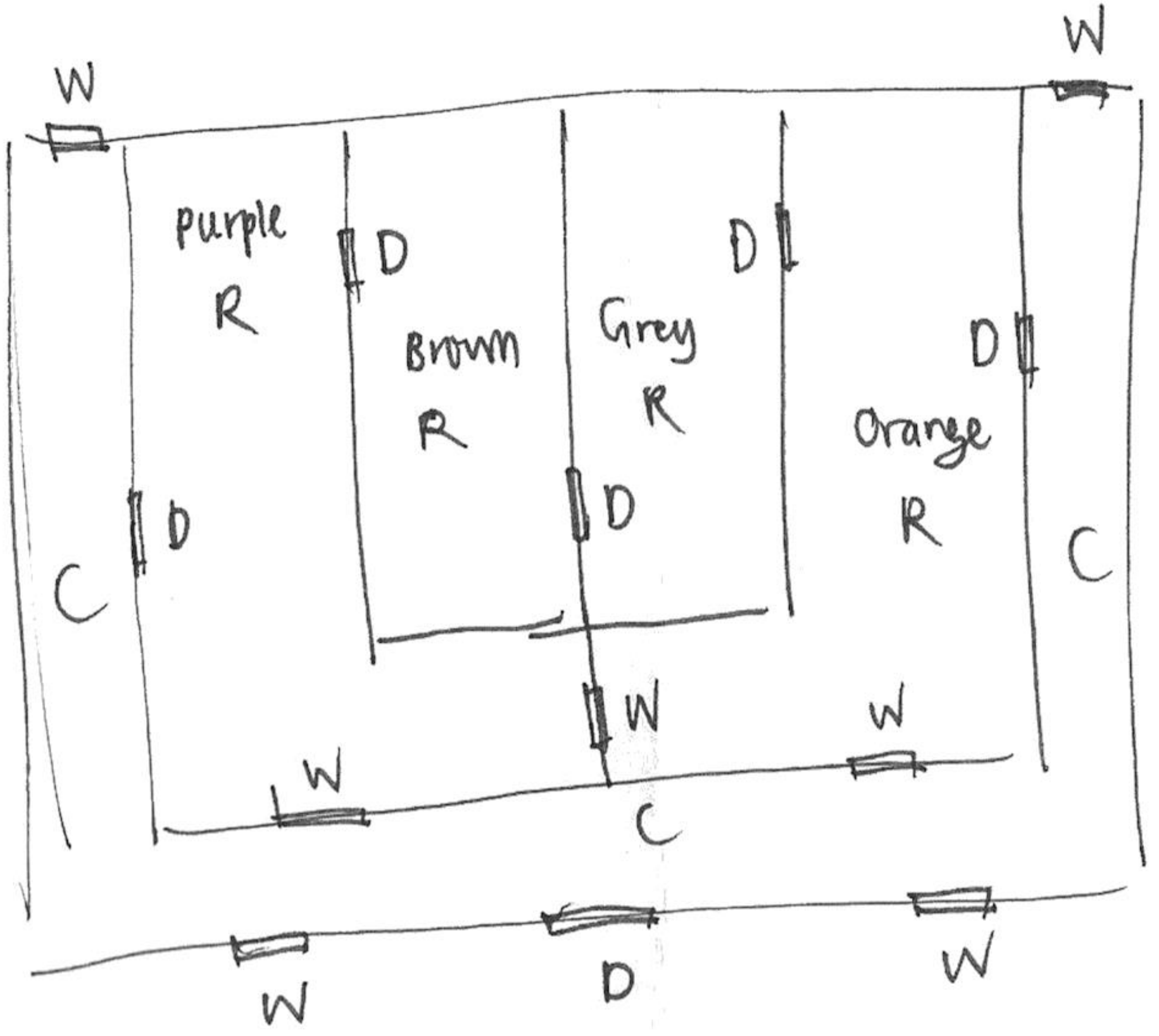

72 map 1 Mirror Video

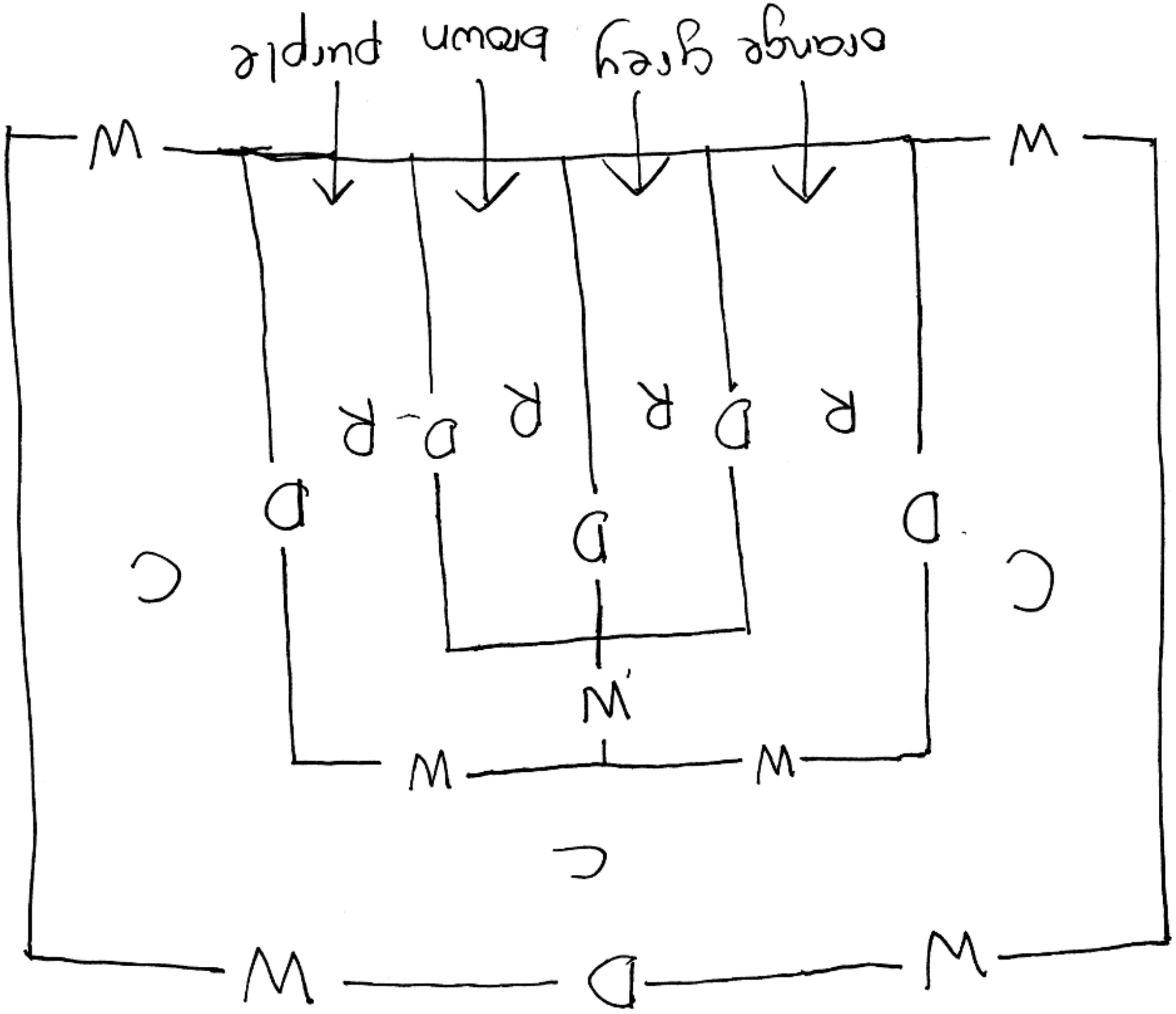

72 map 2 Rotational Written

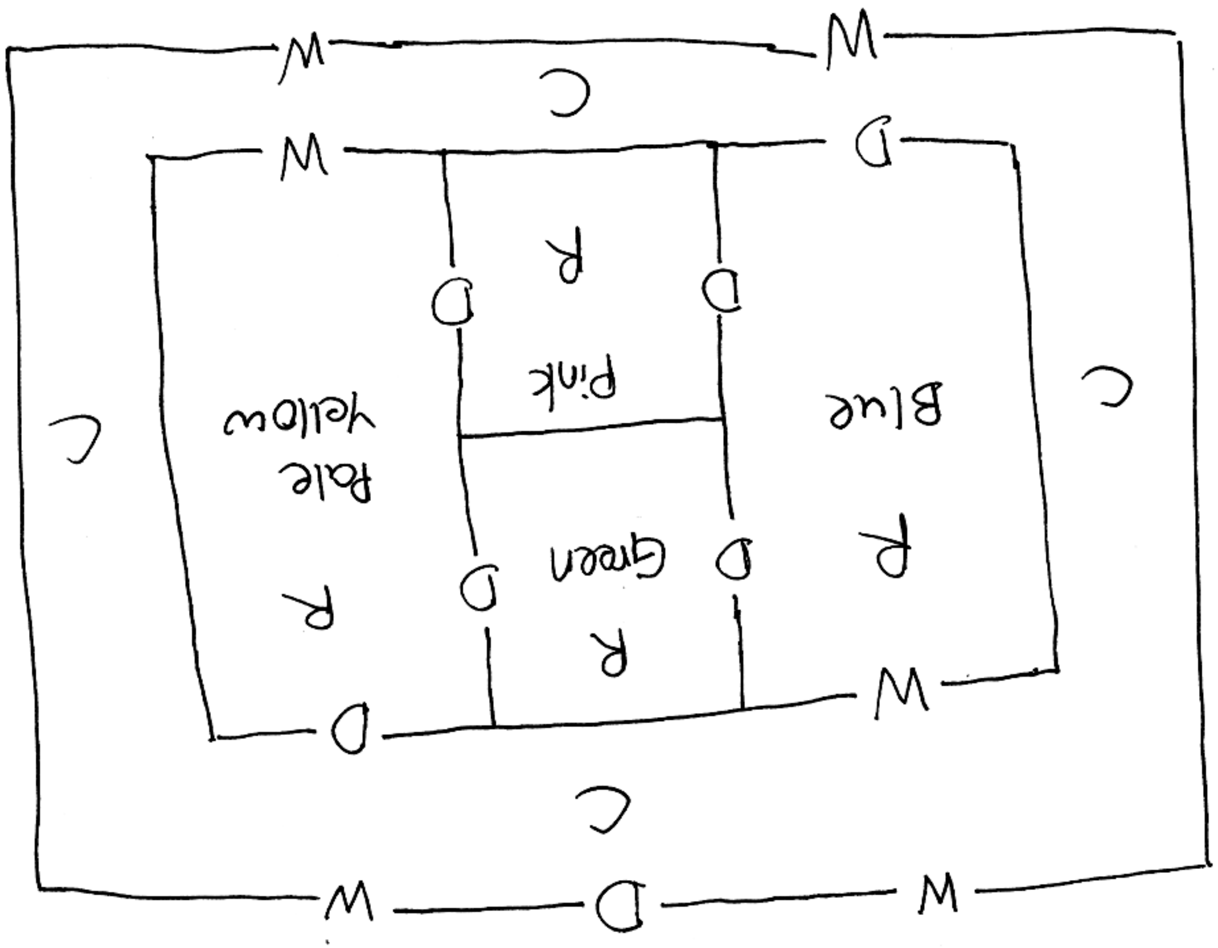

73 map 1 Rotational Video

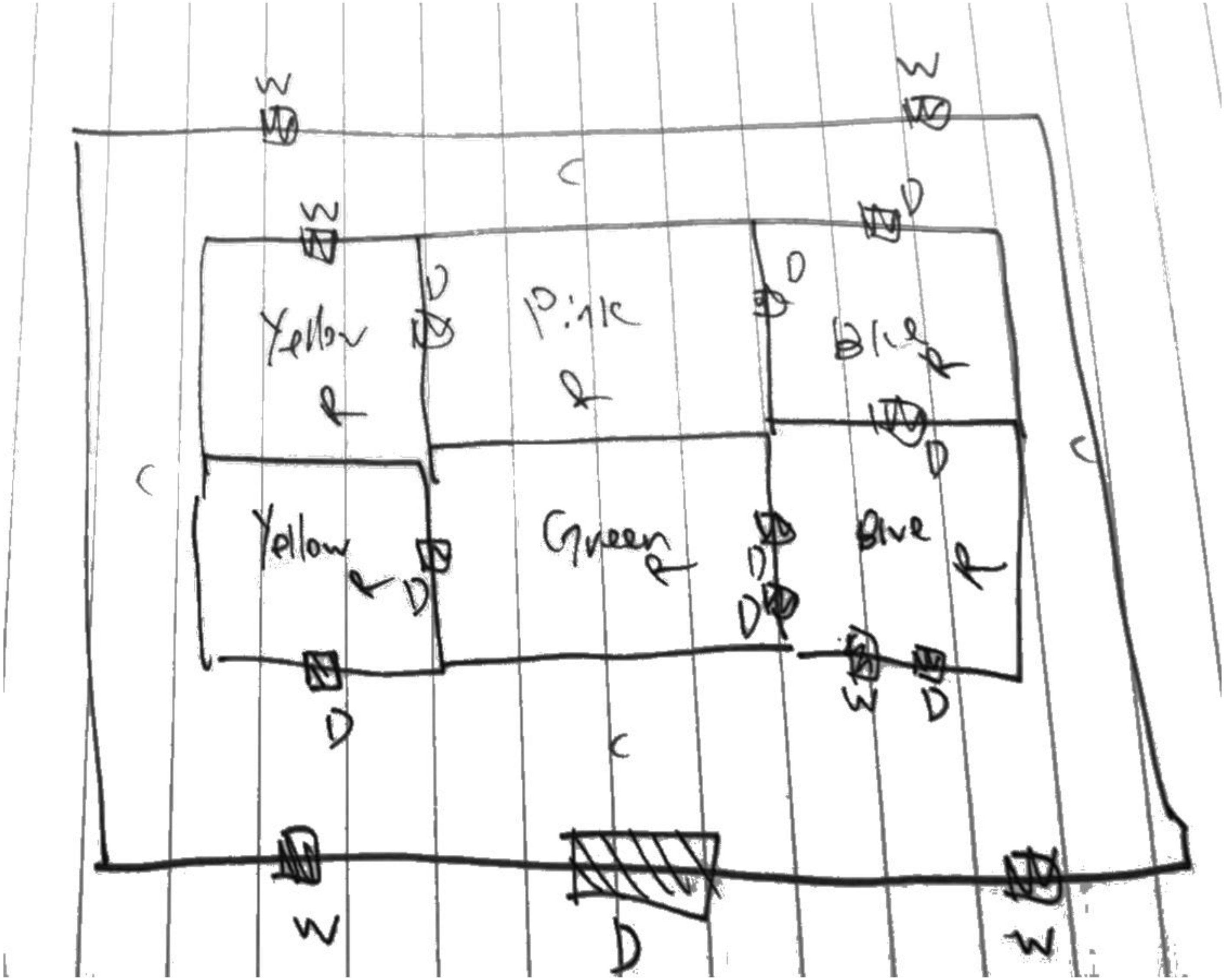

73 map 2 Mirror Written

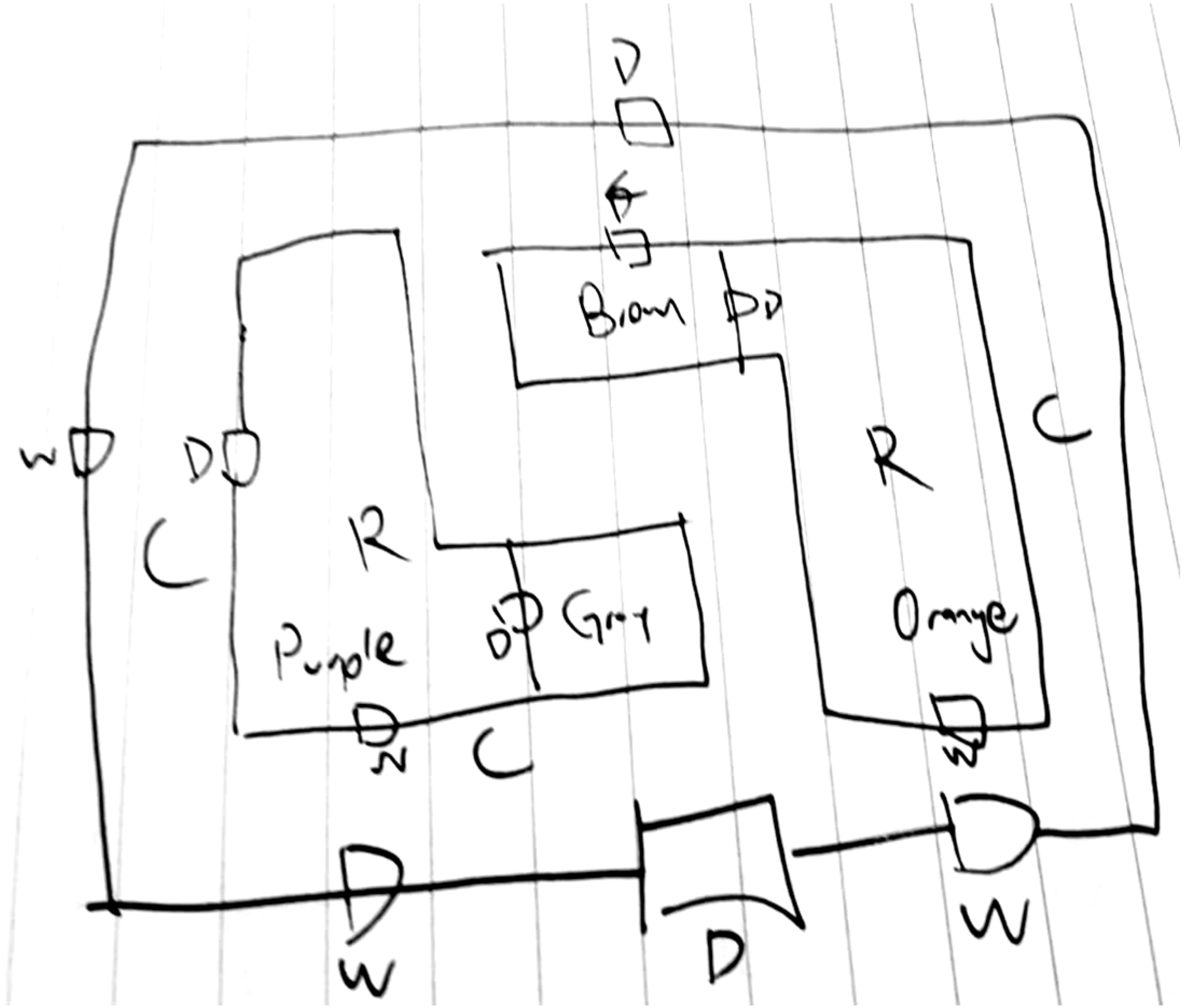

74 map 1 Mirror Written

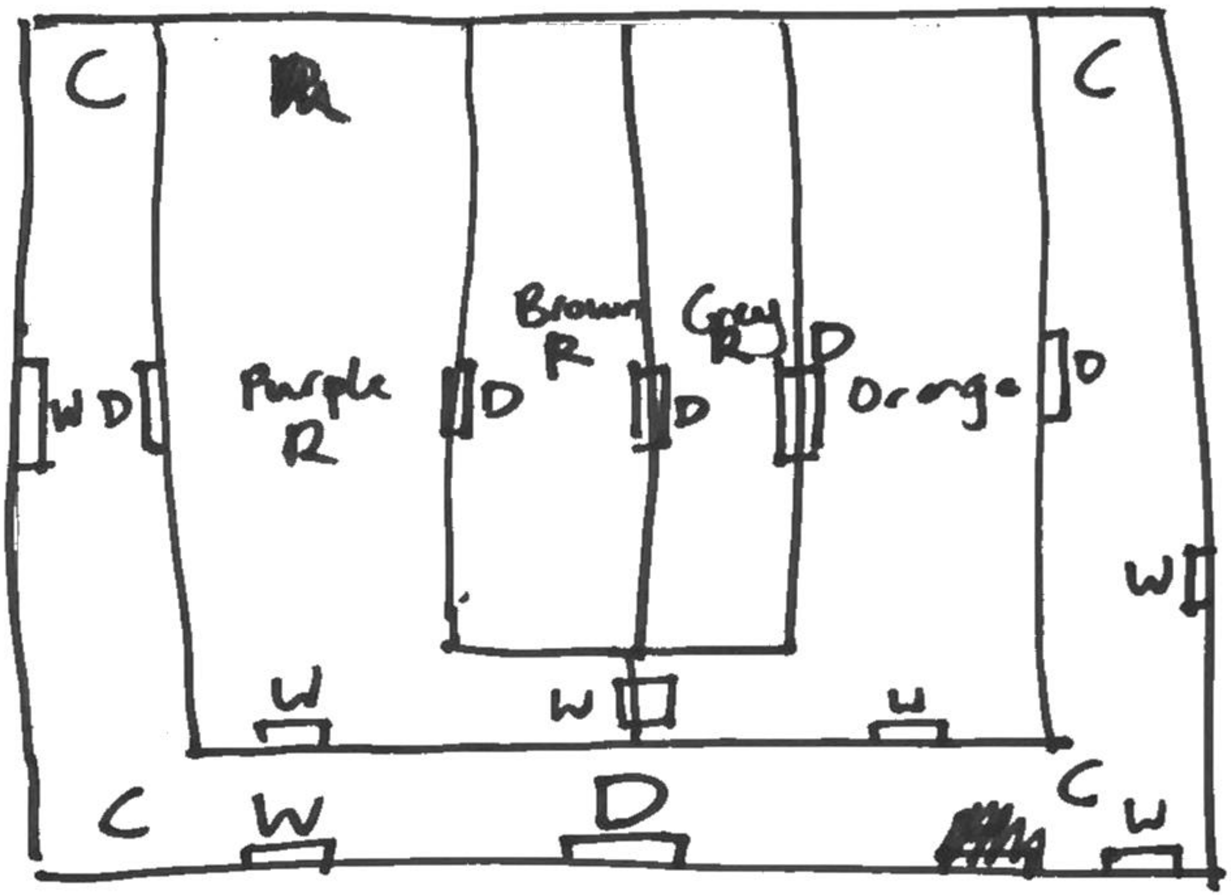

74 map 2 Rotational Video

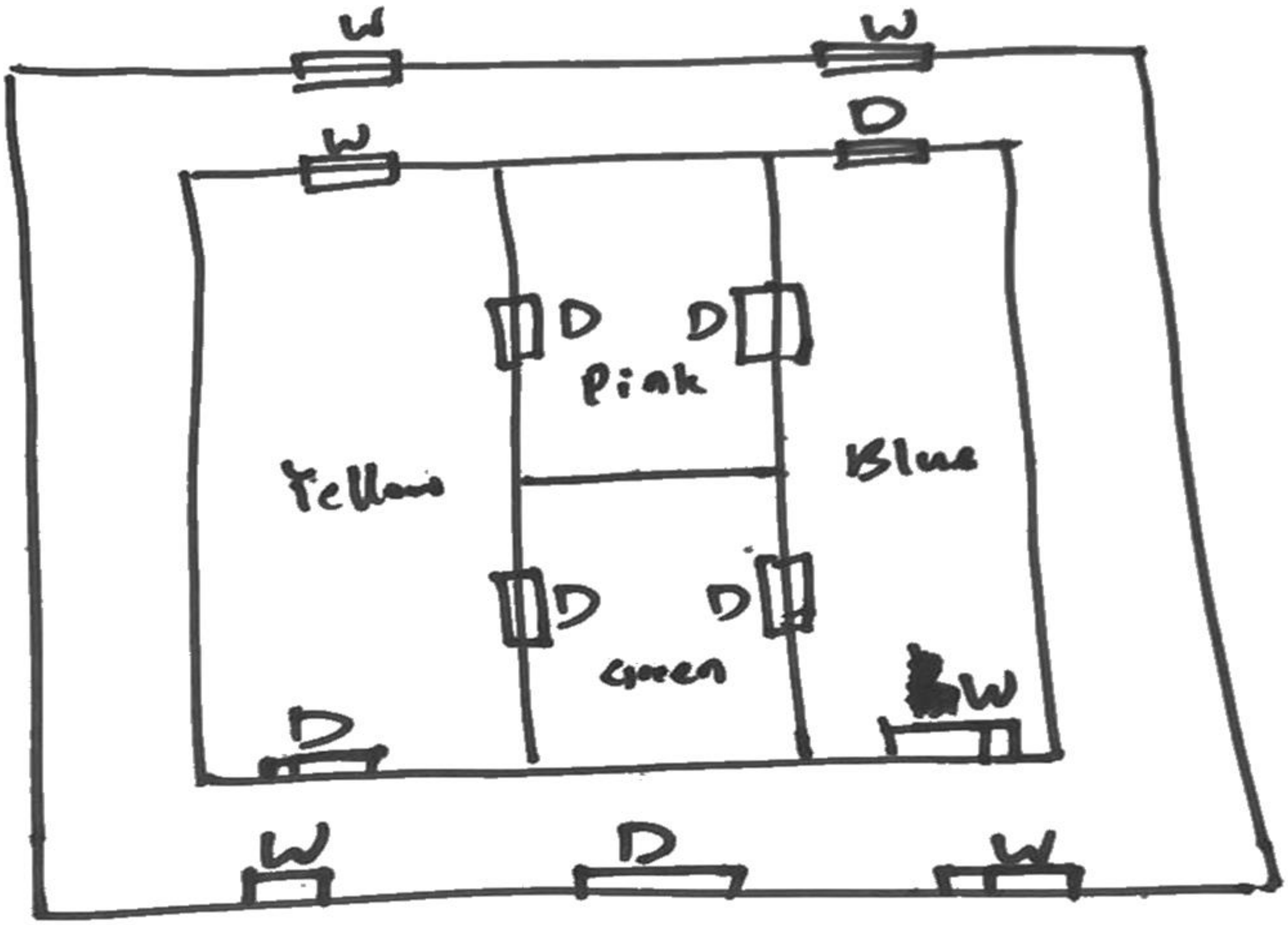

75 map 1 Rotational Written

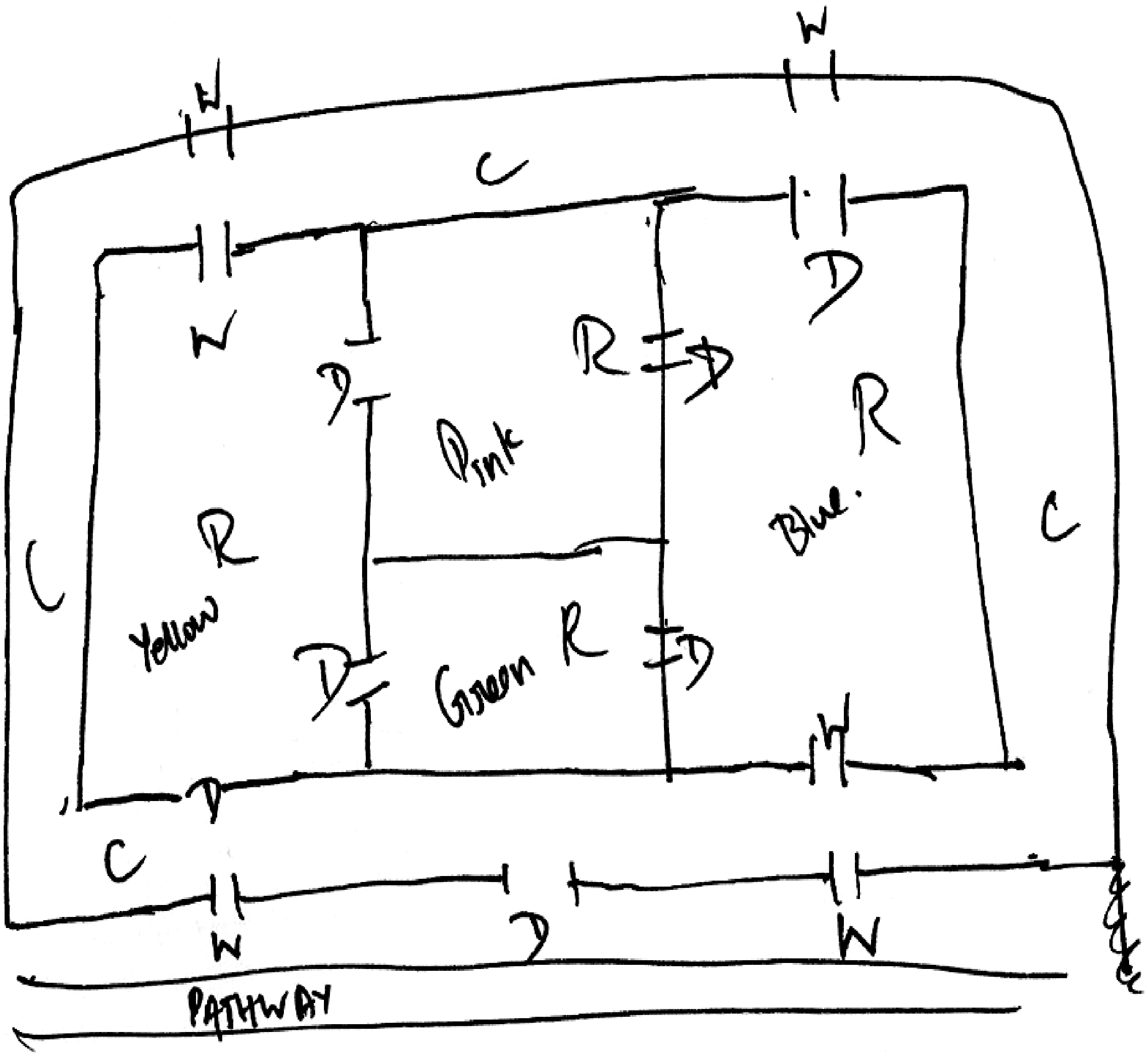

75 map 2 Mirror Video

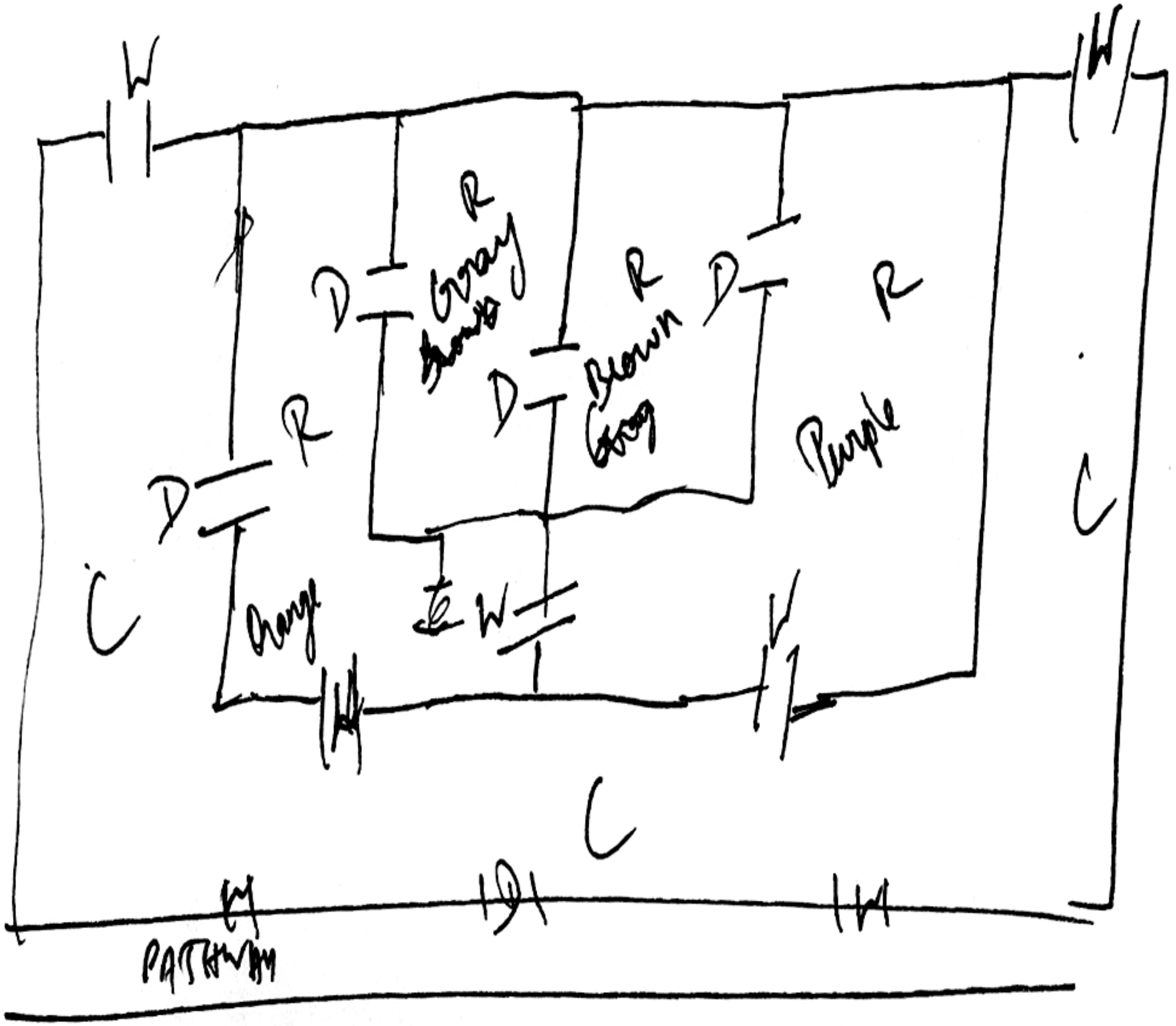

76 map 1 Mirror Video

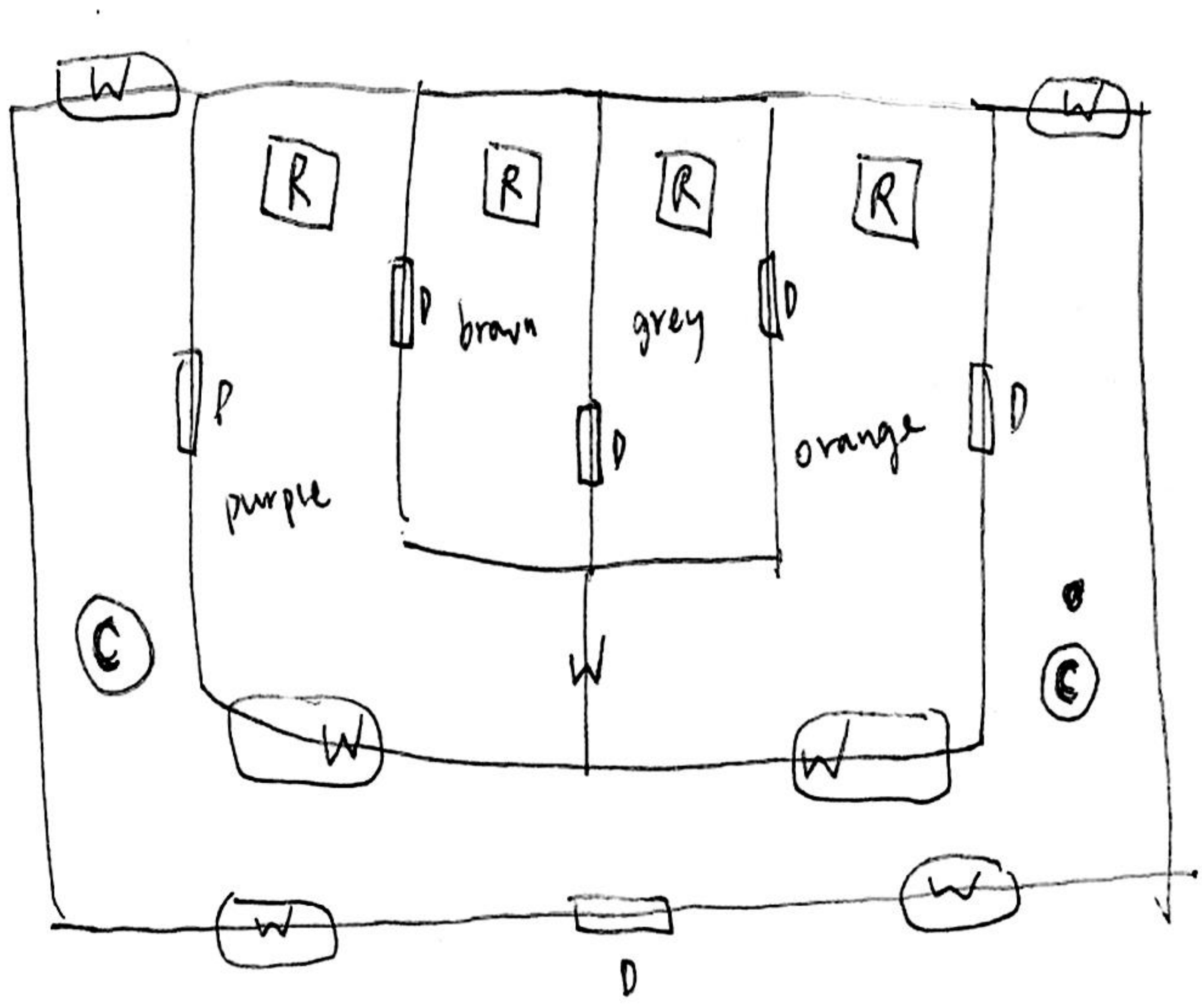

76 map 2 Rotational Written

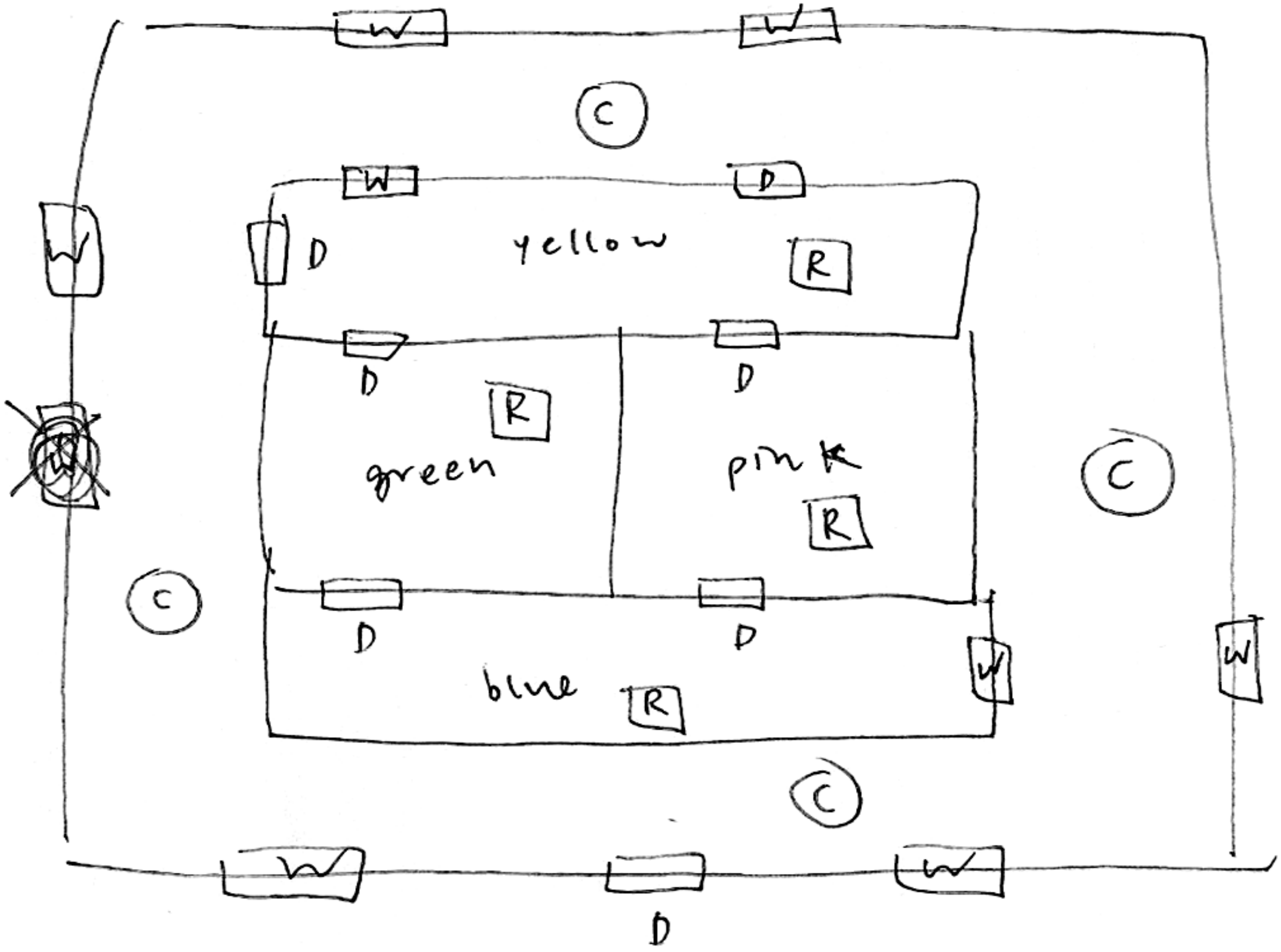

77 map 1 Mirror Video

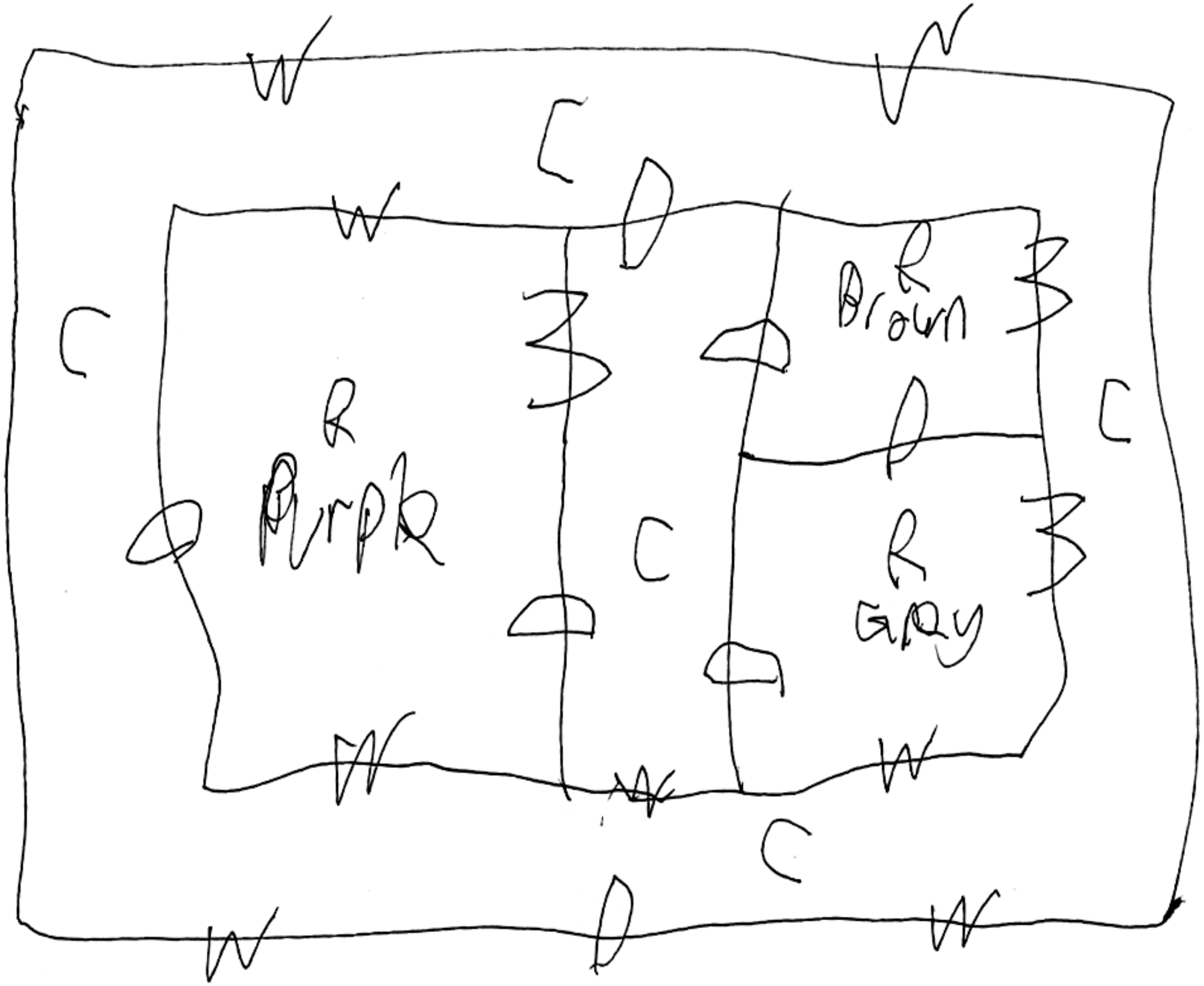

77 map 2 Rotational Written

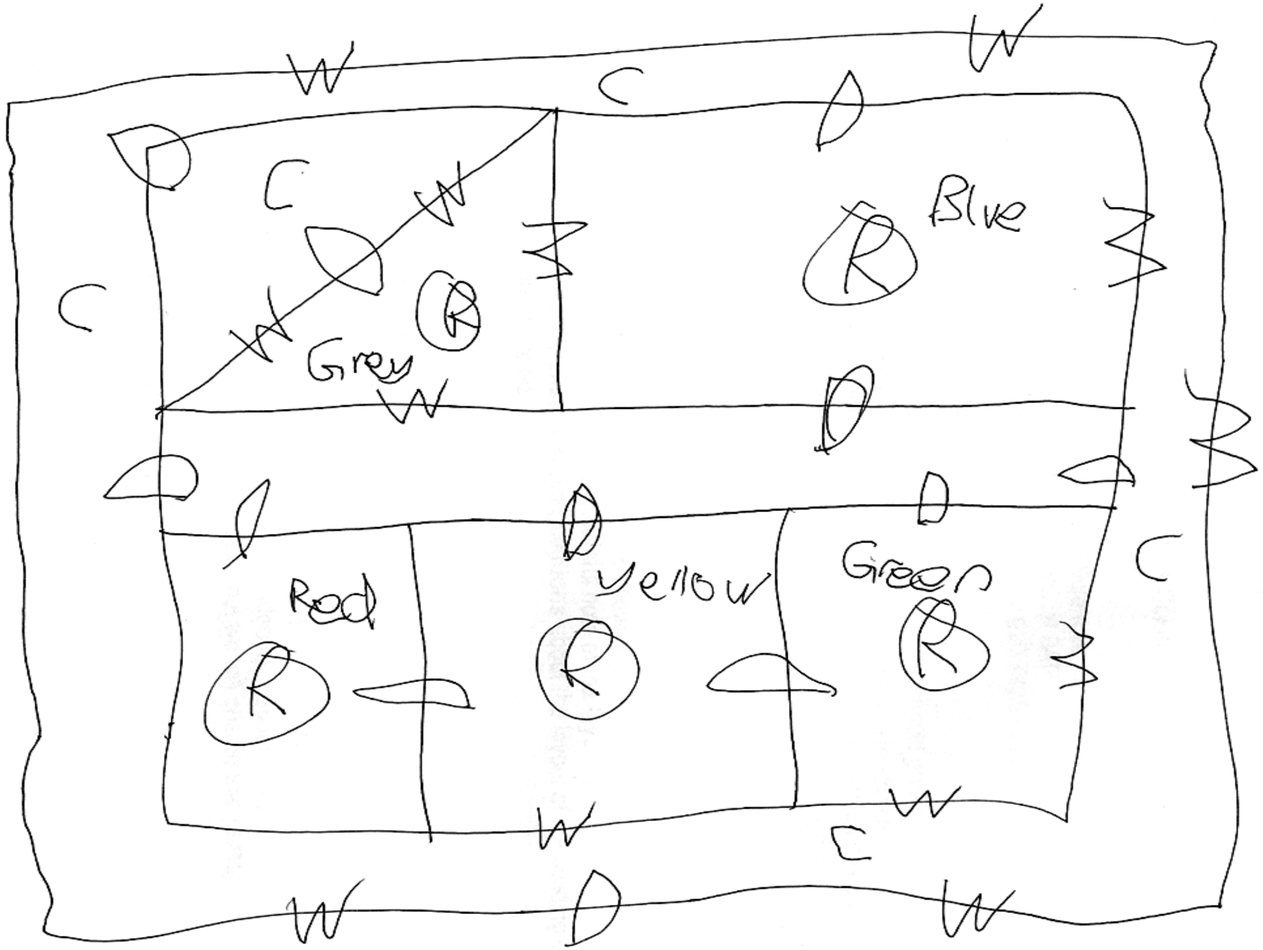

78 map 1 Mirror Written

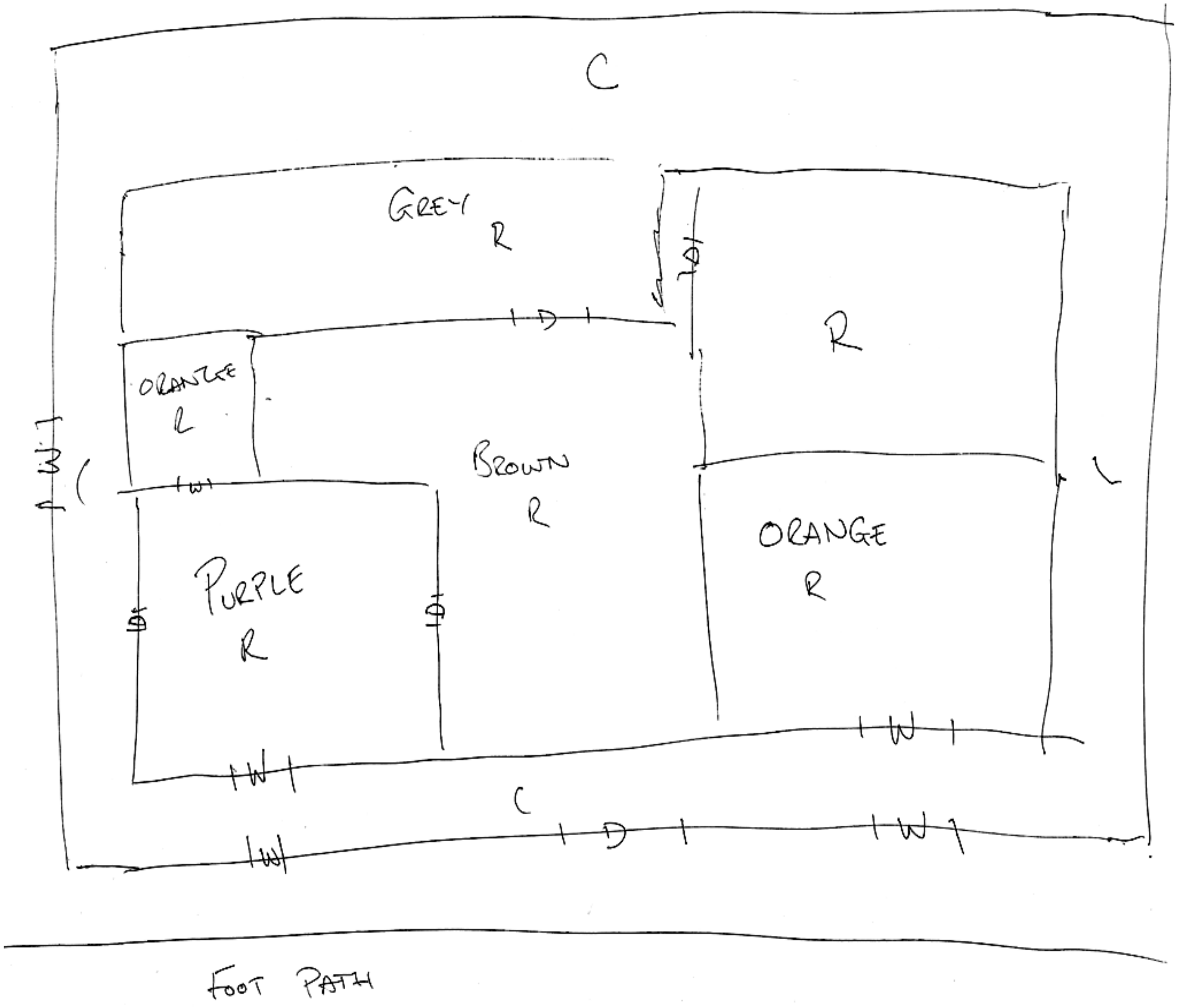

78 map 2 Rotational Video

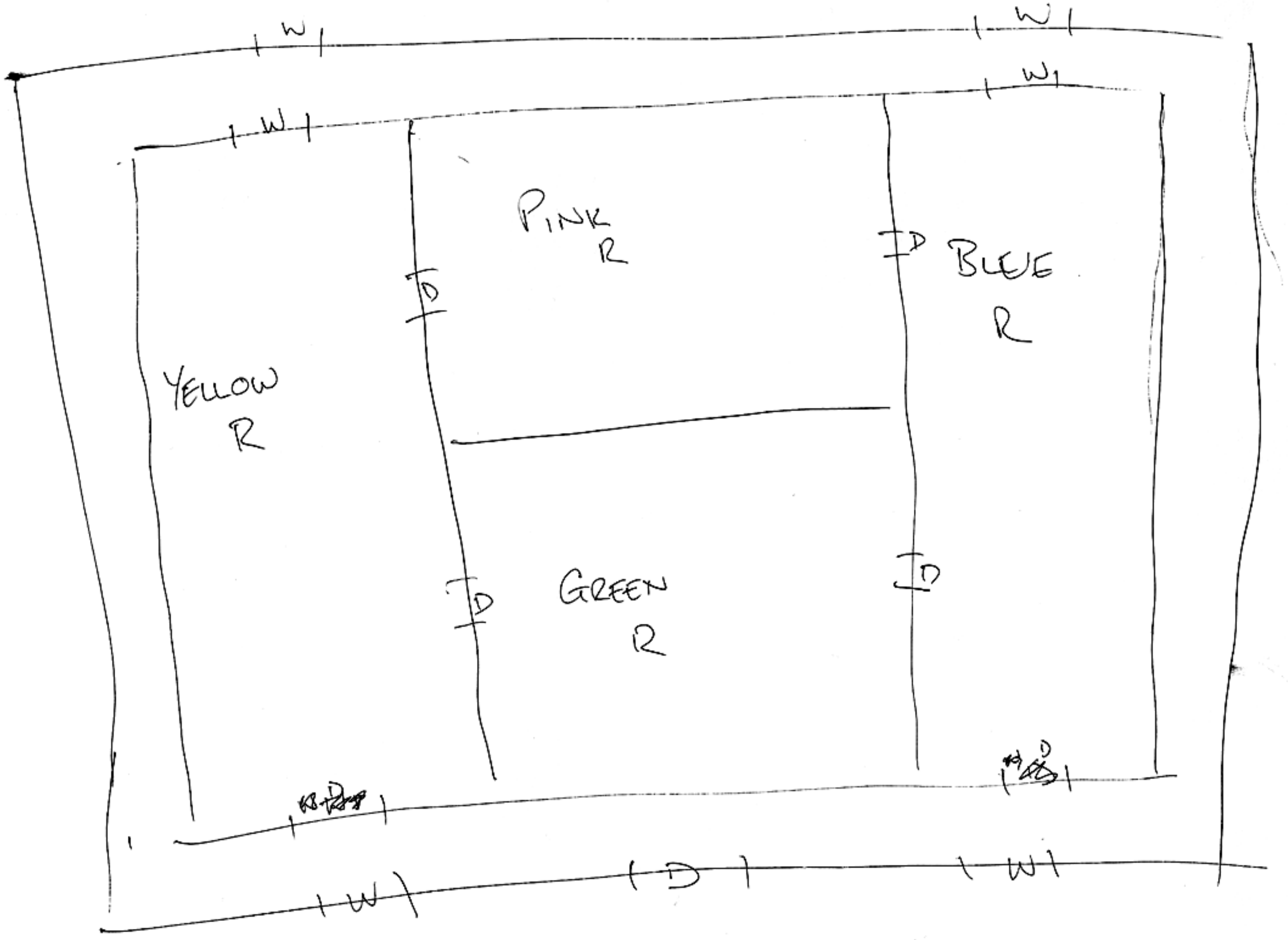

79 map 1 Rotational Video

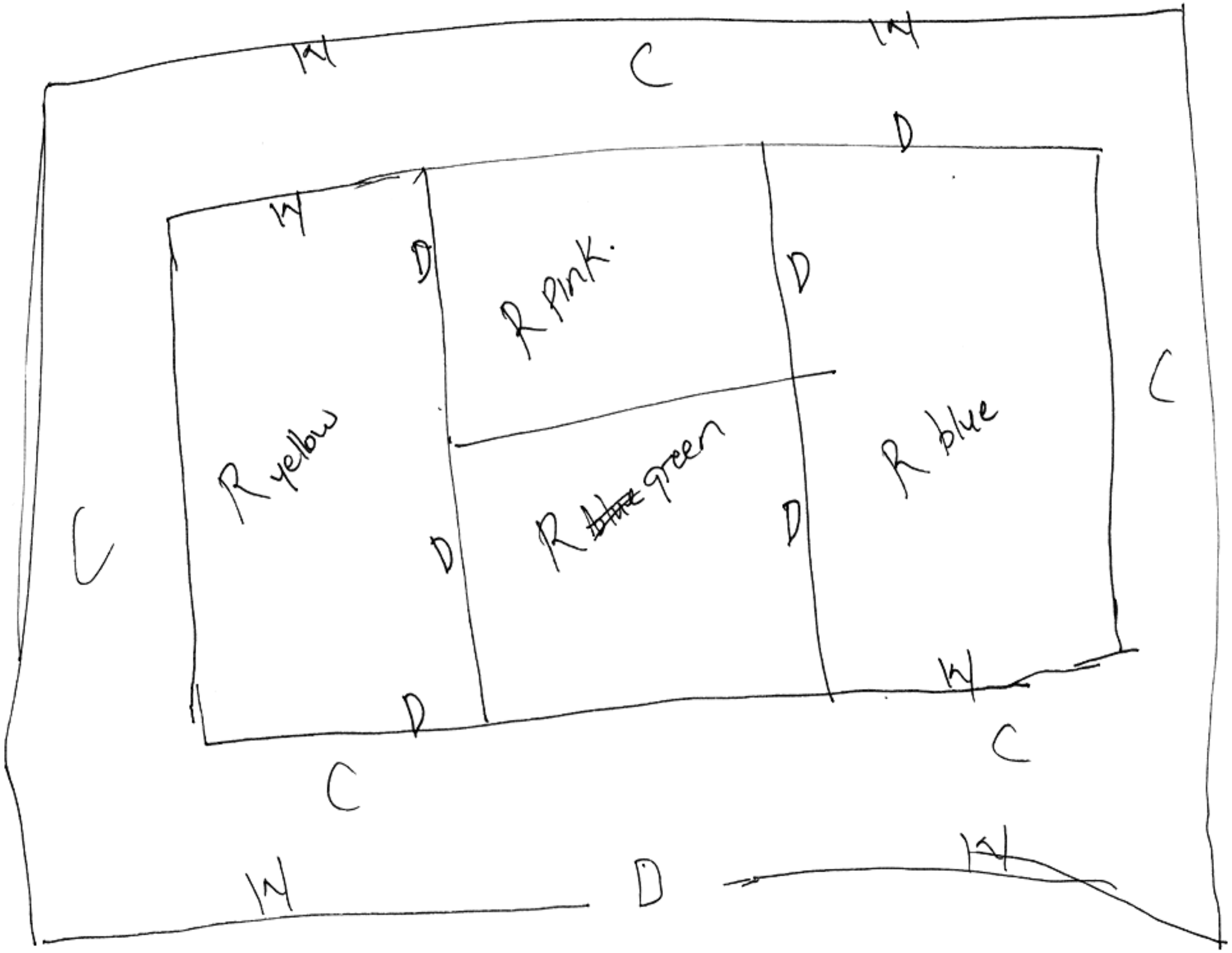

79 map 2 Mirror Written

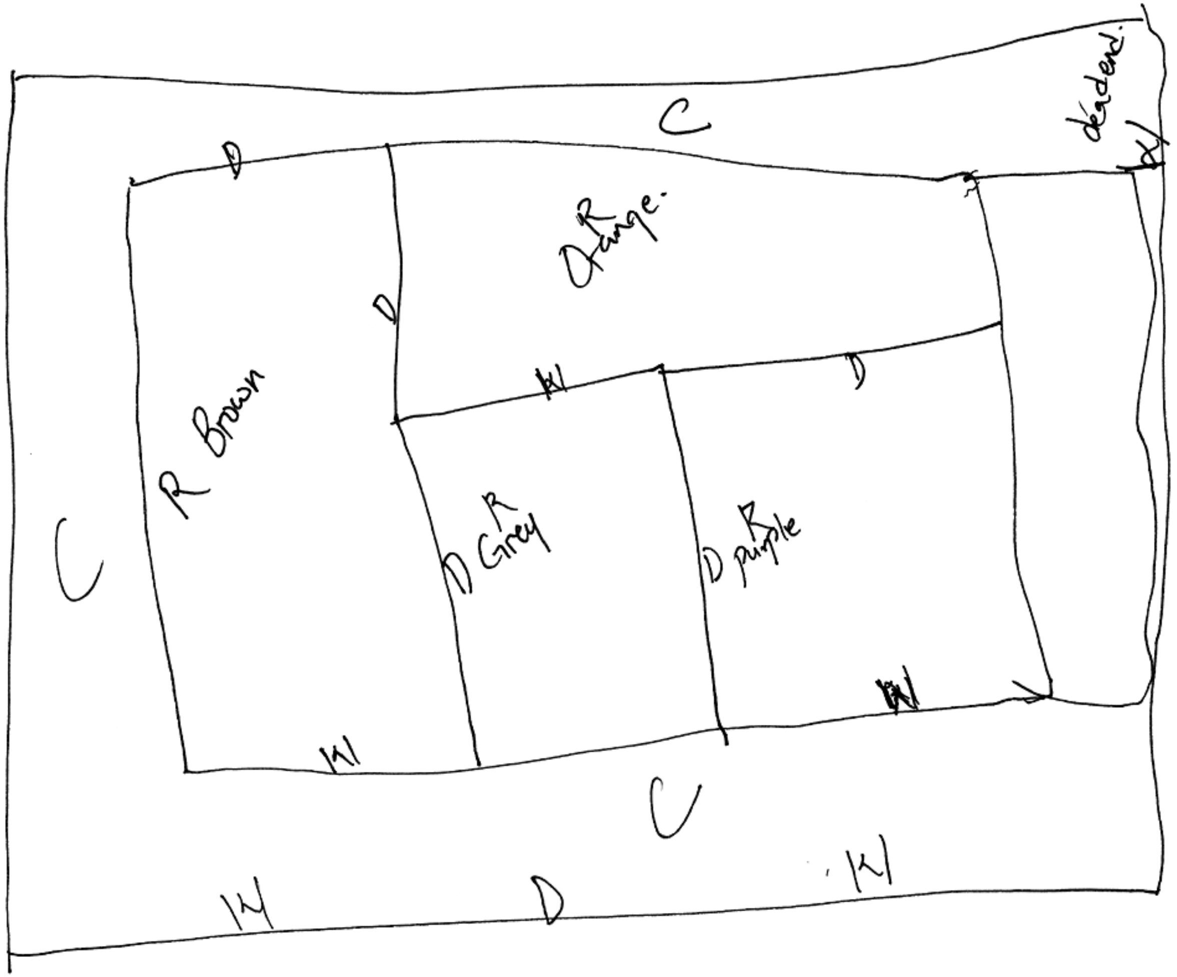

80 map 1 Rotational Written

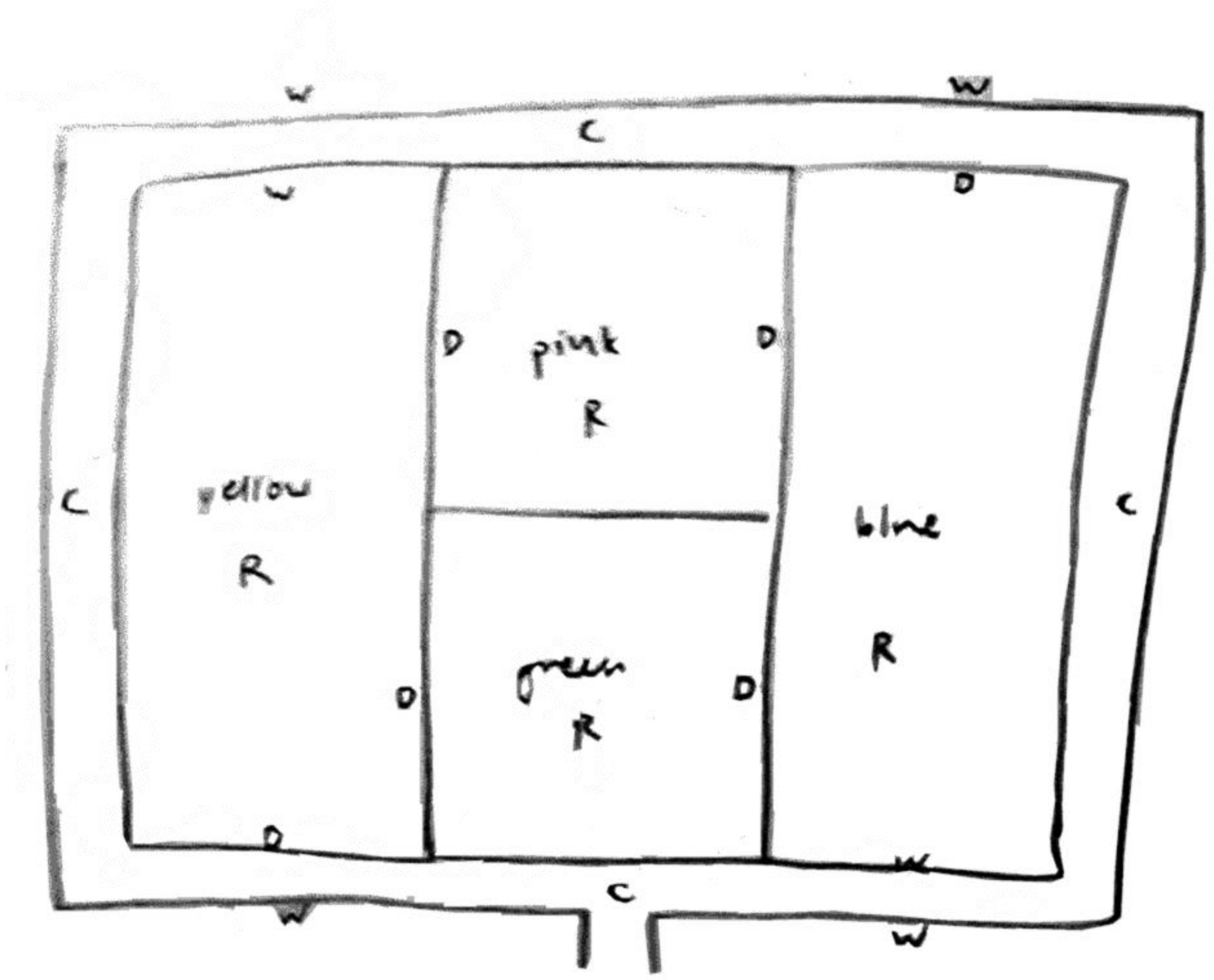

80 map 2 Mirror Video

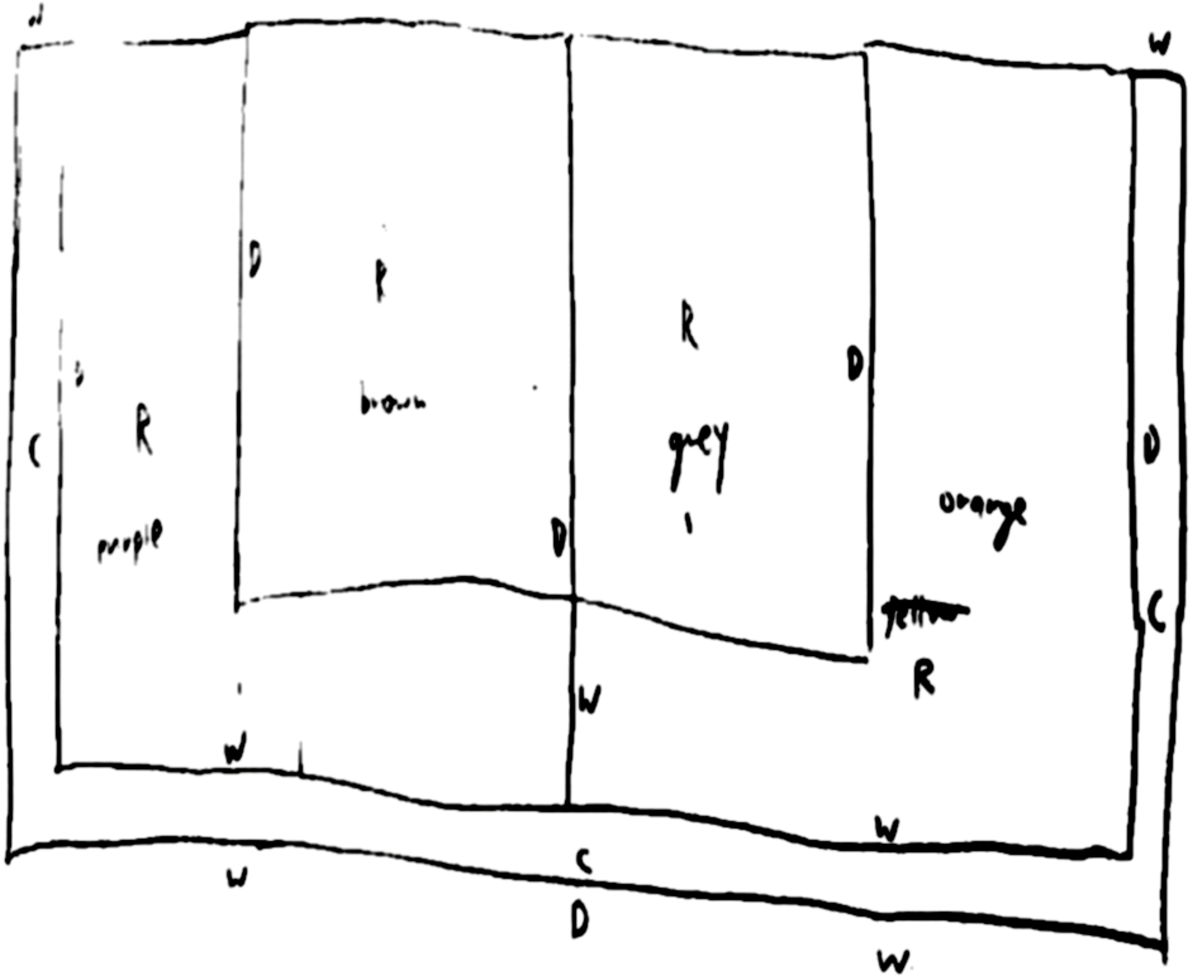

Supplement: 2 [file EMS140824-supplement-2.pdf]
