# Supplementary material for: Visual imagination and cognitive mapping of a virtual building
Source: J Navig. Author manuscript; Available in PMC 2022 Apr 12. (PMC7612610; doi:10.1017/S0373463321000588)
Supplement: 3 [file EMS140824-supplement-3.docx]

#### Questionnaire for the mirror symmetric building

Correct answers are highlighted

1. How many doors are there in the purple room?
   1. 1
   2. 2
   3. 3
   4. 4
2. Are there any windows in the gray room?
   1. Yes
   2. No
3. Which of the following is NOT one of the colours of the rooms in the house?
   1. Orange
   2. Gray
   3. Pink
   4. Purple
4. You leave the gray room and enter the orange room. The wall to your right, at the end of the room, has …
   1. A door
   2. A window
   3. 2 doors
   4. no door or window
5. You just entered the brown room from the door. On the long wall directly in front of you, there is …
   1. A door
   2. A window
   3. 2 windows
   4. no door or window
6. How many windows are there in the orange room?
   1. 1
   2. 2
   3. 3
   4. 4
7. You just entered the purple room. The short wall to your right, at the end of the room, has …
   1. A window
   2. A door
   3. 2 windows
   4. 2 doors
8. How many doors are there in the gray room?
   1. 1
   2. 2
   3. 3
   4. 4
9. . You are looking down into the right hand corner in the orange room. At the dead end of the corner, there is a window, which looks into ...
   1. The gray room
   2. The brown room
   3. The purple room
   4. The corridor
10. How many external windows are there in the house?
    1. 1
    2. 2
    3. 3
    4. 4
