# Supplementary material for: Visual imagination and cognitive mapping of a virtual building
Source: J Navig. Author manuscript; Available in PMC 2022 Apr 12. (PMC7612610; doi:10.1017/S0373463321000588)
Supplement: 4 [file EMS140824-supplement-4.docx]

#### Script for mirror symmetric building

You are walking down a footpath. On your left is a brick building with an open door. When you reach the door you turn to face it. Through the open door you can see a corridor running from left to right.

You look to your left along the façade of the building and see that about halfway long, there is a window. You look to your right and see that there is another, identical window, similarly located about halfway along.

You turn back towards the door and step through it into the building. You find yourself in the centre of a long corridor extending to your left and right. You turn and face down the left-hand arm of the corridor.

This corridor turns around a right-hand corner at its far end. Halfway along the left-hand wall is the window looking out onto the street. Opposite it, in the right-hand wall, is another window, through which you glimpse a purple room.

You turn to look down the right-hand arm of the corridor and see that the corridor makes a left-hand turn at its far end. Halfway along the right-hand wall of this corridor is one of the windows looking out on the street. Opposite it, on the left-hand wall, is an interior window. You walk along the corridor until you reach the windows, then turn to your left and look through the interior window, noting that it looks into an orange room. You continue turning until you are now facing back up the corridor.

You walk along the corridor, past the main entrance, until you reach the windows. You turn to your right to look through the interior window into the purple room, before resuming your walk along the corridor to the end, where it turns the corner to the right.

Ahead of you, the corridor continues straight ahead and ends at a wall, which has a window looking to the outside. Halfway along the right-hand wall there is an open door.

You walk along the corridor to the door, turn to face it and then step through into the room beyond.

 You find yourself in a purple room. In the wall in front of you is another open door, opening into an inner room coloured brown. The wall to your left is blank, with neither doors nor windows. To your right, you see that the room continues around a left-hand corner, making an L-shape. The wall at the far end has an interior window, through which you can see the corridor.

You turn right and walk towards the window until you nearly reach it, and then turn left to face down the second, shorter arm of the L-shaped room.

In front of you is a dead end with an interior window, which looks into an orange-painted room that lies beyond.

You turn left to face back up the room, and then walk forwards until you reach the door in the wall to your right that leads into the brown room. You turn, and go through this door into the room.

This room is small and rectangular. To your left and right, at the ends of the room, are two short walls which are blank, with neither doors nor windows. The long wall that faces you

has a door towards its right-hand end, near the end wall.

You turn and walk towards the door, and then go through.

You find yourself at one end of a rectangular grey room, identical in size and shape to the brown room you just came from. To your left and right, at the ends of the room, are two short walls, which are blank, with neither doors nor windows. The long wall that faces you has another open door near its centre.

You turn and walk towards that door, and then go through.

You find yourself in an orange room. In the long wall in front of you is another door, opening out onto a corridor. The short wall to your left is blank, with neither doors nor windows. To your right, you see that the room continues around a right-hand corner, making an L-shape. The wall at the far end has an interior window, through which you can see the corridor.

You turn right and walk towards the window until you nearly reach it, and then turn right again to face down the second shorter arm of the L-shaped room.

In front of you is a dead end with an interior window, which looks into the purple room.

You turn right once more to face back up the room, and then walk towards the door in the wall to your right. You turn, and go through this door into the corridor beyond.

You find yourself at the centre of the corridor, which stretches to your left and right. To your left, the corridor ends at a wall, which has a window looking to the outside. You look down the right-hand arm of the corridor and see that the corridor makes a right-hand turn at its far end.

You turn right and walk down the corridor to the far end and turn right again.

You find yourself back in the corridor you first encountered. At the centre of the long wall to your left is the main door from which you entered the house, with the two windows on either side. In the long wall to your right are the two interior windows.

You walk along the corridor until you reach the main door. You step through this door and back out of the house onto the street.
