# Supplementary material for: Visual imagination and cognitive mapping of a virtual building
Source: J Navig. Author manuscript; Available in PMC 2022 Apr 12. (PMC7612610; doi:10.1017/S0373463321000588)
Supplement: 5 [file EMS140824-supplement-5.docx]

#### Script for rotationally symmetric building

You are walking down a footpath. On your left is a pale building with an open door. When you reach the door you turn to face it. Through the doorway you can see a corridor running from left to right.

You look to your left along the façade of the building and see that about halfway along, there is a window. You look to your right and see that there is another, identical window, similarly located about halfway along.

You turn back towards the door and step through it into the building. You find yourself in the centre of a long corridor extending to your left and right. You turn and face down the left-hand arm of the corridor.

The corridor turns around a right-hand corner at its far end. Halfway along the left-hand wall is the window looking out onto the street. Opposite it, in the right-hand wall, is an open door.

You turn around to face the other way and look down the right-hand arm of the corridor, and see that the corridor makes a left-hand turn at its far end. Halfway along the right-hand wall of this corridor is one of the windows looking out on the street. Opposite this window, on the left-hand wall, is an interior window. You walk along the corridor until you reach the windows, then turn to your left and look through the interior window, noting that it looks into a blue room. You continue turning until you are now facing back up the corridor.

You walk along the corridor, past the main entrance until you reach the other window and the open door. Your turn right, towards the doorway, and step into the room beyond.

You find yourself at one end of a long, rectangular, pale yellow room, which stretches out in front of you. At the centre of the wall at far end of the room is a window. In the wall to your right are two open doors, located, respectively, one-quarter and three-quarters of the way along the wall. The long wall to your left is empty, with no doors or windows.

You walk over to the nearest door, turn to face it and then walk through the door into the room beyond. You find yourself in a smaller square room, painted green. Immediately in front of you is another open door, through which you can see a blue room beyond. Your turn your head to look at the left wall, which is blank, with no doors or windows. You look to the right and see that this wall is also blank. You turn back to face the door and step through it to the blue room.

This room is rectangular, identical in size and shape to the yellow one you were in earlier. The wall in front of you is blank. You turn to your right and see, in the short wall, a window looking out into the corridor. Through the window you can see that in the far wall of the corridor there is a window looking out onto the street. You turn to your left to face down the room. In the wall at the far end there is a closed wooden door. You turn further and see that the long wall to your left has two doors: the one you just came through from the green square room, which is one quarter of the way along the wall, and another, located three quarters of the way along. This door is open but the door opens into the room you are in, obscuring the view into the room beyond.

You walk down the room towards this second door, turn towards the doorway and go through into the room beyond.

You find yourself in a smaller square room, painted pink. Immediately in front of you is another open door, looking through into the yellow room beyond. Your turn to look at the left wall, which is blank, with no doors or windows. You look to the right and see that this wall is also blank. You turn back to face the door and step through it into the yellow room.

The wall in front of you is blank. You turn to your right and see a window looking out into the corridor. Through the window, in the far wall of the corridor you see an external window looking out onto open land. You turn to your left to face down the room. In the far wall there is an open door, through which you can see the corridor.

You walk down the room to this door, noting that the wall to your left has the open door leading to the green room that you went through earlier. When you reach the door in the end wall you step though into the corridor. In front of you, in the wall opposite, is a window looking out onto the street. You go over to this window and look out. Then you turn to the right, to face down the corridor to the end.  You see that the corridor continues around a corner to the right.

You walk along the corridor and turn the corner.

You find yourself in a blank stretch of corridor that has neither doors nor windows. At the far end, the corridor continues around another right-hand corner. You walk along to the end and turn the corner, to discover that the corridor again continues all the way along to the end. In the wall to your left are two exterior windows, about one-quarter and three quarters of the way along, looking to the outside. In the wall to your right, aligned with the first window, is an internal window, through which you glimpse the yellow-painted room. Further along the same wall, aligned with the second window, is a closed wooden door. At the end of the corridor you can see that it makes a right-hand turn.

You walk along the corridor to the end and go around this corner.

You find yourself in a blank stretch of corridor that has neither doors nor windows. You see that at the far end the corridor continues around a right-hand corner. You walk along the corridor to the far end and turn the corner.

You find yourself back in the corridor you first encountered when you entered the house. At the centre of the long wall to your left is the main door from which you entered the house, with the two windows on either side. In the long wall to your right are the internal window, and further along, the door.

You walk along the corridor until you reach the main door. You step through this door and back out of the house onto the street.
